# Acrolein-Mediated Conversion of Lysine to Electrophilic Heterocycles for Protein Diversification and Toxicity Profiling

**DOI:** 10.1021/jacs.4c12928

**Published:** 2025-02-07

**Authors:** Zachary E. Paikin, Benjamin Emenike, Rajendra Shirke, Christian Michel Beusch, David Ezra Gordon, Monika Raj

**Affiliations:** 1 Department of Chemistry, Emory University, Atlanta, Georgia 30322, United States; 2 Department of Pathology and Laboratory Medicine, Emory University, Atlanta, Georgia 30322, United States; 3 Department of Surgical Sciences, Uppsala University, Uppsala 751 85, Sweden

## Abstract

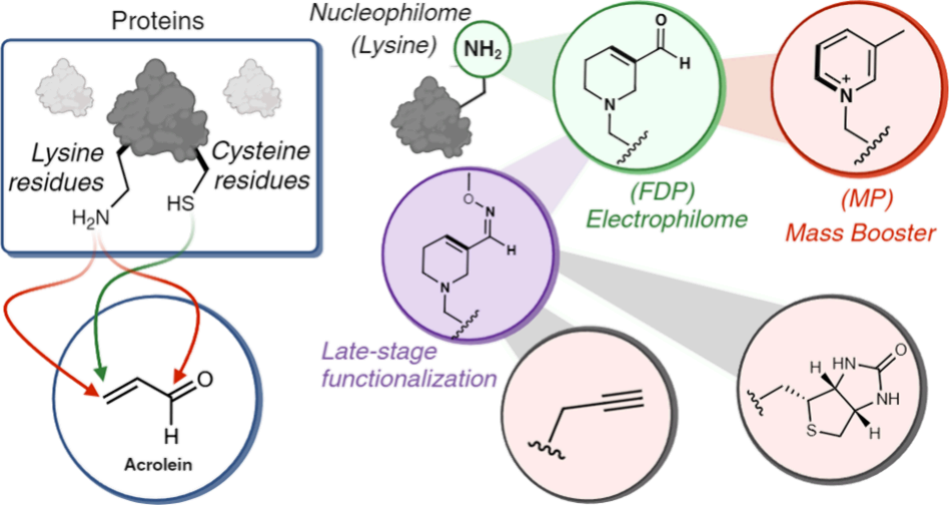

Understanding protein interactions in the presence of biological metabolites is critical for unraveling biological processes and advancing therapeutic interventions. This study focuses on α,β-unsaturated carbonyls, particularly acrolein-derived protein modifications, unveiling a one-pot, four-step, selective chemistry that results in the formation of a heterocyclic α,β-unsaturated carbonyl, termed 3-formyl-3,4-dehydropiperidino (FDP), exclusively on lysine residues. Remarkably, this chemistry transforms lysine, a nucleophile, into an electrophilic warhead. We demonstrate its versatility in late-stage peptide diversification, precision protein engineering, and homogeneous protein labeling with diverse payloads. Additionally, FDP-lysine smoothly transforms into another heterocycle, 3-methylpyridinium (3-MP) lysine via deoxygenation and aromatization in reagentless conditions. This transformation facilitates late-stage peptide functionalization and homogeneous engineering of proteins, with MP-lysine acting as a mass booster. Leveraging this chemistry, we discovered hyperreactive sites responsible for acrolein-induced modification through chemoproteomic profiling of FDP- and MP-modified proteins. Our findings revealed changes in protein–protein interactions mediated by FDP-modified proteins and uncovered ∼1548 novel cross-linking partners of an FDP-modified protein.

## Introduction

**Figure 1:**
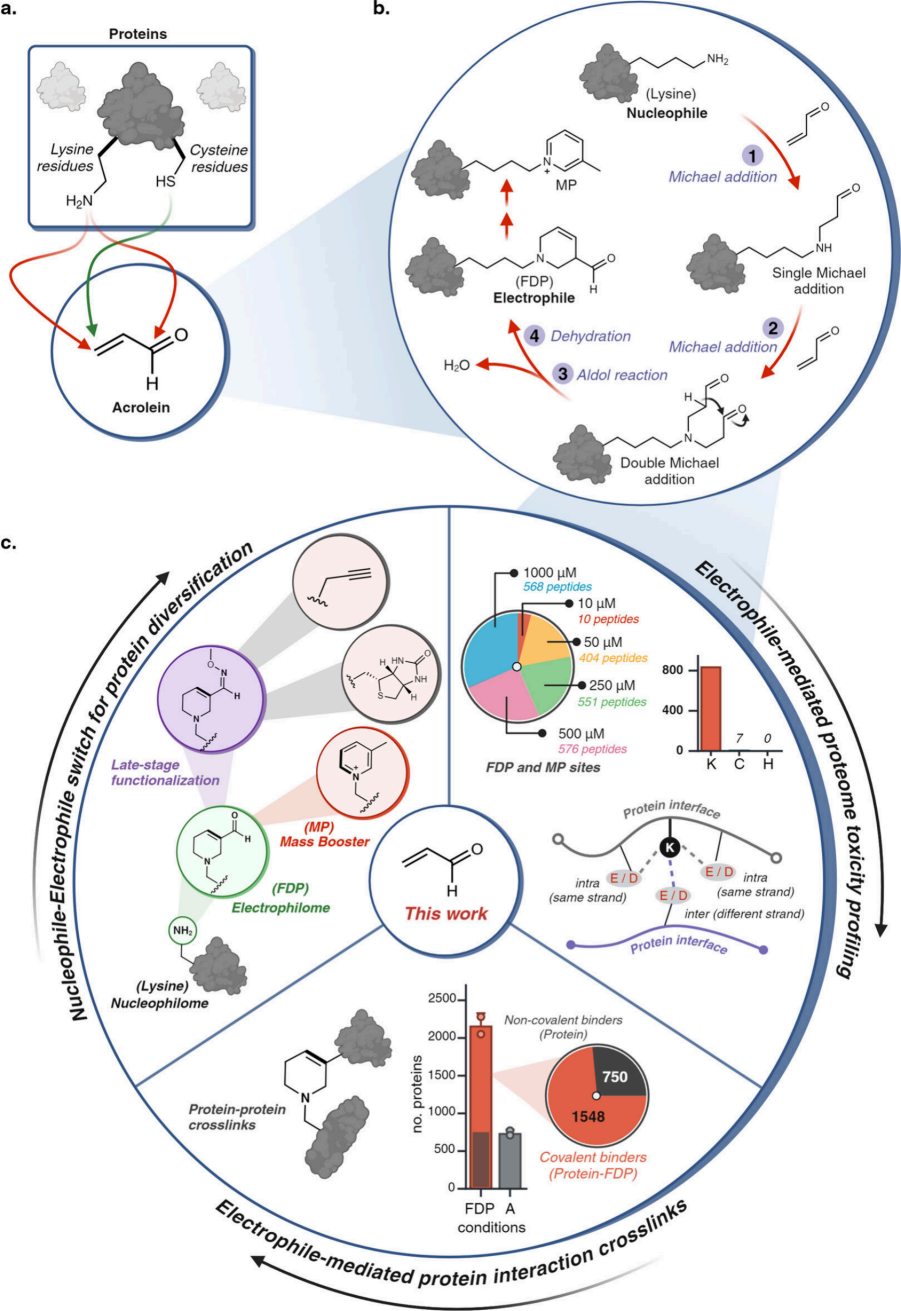
Bioinspired one-pot multistep reactions for selective modification of lysine into unique heterocycles. (a) Acrolein-mediated modification through reactions with nucleophilic residues such as lysine and cysteines on proteins. (b) Multistep reaction pathway involving two Michael additions to acrolein, followed by aldol and dehydration reactions to generate electrophilic heterocycle (FDP). FDP spontaneously generates 3-methylpyridinium (MP), thus introducing FDP, for the first time, as an intermediate for the formation of MP. (c) Selective modification of peptides and proteins to electrophilic heterocyclic warheads (FDP) and pyridinium heterocycles (MP) with mass-boosting properties, and selective labeling of lysine in a whole proteome. Chemoproteomics profiling of acrolein-exposed proteome led to the discovery of FDP- and MP-modified proteins, uncovering a unique acidic microenvironment around lysine residues that are most affected by acrolein-induced modification, in addition to the identification of protein–protein cross-links associated with electrophilic FDP warheads on proteins. Figure 1, created with BioRender.com, released under a Creative Commons Attribution-NonCommercial-NoDerivs 4.0 International license (Agreement number: RF272TFDG1).

Understanding the intricate interplay among peptides, proteins, and their binding partners is paramount in drug discovery and molecular biology.^1−3^ These interactions are dynamically influenced by biological metabolites, which can modify proteins through reactions at specific amino acid residues, leading to cross-linking with other biomolecules like DNA, RNA, lipids, and glycans (Figure 1a).^4−8^ Carbonyl compounds, including monocarbonyls, dicarbonyls, and α,β-unsaturated carbonyls, are reactive biological metabolites that play key roles in these modifications.^9−11^ However, existing methodologies such as mass spectrometry,^12,13^ SDS-PAGE,^14,15^ and antibody-based detection^16,17^ have limitations in identifying site-specific modifications and characterizing cross-linking events. Therefore, there is an urgent need for an innovative platform capable of efficiently addressing several key aspects: (i) identifying site-specific modification of proteins within their microenvironment, (ii) characterizing the type of protein modification, (iii) pinpointing modification sites, and (iv) determining the nature of cross-links between proteins.

Recently, our group and others have pioneered a platform for identifying protein modifications and cross-links induced by furan-derived metabolites, specifically cis-2-butene-1,4-dials (BDAs).^18,19^ In this study, we focus on α,β-unsaturated carbonyls, particularly acrolein. Acrolein, a highly toxic metabolite produced both endogenously during lipid peroxidation and inflammation, and exogenously from environmental pollutants such as cigarette smoke and industrial emissions, reacts with nucleophilic protein residues to form cross-links with other biomolecules.^20^ Exposure to acrolein has been implicated in various diseases, including neurodegenerative conditions,^21^ cardiovascular disease,^22^ diabetes,^23^ and cancer.^24^ Our research unveils a multistep, selective chemistry in which acrolein reacts with lysine residues through two Michael additions, followed by an intramolecular aldol reaction and dehydration, generating an electrophilic warhead exclusively on lysine residues through the formation of the 3-formyl-3,4-dehydropiperidino (FDP) moiety (Figure 1b).^25,26^ This FDP moiety can then transform into 3-methylpyridinium (MP) lysine via deoxygenation and aromatization under physiological conditions, providing a unique tool for tracking protein modifications (Figure 1b).^27,28^

This chemical platform enables the generation of an electrophilic warhead (FDP) from a nucleophilic lysine residue. We demonstrate the applications of FDP-lysine in late-stage peptide diversification and precision engineering of proteins with diverse payloads (Figure 1c). The resulting electrophilic FDP warhead on lysine reacts with other nucleophiles, facilitating protein cross-linking and the identification of acrolein-modified proteins and the binding partners of these modified proteins. Additionally, we illustrate the application of the formation of MP-lysine in late-stage peptide functionalization, as a mass booster, and in proteome profiling to identify proteins involved in acrolein-mediated MP-induced modification (Figure 1c). By offering high specificity and sensitivity in labeling modified proteins, this bioinspired approach provides a robust platform for elucidating complex biological networks, discovering novel protein biomarkers, identifying metabolite-mediated protein–protein interactions, and uncovering potential drug targets related to acrolein toxicity.

## Results and Discussion

### Design and Optimization of One-Pot Multistep Reaction with Unsaturated Aldehydes

**Figure 2:**
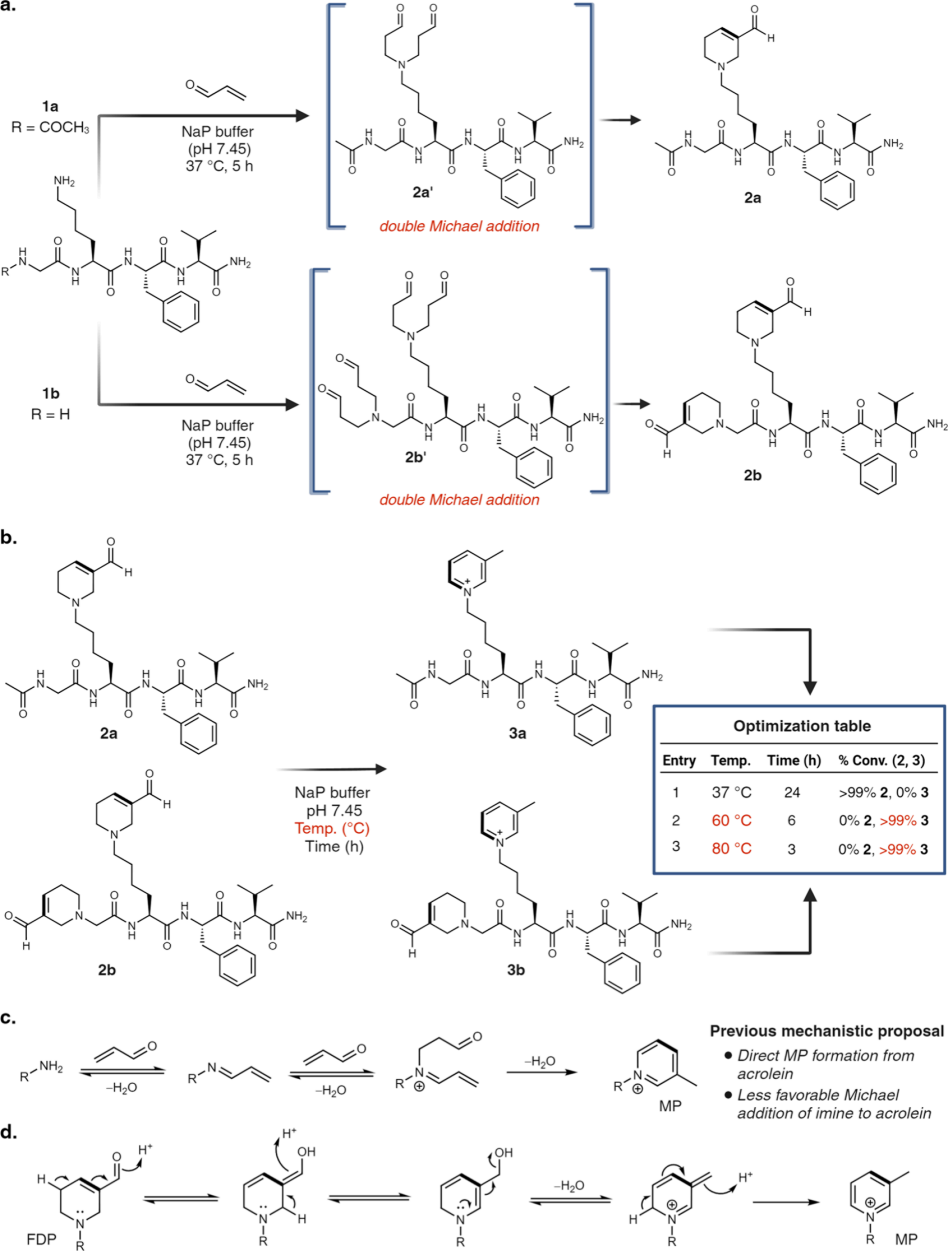
Development of one-pot multistep reaction with unsaturated aldehydes. (a) Reaction of lysine-containing peptide AcGKFV (1a) and free N-termini peptide GKFV (1b) with acrolein to generate heterocyclic FDP product on lysine (2a) and N-termini (2b). (b) Reagentless conversion of FDP-heterocycle to MP-heterocycle using FDP-containing peptides (2a, 2b) at 60 °C for 6 h (entry 2) or 80 °C for 3 h (entry 3). Interestingly, MP formation was not observed for N-terminal FDP. (c) Previous mechanism proposed in literature for formation of MP directly from lysine. In this mechanism, two molecules of acrolein add to lysine, first through Schiff base formation and then Michael addition. Morita–Bayllis–Hillman (MBH) reaction then results in cyclization and formation of the MP product. (d) New proposed mechanism for the formation of MP-lysine from FDP-lysine. Enol formation is followed by rearrangement, deoxygenation, and aromatization to furnish MP-lysine. Figure 2, created with BioRender.com, released under a Creative Commons Attribution-NonCommercial-NoDerivs 4.0 International license (Agreement number: PB272TFJXT).

In our pursuit to identify protein modifications and cross-links induced by α,β-unsaturated aldehyde metabolites and utilize this chemistry for protein engineering, we initially optimized the reaction using a lysine-containing model peptide, AcGKFV 1a. We subjected 1a to varying equivalents (2–5 equiv) of acrolein under physiological conditions (pH 7.45) at differing temperatures (RT – 37 °C) (Figure 2a, Supplementary Figure 1). We observed complete conversion of lysine to FDP 2a within 5 h at 37 °C using either 3 or 5 equiv of acrolein (Figure 2a, Supplementary Figure 1). Given the multistep nature of this reaction, including two Michael addition reactions on lysine followed by an aldol reaction and dehydration to generate FDP 2a, we aimed to optimize and capture reaction intermediates by analyzing the reaction at regular intervals. Interestingly, we did not observe the formation of the mono Michael addition product, primarily due to the higher nucleophilicity of the secondary amine compared to the primary amine. Instead, after 2 h, we detected small amounts of the double Michael addition product 2a’ (17%) alongside FDP product 2a (83%), which eventually converted completely to FDP in 5 h (Supplementary Figure 1). Next, we conducted the reaction on a peptide 1b GKFV, containing both a lysine and a free N-terminus, under the optimized conditions (5 equiv. acrolein, pH 7.45) at 37 °C. The reaction resulted in complete conversion to dual FDP product 2b by modifying both lysine and the N-terminus within 16 h (Figure 2a, Supplementary Figure 2). By reducing the amount of acrolein to 1.5 equiv on peptide 1b, we observed the formation of a single FDP product (57%) along with unreacted peptide (43%) (Supplementary Figure 2). These experiments suggested that once double Michael adducts form, they spontaneously convert to stable FDP products. Since the formation of FDP involves aldol chemistry, we attempted to carry out the reaction in the presence of proline as a catalyst^29^ and observed minor improvement in conversion to the FDP product at room temperature (Supplementary Figure 3). For further studies, proline was omitted as it did not improve reaction efficiency significantly. Next, we synthesized FDP-phenylalanine methyl ester on a larger scale and characterized the FDP product using NMR spectroscopy (Supplementary Figure 4).

In an attempt to accelerate the formation of FDP, we heated the reaction mixture containing peptide 1a to 60 and 80 °C. Surprisingly, deoxygenation followed by aromatization occurred under both conditions, resulting in the formation of 3-methylpyridinium heterocycle (MP) with lysine 3a, as confirmed by LCMS (Supplementary Figure 5). Attempts to synthesize MP directly from lysine on peptide 1a under mild conditions in a basic environment (pH 10.80) failed and gave FDP 2a as the only product (Supplementary Figure 5). Further investigations revealed that heating pure FDP-lysine peptide 2a in buffer without any reagents resulted in complete conversion to MP-lysine, indicating that MP is formed via an intermediate step involving FDP (Figure 2b, Supplementary Figure 5). This challenges the previously held belief that MP is directly formed from acrolein through a mechanism involving Schiff base formation between lysine and acrolein, followed by Michael addition and Morita Bayllis Hillman (MBH) reaction (Figure 2c, Supplementary Figure 6).^27^ The nucleophilic attack by an imine on acrolein, as proposed in the old mechanism, appears less favorable.^22^ Instead, we propose a new reaction pathway where MP is formed directly from the FDP product through enol formation, followed by rearrangement leading to deoxygenation and aromatization (Figure 2d, Supplementary Figure 6, new mechanism). Interestingly, heating the reaction mixture containing peptide 1b at 80 °C resulted in the exclusive formation of product 3b, where MP was formed only on the lysine side chain without conversion of N-terminal FDP to MP, even with prolonged reaction time (Figure 2b, Supplementary Figure 6). This observation suggests that FDP-lysine exhibits greater reactivity compared to N-terminal FDP, possibly due to the amide backbone chain reducing the ability of the FDP nitrogen to donate lone pair electrons and undergo aromatization to form MP, further supporting the proposed mechanism. We then synthesized MP on a small molecule, phenyl butylamine, and characterized it by NMR (Supplementary Figure 7).

### Substrate Scope of α,β-Unsaturated Aldehydes

For subsequent investigations, we directed our attention toward utilizing this chemistry to generate substituted FDP and MP derivatives with lysine. Equipped with the optimized conditions, we delved into the reaction’s versatility by exploring its compatibility with substituted α,β-unsaturated aldehydes, such as methacrolein. To our surprise, no FDP product was formed with methacrolein in a reaction with peptide 1a; instead, we only detected double Michael addition (52%) under the reaction conditions (Supplementary Figure 8). This outcome can be attributed to the resulting Michael adduct’s inability to undergo dehydration following the aldol reaction, which is crucial for stabilizing the FDP adduct. In contrast, the reaction with crotonaldehyde, featuring substitution at the beta position, hindered lysine’s nucleophilic attack, thus preventing the formation of any Michael adduct under the optimized conditions. Even upon elevating the reaction temperature to 80 °C, we observed poor conversion (24%) to substituted MP-lysine 3a’ after 24 h, as confirmed by LCMS analysis (Supplementary Figure 9). To enhance the conversion to substituted MP-lysine 3a’ and facilitate FDP-lysine generation, we introduced varying amounts of silver salts, such as silver oxide Ag_2_O and silver acetate AgOAc, to activate crotonaldehyde for nucleophilic attack by lysine for 1,4-addition.^30^ Additionally, we added Et_3_N as base while adjusting the temperature (40–80 °C) to promote the formation of the initial Michael addition product with crotonaldehyde. Despite these attempts, only 26% conversion to substituted MP-lysine 3a’ was achieved at 80 °C in the presence of silver acetate (Supplementary Figure 9). Based on these results, we proceeded with acrolein for further studies.

### Chemoselectivity Studies

**Figure 3:**
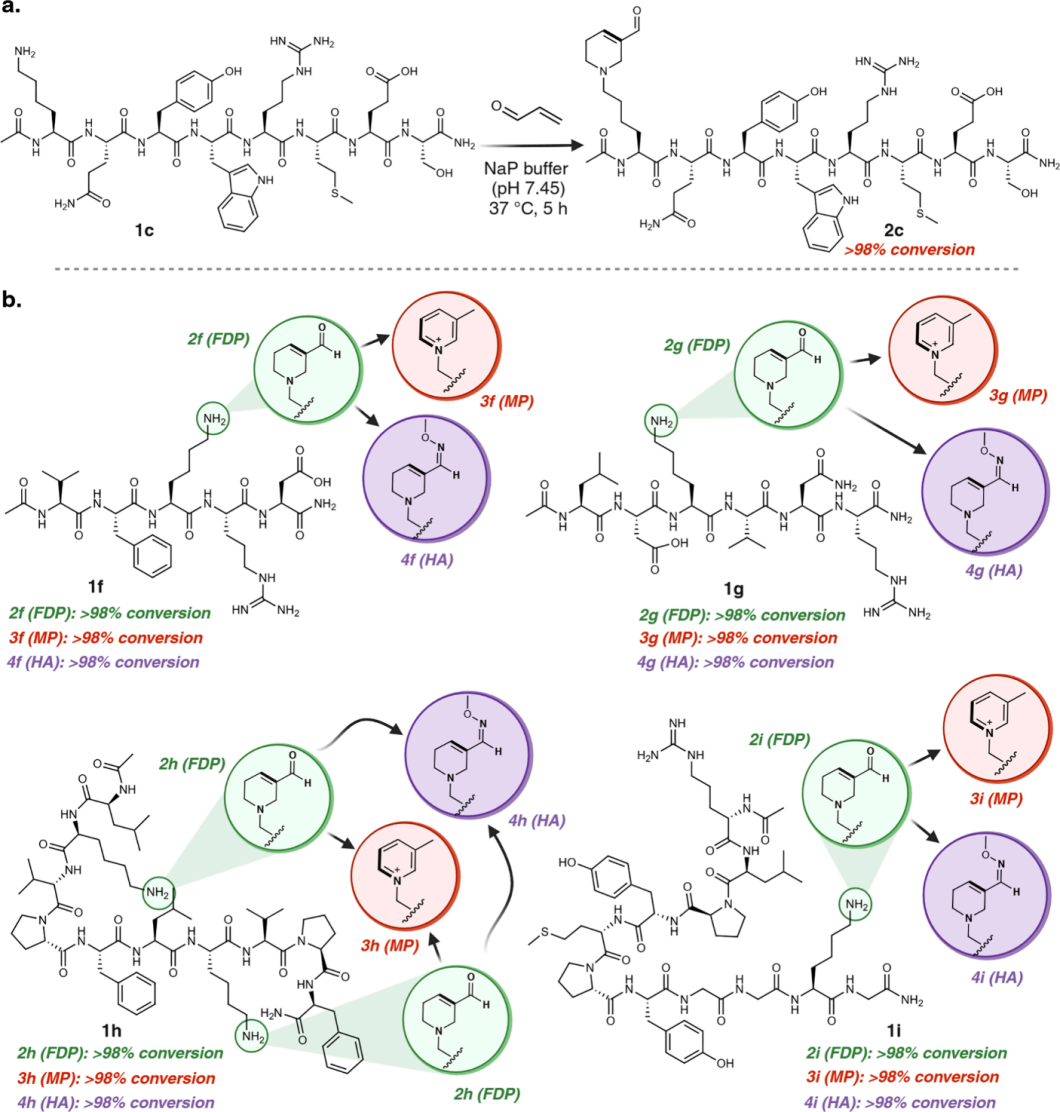
Chemoselective and late-stage functionalization of peptides and their further modifications. (a) Chemoselective modification of lysine peptide 1c to FDP product 2c in the presence of other reactive amino acids such as Tyr, Arg, Ser, Met, Trp, Asp, and Asn. (b) Late-stage functionalization of bioactive peptides containing lysine to FDP and MP, independent of chain length and amino acid sequence. Figure 3, created with BioRender.com, released under a Creative Commons Attribution-NonCommercial-NoDerivs 4.0 International license (Agreement number: HU272TFQKS).

Due to the pronounced electrophilicity of acrolein, we hypothesized its potential reactivity with other nucleophilic amino acids. To explore the specificity of lysine in generating FDP- and MP-lysine, we subjected a peptide Ac-KQYWRMES 1c, featuring reactive amino acids Tyr, Arg, Ser, Met, Trp, Glu, and Gln, alongside lysine, to acrolein under optimized reaction conditions (pH 7.45, 37 °C). Remarkably, the peptide converted >98% to FDP peptide product 2c with lysine within 5 h, without the formation of any side-adducts with other amino acids (Figure 3a, Supplementary Figure 10). To confirm the enhanced reactivity of lysine toward acrolein compared to histidine, we incubated two peptides, AcGKFV 1a and AcGHFV 1d, in equal amounts with a limited quantity of acrolein (2 equiv). Notably, we observed modification exclusively in the lysine peptide, resulting in the formation of FDP-lysine 2a, with no formation of 1,4-addition products with histidine (Supplementary Figure 11). Given the higher prevalence of lysine in the proteome, in addition to higher reactivity relative to histidine, we did not anticipate any cross-reactivity with histidine during protein lysine modification under the reaction conditions. As expected, during an analogous experiment using limited acrolein (2 equiv), we observed the formation of 1,4-single addition product 2e with the cysteine peptide AcGCFV 1e in the presence of lysine AcGKFV 1a (Supplementary Figure 12). However, with cysteine being the least abundant amino acid residue, in addition to its occurrence in disulfide bonds stabilizing the tertiary structures of proteins, we do not anticipate significant chemoselectivity issues. Additionally, the formation of FDP and MP products is unique to lysine, which further enhances the specificity of the reaction. To further improve chemoselectivity, prior labeling of cysteine residues with thiol-reactive probes can be employed, ensuring that the reaction selectively targets lysine modifications without interference from cysteine. This property enables further functionalization to differentiate acrolein-modified cysteine handles from acrolein-modified lysine, FDP.

### Substrate Scope and Late-Stage Functionalization of Peptides

Due to the remarkable specificity observed in forming FDP products with lysine, our subsequent efforts were directed toward synthesizing FDP-lysine on various linear peptides of diverse sizes and amino acid compositions, with lysine positioned variably. Employing Fmoc-solid-phase peptide synthesis, we generated several bioactive linear peptides, including Ac-VFKRD 1f, Ac-LDKVNR 1g, Ac-LKVPFLKVPF 1h, and Ac-RLPYMPYGGKG 1i, some exhibiting antihypertensive 1f,^31^ anti-inflammatory 1g,^32^ and anticancer 1i^33^ properties. These peptides were subjected to optimized reaction conditions. In all peptides, lysine successfully formed FDP, producing products 2f-2i with high conversion (>98%), without generating any byproducts involving other reactive amino acids, including Tyr, Arg, Asp, Met, and Asn (Figure 3b, Supplementary Figure 13). Notably, peptide Ac-LKVPFLKVPF 1h containing two lysines yielded the doubly modified FDP product 2h (>98%) in the presence of excess acrolein (5 equiv) and selectively formed a single-modified FDP product 2h’ (61%) when using a limited quantity of acrolein (2 equiv), demonstrating remarkable control for modification of a single lysine to FDP once the multistep reaction with acrolein begins (Figure 3b, Supplementary Figure 13). These examples highlight the exceptional precision of this reaction in selectively modifying lysine to FDP, irrespective of the presence of other amino acids, rendering it ideal for late-stage peptide functionalization. Having installed FDP-lysine onto peptides, we proceeded to diversify them through a chemoselective reaction, utilizing oxime chemistry due to its widespread application in medicinal chemistry.^34^ FDP peptide 2a, as well as bioactive FDP peptides 2f-2i, including peptide 2h containing two FDP groups, were diversified through reactions with aminoxy-functionalized molecules, specifically *o*-2-methoxyhydroxylamine, in aqueous buffer at physiological pH (7.45). We observed full conversion (>98%) to oxime-conjugation products 4a and 4f-4i, as confirmed by LCMS analysis (Figure 3b, Supplementary Figure 14). Notably, the addition of excess methoxyamine resulted in the formation of a double addition product, with modification at the 1,4 position along with 1,2-oxime addition (Supplementary Figure 14).

Subsequently, we heated peptides 1f-1i to 60 °C with acrolein in buffer (pH 7.45) and observed complete conversion to the corresponding MP-lysine peptides 3f-3i through FDP as an intermediate (Figure 3b, Supplementary Figure 15). Given that this transformation generates pyridinium, a heterocyclic pharmacophore, from lysine, we anticipate that this strategy could be utilized in the synthesis and incorporation of this essential pharmacophore on small molecules and peptide-based therapeutics.^35−37^

### Substrate Scope for Homogeneous Labeling of Proteins

**Figure 4:**
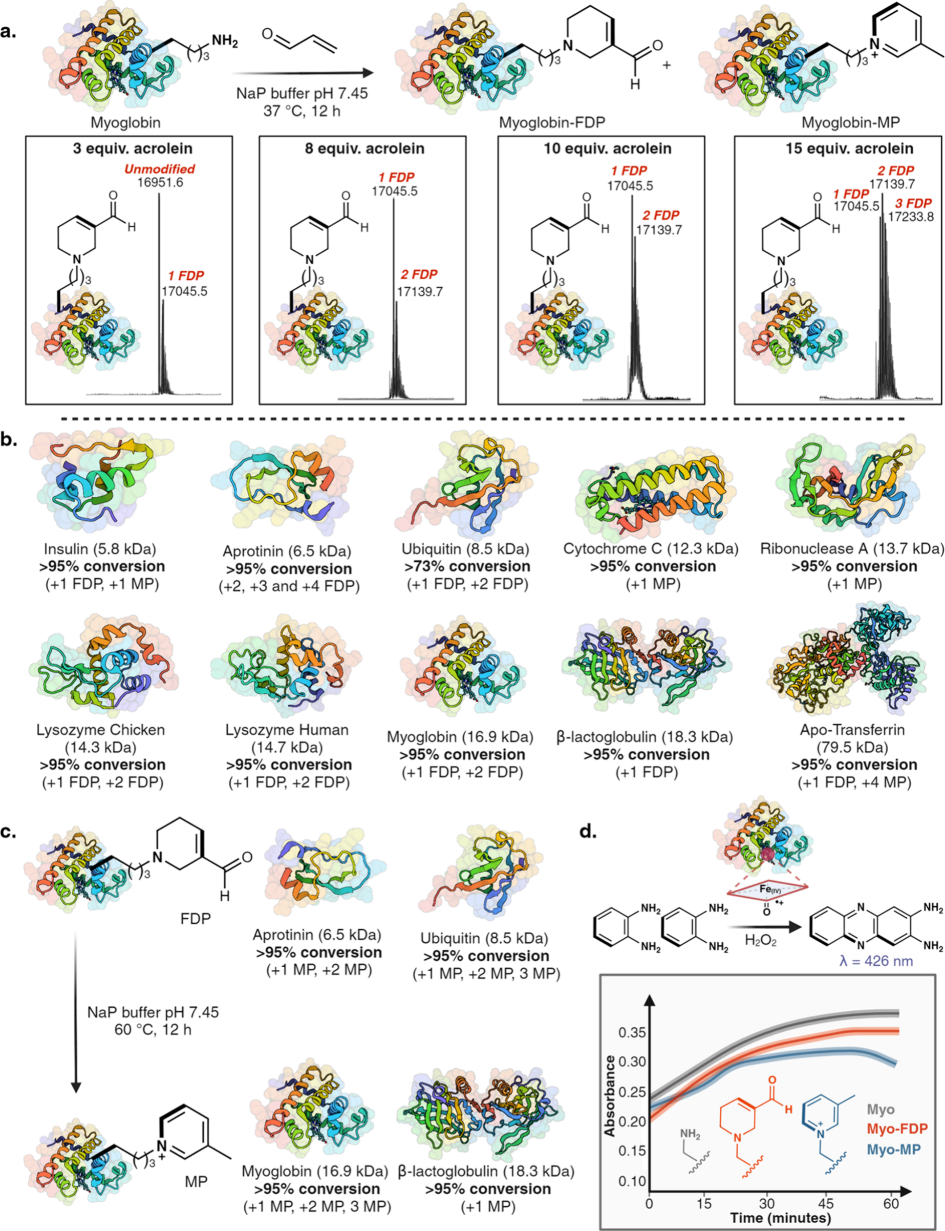
One-pot multicomponent modification of proteins to heterocycles. (a) Dose-dependent increase in the labeling of lysine to FDP on myoglobin with varying equivalents of acrolein (3–15 equiv). Full conversion to homogeneous labeling product was observed by using 8 equiv of acrolein, indicating the reaction’s high robustness and efficiency. (b) Chemoselective modification of lysine on proteins of varying sizes (5.8–79.5 kDa) and 3D-structures with high conversions in all the cases under optimized conditions as analyzed by MS. (c) Homogeneous modification of four FDP-proteins (aprotinin, ubiquitin, myoglobin, and β-lactoglobulin) to MP-proteins. (d) FDP-modified myoglobin under optimized condition demonstrates similar ability to oxidize *o*-phenylenediamine compared with unmodified myoglobin. These data support the hypothesis that the 3D structure of the myoglobin remains intact after the modification. Figure 4, created with BioRender.com, released under a Creative Commons Attribution-NonCommercial-NoDerivs 4.0 International license (Agreement number: HY272TG4SF).

Next, we proceeded to assess the potential of this reaction for the chemoselective modification of lysine on proteins. We initiated our experiments using a model protein, myoglobin with uniprot ID (1MBN), containing 19 lysines, by varying the equivalents of acrolein (3–15 equiv). Remarkably, with 8 equiv of acrolein, we efficiently modified >95% of myoglobin, generating a homogeneous FDP product with selective labeling of only one lysine among 18 other lysines in myoglobin (Figure 4a, Supplementary Figure 16). Despite myoglobin having 19 lysines, only a few underwent modifications at low reagent equivalents. The MS/MS analysis of myoglobin, dominated by single lysine modification, obtained by adding 8-equivalents of acrolein, showed K63 as the modification site (Supplementary Figure 16). Following purification, incubation of the modified myoglobin over a pH range of 3 to 11 at 37 °C demonstrated the full stability of FDP over a 24-h period (Supplementary Figure 17). This outcome confirms the high stability of FDP under physiological conditions, an essential feature required for its utilization as an electrophilic warhead for downstream conjugation of reactive nucleophilic affinity tags, capturing of protein partners, and profiling of proteins potentially associated with acrolein toxicity through modification with varying amounts of acrolein.

With the optimized conditions established for the selective homogeneous modification of myoglobin, we proceeded to apply this protocol to nine different commercially available protein substrates of varying molecular weights (5.8 kDa-79.5 kDa) and different three-dimensional architectures, including insulin, aprotinin, ubiquitin, cytochrome C, ribonuclease A, lysozyme chicken, lysozyme human, β-lactoglobulin, and apo-Transferrin (Figure 4b, Supplementary Figure 16). Homogeneous labeling was observed in all cases. The mild conditions of this reaction preserved structurally critical disulfide bonds in insulin. MS/MS analysis of the modified proteins clearly revealed lysine modification without any modification of histidine (Figure 4b, Supplementary Figure 17). Interestingly, we noted the formation of MP-lysine with certain proteins such as insulin, cytochrome C, and ribonuclease-A under the reaction conditions, suggesting a unique microenvironment might favor the spontaneous deoxygenation and aromatization of FDP-lysine to MP-lysine (Figure 4b, Supplementary Figure 16). Furthermore, we also observed the transformation of FDP sites to MP sites during proteolytic digestion and LCMS/MS analysis (Supplementary Figure 16). Since the transformation of FDP to MP is temperature mediated, we attribute this observation to the digestion protocol, which involves denaturing protein samples at 95 °C. Next, we converted a few FDP-modified proteins such as aprotinin, ubiquitin, myoglobin and β-lactoglobulin to MP-modified proteins by incubating them at 60 °C and observed full conversion of FDP sites on proteins to MP in all cases (Figure 4c, Supplementary Figure 18). The high preference for labeling particular lysines among others signifies the utility of this approach for activity-based protein profiling (ABPP) in identifying proteins and sites more susceptible to modification under oxidative stress conditions in which acrolein levels are elevated.

### Protein Function Is Retained after Modification

To explore the ability of our method to modify a protein without altering its tertiary structure, we examined the activity of FDP- and MP-modified homogeneous myoglobin by its ability to carry out the oxidation of *o*-phenylenediamine with hydrogen peroxide to 2,3-diaminophenazine as monitored at 426 nm by UV spectroscopy.^38^ Negligible change in the UV signal was observed with FDP-modified myoglobin as compared to unmodified myoglobin (Figure 4d, Supplementary Figure 19). As expected, heating at 60 °C for the formation of MP slightly reduced the activity of MP-modified myoglobin (Figure 4d). These results highlight the ability of this multistep chemistry to enable efficient and selective modification of proteins without denaturation, conserving their structure and bioactivity.

### Selective Labeling of Proteins with Varying Warheads

**Figure 5:**
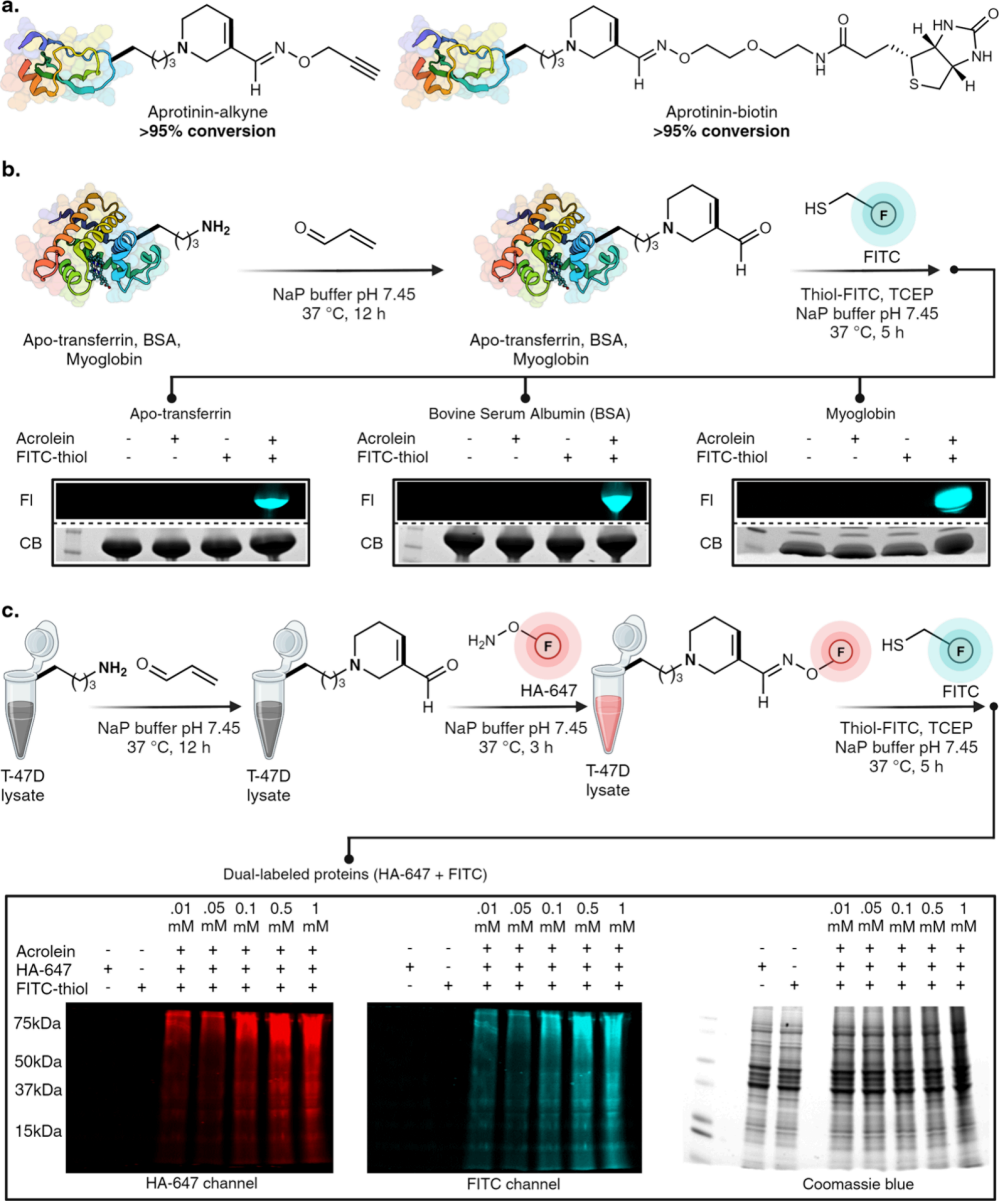
Selective labeling of FDP-modified proteins with varying warheads. (a) Late-stage functionalization of FDP-modified aprotinin with varying affinity tags using aminoxy-alkyne and aminoxy-biotin analogs. (b) Thiol-FITC labeling of FDP-modified apo-transferrin, bovine serum albumin (BSA), and myoglobin. No fluorophore labeling was observed in control experiments in the absence of acrolein or thiol FITC. Fl = fluorescence, CB = Coomassie Blue. (c) Dual labeling of FDP-proteins in cell lysate with two different fluorophores by two different chemistries, oxime with hydroxylamine fluorophore and 1,4-addition with cysteine fluorophore, and their analysis by in-gel fluorescence. Figure 5, created with BioRender.com, released under a Creative Commons Attribution-NonCommercial-NoDerivs 4.0 International license (Agreement number: BO272TGAHH).

Encouraged by the high efficiency and selectivity in lysine labeling, we demonstrated the application of our approach in the selective labeling of native proteins with varying warheads such as affinity tags and fluorophores. To conjugate affinity tags onto FDP proteins, FDP-modified aprotinin was treated with aminoxy-alkyne and aminoxy-biotin analogs, and full conversion to the oxime-modified alkyne- and biotin-derivatized aprotinin was observed (Figure 5a, Supplementary Figure 20). Next, we labeled electrophilic FDP-proteins with fluorophores by reacting native proteins such as apo-Transferrin, BSA, and myoglobin with acrolein under optimized conditions for 12 h, followed by incubation with thiol-FITC for 1,4-addition to the resulting FDP-lysine on proteins at room temperature. Purification of proteins and in-gel fluorescence analysis clearly showed the formation of FDP-lysine and labeling of Apo-transferrin, BSA, and myoglobin with FITC fluorophore (Figure 5b, lanes 4, Supplementary Figure 21). No fluorophore labeling was observed in control experiments in the absence of acrolein or thiol FITC (Figure 5b, lanes 1–3, Supplementary Figure 22). While various electrophiles can label nucleophilic lysine, such as activated esters,^39^ sulfonyl chlorides,^40^ isothiocyanates,^41^ or alkylation,^42^ none have shown the ability to generate another electrophilic warhead on lysine with such exceptional efficiency (>95%) regardless of protein size and 3D structure under mild and dilute reaction conditions (∼8 μM). The precise and efficient formation of α,β-unsaturated electrophilic warheads on lysine is remarkable and broadens its potential applications, including dual protein modification with different functional tags. To demonstrate this application, we modified T-47D cell lysates with acrolein to incorporate FDP sites on proteins. We then performed oxime chemistry with hydroxylamine-647 dye, followed by a 1,4-addition reaction with thiol-FITC. This resulted in the dual labeling of proteins in cell lysates with two distinct fluorophores (Figure 5c, Supplementary Figure 22). In-gel fluorescence analysis confirmed the dual labeling of cell lysate with FITC and alexafluor-647 dye, demonstrating the utility of the orthogonal reactive handles on FDP for incorporating diverse tags onto proteins. These results open avenues for combining drug molecules with imaging agents to design precise protein therapeutics.

### Identification of Acrolein-Modified Proteins by Activity-Based Protein Profiling

**Figure 6:**
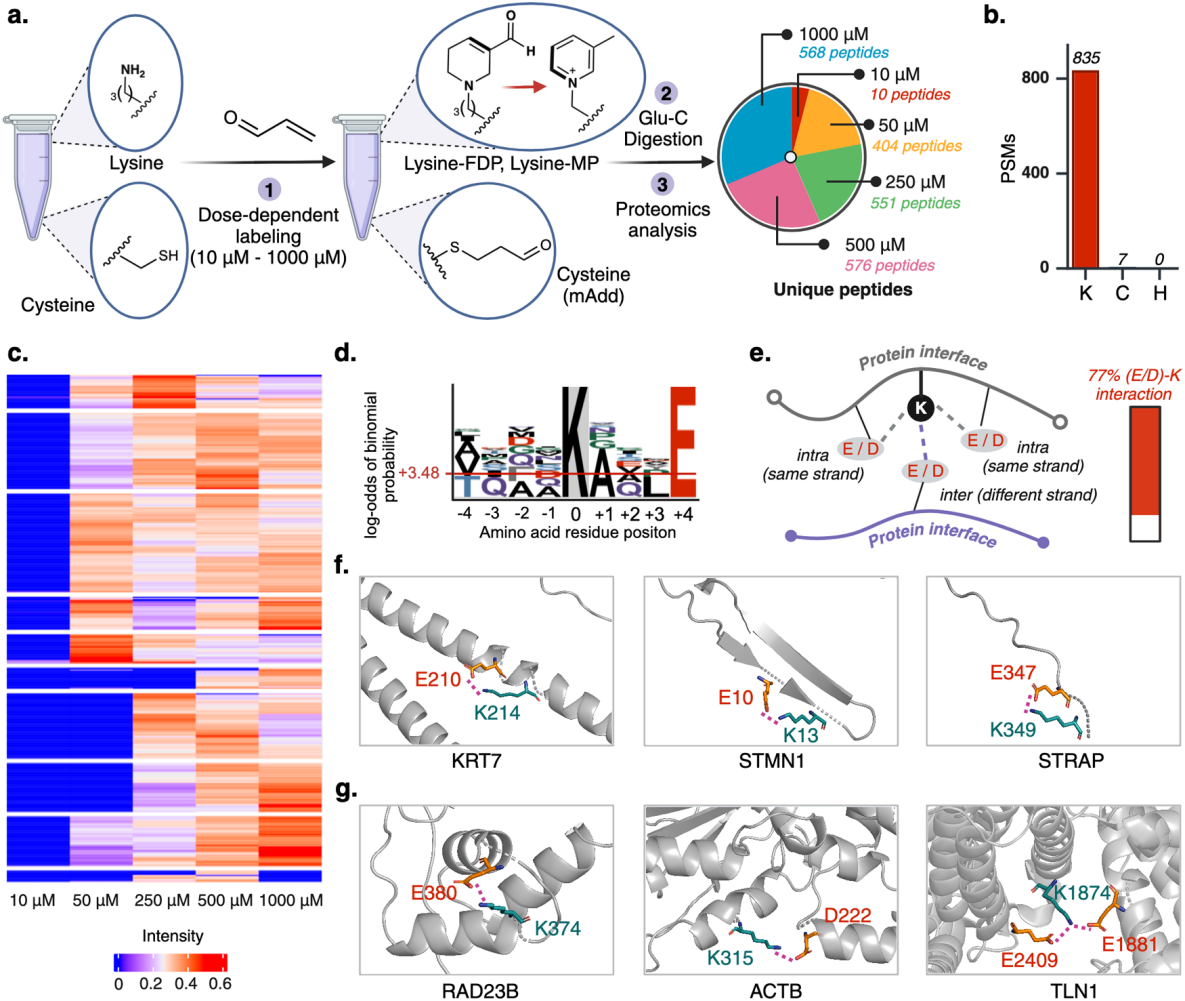
Mechanism-based activity-based protein profiling (MABPP) of proteome associated with acrolein modification. (a) Schematic representation of the formation of FDP- and MP-modified proteins from acrolein-treated cell lysate samples followed by subsequent analysis by LCMS/MS. Analysis of proteomics results showed a dose-dependent generation of FDP and MP adducts on lysine. Excel sheet of analysis is included as Supplementary Data 1. (b) High selectivity of acrolein for lysine, as significant modification of lysine was observed with only 7 modifications of cysteine and no modification of histidine. (c) Heatmap visualization of FDP- and MP-modified peptides confirmed a dose-dependent modification of lysine, with a greater ratio of modified to unmodified peptides as acrolein concentration increased (250 to 1000 μM). (d) Sequence motif analysis of the FDP- and MP-modified peptides demonstrates an overrepresentation of acidic residues (D, E) along with aliphatic residues (A, V, L). (e) Analysis of PDB structures of 100 FDP and MP sites showed electrostatic stabilization by D or E in the vast majority of modified lysine residues (77%). (f) Representative examples of intrastrand (D/E) interaction with lysine in α-helical structures (KRT7), β-sheets (STMN1), and random coiled structure (STRAP). (g) Representative examples of interstrand (D/E) interaction with modified lysine sites (RAD23B, ACTB, TLN1). The data utilized for analysis of (a)–(g) were acquired with *n* = 2 biological independent samples. Excel sheet of analysis is included as Supplementary Data 1 and 2. Source data are provided as a Source Data file. Figure 6, created with BioRender.com, released under a Creative Commons Attribution-NonCommercial-NoDerivs 4.0 International license (Agreement number: IY272TD0GU).

To elucidate toxicity via acrolein-modified proteins, we established a Mechanism-Based Activity-Based Protein Profiling (MABPP)^19,43−45^ platform using human breast cancer cells (T-47D). We incubated lysates with varying amounts of acrolein, representing physiological and pathological concentrations (Figure 6a, Supplementary Figure 23). Our approach aimed to pinpoint modification sites without alkynylation or azidation of acrolein, as these modifications may alter the bioactivity of the original compounds. Proteomic analysis of digested lysate revealed a dose-dependent identification of unique FDP-lysine labeled peptides (10 at 10 μM; 404 at 50 μM, 551 at 250 μM, 576 at 500 μM, 568 at 1000 μM) (Figure 6a, Supplementary Figure 23, Supplementary Data 1). Notably, the majority of identified sites were MP, likely converting from FDP during sample digestion for LCMS/MS analysis as observed with previous results with recombinant proteins (see Supplementary Figure 16). Our study revealed two primary chemical reactions involved in acrolein-derived protein adduction: formation of FDP and MP adducts with lysine and Michael addition adducts with cysteine (mAdd). Interestingly, evaluation of FDP and MP modifications on amino acid residues clearly identified lysine as the major site of modification with 835 lysine-modified peptides and only 7 cysteine-modified peptides (Figure 6b, Supplementary Figure 23, Supplementary Data 1). No modification of histidine was observed under protein profiling conditions, thus confirming high chemoselectivity for lysine. Heat map analysis of the modified FDP-lysine sites demonstrated a greater ratio of modified to unmodified proteins at higher concentrations (50–1000 μM), further confirming the dose-dependent generation of FDP and MP sites with acrolein (Figure 6c, Supplementary Figure 23).

We evaluated the sequence motif surrounding FDP and MP sites using *Plogo* map tool,^46^ which revealed a significant abundance of glutamic acid (E) at +4 position and aspartic acid at −1 and −2 positions, although to a slightly lesser extent compared to E (Figure 6d, Supplementary Figure 23). This result suggests a sequence motif for lysine residues most likely to be modified by acrolein. To elucidate the potential microenvironment-induced reactivity of modified lysine residues, we analyzed the sequence motif of recombinant proteins that were labeled with acrolein in Figure 4b. Of the 11 sites that were modified in cytochrome C, lysozyme chicken, myoglobin, β-lactoglobulin, and apo-transferrin, 9 possessed E or D in close proximity to the modified lysine residues (81%) (Supplementary Figure 23, Supplementary Data 2). A similar observation was seen when we profiled 100 sites containing FDP or MP modification and observed that 77% of sites possess D or E residues within electrostatic and hydrogen bonding interaction distance (<3.2 Å) of a modified lysine (Figure 6e, Supplementary Figure 23, Supplementary Data 2). The interactions were observed with D or E residues both on the same strand as the modified lysine (intra, Figure 6f) and on a different strand (inter, Figure 6g). Based on these observations, it is plausible to propose that electrostatic interactions mediate nucleophilic activation of lysine residues to undergo the two Michael additions with acrolein enroute to FDP or MP. Taken together, this observation suggests that lysine residues flanked by a rich distribution of acidic residues such as D or E are more prone to acrolein modification. To assess the impact of acrolein modification on key biological pathways, we performed Gene Ontology (GO),^47,48^ Molecular Function (MF), Biological Process (BP), Cellular Compartment (CC), KEGG, REACTOME, and WikiPathways analyses. These analyses showed that FDP- and MP-modified proteins are involved in diverse pathways such as cellular stress response, protein folding, cytoskeletal organization, methylation, and metabolic pathways (Supplementary Figure 23). These findings highlight the broad impact of acrolein modification, consistent with existing literature,^49−56^ suggesting a complex mechanism underlying its toxic effects. Further analysis is needed to fully understand the biological processes affected.

### Platform for Increasing the Sensitivity of the Acrolein-Modified Proteome

**Figure 7:**
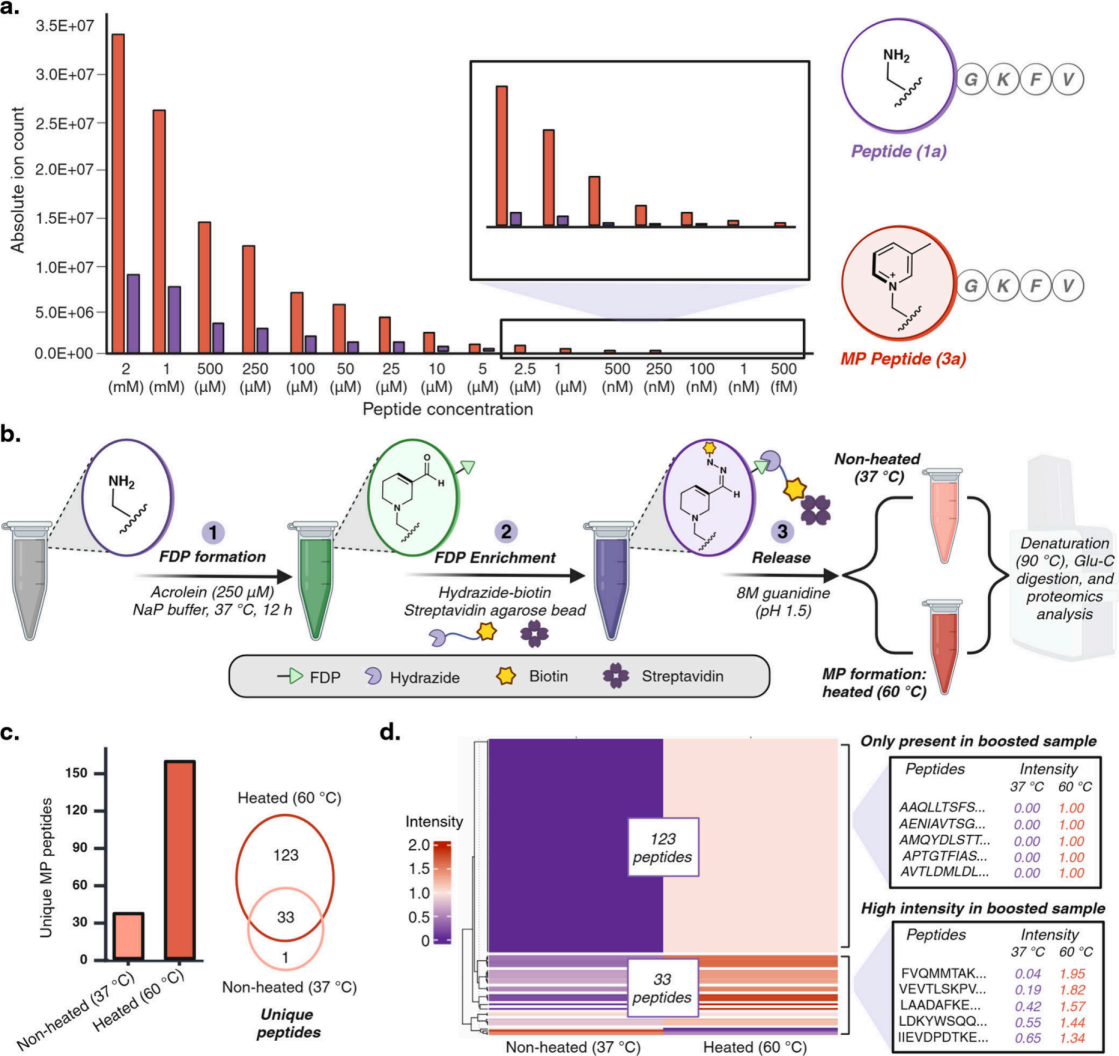
Platform for increasing the sensitivity of the acrolein-modified proteome. (a) MP-modified peptide exhibits high mass-boosting sensitivity, with detection of the MP peptide at femtomolar concentrations and greater ionizability of the MP peptide at all concentrations. (b) Schematic representation of proteome-wide mass-boosting of acrolein-modified lysine sites. Cell lysates are incubated with acrolein to generate FDP followed by reaction with biotin-hydrazide and enrichment with streptavidin functionalized agarose bead. Enriched proteins are released with 8 M guanidine hydrochloride followed by MP formation by heating of proteins at 60 °C for 12 h. Proteins are digested followed by LCMS-MS analysis. (c) Unique peptides quantification of MP peptides in nonheated samples (37 °C) and heated samples (60 °C). One unique MP site was observed in nonheated sample, 123 unique peptides in heated sample, and 33 peptides between the heated and nonheated samples. (d) Heatmap visualization of nonheated (37 °C) and heated (60 °C) samples confirm the MP-mediated mass-boosting of 123 peptides not previously observed in nonheated sample. Additionally, MP led to an increased intensity of peptides observed in nonheated samples. Excel sheet of analysis is included as Supplementary Data 3. Source data are provided as a Source Data file. Figure 7, created with BioRender.com, released under a Creative Commons Attribution-NonCommercial-NoDerivs 4.0 International license (Agreement number: BI27L1PWWT).

Given that this transformation generates pyridinium ions from lysine, we anticipated that these molecules would serve as mass boosters, enhancing peptide detection by multiple orders in mass spectrometry.^57^ To evaluate this, we measured the mass-enhancing properties of MP by incubating MP-modified peptide 3a with unmodified peptide 1a. The MP modification allowed for the detection of 3a at femtomolar concentrations and produced approximately a 10x increase in ionizability at higher concentrations where both peptides were detectable (Figure 7a, Supplementary Figure 24).

We next utilized the mass-boosting properties of MP to enhance the sensitivity of acrolein-modified lysine residue detection across the proteome. We hypothesized that enriching for FDP-modified proteins and converting them to MP would improve detection sensitivity. Initial experiments focused on optimizing this strategy with a model FDP-modified protein. FDP-modified aprotinin was labeled with biotin-hydrazide, then heated at 60 °C for 12 h, resulting in 61% conversion to MP-modified aprotinin after detachment of the hydrazide (Supplementary Figure 24). Following this successful demonstration, we applied the strategy to cell lysates. T-47D cell lysates were modified with 250 μM acrolein and then treated with 200 μM biotin-hydrazide. Biotinylated proteins were enriched using streptavidin-functionalized agarose beads and eluted with 8 M guanidine hydrochloride. The eluted proteins were divided into two fractions: one was heated at 60 °C for 12 h to generate MP-modified proteins. Both fractions were then processed for digestion: the samples were heated at 90 °C for 10 min, followed by Glu-C digestion, peptide quantification, and LC-MS/MS analysis (Figure 7b, Supplementary Figure 24, Supplementary Data 3). Comparison of the heated and nonheated samples revealed 156 unique MP peptides in the heated sample, 1 MP peptide in the nonheated sample, and 33 MP peptides in both (Figure 7c, Supplementary Figure 24, Supplementary Data 3). The MP peptides in the nonheated sample were likely due to the conversion of FDP to MP during the denaturation process at 90 °C. Notably, we did not observe FDP-modified fragments in the proteomic analysis of either fraction, possibly due to the low ionization efficiency of FDP in MS. The additional 123 MP-modified sites observed in the heated sample were a result of the conversion of FDP to MP, which enhanced mass detection (Figure 7d). Moreover, the intensity analysis of peptides from both heated and nonheated samples showed significantly higher intensities in the heated samples, reflecting the increased conversion of FDP to MP (Figure 7d). These findings demonstrate the utility of MP as a mass booster, a powerful tool for improving the detection of low-abundance proteins.

### Identification of FDP-Induced Protein Cross-Links

We next sought clarify the mechanism of acrolein toxicity through protein cross-linking, as mediated by FDP-modified proteins. We envisioned utilizing the electrophilic FDP warhead on proteins as a linchpin for capturing protein–protein cross-links caused by acrolein toxicity, which occurs via lysine and cysteine residues of binding partners forming reversible or irreversible covalent bonds with FDP. As a proof of concept, we evaluated the formation of Schiff base between FDP on aprotinin and propargylamine followed by reduction with NaBH_3_CN (Supplementary Figure 25). Upon successful formation of the amine coupled product, we proceeded to incubate an FDP-modified protein, aprotinin, with its known binder (chymotrypsin) to cross-link them through covalent bonds with cysteine or lysine residues on chymotrypsin.

Subsequently, to convert the reversible covalent bond between the FDP of aprotinin and lysine of chymotrypsin to an irreversible bond, we introduced a reducing agent (NaBH_3_CN). The results demonstrated the successful covalent cross-linking of aprotinin with chymotrypsin, as validated through gel electrophoresis and mass spectrometry analysis (Supplementary Figure 25). We observed higher-ordered cross-links indicative of chymotrypsin-aprotinin interaction (-hetero and -poly cross-links). The results also confirmed the homo-crosslinking of FDP-aprotinin, showcasing nonspecific binding (Supplementary Figure 25). This might be due to the presence of the highly reactive electrophilic FDP-moiety. In a validation experiment using lysozyme chicken, cross-linking was also observed between chymotrypsin and the FDP-modified lysozyme chicken, two nonbinding partners used as a control, indicating that the influence of nonspecific cross-linking has not been eliminated (Supplementary Figure 25). We further incubated FDP-aprotinin with T-47D cell lysate and compiled the extensive list of candidate proteins binding to FDP-aprotinin (1548 protein binders, Supplementary Figure 26, Supplementary Data 4). Detailed biological studies of these proteins suggest a broad range of potential targets for acrolein-derived modifications (Supplementary Figures 27 and 28, Supplementary Data 5 and 6). Further studies are required to determine the significance of these FDP-aprotinin protein cross-links, which is beyond the scope of this manuscript.

## Conclusion

Motivated by the toxicity elicited by acrolein-mediated protein modification and cross-linking within cellular environments, we devised a robust and highly efficient one-pot multistep chemoselective reaction for lysine modification with acrolein. This reaction yields stable heterocyclic products, namely FDP-lysine and MP-lysine derivatives, showcasing exceptional reactivity due to the intricately orchestrated interplay between acrolein and lysine. Sequentially, this process involves four distinct steps: two Michael additions, one aldol reaction, and subsequent dehydration, all occurring under mild conditions and without necessitating catalysts. The resulting FDP-lysine adduct introduces an electrophilic warhead, enabling further functionalization with diverse nucleophiles. Intriguingly, contingent upon the microenvironment of lysine within proteins, it undergoes additional deoxygenation and aromatization steps in physiological conditions, yielding highly stable 3-methylpyridinium adducts (MP). This pyridinium pharmacophore can also be generated through gentle heating of any FDP-lysine, a unique and reagentless transformation.

This reaction demonstrates a broad substrate scope, facilitating clean conversion to stable FDP and MP adducts with lysine, regardless of peptide amino acid sequence. Expanding on this, we derivatized peptides containing the FDP-lysine electrophilic warhead with nucleophiles, including hydroxylamines via 1,4- or 1,2-additions. MS analysis revealed heightened sensitivity of MP-modified lysine compared to native peptides and proteins. We homogeneously modified ten different proteins of diverse sizes and 3D structures with remarkable efficiency, underscoring acrolein’s capacity to discern subtle reactivity differences among lysines on native proteins, an attribute invaluable for protein engineering and Antibody-Drug Conjugate (ADC) synthesis.

We deployed this method for a metabolite-based chemoproteomic platform to elucidate proteins adducted by reactive acrolein, generated endogenously due to oxidative stress and inhaled exogenously from environmental pollution. This platform facilitated the identification of novel acrolein-modified proteins and lysine sites containing FDP or MP. Using this precise method, we were able to demonstrate that lysine flanked by negatively charged residues may be more susceptible to acrolein modification, a finding with broad utility in better understanding this pollutant. We additionally explored the mass-boosting capabilities of MP on a proteome level, increasing the detection level of acrolein-modified proteins by multiple orders. We attempted to capture the protein binding partners of FDP-modified aprotinin using reductive amination with a reactive FDP moiety and identified 1548 cross-links. Our findings highlight the presence of both specific and nonspecific binding interactions, warranting further investigation to fully understand the biological relevance and implications of cross-links. The chemical platform we have developed proves both feasible and apt for profiling modified proteins in response to acrolein-induced modification, potentially unveiling novel biomarkers and new therapeutic protein targets of human diseases.

## Data Availability

The mass spectrometry proteomics data generated in this study have been deposited to the ProteomeXchange Consortium via the PRIDE partner repository with the data set identifier PXD054410. Protein identification was performed with the human Swisspot database (20 456 entries) [https://www.uniprot.org/uniprotkb?query=*&facets=model_organism%3A9606]. Source data are provided as a Source Data file. Supplementary data are provided with this paper. Source data are provided with this paper.

## Supporting Information Available

Optimization of the reaction with varying α,β-unsaturated aldehydes, the procedure of optimized reactions with peptides to generate FDP-peptide and MP-peptide, late stage functionalization of peptides, protein modification, affinity handle derivatization of proteins, dual modification of proteins, activity assay, cell lysate labeling, capturing and identification of protein binding partners of FDP-modified protein, chemoproteomic profiling and product characterization by NMR, HPLC, LCMS, MS/MS, and HRMS (PDF)
Supplementary Data 1 showing sequence and gene data (XLSX)
Supplementary Data 2 showing proteome and recombinant data (XLSX)
Supplementary Data 3, showing mass-booster data (XLSX)
Supplementary Data 4, showing protein intensity data (XLSX)
Supplementary Data 5, showing protein and gene data (XLSX)
Supplementary Data 6, showing inhibition results (XLSX)
