# Supplementary material for: Acrolein-Mediated Conversion of Lysine to Electrophilic Heterocycles for Protein Diversification and Toxicity Profiling
Source: J Am Chem Soc. 2025 Feb 7;147(7):5679–92. doi: 10.1021/jacs.4c12928 (PMC11848821; doi:10.1021/jacs.4c12928)
Supplement: Supplementary file 1 — ja4c12928_si_001.pdf [file ja4c12928_si_001.pdf]

Acrolein-Mediated Conversion of Lysine to Electrophilic Heterocycles for Protein Diversification and Toxicity Profiling

Zachary E. Paikin[1#], Benjamin Emenike[1#], Rajendra Shirke[1], Christian Michel Beusch[2,3], David Ezra Gordon[2], Monika Raj[1*]

Authors and Affiliations

1. Department of Chemistry, Emory University, Atlanta, GA, United States
   Zachary Paikin, Benjamin Emenike, Rajendra Shirke & Monika Raj
2. Department of Pathology and Laboratory Medicine, Emory University, Atlanta, GA, United States
   Christian Michel Beusch & David Ezra Gordon
3. Department of Surgical Sciences, Uppsala University, Uppsala, Sweden

[#]equal contribution

Table of Contents

Authors and Affiliations 1
Materials and Methods 2
Fig. S1 – Optimization of FDP lysine formation with Ac-GKFV 1a 4
Fig. S2 – Two sites for modification: H_2N-GKFV 8
Fig. S3 – Addition of base to accelerate FDP formation at RT 11
Fig. S4 – Synthesis and characterization: FDP-phenylalanine methyl ester 12
Fig. S5 – Formation of MP lysine with Ac-GKFV 1a and H_2N-GKFV 1b 14
Fig. S6 – Proposed plausible mechanisms for MP formation 18
Fig. S7 – Synthesis of MP on small molecule 19
Fig. S8 – Reaction of 1a with methacrolein 21
Fig. S9 – Reaction of 1a with crotonaldehyde 23
Fig. S10 – Chemoselectivity peptide Ac-KQYWRMES 1c 29
Fig. S11 – Acrolein histidine reactivity: incubation of Ac-GKFV 1a with Ac-GHFV 1d 31
Fig. S12 – Acrolein cysteine reactivity: incubation of Ac-GKFV 1a with Ac-GCFV 1e 32
Fig. S13 – Formation of FDP lysine on bioactive peptides 1f-1i 34
Fig. S14 – Tagging FDP lysine with hydroxylamine 43
Fig. S15 – Formation of MP lysine on bioactive peptides 1f-1i 51
Fig. S16 – Homogeneous labeling of proteins 56

Fig. S17 – Stability studies using FDP myoglobin 98
Fig. S18 – Conversion of FDP to MP on protein 99
Fig. S19 – Bioactivity assay of FDP and MP modified myoglobin in oxidation of o-phenylenediamine with hydrogen peroxide. 105
Fig. S20 – Derivatization of FDP-aprotinin with aminoxy affinity handles. 107
Fig. S21 – Fluorophore labeling of proteins. 109
Fig. S22 – Dual labeling of acrolein treated T47D cell lysate 112
Fig. S23 – Acrolein-mediated activity-based protein profiling. 113
Fig. S24 – MP lysine mass booster data 116
Fig. S25. Identification of protein crosslinking partners of FDP-modified proteins. 123
Fig. S26. Identification of protein binding partners of FDP-modified proteins from cell lysate. 131
Fig. S27. Structural evaluation of FDP-aprotinin and unmodified aprotinin. 134
Fig. S28. Downstream analysis of FDP-aprotinin binders. 134
References 137

Materials and Methods

General.

All commercial materials (Sigma-Aldrich, Oakwood, and Novabiochem) were used without further purification. All solvents were reagent or HPLC (Fisher) grade. All reactions were performed under air in glass vials. Yields refer to chromatographically pure compounds; percent conversions were obtained by comparing HPLC peak areas of products and starting materials. Analytical thin layer chromatography was carried out using silica gel 60 F254 plates (0.2 mm, Merck), that were visualized under UV light (at 220 nm). TLC, HPLC and MS were used to monitor reaction progress, and product elucidation was done using MS, IR, and NMR.

Materials.

Fmoc-amino acids, Rink amide resin, and hexafluorophosphate benzotriazole tetramethyl uronium (HBTU) were obtained from CreoSalus (Louisville, Kentucky). Wang resin was obtained from Sigma Aldrich (St. Louis, Missouri). N,N′-diisopropylethylamine (DIPEA) was obtained from TCI (Portland, Oregon). Piperidine and trifluoroacetic acid (TFA) were obtained from Alfa Aesar (Ward Hill, Massachusetts). N,N-dimethylformamide (DMF), dichloromethane (DCM), methanol (MeOH), ethyl acetate (EtOAc), hexanes, and acetonitrile (ACN) were obtained from VWR (100 Matsonford Road Radnor, Pennsylvania). All other small molecules were obtained from Sigma. Commercially available proteins: myoglobin, lysozyme from chicken egg white, lysozyme from

human, aprotinin, ubiquitin, bovine serum albumin (BSA), apo-transferrin, chymotrypsin, and ribonuclease-A were obtained from Sigma. Acrolein was obtained from Sigma. Alexafluor-647 dye was obtained from Thermo Fisher Scientific.

Purification.

Purification of peptide starting materials was performed using high performance liquid chromatography (HPLC) on an Agilent 1100 series HPLC equipped with a C-18 reverse phase column with a particle size of 5 µm or PREP HPLC. Teledyne ISCO ACCQ Prep HP150 equipped with a C-18 reverse phase 9.4x250 mm column with a particle size of 5 µm. All separations involved a mobile phase of 0.1 % formic acid in water (solvent A) and 0.1 % formic acid in acetonitrile (solvent B). The HPLC method used a linear gradient of 0-80% solvent B over 30 min at RT with a flow rate of 1 mL min-1. The eluent was monitored by absorbance at 220 nm.

Instrumentation and sample analysis.

NMR. ^{1}H and ^{13}C spectra were acquired at 25 °C in $CDCl_3$ using an Agilent DD2 (400 MHz) spectrometer with a 3-mm He triple resonance (HCN) cryoprobe. All 1H NMR chemical shifts (δ) were referenced relative to the residual DMSO-d_6 peak at 2.50 ppm or CDCl3 peak at 7.26 ppm. ^{13}C NMR chemical shifts were referenced to $CDCl_3$ at 77.16 ppm. ^{13}C NMR spectra were proton decoupled. NMR spectral data are reported as chemical shift (multiplicity, coupling constants (J), integration). The following abbreviations (or combinations thereof) were used to explain multiplicities: singlet (s), doublet (d), triplet (t), quartet (q), multiplet (m), broad (b). Coupling constants (J) are reported in hertz (Hz).

Analytical HPLC. Analytical HPLC chromatography (HPLC) was performed on an Agilent 1200 series HPLC equipped with a 5 µm pore size C-18 reversed-phase column. All separations involved mobile phase of 0.1 % formic acid in water (solvent A) and 0.1 % formic acid in acetonitrile (solvent B) run in linear gradients with a constant flow rate of 1 mL min-1. The eluent was monitored with a detection wavelength of 220 nm. HPLC METHOD A: Gradient: 0-80 % B over 30 min. HPLC METHOD B: Gradient: 0-40 % B over 30 min. HPLC METHOD C: Gradient: 0-20 % B over 30 min.

HRMS. High resolution MS data were acquired on Thermo Exactive Plus using a heated electrospray source. The solution was infused at a rate of 10-25 µL min-1 electrospray using 3.3 kV. The typical settings were Capillary temp 320 °C. S-lens RF level was between 30-80 with an AGC setting of 1 E6. The maximum injection time was set to 50 ms. Spectra were taken at 140,000 resolutions at m/z 200 using Tune software and analyzed with Thermo's Freestyle software.

Fmoc Solid-Phase Peptide Synthesis (Fmoc-SPPS).[1] Peptides were synthesized using standard protocols. Peptides were synthesized manually on a 0.25 mmol scale using Rink amide resin or Wang resin. Resin was swollen with DCM for 1 hour at RT. Fmoc was deprotected using 20 % piperidine–DMF for 15 min to obtain a deprotected resin. Fmoc protected amino acid (1.25 mmol, 5 equiv.) was coupled using HBTU (1.25 mmol, 5 equiv.) and DIPEA (1.25 mmol, 5 equiv.) in DMF for 20 min at RT. Fmoc deprotection was achieved using 20% piperidine in DMF for 15 min at RT. N-terminal acetylation was carried out with 30% acetic anhydride in DMF for 1 hour at RT.

Peptides were cleaved from the resin using 4 mL of a cocktail consisting of 95:2.5:2.5 trifluoroacetic acid : water : triethylsilane (TES) for 2 hours. The resin was removed by filtration and the resulting solution was concentrated. Peptides were precipitated and centrifugated with cold diethyl ether (3 x 10 mL) to obtain the crude product. Crude peptides were dissolved in ACN:H2O and purified by preparatory HPLC.

General cell culture techniques. Cells were maintained at 37 °C and 5% CO2. T-47D cells were cultured in RPMI supplemented with 10% (V/V) fetal bovine serum (FBS), 1% (V/V) penicillin/streptomycin (100 µg/mL), and amphotericin-b (1 mL).

Fig. S1 – Optimization of FDP lysine formation with Ac-GKFV 1a

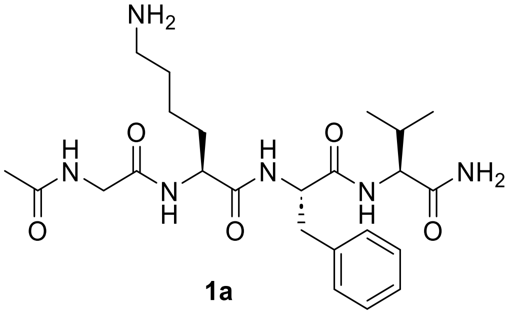

1a

Ac-GKFV-CONH₂ (1a) peptide. LCMS, m/z 491.2883 (calcd. [M+H$^+$] = 491.2976), Purity: >99% (HPLC analysis at 220 nm). Retention time in HPLC: 6.9 min.

HPLC Trace of Ac-GKFV 1a

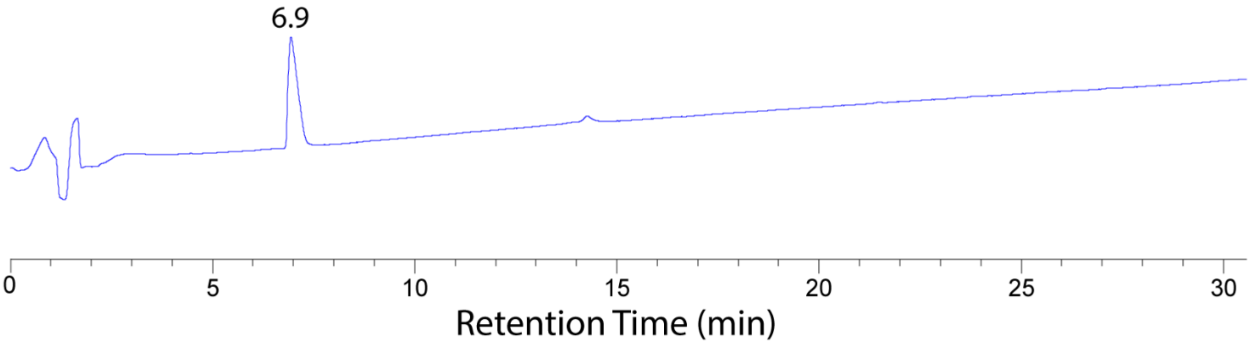

HRMS of Ac-GKFV 1a

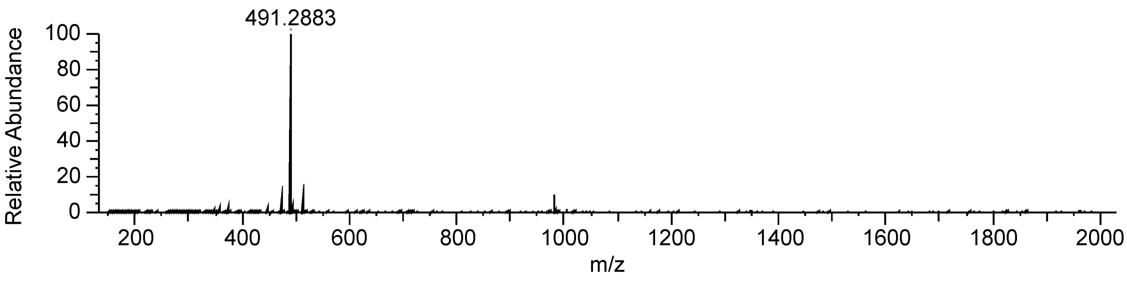

Formation of FDP Product 2a

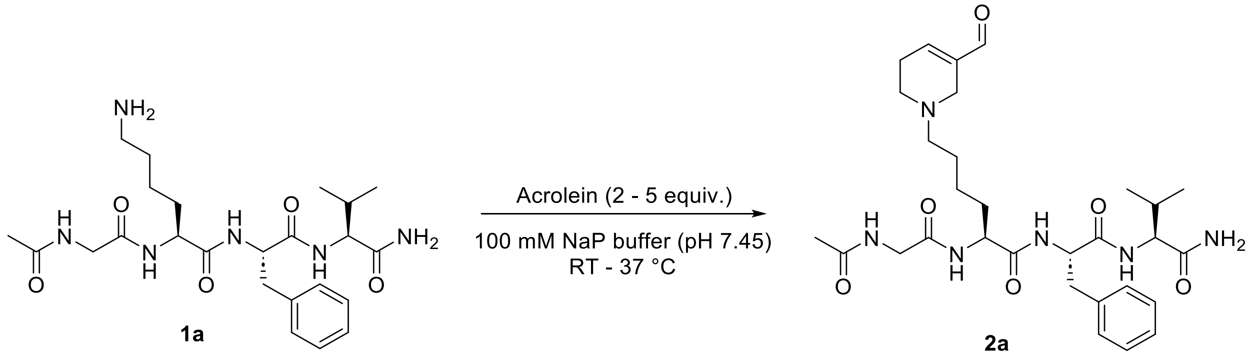

Ac-GKFV **1a** (2 mg, 4.1 µmol, 1 equiv.) was dissolved in 580 µL of 100 mM NaP buffer (pH 7.45) in a 1” dram vial. The designated amount of acrolein was added to the vial from a freshly prepared stock solution (20 µL). The mixture was stirred at the designated temperature for the time listed below, after which the reaction was analyzed using **HPLC Method A**. Conversion to FDP product **2a** for each of the attempted conditions is listed in the table below.

Optimization Table

Entry	Equivalents of Acrolein	Temperature	Time	Conversion to 2a
1	3 equiv.	RT	24 hrs	44% (56% Double Michael Addition Intermediate **2a’**)
2	2 equiv.	37 °C	5 hrs	88% (12% Starting Peptide **1a**)
3	3 equiv.	37 °C	2 hrs	83% (17% Double Michael Addition Intermediate **2a’**)
4	3 equiv.	37 °C	5 hrs	>98%
5	5 equiv.	37 °C	5 hrs	>98%

Structure of Observed Double Michael Addition Intermediate 2a’

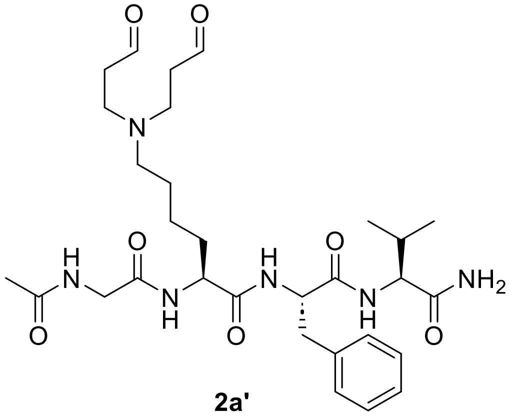

2a'

HPLC Trace for Entry 1: Optimization of Formation of FDP Lysine Peptide 2a

Double Michael Addition Intermediate 2a'. LCMS, m/z 603.3489 (calcd. $[M+H^+]$ = 604.3501), Purity: >99% (HPLC analysis at 220 nm). Retention time in HPLC: 8.0 min.

FDP Peptide 2a. LCMS, m/z 585.3393 (calcd. $[M+H^+]$ = 585.3395), Purity: >99% (HPLC analysis at 220 nm). Retention time in HPLC: 9.2 min.

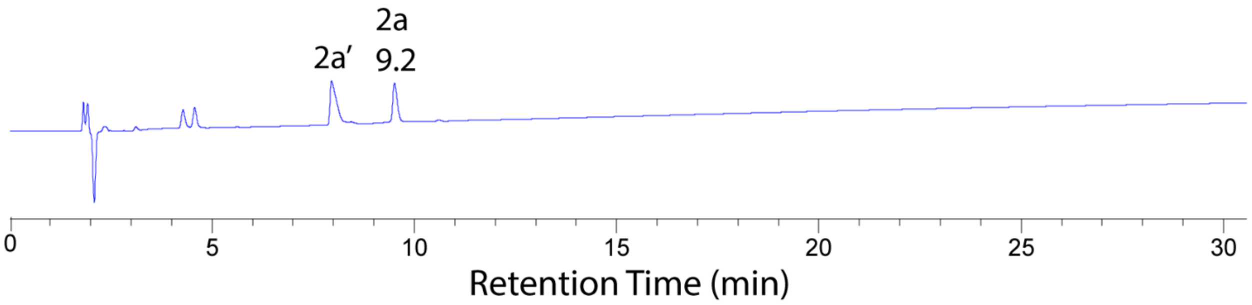

HPLC Trace for Entry 2: Optimization of Formation of FDP Lysine Peptide 2a

Ac-GKFV-CONH$_2$ (1a) peptide. LCMS, m/z 491.2883 (calcd. $[M+H^+]$ = 491.2976), Purity: >99% (HPLC analysis at 220 nm). Retention time in HPLC: 7.4 min.

FDP Peptide 2a. LCMS, m/z 585.3393 (calcd. $[M+H^+]$ = 585.3395), Purity: >99% (HPLC analysis at 220 nm). Retention time in HPLC: 8.8 min.

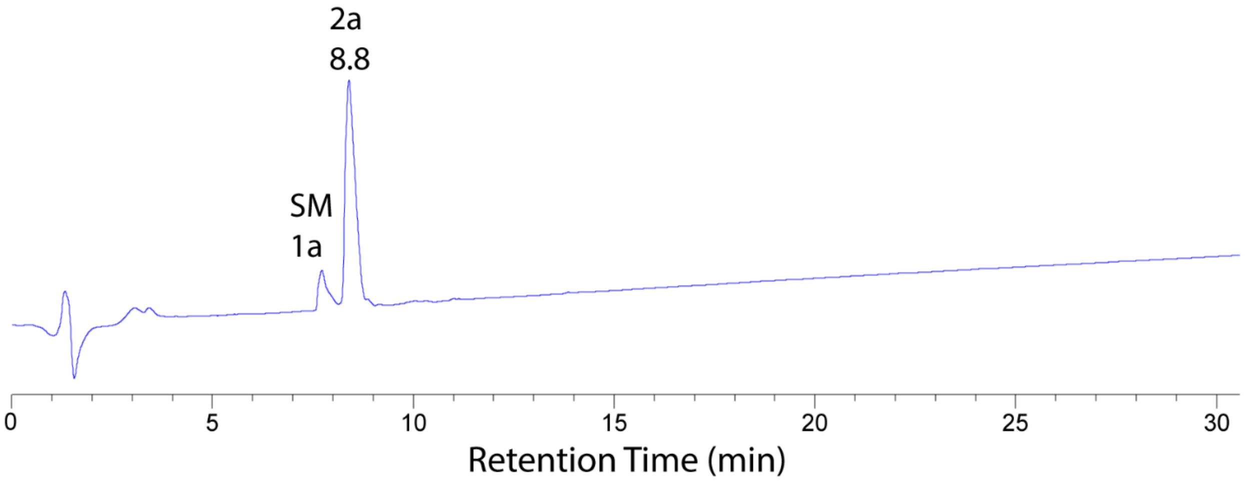

HPLC Trace for Entry 3: Optimization of Formation of FDP Lysine Peptide 2a

Double Michael Addition Intermediate 2a'. LCMS, m/z 603.3489 (calcd. $[M+H^+]$ = 604.3501), Purity: >99% (HPLC analysis at 220 nm). Retention time in HPLC: 7.2 min.

FDP Peptide 2a. LCMS, m/z 585.3393 (calcd. $[M+H^+]$ = 585.3395), Purity: >99% (HPLC analysis at 220 nm). Retention time in HPLC: 8.1 min.

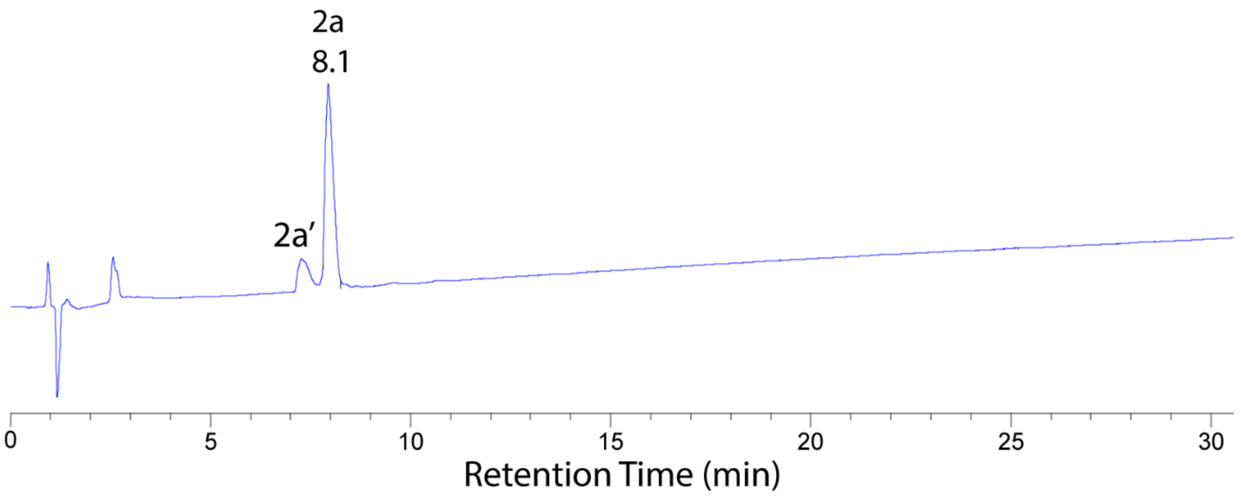

HPLC Trace for Entry 4: Optimization of Formation of FDP Lysine Peptide 2a

FDP Peptide 2a. LCMS, m/z 585.3393 (calcd. [M+H$^+$] = 585.3395), Purity: >99% (HPLC analysis at 220 nm). Retention time in HPLC: 9.2 min.

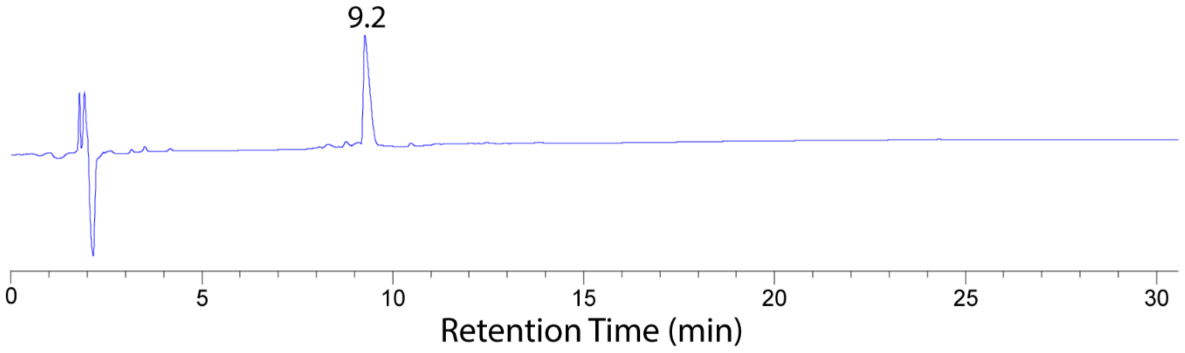

HPLC Trace for Entry 5: Optimization of Formation of FDP Lysine Peptide 2a

FDP Peptide 2a. LCMS, m/z 585.3393 (calcd. [M+H$^+$] = 585.3395), Purity: >99% (HPLC analysis at 220 nm). Retention time in HPLC: 7.9 min.

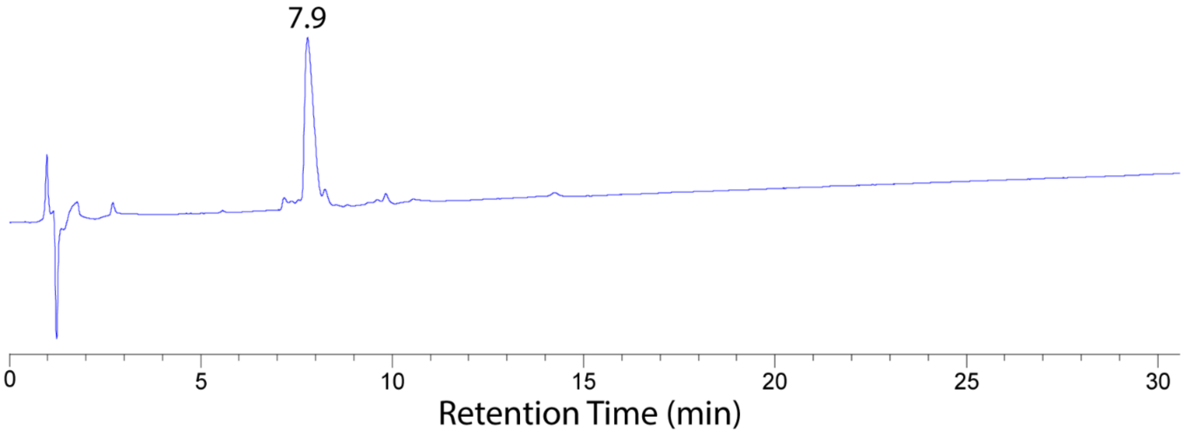

HRMS of FDP Lysine Peptide 2a

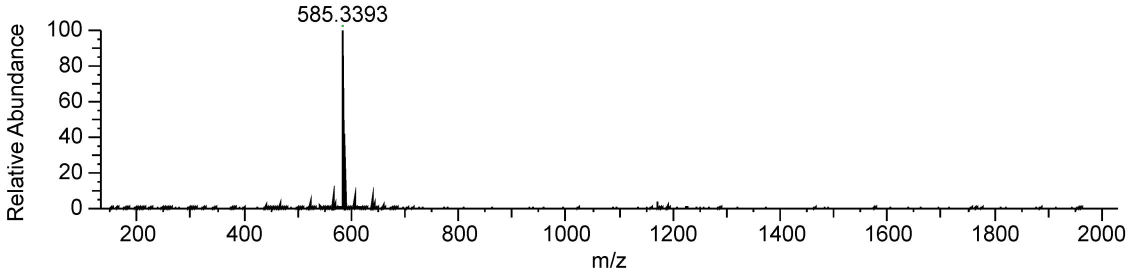

HRMS of Double Michael Addition Intermediate 2a'

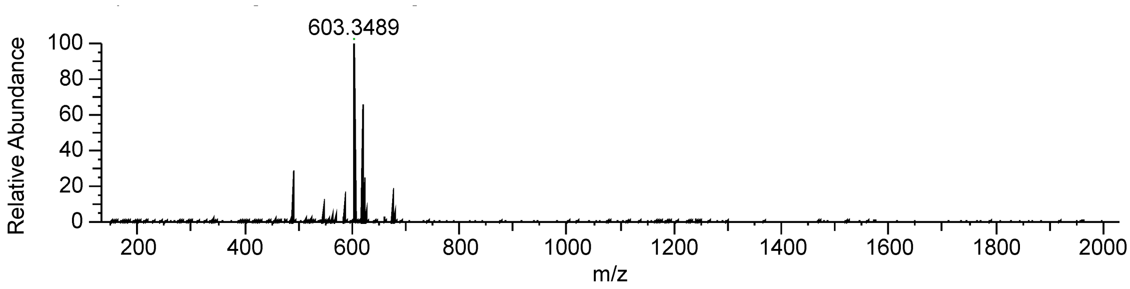

Fig. S2 – Two sites for modification: H_2N-GKFV

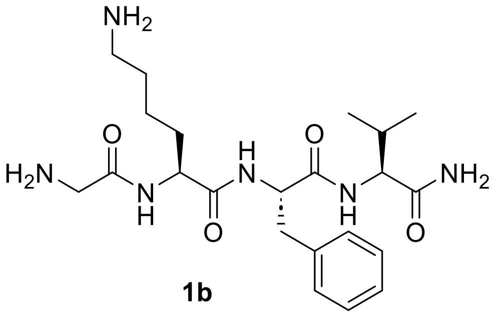

1b

H_2N-GKFV-$CONH_2$ (1b) peptide. LCMS, m/z 449.2869 (calcd. [$M+H^+$] = 449.2871), Purity: >99% (HPLC analysis at 220 nm). Retention time in HPLC: 4.0 min.

HPLC Trace of H_2N-GKFV 1b

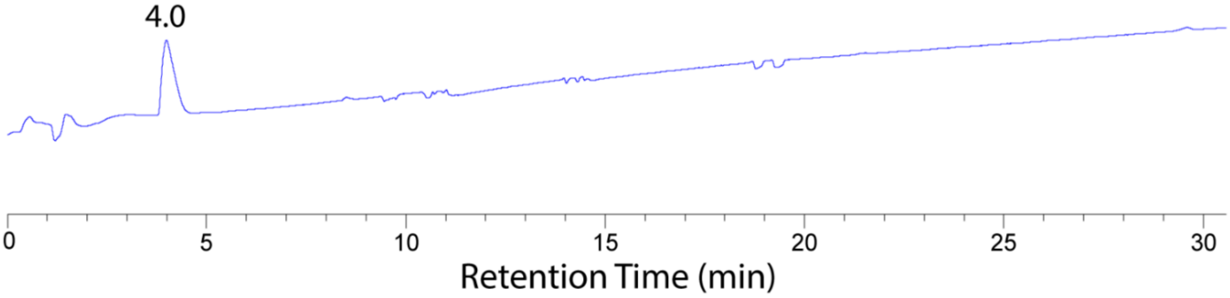

HRMS of H_2N-GKFV 1b

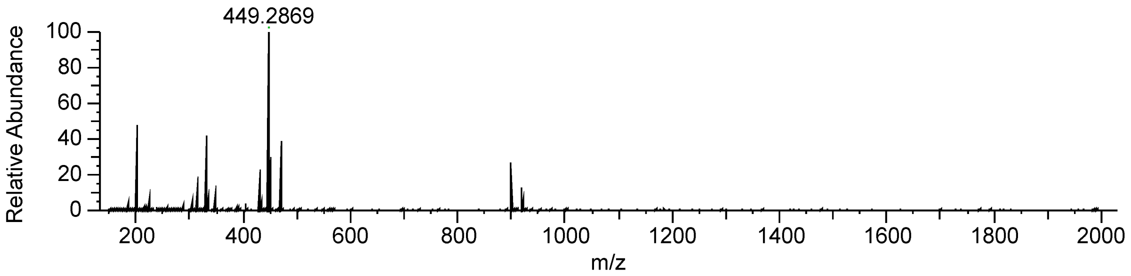

Full Conversion to Dual FDP Product 2b with 5 Equivalents of Acrolein

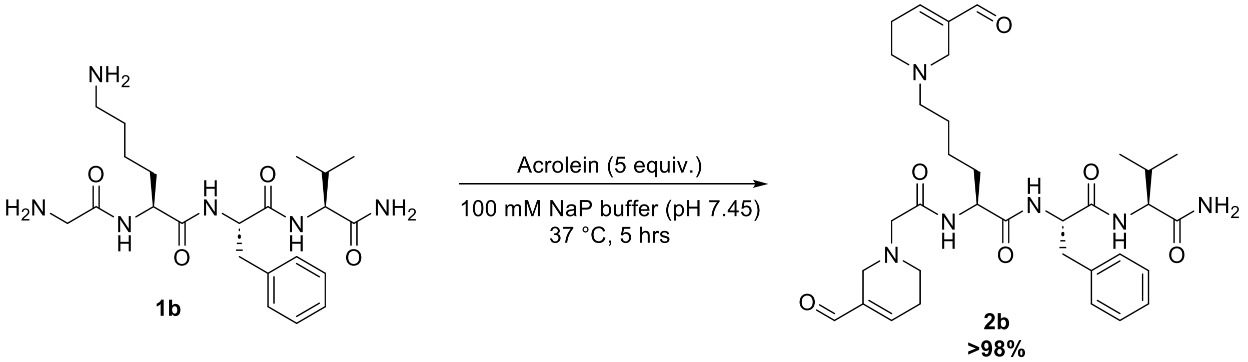

H_2N-GKFV **1b** (2 mg, 4.5 µmol, 1 equiv.) was dissolved in 580 µL of 100 mM NaP buffer (pH 7.45) in a 1" dram vial. Acrolein (1.49 µL, 22.3 µmol, 5 equiv.) was added to the vial from a freshly prepared stock solution (20 µL). The mixture was stirred at 37 °C for 5 hours, after which the reaction was analyzed using **HPLC Method A**, revealing >98% conversion to Dual FDP Product **2b.**

Dual FDP Product 2b. LCMS, m/z 637.3708 (calcd. $[M+H^+]$ = 637.3708), Purity: >99% (HPLC analysis at 220 nm). Retention time in HPLC: 6.2 min.

HPLC Trace for Formation of Dual FDP Product 2b

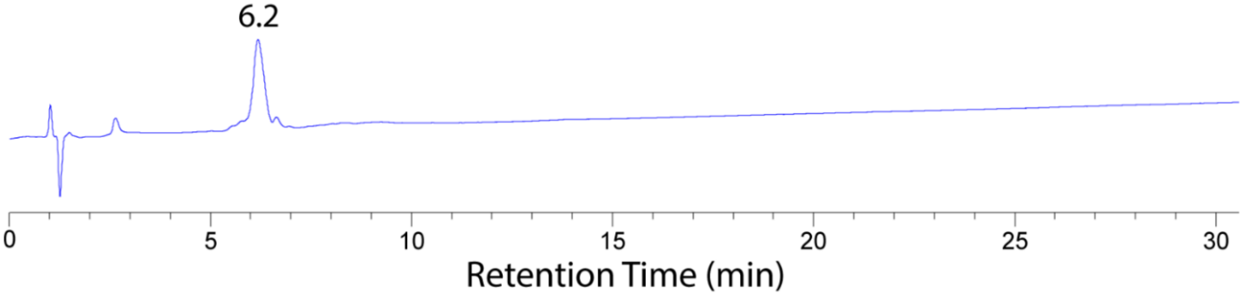

HRMS of Dual FDP Product 2b

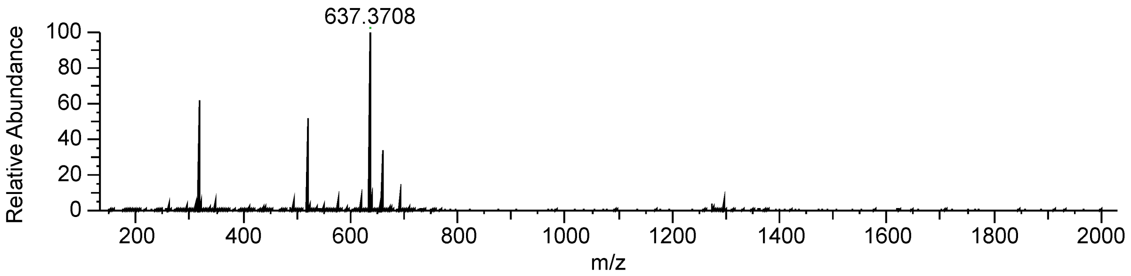

Formation of Single FDP Product Using 1.5 Equivalents of Acrolein

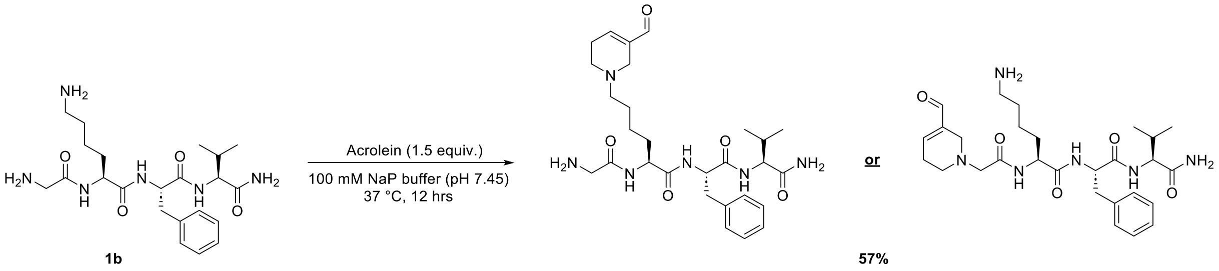

H_2N-GKFV **1b** (2 mg, 4.5 µmol, 1 equiv.) was dissolved in 580 µL of 100 mM NaP buffer (pH 7.45) in a 1" dram vial. Acrolein (0.45 µL, 6.7 µmol, 1.5 equiv.) was added to the vial from a freshly prepared stock solution (20 µL). The mixture was stirred at 37 °C for 12 hours, after which the reaction was analyzed using **HPLC Method A**, revealing 57% conversion to a product containing a single FDP modification and 43% remaining starting peptide **1b**.

H_2N-GKFV-$CONH_2$ (1b) peptide. LCMS, m/z 449.2869 (calcd. [$M+H^+$] = 449.2871), Purity: >99% (HPLC analysis at 220 nm). Retention time in HPLC: 4.0 min.

Single FDP Product. LCMS, m/z 543.3126 (calcd. [$M+H^+$] = 543.3289), m/z 565.2935 (calcd. [$M+Na^+$] = 565.3109), m/z 272.1599 (calcd. [($M+2H^+$)/2] = 272.1681), Purity: >99% (HPLC analysis at 220 nm). Retention time in HPLC: 5.5 min.

HPLC Trace for Formation of Single FDP Product

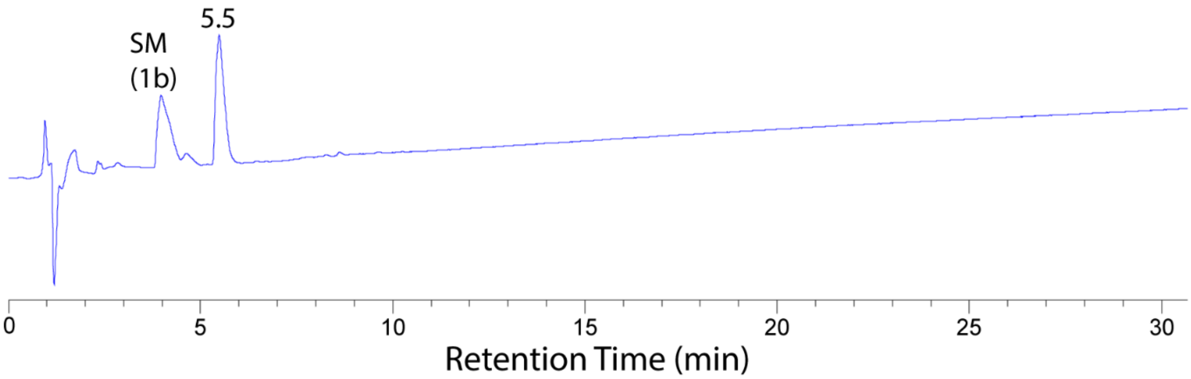

HRMS of Single FDP Product

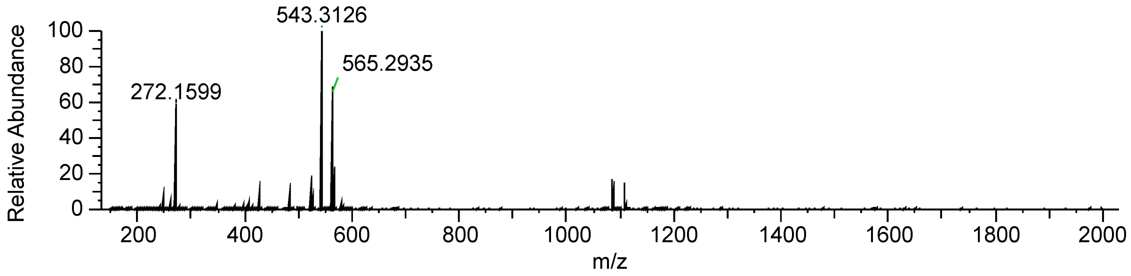

Fig. S3 – Addition of base to accelerate FDP formation at RT

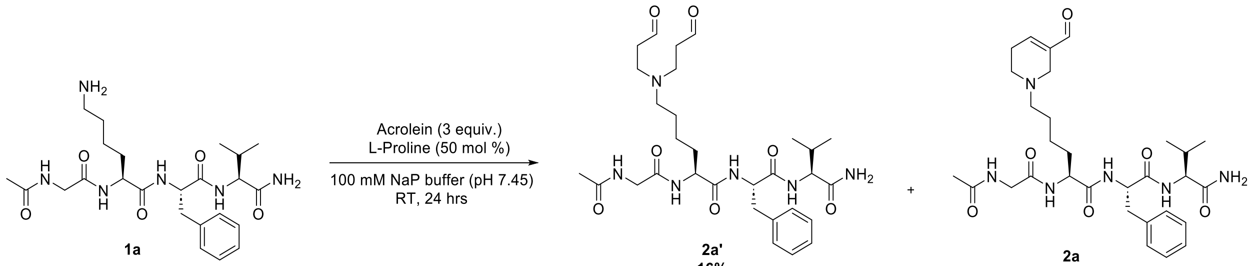

Ac-GKFV **1a** (2 mg, 4.1 µmol, 1 equiv.) was dissolved in 550 µL of 100 mM NaP buffer (pH 7.45) in a 1" dram vial. Acrolein (0.82 µL, 12.2 µmol, 3 equiv.) was added to the vial from a freshly prepared stock solution (20 µL). Next, L-proline (0.23 mg, 2.0 µmol, 50 mol %) was transferred to the reaction, again from a stock solution (30 µL). The mixture was stirred at room temperature for 24 hours, after which the reaction was analyzed using **HPLC Method A**, revealing 84% conversion to FDP peptide **2a**, with 16% remaining as double Michael addition intermediate **2a'**, an improvement in conversion compared to FDP formation at room temperature in the absence of catalytic base.

Double Michael Addition Intermediate 2a'. LCMS, m/z 603.3489 (calcd. $[M+H^+]$ = 604.3501), Purity: >99% (HPLC analysis at 220 nm). Retention time in HPLC: 7.6 min.

FDP Peptide 2a. LCMS, m/z 585.3393 (calcd. $[M+H^+]$ = 585.3395), Purity: >99% (HPLC analysis at 220 nm). Retention time in HPLC: 8.9 min.

HPLC Trace for FDP Formation at RT with Addition of Base

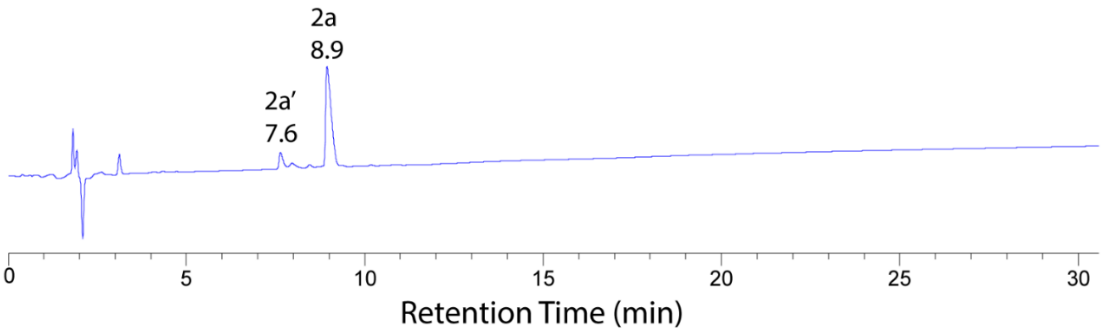

Fig. S4 – Synthesis and characterization: FDP-phenylalanine methyl ester

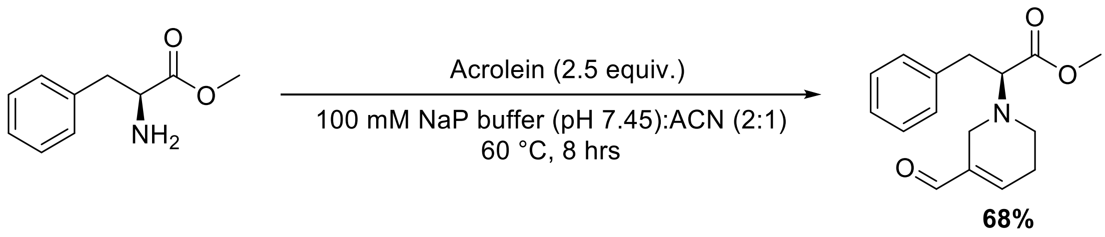

L-Phenylalanine methyl ester hydrochloride (100 mg, 0.46 mmol, 1 equiv.) was added to a 35 mL high-pressure tube and dissolved in 2:1 100 mM NaP buffer (pH 7.45):ACN (4 mL:2 mL). Acrolein (77 µL, 1.16 mmol, 2.5 equiv.) was added to the reaction mixture. The high-pressure tube was sealed, and the reaction was stirred at 60°C for 8 hours. Upon completion by TLC, the reaction mixture was transferred to a separatory funnel, diluted with EtOAc (15 mL) and washed with NaCl brine (3 x 15 mL). The organic layer was dried over Na_2SO_4 and then adsorbed onto silica. Purification by column chromatography (1:25 MeOH:DCM eluent) yielded the FDP product as a white-yellow solid (85 mg, 68% yield). Analytical TLC, 1:19 MeOH:DCM eluent, $R_f = 0.45$.

^{1}H NMR (400 MHz, $CDCl_3$): δ = 9.41 (s, 1H), 7.30 – 7.24 (m, 2H), 7.23 – 7.17 (m, 3H), 6.84 (tt, *J* = 3.9, 1.8 Hz, 1H), 3.60 (s, 3H), 3.44 – 3.41 (m, 2H), 3.19 – 2.96 (m, 2H), 2.96 – 2.68 (m, 2H), 2.50 – 2.44 (m, 1H) ppm. **^{13}C NMR** (101 MHz, $CDCl_3$): δ = 192.34, 171.92, 148.55, 140.45, 137.96, 129.24, 128.55, 126.67, 69.03, 51.37, 46.36, 45.80, 35.77, 27.96 ppm. **HRMS**: calcd. for $C_{16}H_{19}NO_3$ [M+H$^+$] 274.1438; found 274.1439.

^{1}H NMR of FDP-Phenylalanine Methyl Ester

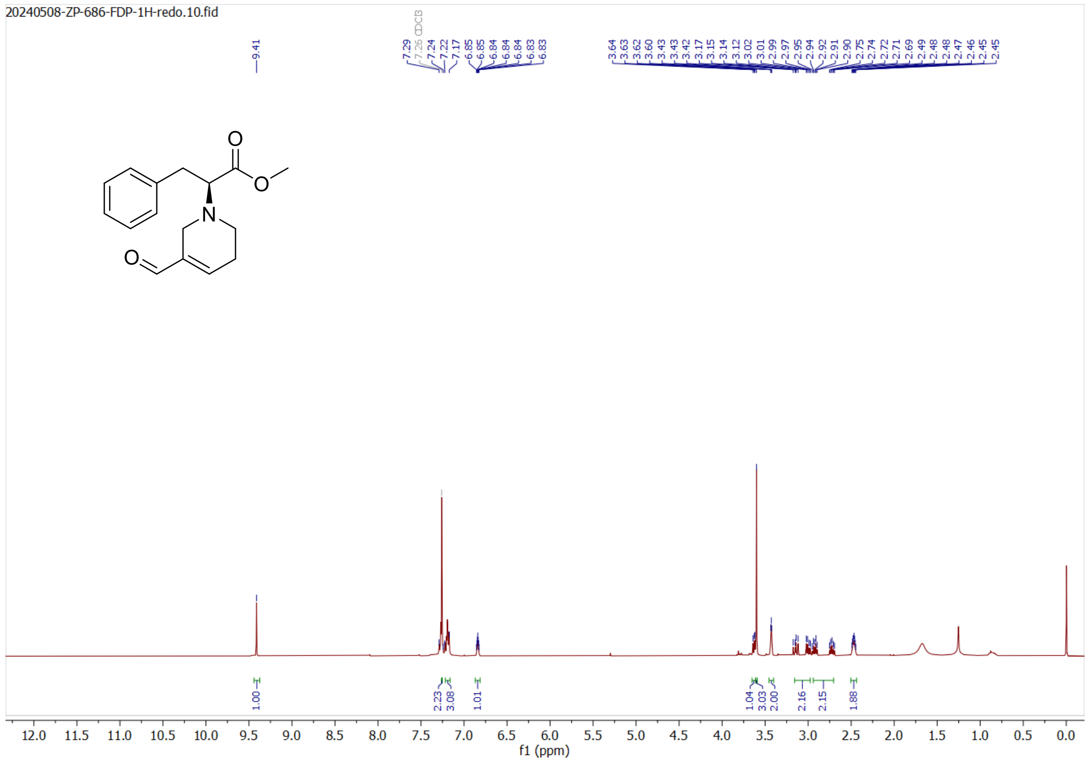

^{13}C NMR of FDP-Phenylalanine Methyl Ester

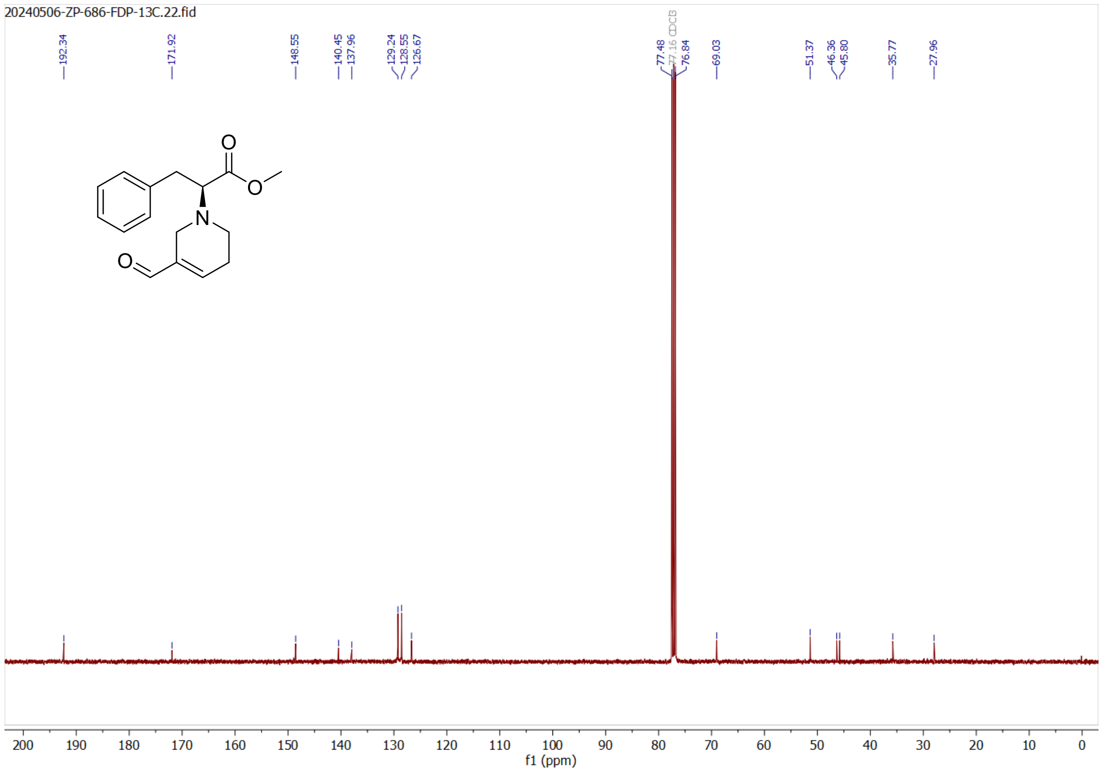

HRMS of FDP-Phenylalanine Methyl Ester

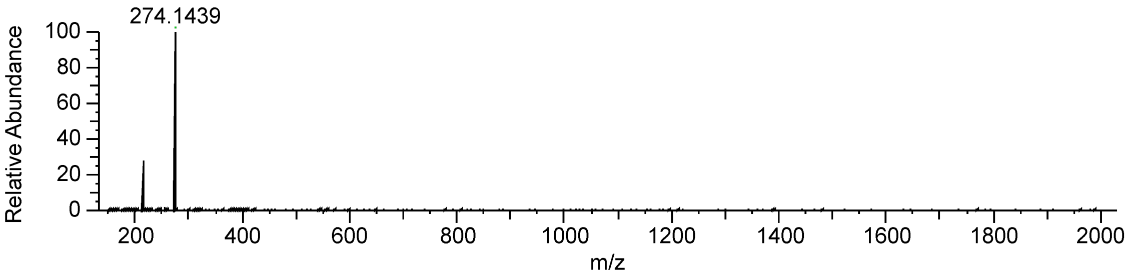

Fig. S5 – Formation of MP lysine with Ac-GKFV 1a and H_2N-GKFV 1b

Formation of MP Lysine Peptide 3a at 60°C

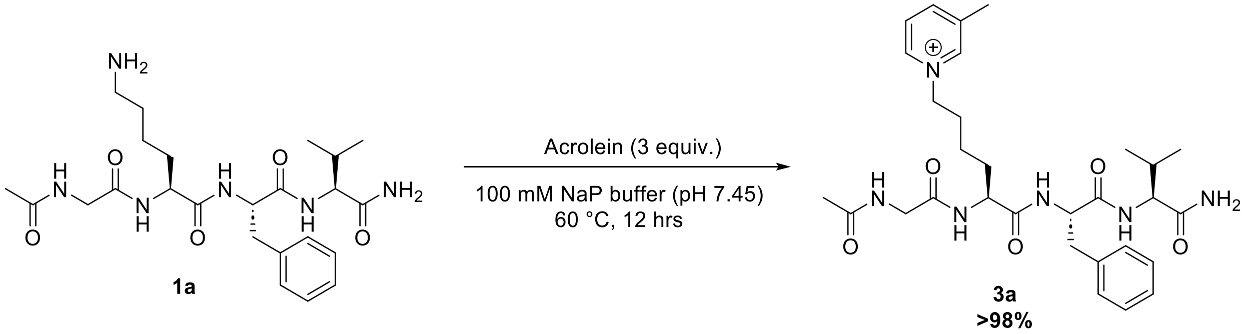

Ac-GKFV **1a** (2 mg, 4.1 µmol, 1 equiv.) was dissolved in 580 µL of 100 mM NaP buffer (pH 7.45) in a 1” dram vial. Acrolein (0.82 µL, 12.2 µmol, 3 equiv.) was added to the vial from a freshly prepared stock solution (20 µL). The mixture was stirred at 60 °C for 12 hours, after which the reaction was analyzed using **HPLC Method A**, revealing >98% conversion to MP-modified product **3a**.

MP Peptide 3a. LCMS, m/z 567.3285 (calcd. [M] = 567.3289), Purity: >99% (HPLC analysis at 220 nm). Retention time in HPLC: 7.6 min.

HPLC Trace for Formation of MP Lysine Peptide 3a at 60°C

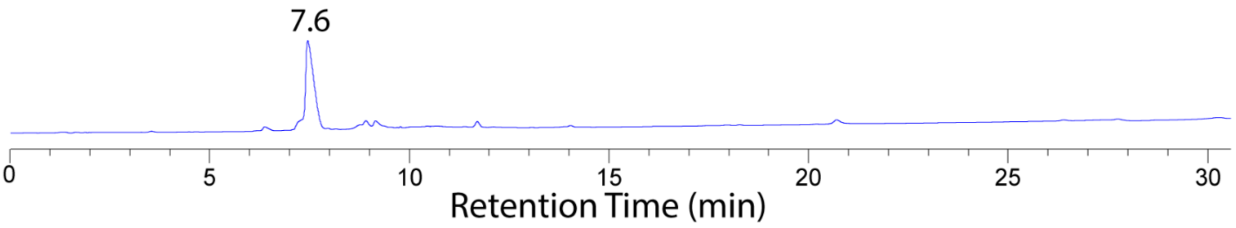

HRMS of MP Lysine Peptide 3a

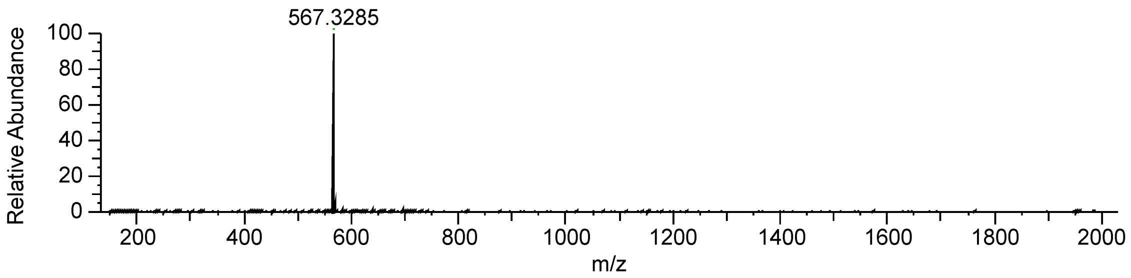

Formation of MP Lysine Peptide 3a at 80°C

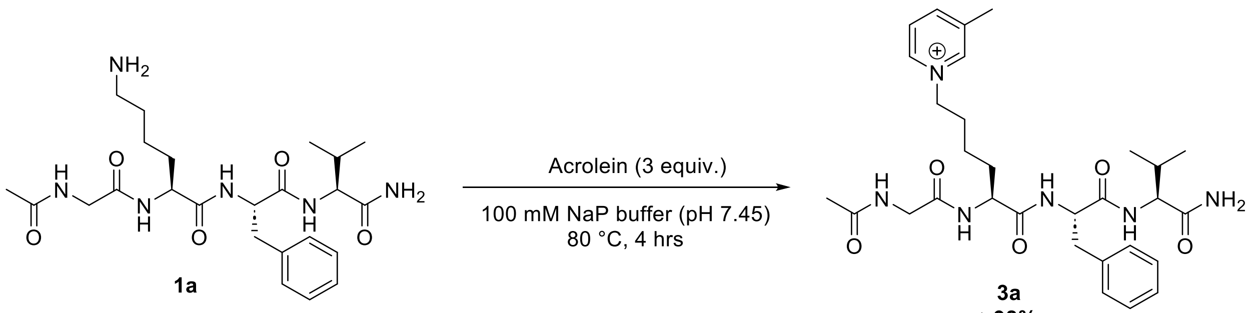

Ac-GKFV **1a** (2 mg, 4.1 µmol, 1 equiv.) was dissolved in 580 µL of 100 mM NaP buffer (pH 7.45) in a 1" dram vial. Acrolein (0.82 µL, 12.2 µmol, 3 equiv.) was added to the vial from a freshly prepared stock solution (20 µL). The mixture was stirred at 80 °C for 4 hours, after which the reaction was analyzed using **HPLC Method A**, revealing >98% conversion to MP-modified product **3a**.

MP Peptide 3a. LCMS, m/z 567.3285 (calcd. [M] = 567.3289), Purity: >99% (HPLC analysis at 220 nm). Retention time in HPLC: 8.0 min.

HPLC Trace for Formation of MP Lysine Peptide 3a at 80°C

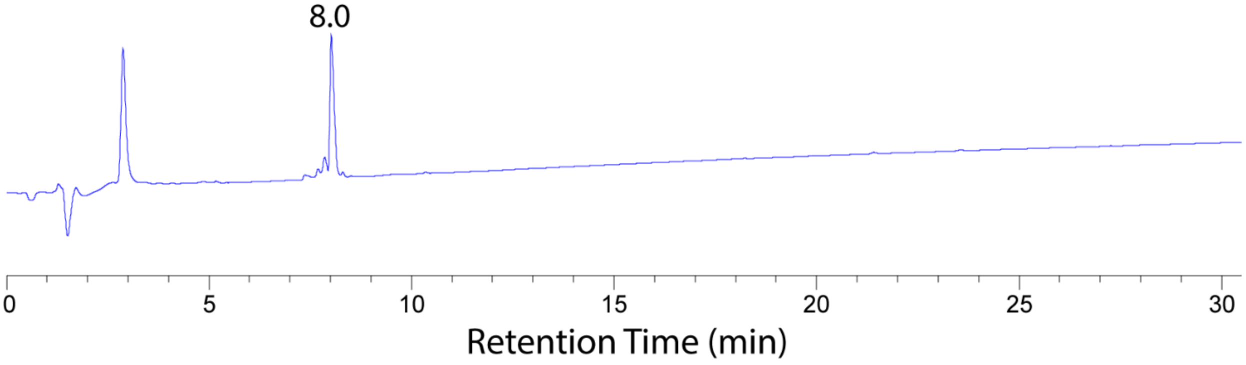

Unsuccessful Attempted MP Lysine Formation in Base Under Mild Conditions

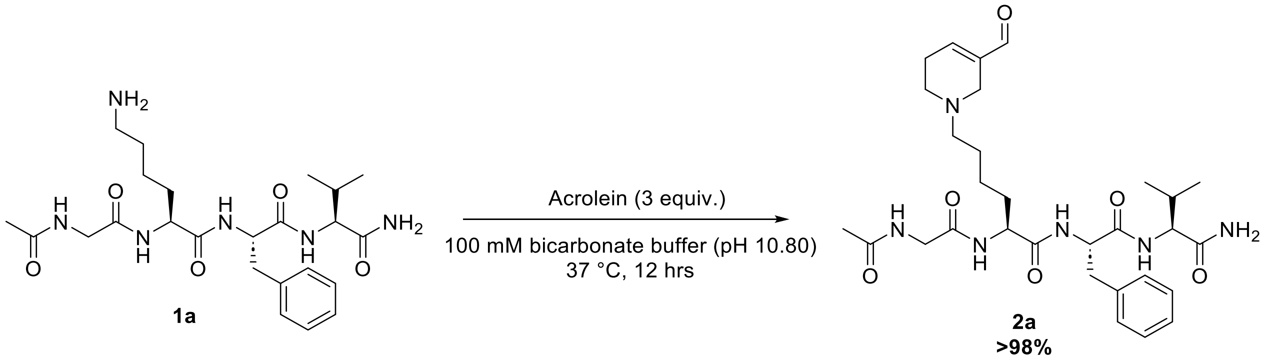

Ac-GKFV **1a** (2 mg, 4.1 μmol, 1 equiv.) was dissolved in 580 μL of 100 mM bicarbonate buffer (pH 10.80) in a 1” dram vial. Acrolein (0.82 μL, 12.2 μmol, 3 equiv.) was added to the vial from a freshly prepared stock solution (20 μL). The mixture was stirred at 37 °C for 12 hours, after which the reaction was analyzed using **HPLC Method A**, revealing >98% conversion to FDP-modified product **2a**, with negligible formation of the MP product **3a**.

FDP Peptide 2a. LCMS, m/z 585.3393 (calcd. [M+H$^+$] = 585.3395), Purity: >99% (HPLC analysis at 220 nm). Retention time in HPLC: 9.4 min.

HPLC Trace for Attempted MP Lysine Formation in Base Under Mild Conditions

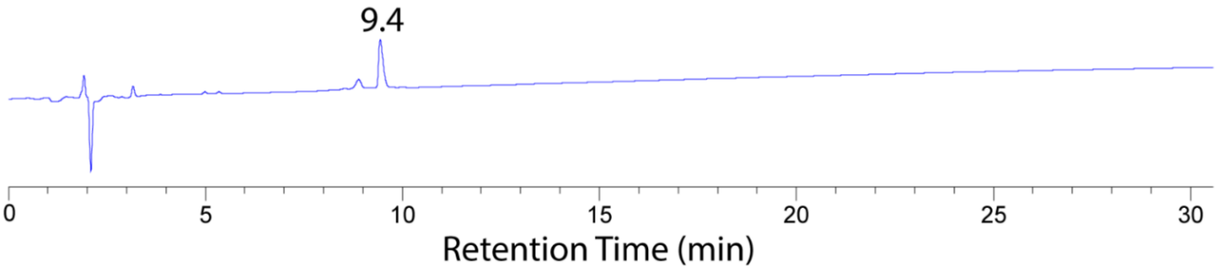

MP Lysine Formation from Heating Pure FDP Lysine in Buffer – 60 °C

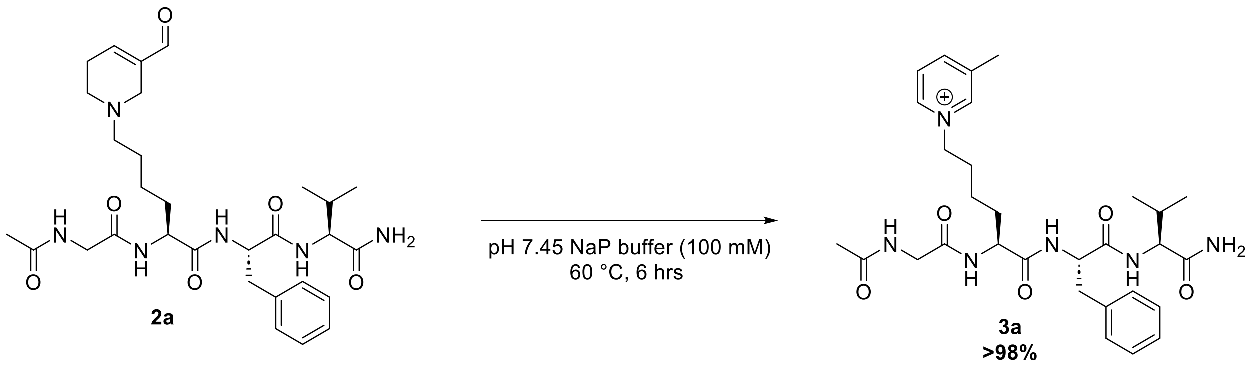

FDP Peptide **2a** (1 mg, 2.0 μmol), purified via analytical HPLC, was dissolved in 400 μL of 100 mM NaP buffer (pH 7.45) in a 1” dram vial. The peptide was heated at 60 °C for 6 hours, after

which the reaction was analyzed using **HPLC Method A**, revealing >98% conversion to MP-modified product **3a** solely from heating the pure FDP peptide.

MP Peptide 3a. LCMS, m/z 567.3285 (calcd. [M] = 567.3289), Purity: >99% (HPLC analysis at 220 nm). Retention time in HPLC: 8.0 min.

HPLC Trace for MP Lysine Formation from Heating Pure FDP Lysine in Buffer – 60 °C

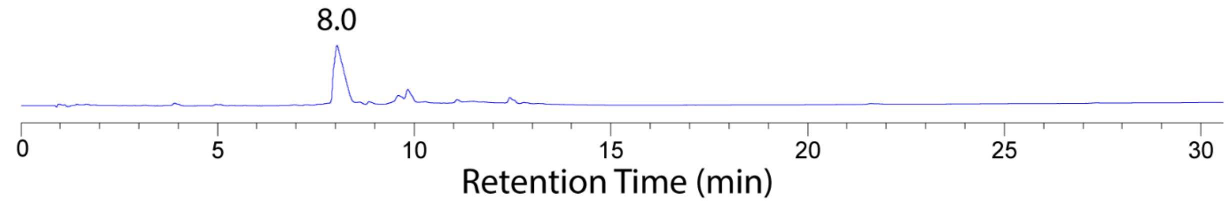

MP Lysine Formation from Heating Pure FDP Lysine in Buffer – 80 °C

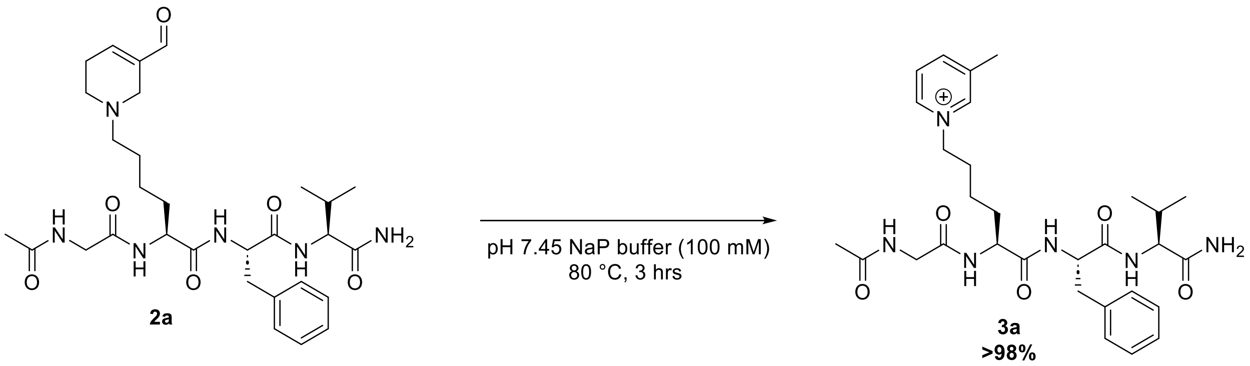

FDP Peptide **2a** (1 mg, 2.0 µmol), purified via analytical HPLC, was dissolved in 400 µL of 100 mM NaP buffer (pH 7.45) in a 1” dram vial. The peptide was heated at 80 °C for 3 hours, after which the reaction was analyzed using **HPLC Method A**, revealing >98% conversion to MP-modified product **3a** solely from heating the pure FDP peptide.

MP Peptide 3a. LCMS, m/z 567.3285 (calcd. [M] = 567.3289), Purity: >99% (HPLC analysis at 220 nm). Retention time in HPLC: 8.0 min.

HPLC Trace for MP Lysine Formation from Heating Pure FDP Lysine in Buffer – 80 °C

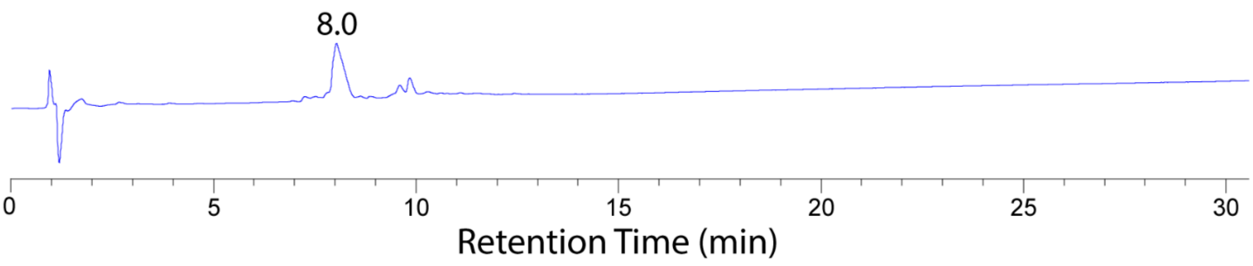

Fig. S6 – Proposed plausible mechanisms for MP formation

Previous Mechanism in Literature[2]

The previous mechanism reported in literature for MP formation involves Schiff base formation, followed by a Michael addition and intramolecular cyclization.

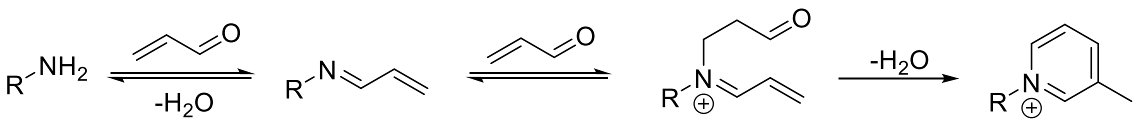

New Proposed FDP to MP Mechanism

We discovered that MP formation proceeds through FDP as an intermediate and propose the following mechanism for the conversion of FDP to MP. Enol formation is followed by rearrangement and aromatization to produce MP in this reagentless transformation.

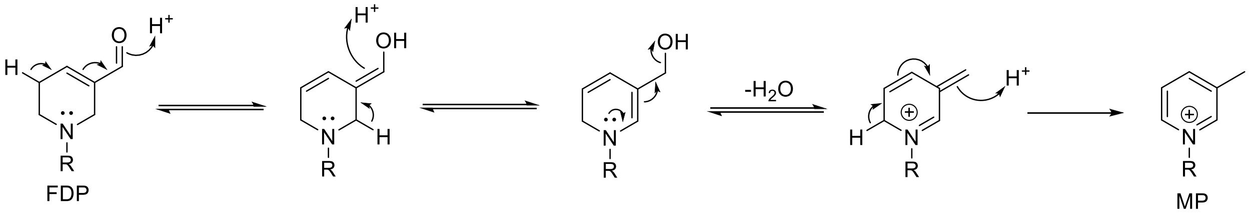

MP Lysine Formation Using Peptide 1b: No N-Terminal MP

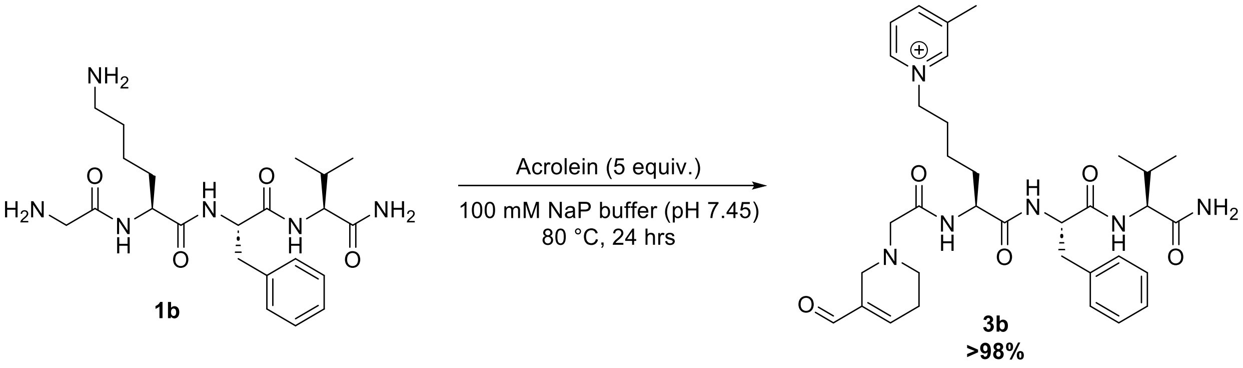

H_2N-GKFV **1b** (2 mg, 4.5 µmol, 1 equiv.) was dissolved in 580 µL of 100 mM NaP buffer (pH 7.45) in a 1” dram vial. Acrolein (1.49 µL, 22.3 µmol, 5 equiv.) was added to the vial from a freshly prepared stock solution (20 µL). The mixture was stirred at 80 °C for 24 hours, after which the reaction was analyzed using **HPLC Method A**, revealing >98% conversion to peptide product **3b** containing an MP lysine side chain and FDP N-terminus.

Peptide Product 3b. LCMS, m/z 619.3602 (calcd. [M] = 619.3602), Purity: >99% (HPLC analysis at 220 nm). Retention time in HPLC: 5.9 min.

HPLC Trace for MP Lysine Formation Using Peptide 1b

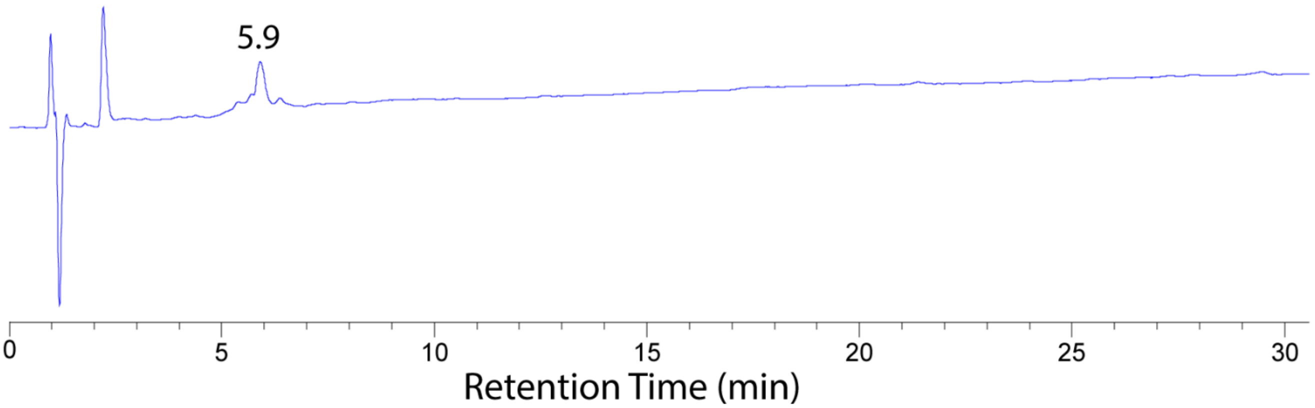

HRMS for Product 3b from MP Lysine Formation Using Peptide 1b

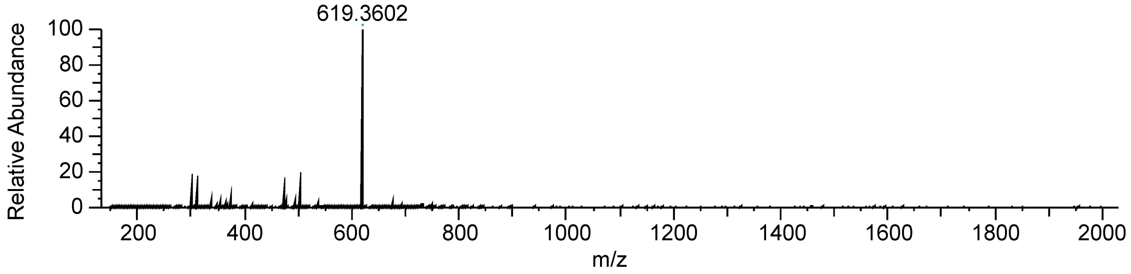

Fig. S7 – Synthesis of MP on small molecule

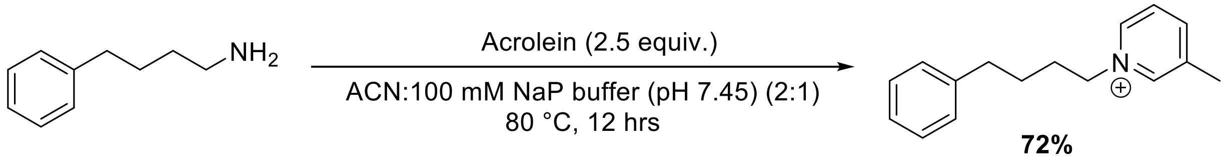

4-Phenylbutylamine (106 µL, 0.67 mmol, 1 equiv.) was added to a 35 mL high-pressure tube and dissolved in 2:1 ACN:100 mM NaP buffer (pH 7.45) (4 mL:2 mL). Acrolein (112 µL, 1.68 mmol, 2.5 equiv.) was added to the reaction mixture. The high-pressure tube was sealed, and the reaction was stirred at 80 °C for 12 hours. After 12 hours, reaction mixture was transferred to a separatory funnel, diluted with H_2O (10 mL) and washed with EtOAc (3 x 15 mL) to remove any organic impurities; the positively charged MP product migrated to the aqueous layer. The aqueous layer was concentrated on the SpeedVac. The aqueous layer was then purified using preparatory HPLC, and fractions containing the MP product were lyophilized to afford the product as a dark yellow liquid (108 mg, 72% yield).

^{1}H NMR (400 MHz, $CDCl_3$): δ = 9.12 (d, *J* = 5.9 Hz, 1H), 8.75 (s, 1H), 8.11 (d, *J* = 7.7 Hz, 1H), 7.89 (t, *J* = 7.0 Hz, 1H), 7.26 (d, *J* = 16.6 Hz, 2H), 7.16 (d, *J* = 29.9 Hz, 3H), 4.84 (t, *J* = 7.6 Hz, 2H), 2.65 (t, *J* = 7.5 Hz, 2H), 2.58 (s, 3H), 2.07 – 1.96 (m, 2H), 1.73 – 1.63 (m, 2H) ppm. **^{13}C NMR** (101 MHz, $CDCl_3$): δ = 168.33, 145.23, 144.97, 142.72, 141.20, 139.86, 128.65, 128.58,

127.76, 126.29, 61.86, 35.14, 31.34, 27.72, 18.80 ppm. **HRMS**: calcd. for $C_{16}H_{20}N^{+}$ [M] 226.1590; found 226.1590.

^{1}H NMR of MP Product

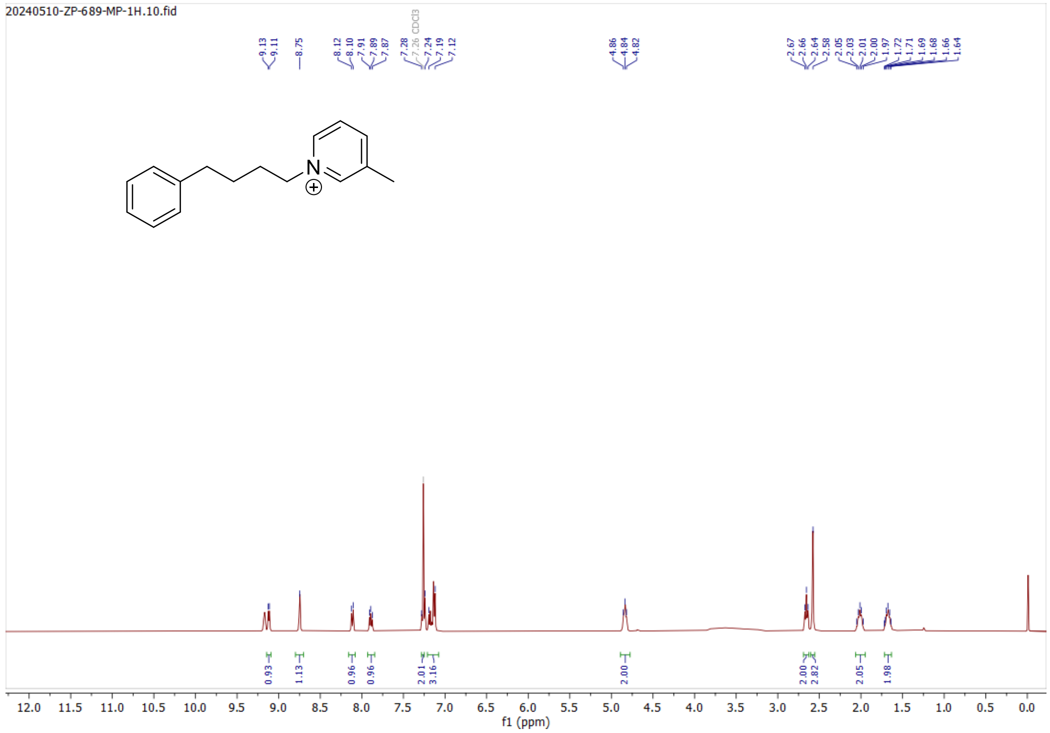

^{13}C NMR of MP Product

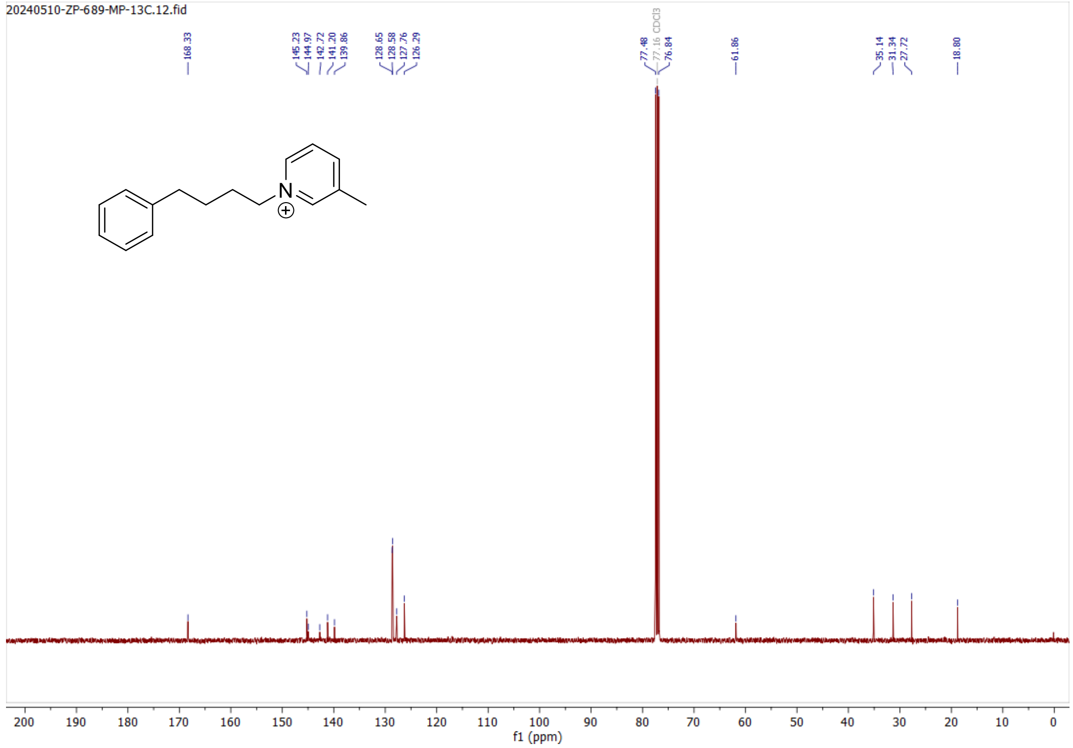

HRMS of MP Product

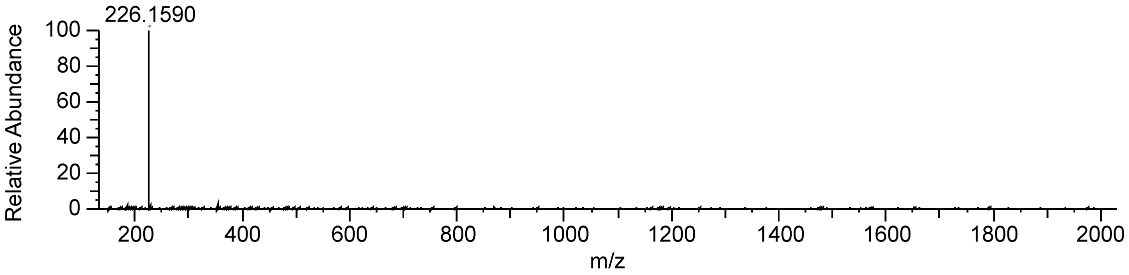

Fig. S8 – Reaction of 1a with methacrolein

Reaction of 1a with Methacrolein at 37 °C

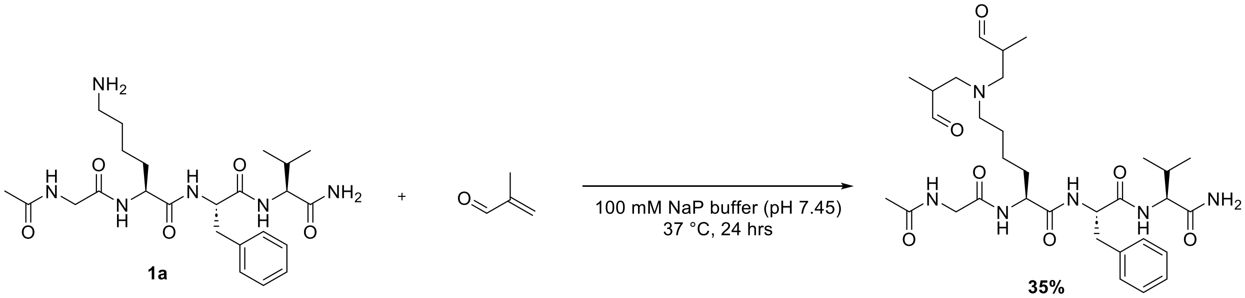

Ac-GKFV **1a** (2 mg, 4.1 µmol, 1 equiv.) was dissolved in 580 µL of 100 mM NaP buffer (pH 7.45) in a 1” dram vial. Methacrolein (1.68 µL, 20.4 µmol, 5 equiv.) was added to the vial from a freshly prepared stock solution (20 µL). The reaction was stirred at 37 °C for 24 hours, after which the reaction was analyzed using **HPLC Method A**, revealing 35% conversion to a **Methacrolein Double Addition Product.**

Ac-GKFV-CONH$_2$ (1a) peptide. LCMS, m/z 491.883 (calcd. [M+H$^+$] = 491.2976), Purity: >99% (HPLC analysis at 220 nm). Retention time in HPLC: 5.6 min.

Methacrolein Double Addition Product. LCMS, m/z 631.3697 (calcd. [M+H$^+$] = 631.3814), m/z 653.3508 (calcd. [M+Na$^+$] = 653.3633), Purity: >99% (HPLC analysis at 220 nm). Retention time in HPLC: 6.8 min.

HPLC Trace for Reaction of 1a with Methacrolein at 37 °C

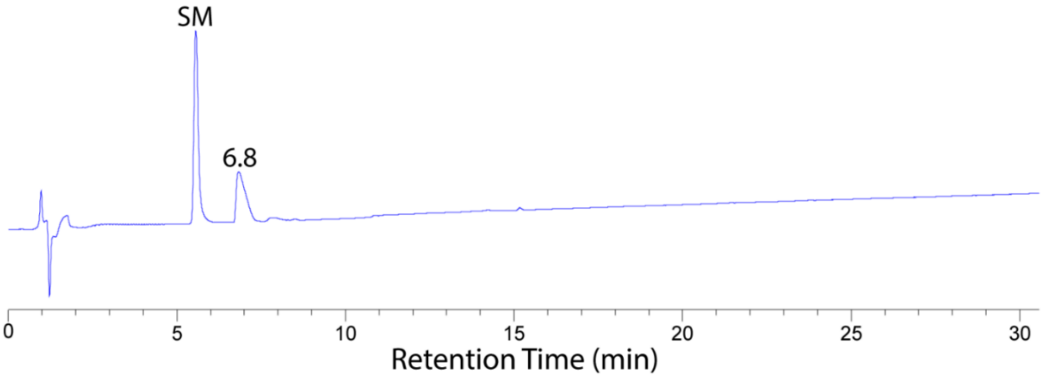

HRMS of Double Michael Addition Product with Methacrolein

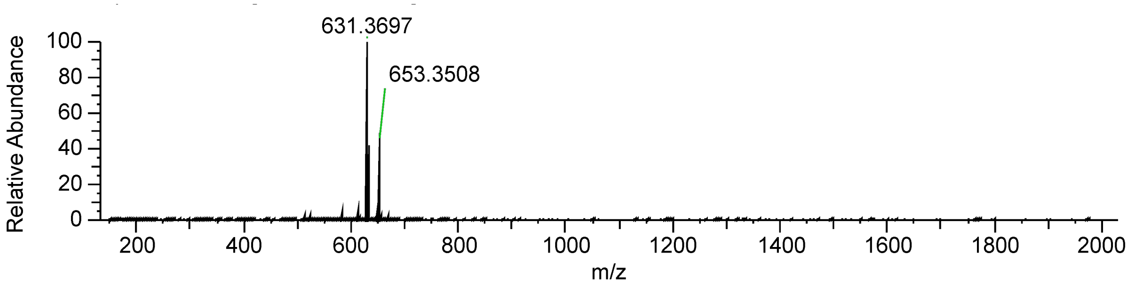

Reaction of 1a with Methacrolein at 80 °C

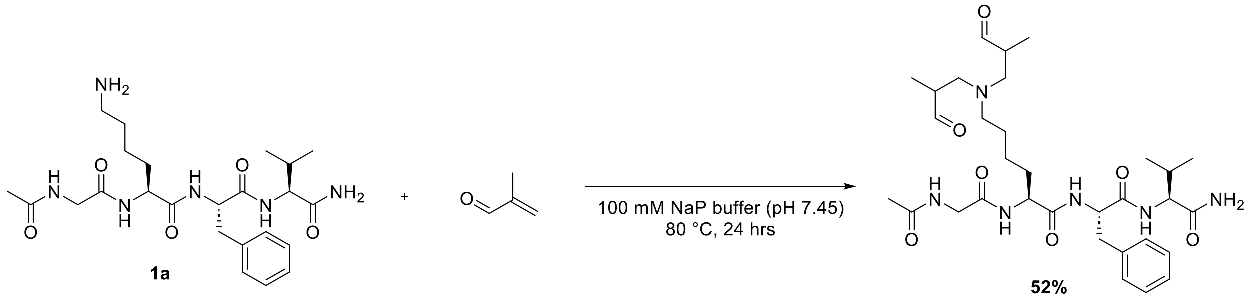

Ac-GKFV **1a** (2 mg, 4.1 µmol, 1 equiv.) was dissolved in 580 µL of 100 mM NaP buffer (pH 7.45) in a 1" dram vial. Methacrolein (1.68 µL, 20.4 µmol, 5 equiv.) was added to the vial from a freshly prepared stock solution (20 µL). The reaction was stirred at 80 °C for 24 hours, after which the reaction was analyzed using **HPLC Method A**, revealing 52% conversion to a **Methacrolein Double Addition Product.**

Ac-GKFV-CONH$_2$ (1a) peptide. LCMS, m/z 491.2883 (calcd. [M+H$^+$] = 491.2976), Purity: >99% (HPLC analysis at 220 nm). Retention time in HPLC: 5.6 min.

Methacrolein Double Addition Product. LCMS, m/z 631.3697 (calcd. [M+H$^+$] = 631.3814), m/z 653.3508 (calcd. [M+Na$^+$] = 653.3633), Purity: >99% (HPLC analysis at 220 nm). Retention time in HPLC: 6.8 min.

HPLC Trace for Reaction of 1a with Methacrolein at 80 °C

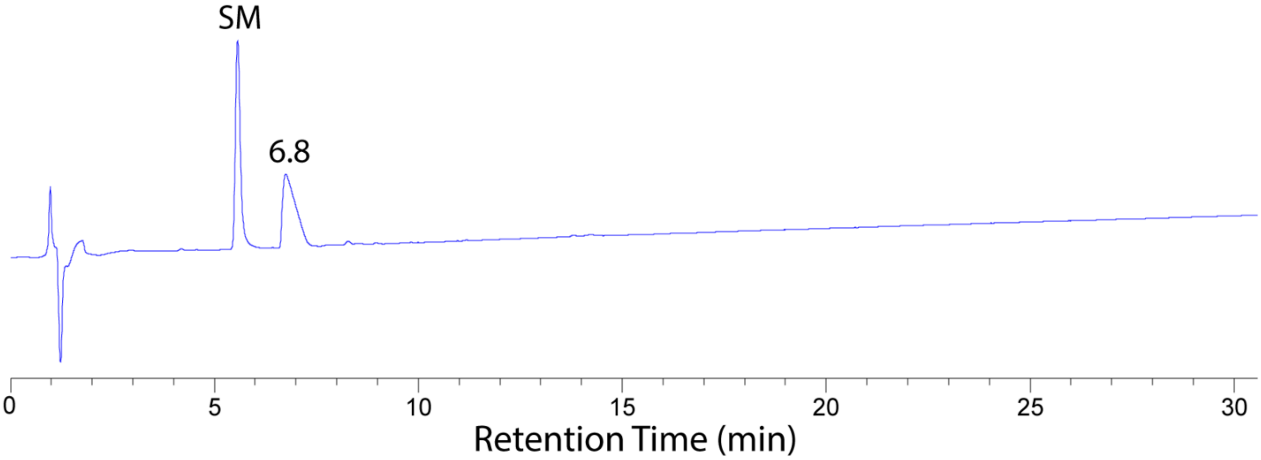

Fig. S9 – Reaction of 1a with crotonaldehyde

Reaction of 1a with Crotonaldehyde and No Silver

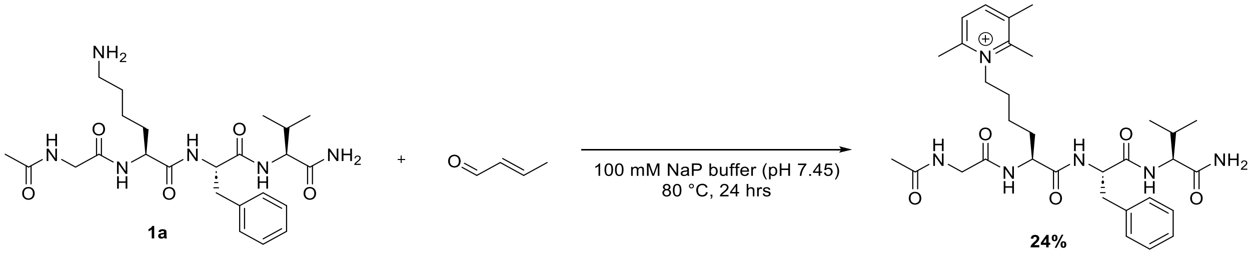

Ac-GKFV **1a** (2 mg, 4.1 µmol, 1 equiv.) was dissolved in 580 µL of 100 mM NaP buffer (pH 7.45) in a 1” dram vial. Crotonaldehyde (mixture of cis and trans) (1.67 µL, 20.4 µmol, 5 equiv.) was added to the vial from a freshly prepared stock solution (20 µL). The reaction was stirred at 80 °C for 24 hours, after which the reaction was analyzed using **HPLC Method A**, revealing 24% conversion to Substituted MP Lysine Product **3a’**.

Ac-GKFV-CONH$_2$ (1a) peptide. LCMS, m/z 491.2883 (calcd. [M+H$^+$] = 491.2976), Purity: >99% (HPLC analysis at 220 nm). Retention time in HPLC: 4.9 min.

Substituted MP Lysine Product 3a’. LCMS, m/z 595.3459 (calcd. [M] = 595.3602), Purity: >99% (HPLC analysis at 220 nm). Retention time in HPLC: 8.8 min.

HPLC Trace for Reaction of 1a with Crotonaldehyde and No Silver

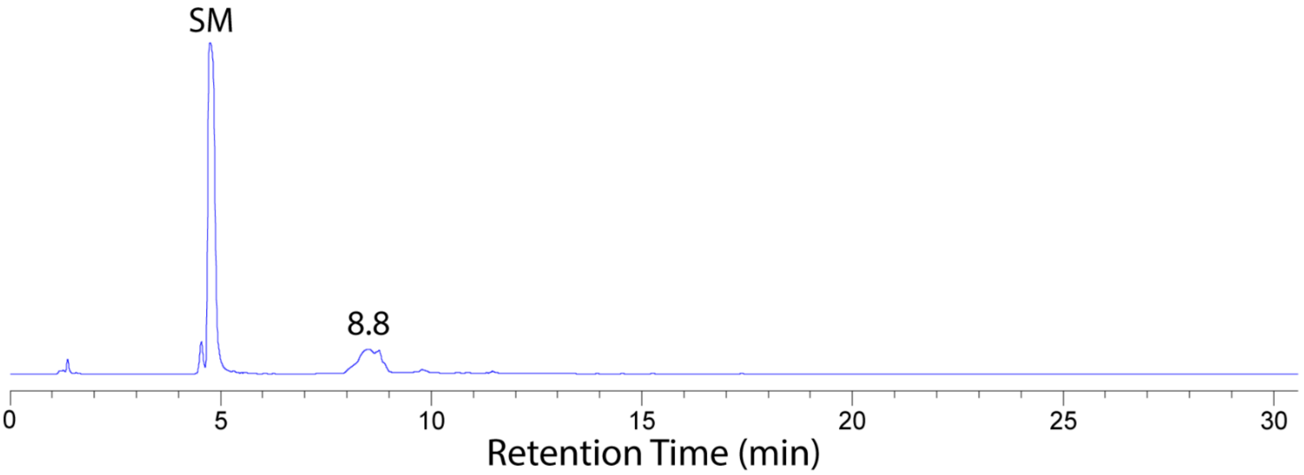

HRMS of MP Product from Reaction of 1a with Crotonaldehyde

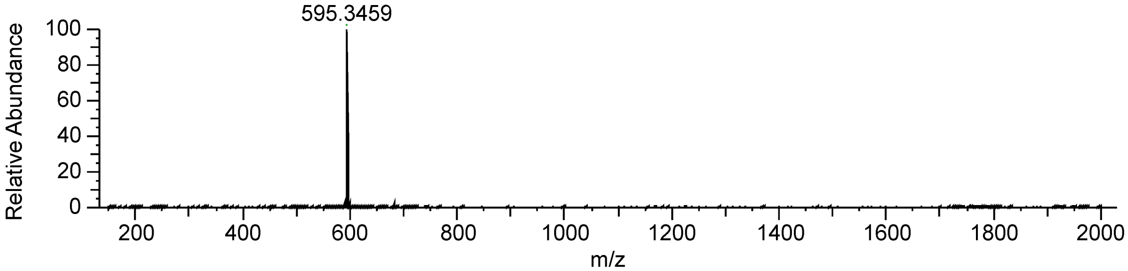

Reaction of 1a with Crotonaldehyde and Varying Equivalents of Ag_2O and Base

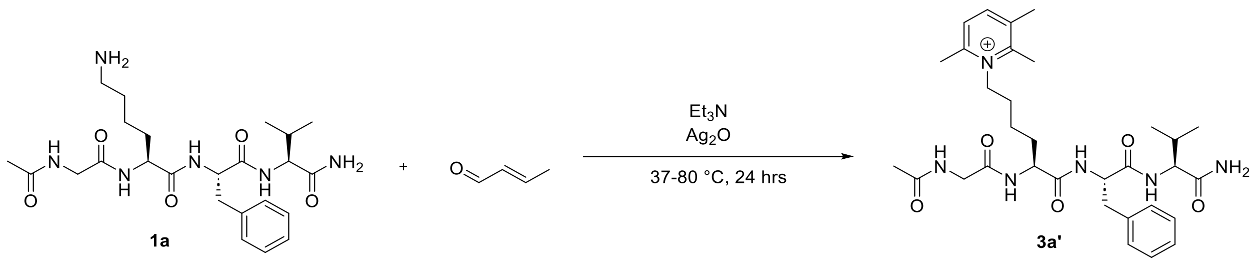

Ac-GKFV **1a** (2 mg, 4.1 µmol, 1 equiv.) was dissolved in 500 µL of the solvent system listed in the table below in a 1" dram vial. Crotonaldehyde (mixture of cis and trans) (1.67 µL, 20.4 µmol, 5 equiv.) was added to the vial from a freshly prepared stock solution (20 µL). Next, the designated amount of Ag_2O was transferred to the reaction from a stock solution (60 µL). Finally, the listed quantity of Et_3N was added to the vial, again from a stock solution (20 µL). The reaction was stirred at the listed temperature for 24 hours, after which the reaction was analyzed using **HPLC Method A**. See table below for the conversion to Substituted MP Lysine Product **3a'** under each attempted condition.

Entry	Temp	Equiv. Ag_2O	Equiv. Et_3N	Solvent	Conversion to Substituted MP Product **3a'**
1	80 °C	2 equiv.	None	100 mM NaP buffer (pH 7.45)	7%
2	80 °C	2 equiv.	5 equiv.	THF:ACN:H_2O (1:1:1)	<5%
3	37 °C	50 mol %	5 equiv.	100 mM NaP buffer (pH 7.45)	16%
4	80 °C	50 mol %	5 equiv.	100 mM NaP buffer (pH 7.45)	10%
5	37 °C	50 mol %	None	100 mM NaP buffer (pH 7.45)	19%
6	80 °C	50 mol %	None	100 mM NaP buffer (pH 7.45)	8%

HPLC Trace for Crotonaldehyde and Ag_2O Reaction Entry 1

Ac-GKFV-$CONH_2$ (1a) peptide. LCMS, m/z 491.2883 (calcd. $[M+H^+]$ = 491.2976), Purity: >99% (HPLC analysis at 220 nm). Retention time in HPLC: 4.6 min.

Substituted MP Lysine Product 3a'. LCMS, m/z 595.3459 (calcd. [M] = 595.3602), Purity: >99% (HPLC analysis at 220 nm). Retention time in HPLC: 7.2 min.

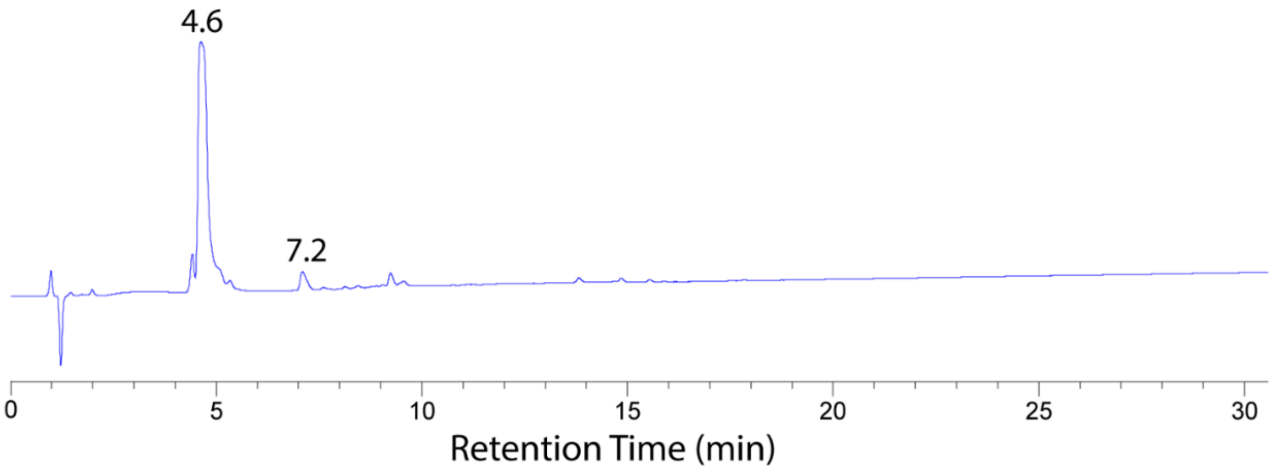

HPLC Trace for Crotonaldehyde and Ag_2O Reaction Entry 2

Ac-GKFV-CONH$_2$ (1a) peptide. LCMS, m/z 491.2883 (calcd. [M+H$^+$] = 491.2976), Purity: >99% (HPLC analysis at 220 nm). Retention time in HPLC: 4.7 min.

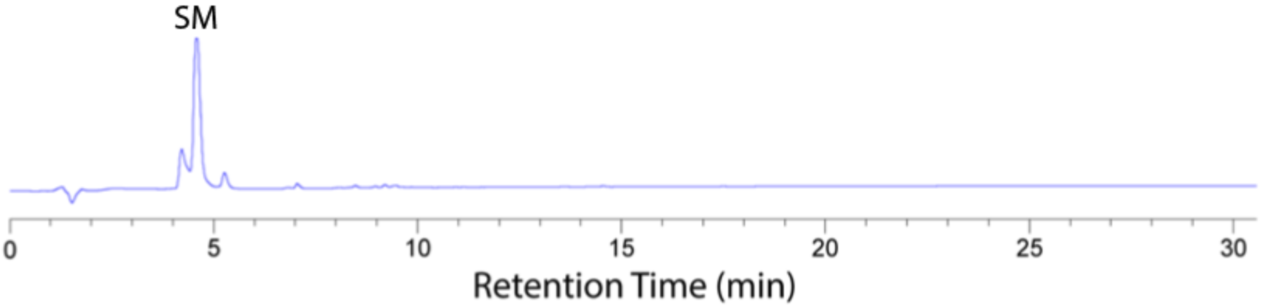

HPLC Trace for Crotonaldehyde and Ag_2O Reaction Entry 3

Ac-GKFV-CONH$_2$ (1a) peptide. LCMS, m/z 491.2883 (calcd. [M+H$^+$] = 491.2976), Purity: >99% (HPLC analysis at 220 nm). Retention time in HPLC: 4.6 min.

Substituted MP Lysine Product 3a'. LCMS, m/z 595.3459 (calcd. [M] = 595.3602), Purity: >99% (HPLC analysis at 220 nm). Retention time in HPLC: 8.6 min.

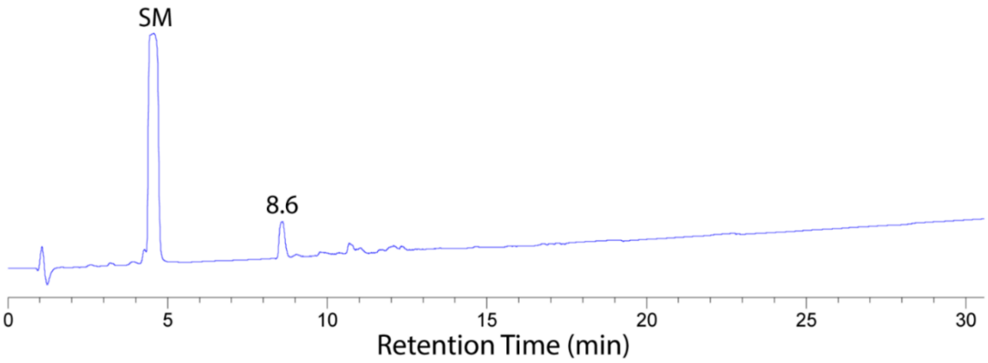

HPLC Trace for Crotonaldehyde and Ag_2O Reaction Entry 4

Ac-GKFV-CONH$_2$ (1a) peptide. LCMS, m/z 491.2883 (calcd. [M+H$^+$] = 491.2976), Purity: >99% (HPLC analysis at 220 nm). Retention time in HPLC: 4.6 min.

Substituted MP Lysine Product 3a'. LCMS, m/z 595.3459 (calcd. [M] = 595.3602), Purity: >99% (HPLC analysis at 220 nm). Retention time in HPLC: 8.6 min.

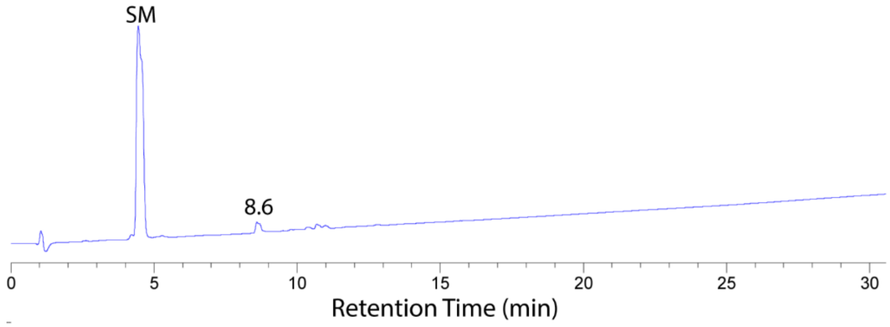

HPLC Trace for Crotonaldehyde and Ag_2O Reaction Entry 5

Ac-GKFV-CONH$_2$ (1a) peptide. LCMS, m/z 491.2883 (calcd. [M+H$^+$] = 491.2976), Purity: >99% (HPLC analysis at 220 nm). Retention time in HPLC: 4.6 min.

Substituted MP Lysine Product 3a'. LCMS, m/z 595.3459 (calcd. [M] = 595.3602), Purity: >99% (HPLC analysis at 220 nm). Retention time in HPLC: 8.6 min.

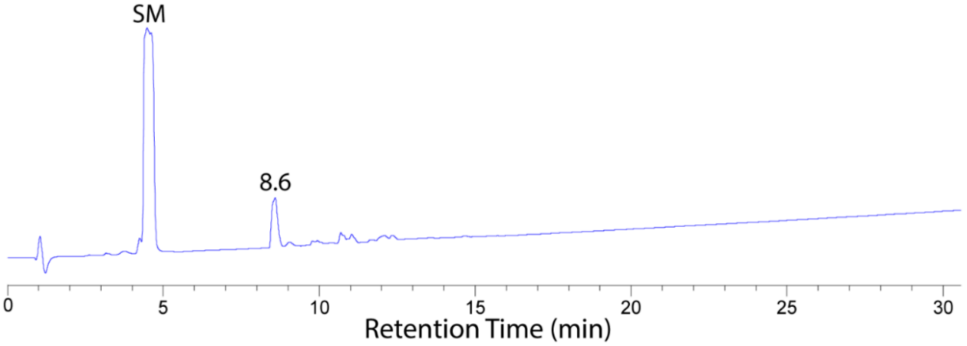

HPLC Trace for Crotonaldehyde and Ag_2O Reaction Entry 6

Ac-GKFV-CONH$_2$ (1a) peptide. LCMS, m/z 491.2883 (calcd. [M+H$^+$] = 491.2976), Purity: >99% (HPLC analysis at 220 nm). Retention time in HPLC: 4.6 min.

Substituted MP Lysine Product 3a'. LCMS, m/z 595.3459 (calcd. [M] = 595.3602), Purity: >99% (HPLC analysis at 220 nm). Retention time in HPLC: 8.6 min.

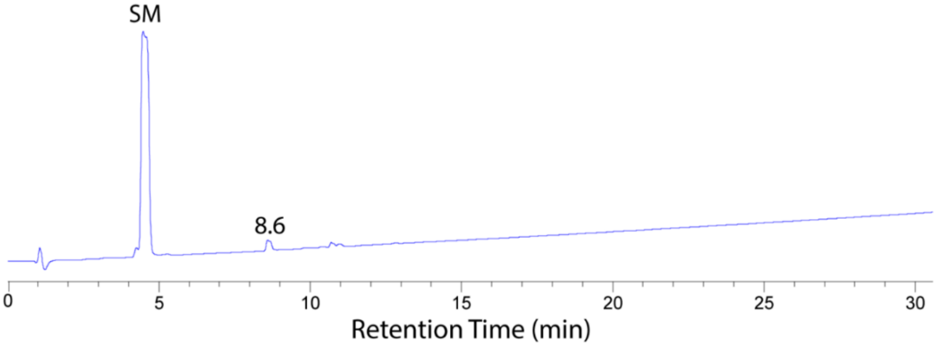

Reaction of 1a with Crotonaldehyde and AgOAc at 37 °C

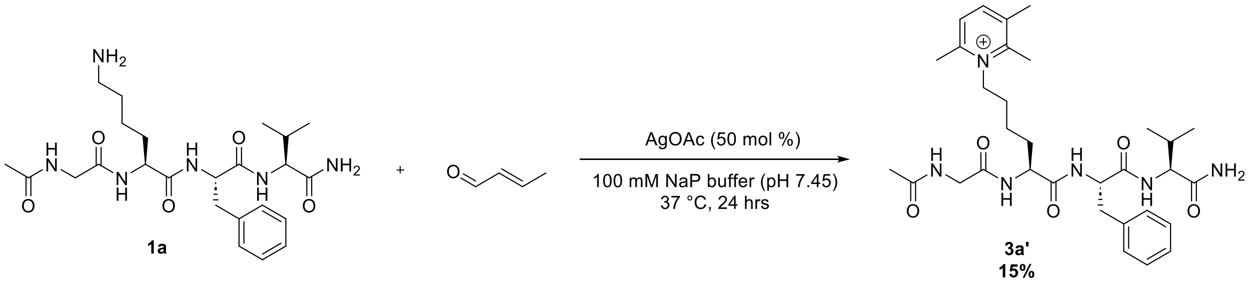

Ac-GKFV **1a** (2 mg, 4.08 µmol, 1 equiv.) was dissolved in 530 µL of 100 mM NaP buffer (pH 7.45) in a 1" dram vial. Crotonaldehyde (mixture of cis and trans) (1.67 µL, 20.4 µmol, 5 equiv.)

was added to the vial from a freshly prepared stock solution (20 µL). Next, AgOAc (0.34 mg, 2.04 µmol, 50 mol %) was transferred to the reaction, again from a stock solution (50 µL). The reaction was stirred at 37 °C for 24 hours, after which the reaction was analyzed using **HPLC Method A**, revealing 15% conversion to the Substituted MP Lysine Product **3a'**.

Ac-GKFV-CONH₂ (1a) peptide. LCMS, m/z 491.2883 (calcd. [M+H$^+$] = 491.2976), Purity: >99% (HPLC analysis at 220 nm). Retention time in HPLC: 4.7 min.

Substituted MP Lysine Product 3a'. LCMS, m/z 595.3459 (calcd. [M] = 595.3602), Purity: >99% (HPLC analysis at 220 nm). Retention time in HPLC: 8.3 min.

HPLC Trace for Reaction of 1a with Crotonaldehyde and AgOAc at 37 °C

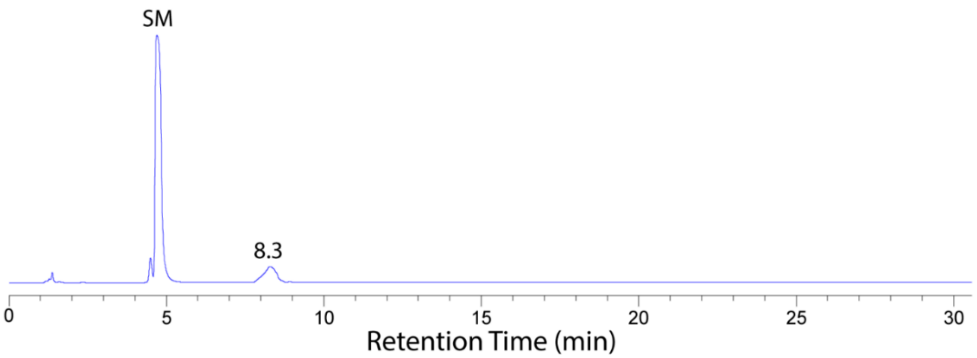

Reaction of 1a with Crotonaldehyde and AgOAc at 80 °C

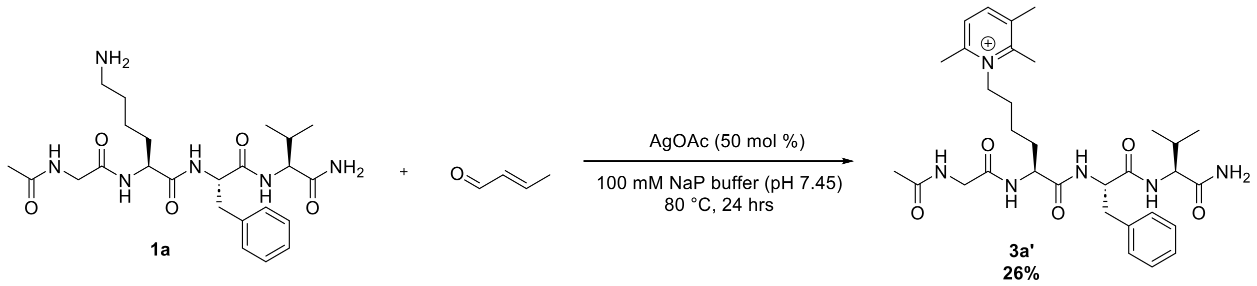

Ac-GKFV **1a** (2 mg, 4.08 µmol, 1 equiv.) was dissolved in 530 µL of 100 mM NaP buffer (pH 7.45) in a 1" dram vial. Crotonaldehyde (mixture of cis and trans) (1.67 µL, 20.4 µmol, 5 equiv.) was added to the vial from a freshly prepared stock solution (20 µL). Next, AgOAc (0.34 mg, 2.04 µmol, 50 mol %) was transferred to the reaction, again from a stock solution (50 µL). The reaction was stirred at 80 °C for 24 hours, after which the reaction was analyzed using **HPLC Method A**, revealing 26% conversion to the Substituted MP Lysine Product **3a'**.

Ac-GKFV-CONH₂ (1a) peptide. LCMS, m/z 491.2883 (calcd. [M+H$^+$] = 491.2976), Purity: >99% (HPLC analysis at 220 nm). Retention time in HPLC: 4.8 min.

Substituted MP Lysine Product 3a'. LCMS, m/z 595.3459 (calcd. [M] = 595.3602), Purity: >99% (HPLC analysis at 220 nm). Retention time in HPLC: 8.6 min.

HPLC Trace for Reaction of 1a with Crotonaldehyde and AgOAc at 80 °C

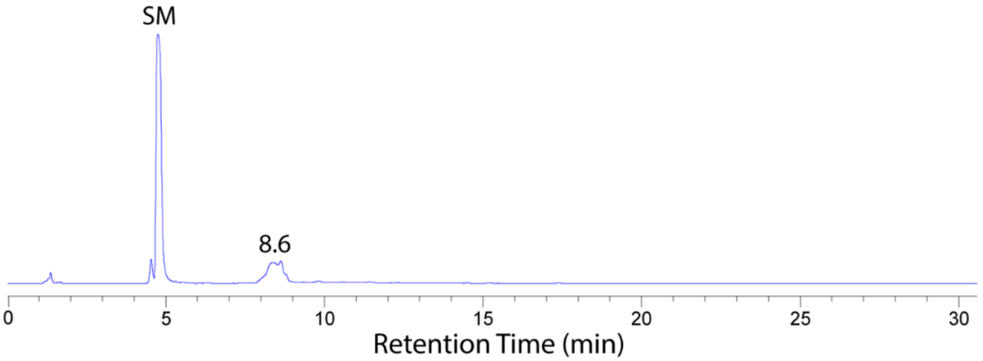

Fig. S10 – Chemoselectivity peptide Ac-KQYWRMES 1c

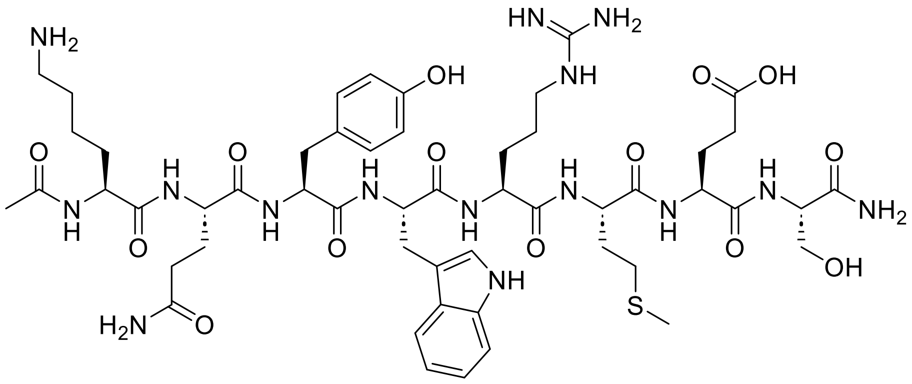

1c

Ac-KQYWRMES-CONH₂ (1c) peptide. LCMS, m/z 1168.5566 (calcd. $[M+H^+]$ = 1168.5568), m/z 584.7821 (calcd. $[(M+2H^+)/2]$ = 584.7823), Purity: >99% (HPLC analysis at 220 nm). Retention time in HPLC: 10.9 min.

HPLC Trace of Ac-KQYWRMES 1c

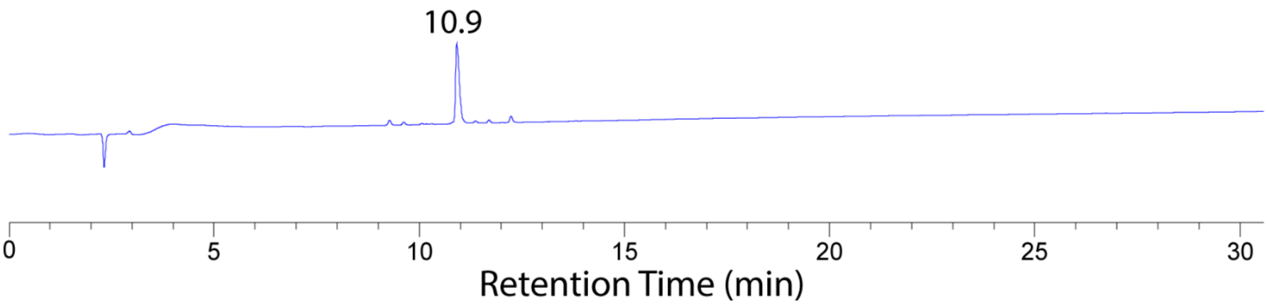

HRMS of Ac-KQYWRMES 1c

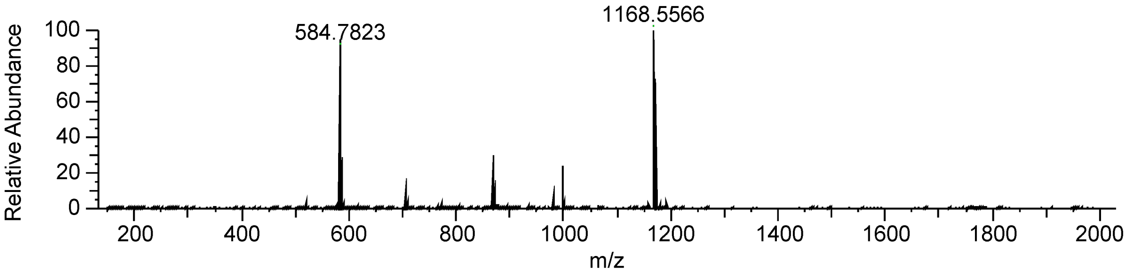

Formation of Chemoselectivity Peptide Ac-KQYWRMES FDP Product 2c

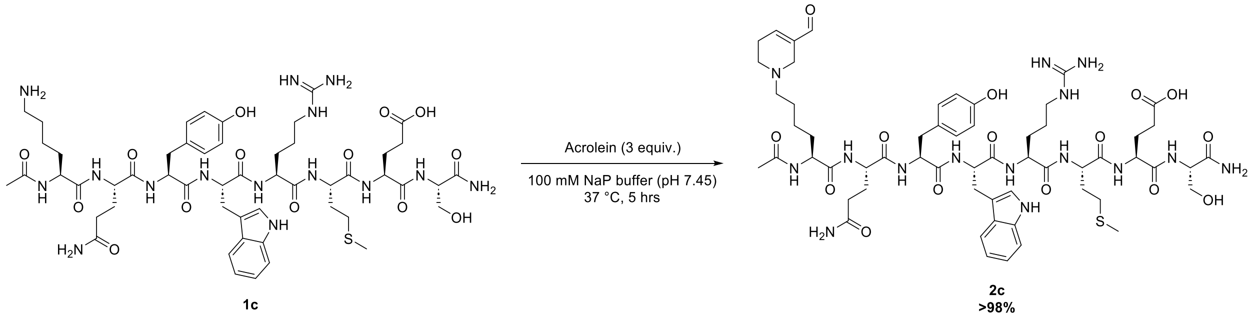

Ac-KQYWRMES **1c** (2 mg, 1.71 µmol, 1 equiv.) was dissolved in 580 µL of 100 mM NaP buffer (pH 7.45) in a 1” dram vial. Acrolein (0.34 µL, 5.14 µmol, 3 equiv.) was added to the vial from a freshly prepared stock solution (20 µL). The mixture was stirred at 37 °C for 5 hours, after which the reaction was analyzed using **HPLC Method A**, revealing >98% conversion to FDP-modified product **2c**.

FDP Peptide 2c. LCMS, m/z 1262.6217 (calcd. [M+H$^+$] = 1262.5987), m/z 1284.6033 (calcd. [M+Na$^+$] = 1284.5806), m/z 631.8148 (calcd. [(M+2H$^+$)/2] = 631.8030), Purity: >99% (HPLC analysis at 220 nm). Retention time in HPLC: 11.1 min.

HPLC Trace for Formation of Chemoselectivity Peptide Ac-KQYWRMES FDP Product 2c

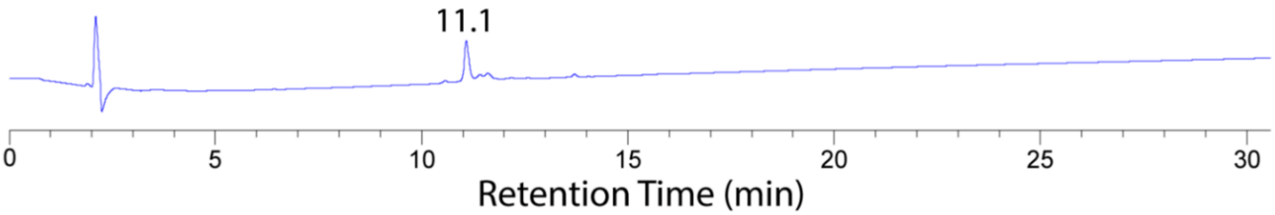

HRMS of Chemoselectivity Peptide Ac-KQYWRMES FDP Product 2c

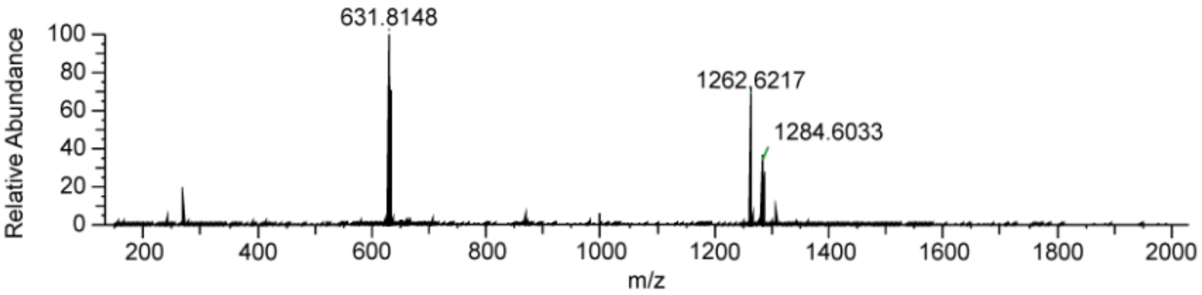

Fig. S11 – Acrolein histidine reactivity: incubation of Ac-GKFV 1a with Ac-GHFV 1d

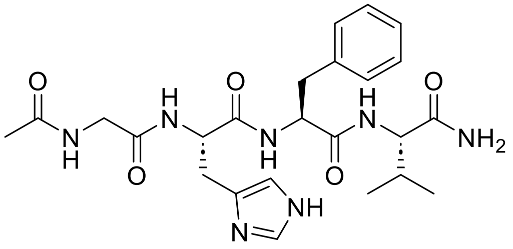

1d

Ac-GHFV-CONH$_2$ (1d) peptide. LCMS, m/z 500.2610 (calcd. [M+H$^+$] = 500.2616), Purity: >99% (HPLC analysis at 220 nm). Retention time in HPLC: 7.0 min.

HPLC Trace of Ac-GHFV 1d

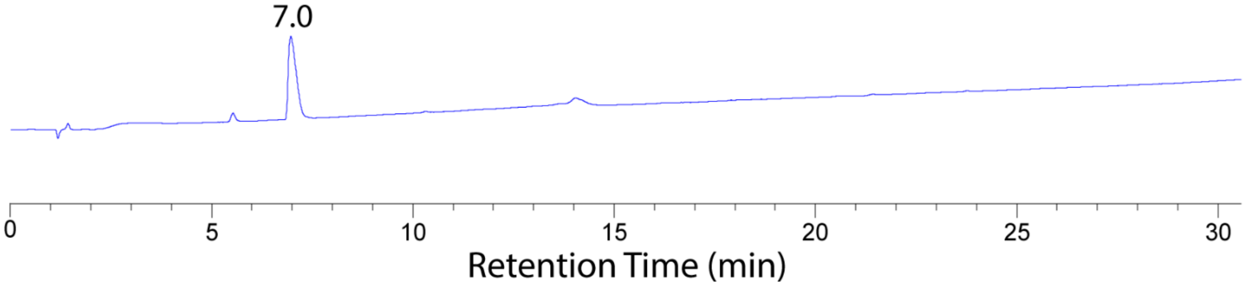

HRMS of Ac-GHFV 1d

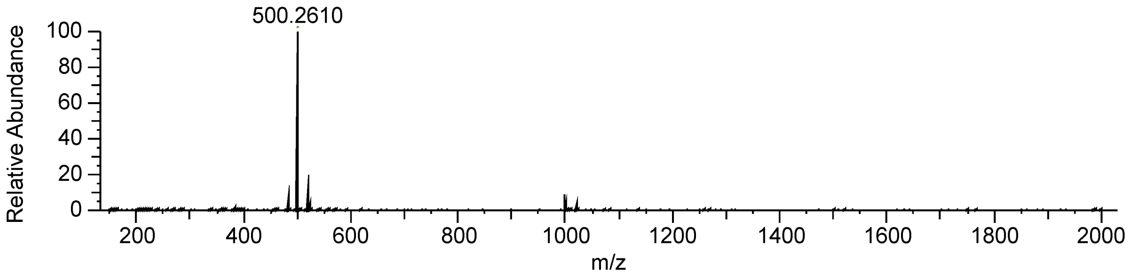

Incubation of Ac-GKFV 1a with Ac-GHFV 1d

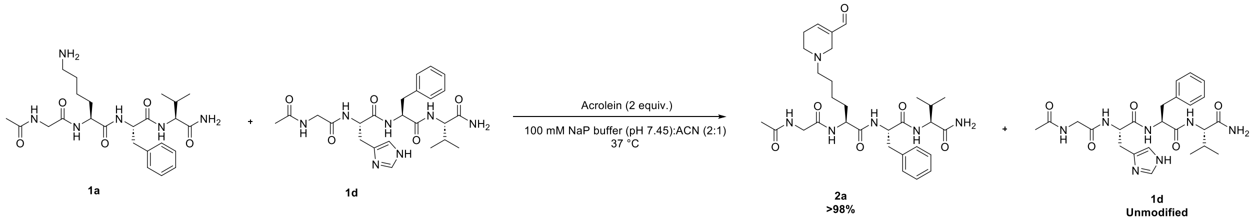

Ac-GKFV **1a** (1 mg, 2.0 µmol, 1 equiv.) and Ac-GHFV **1d** (1 mg, 2.0 µmol, 1 equiv.) were dissolved in 580 µL of 1:1 100 mM NaP buffer (pH 7.45):ACN in a 1" dram vial. Acrolein (0.27 µL, 4.1 µmol, 2 equiv.) was added to the vial from a freshly prepared stock solution (20 µL). The mixture was stirred at 37 °C for 5 hours, after which the reaction was analyzed using **HPLC Method A**, revealing >98% conversion to FDP-modified product **2a** and no modification of Ac-GHFV **1d**.

FDP Peptide 2a. LCMS, m/z 585.3393 (calcd. $[M+H^+]$ = 585.3395), Purity: >99% (HPLC analysis at 220 nm). Retention time in HPLC: 7.9 min.

Ac-GHFV-CONH₂ (1d) peptide. LCMS, m/z 500.2610 (calcd. $[M+H^+]$ = 500.2616), Purity: >99% (HPLC analysis at 220 nm). Retention time in HPLC: 6.8 min.

HPLC Trace for Incubation of Ac-GKFV 1a with Ac-GHFV 1d

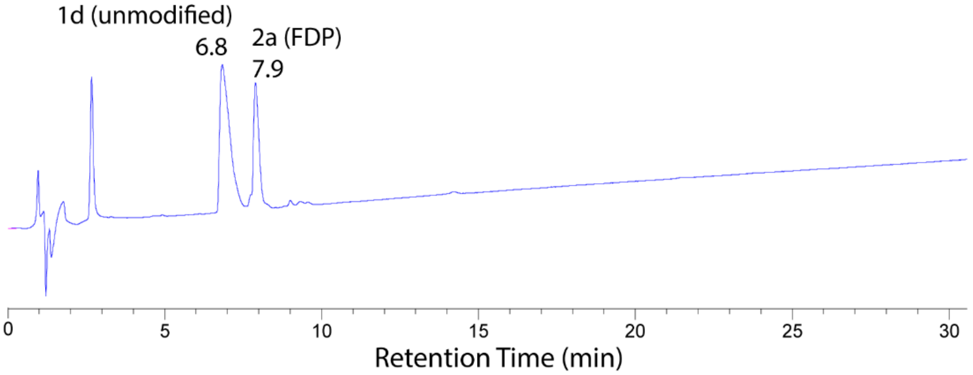

Fig. S12 – Acrolein cysteine reactivity: incubation of Ac-GKFV 1a with Ac-GCFV 1e

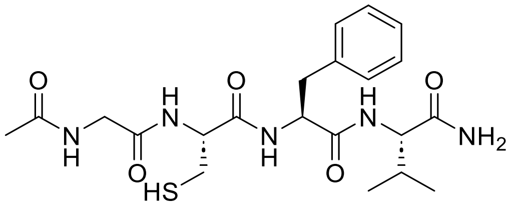

1e

Ac-GCFV-CONH₂ (1e) peptide. LCMS, m/z 488.1933 (calcd. [M+Na$^+$] = 488.1938), Purity: >99% (HPLC analysis at 220 nm). Retention time in HPLC: 10.6 min.

HPLC Trace of Ac-GCFV 1e

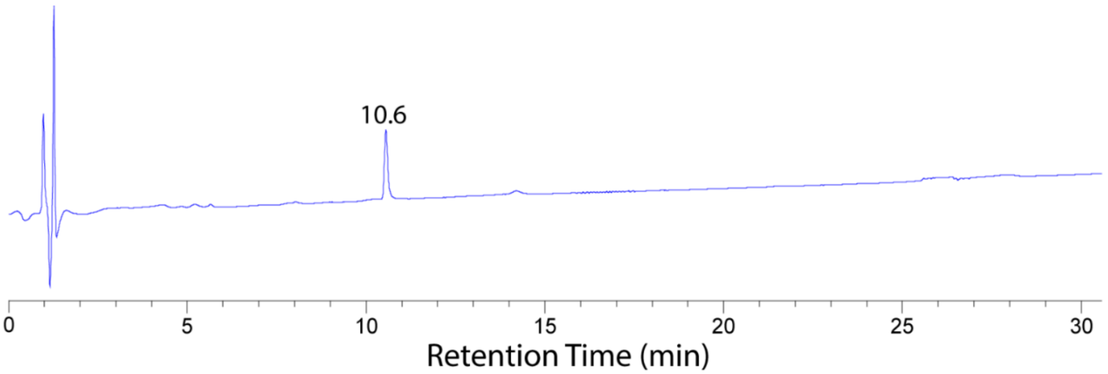

HRMS of Ac-GCFV 1e

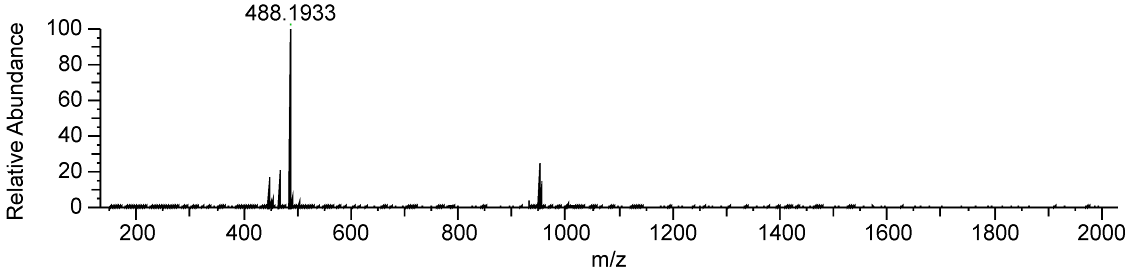

Incubation of Ac-GKFV 1a with Ac-GCFV 1e

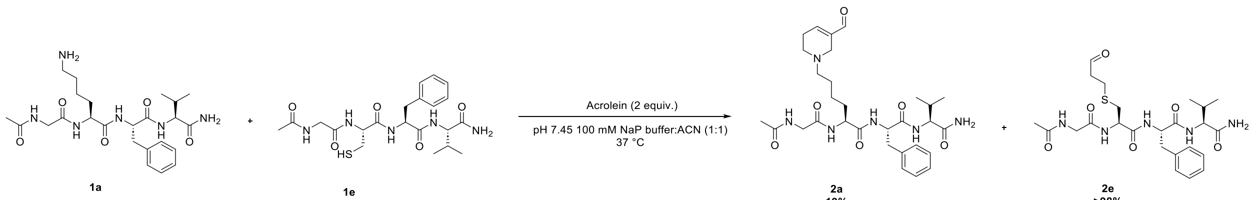

Ac-GKFV **1a** (1 mg, 2.0 µmol, 1 equiv.) and Ac-GCFV **1e** (1.05 mg, 2.0 µmol, 1 equiv.) were dissolved in 580 µL of 1:1 100 mM NaP buffer (pH 7.45):ACN in a 1" dram vial. Acrolein (0.27 µL, 4.1 µmol, 2 equiv.) was added to the vial from a freshly prepared stock solution (20 µL). The mixture was stirred at 37 °C for 5 hours, after which the reaction was analyzed using **HPLC Method A**, revealing >98% conversion to modified cysteine product **2e** and only 19% conversion to FDP-modified product **2a**.

Ac-GKFV-CONH₂ (1a) peptide. LCMS, m/z 491.2883 (calcd. [M+H$^+$] = 491.2976), Purity: >99% (HPLC analysis at 220 nm). Retention time in HPLC: 7.0 min.

FDP Peptide 2a. LCMS, m/z 585.3393 (calcd. [M+H$^+$] = 585.3395), Purity: >99% (HPLC analysis at 220 nm). Retention time in HPLC: 8.0 min.

Modified Cysteine Product 2e. LCMS, m/z 544.2197 (calcd. [M+Na$^+$] = 544.2200), Purity: >99% (HPLC analysis at 220 nm). Retention time in HPLC: 10.2 min.

HPLC Trace for Incubation of Ac-GKFV 1a with Ac-GCFV 1e

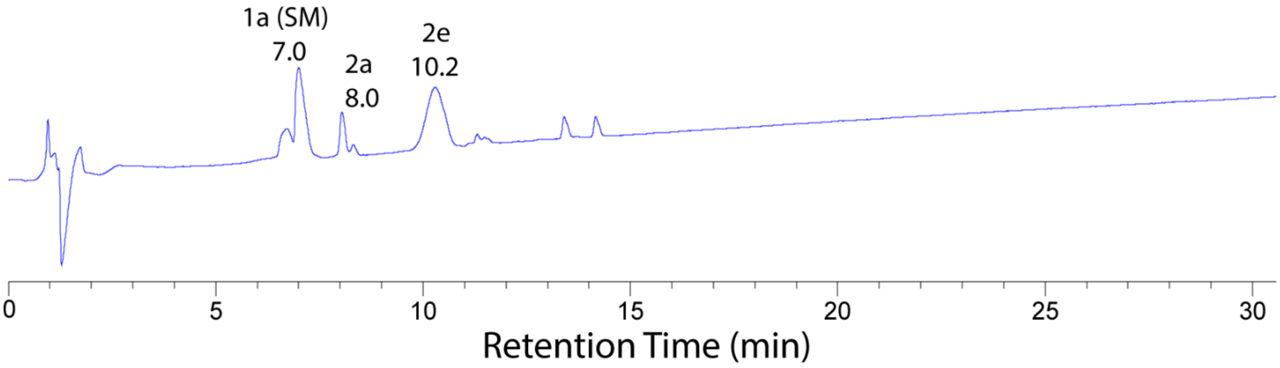

HRMS for Modified Cysteine Product 2e

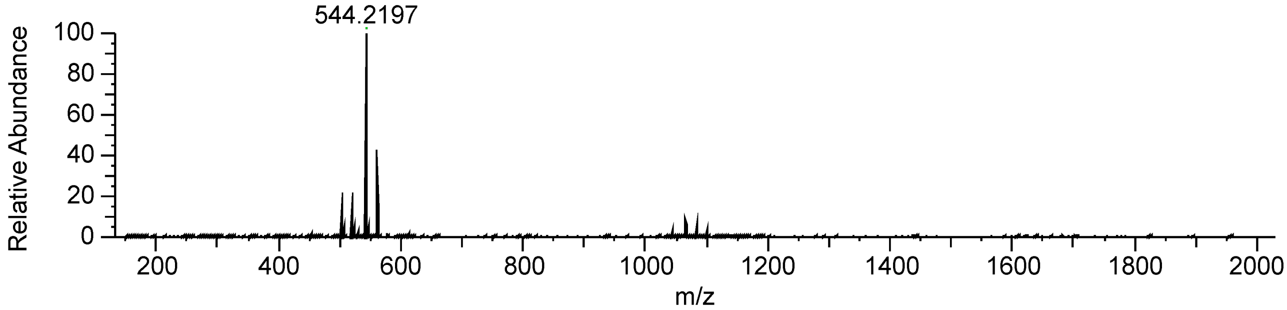

Fig. S13 – Formation of FDP lysine on bioactive peptides 1f-1i

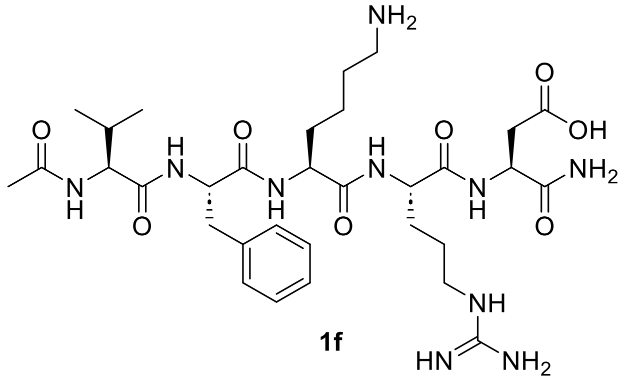

1f

Ac-VFKRD-CONH$_2$ (1f) peptide. LCMS, m/z 705.4030 (calcd. [M+H$^+$] = 705.4042), m/z 353.2050 (calcd. [(M+2H$^+$)/2] = 353.2058), Purity: >99% (HPLC analysis at 220 nm). Retention time in HPLC: 7.7 min.

HPLC Trace for Bioactive Peptide Ac-VFKRD 1f

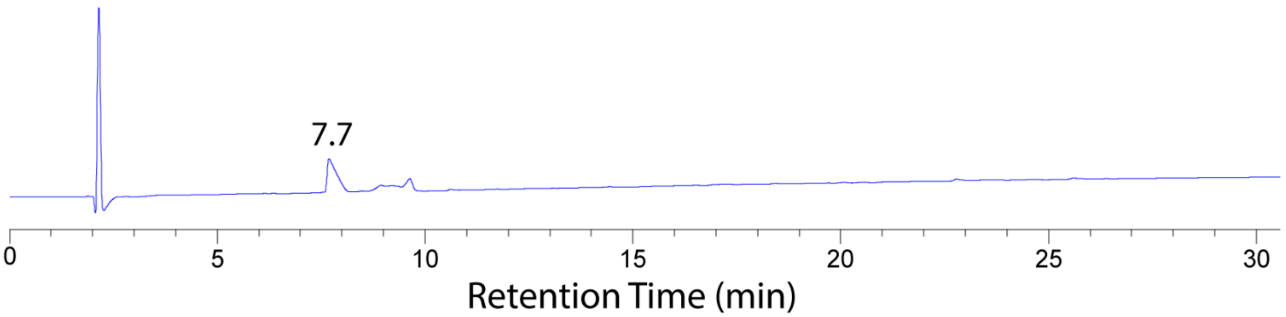

HRMS of Bioactive Peptide Ac-VFKRD 1f

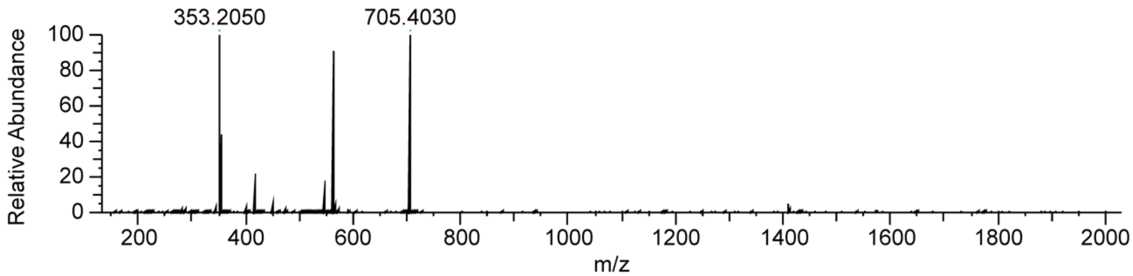

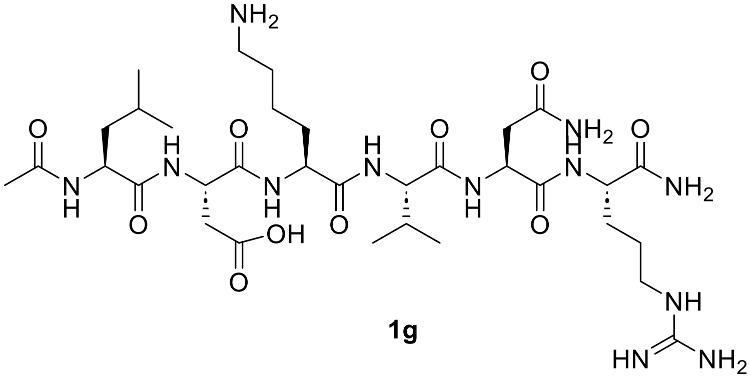

1g

Ac-LDKVNR-CONH₂ (1g) peptide. LCMS, m/z 785.4624 (calcd. $[M+H^+]$ = 785.4628), m/z 393.2347 (calcd. $[(M+2H^+)/2]$ = 393.2351), Purity: >99% (HPLC analysis at 220 nm). Retention time in HPLC: 5.7 min.

HPLC Trace for Bioactive Peptide Ac-LDKVNR 1g

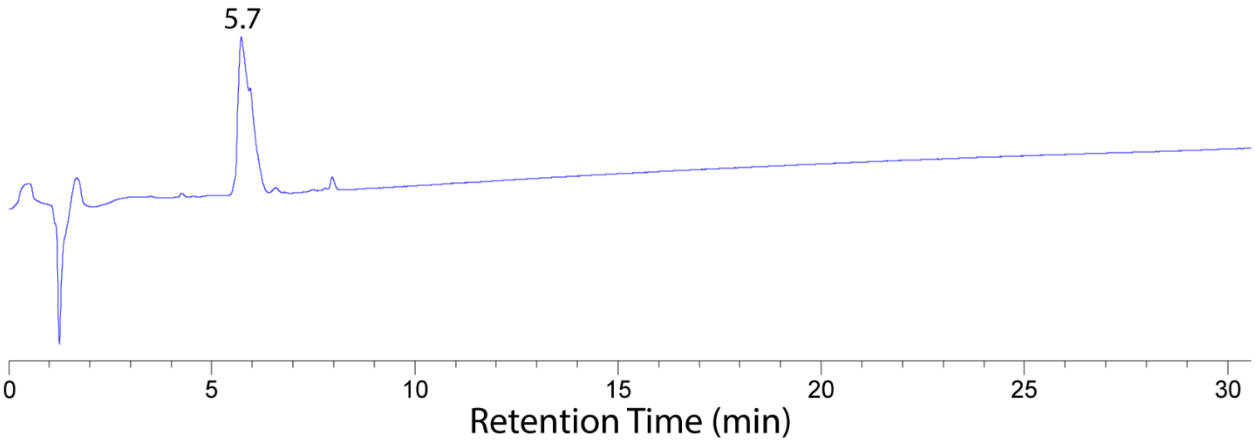

HRMS of Bioactive Peptide Ac-LDKVNR 1g

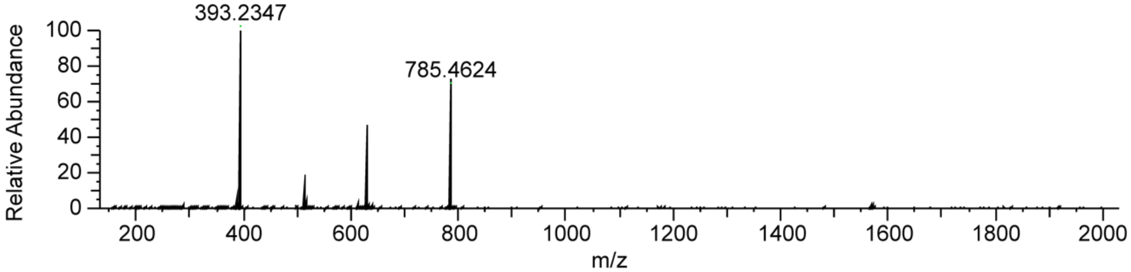

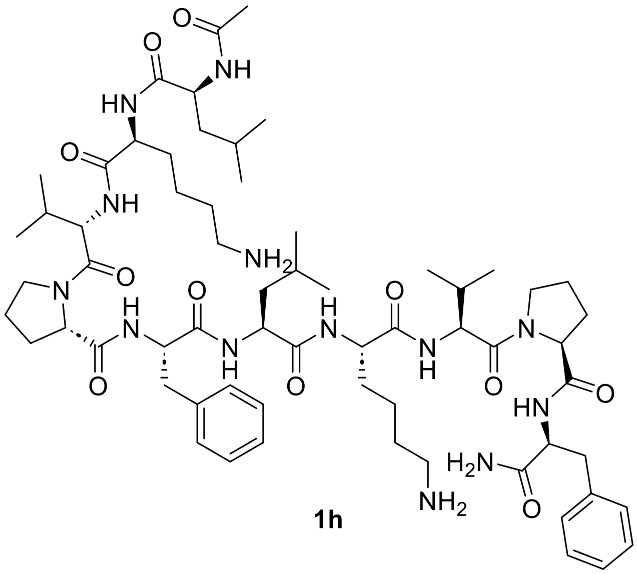

1h

Ac-LKVPFLKVPF-CONH₂ (1h) peptide. LCMS, m/z 1228.7816 (calcd. [M+H$^+$] = 1228.7816), m/z 1250.7626 (calcd. [M+Na$^+$] = 1250.7636), m/z 614.8947 (calcd. [(M+2H$^+$)/2] = 614.8945), Purity: >99% (HPLC analysis at 220 nm). Retention time in HPLC: 16.3 min.

HPLC Trace for Bioactive Peptide Ac-LKVPFLKVPF 1h

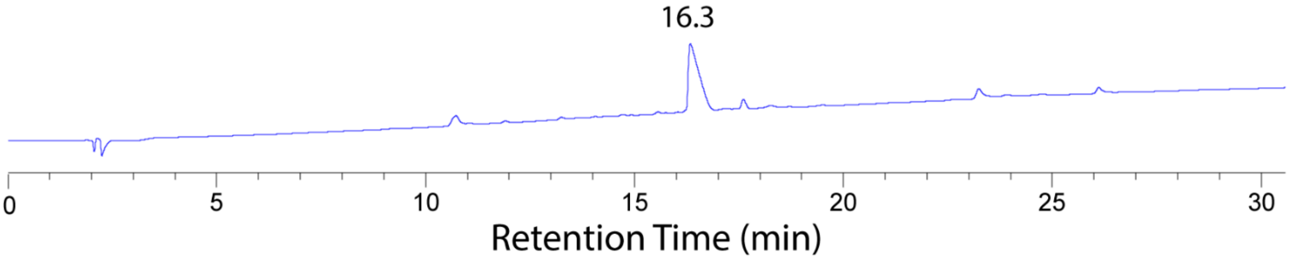

HRMS of Bioactive Peptide Ac-LKVPFLKVPF 1h

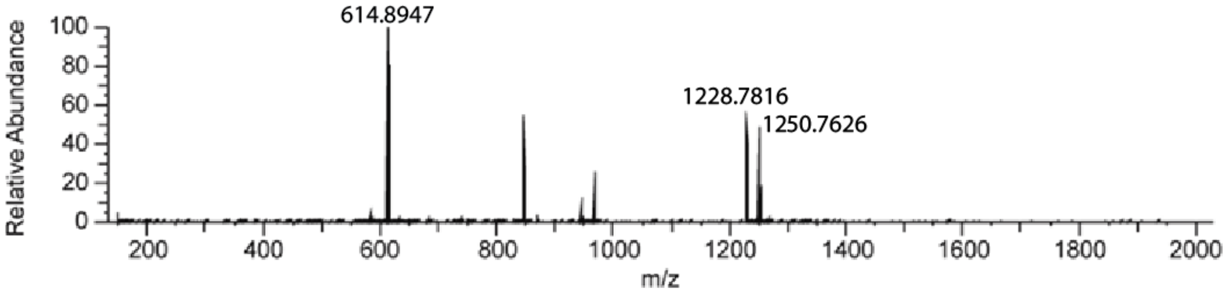

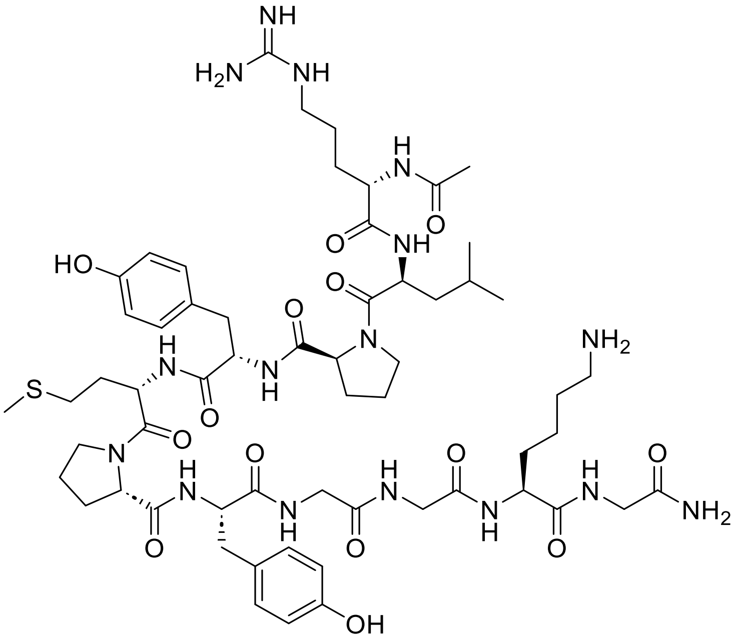

1i

Ac-RLPYMPYGGKG-CONH₂ (1i) peptide. LCMS, m/z 1279.6613 (calcd. [M+H$^+$] = 1279.6616), m/z 640.3348 (calcd. [(M+2H$^+$)/2] = 640.3345), Purity: >99% (HPLC analysis at 220 nm). Retention time in HPLC: 9.3 min.

HPLC Trace for Bioactive Peptide RLPYMPYGGKG 1i

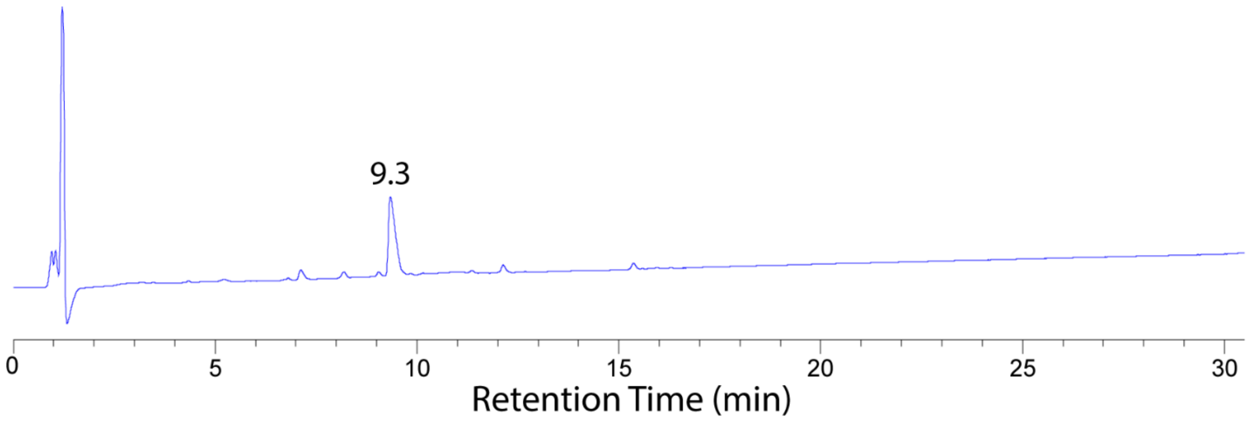

HRMS of Bioactive Peptide RLPYMPYGGKG 1i

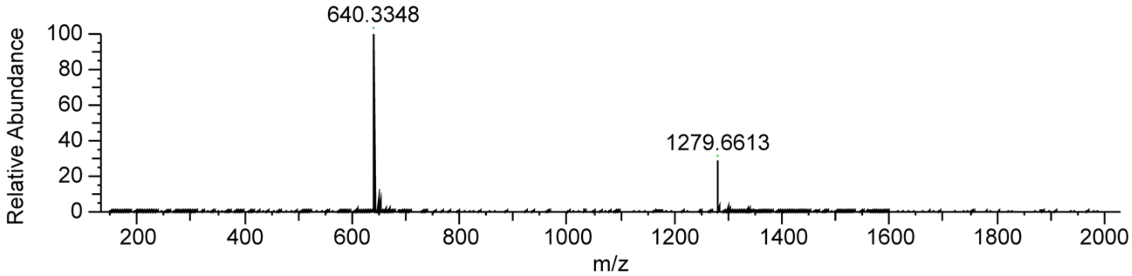

Formation of FDP Product 2f

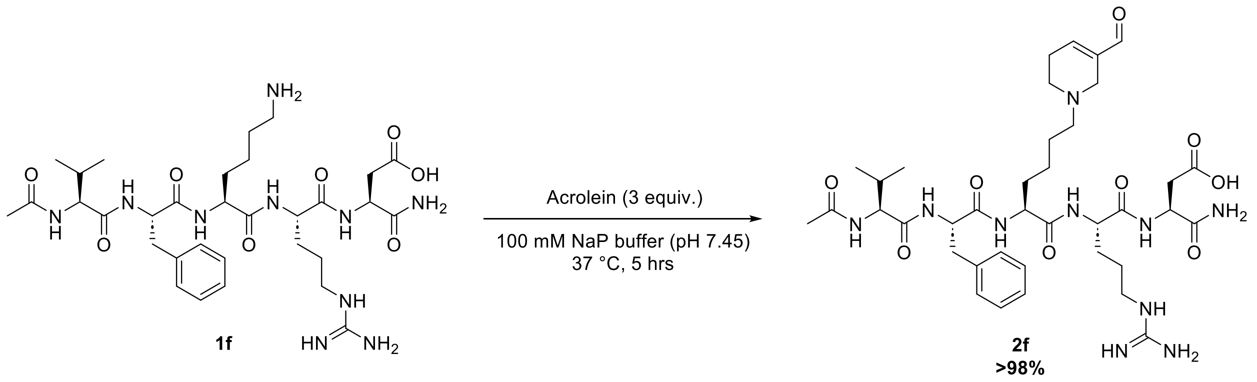

Ac-VFKRD **1f** (2 mg, 2.8 µmol, 1 equiv.) was dissolved in 580 µL of 100 mM NaP buffer (pH 7.45) in a 1” dram vial. Acrolein (0.57 µL, 8.5 µmol, 3 equiv.) was added to the vial from a freshly prepared stock solution (20 µL). The mixture was stirred at 37 °C for 5 hours, after which the reaction was analyzed using **HPLC Method A**, revealing >98% conversion to the FDP-modified product **2f**.

FDP Peptide 2f. LCMS, m/z 799.4476 (calcd. [M+H$^+$] = 799.4461), m/z 400.2274 (calcd. [(M+2H$^+$)/2] = 400.2267), Purity: >99% (HPLC analysis at 220 nm). Retention time in HPLC: 8.8 min.

HPLC Trace for Reaction Forming FDP Product 2f

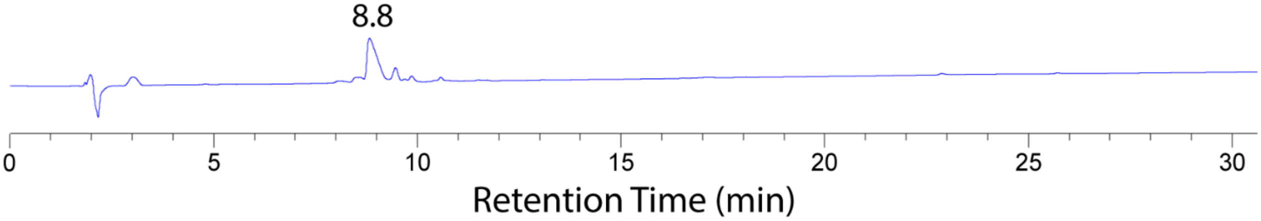

HRMS for FDP Product 2f

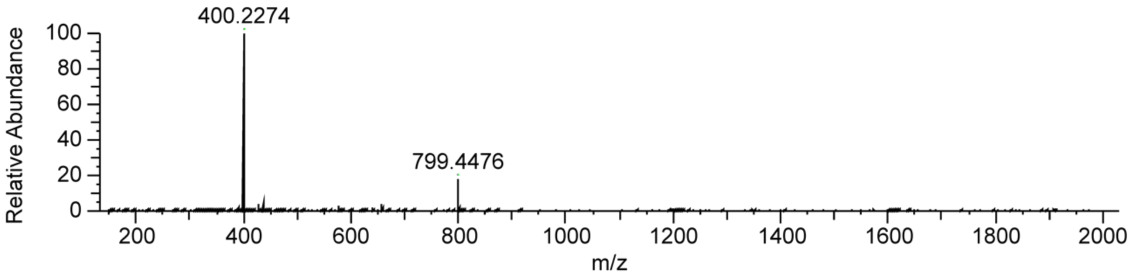

Formation of FDP Product 2g

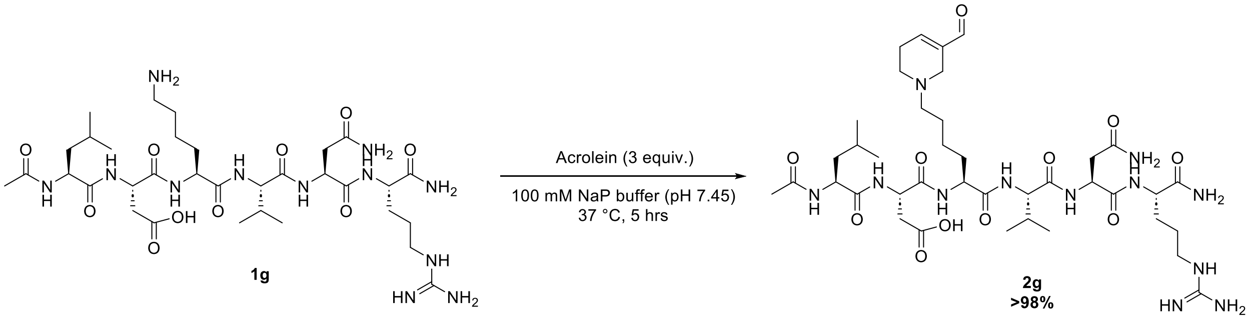

Ac-LDKVNR **1g** (2 mg, 2.5 µmol, 1 equiv.) was dissolved in 580 µL of 100 mM NaP buffer (pH 7.45) in a 1” dram vial. Acrolein (0.51 µL, 7.6 µmol, 3 equiv.) was added to the vial from a freshly prepared stock solution (20 µL). The mixture was stirred at 37 °C for 5 hours, after which the reaction was analyzed using **HPLC Method A**, revealing >98% conversion to the FDP-modified product **2g**.

FDP Peptide 2g. LCMS, m/z 879.5044 (calcd. $[M+H^+]$ = 879.5047), m/z 440.2558 (calcd. $[(M+2H^+)/2]$ = 440.2560), Purity: >99% (HPLC analysis at 220 nm). Retention time in HPLC: 8.0 min.

HPLC Trace for Reaction Forming FDP Product 2g

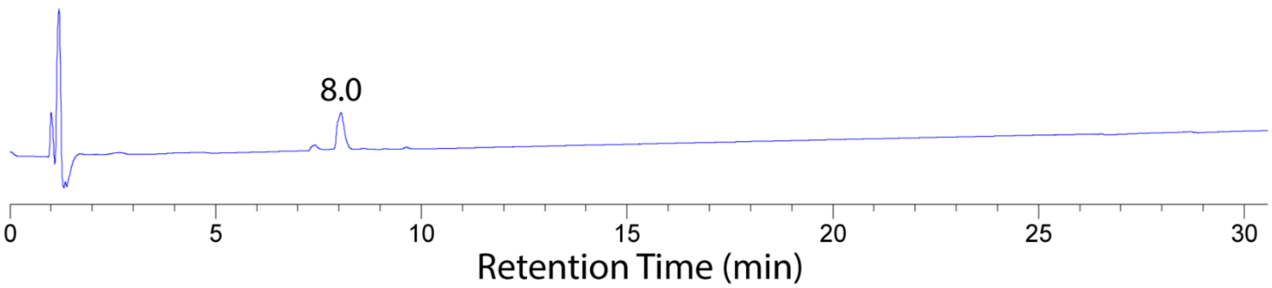

HRMS of FDP Product 2g

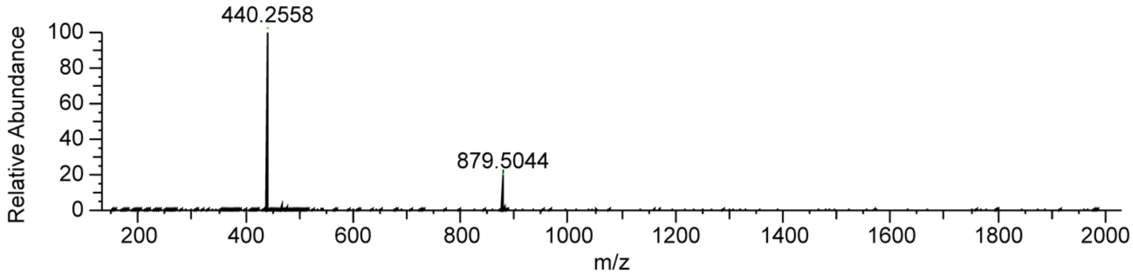

Formation of Doubly Modified FDP Product 2h

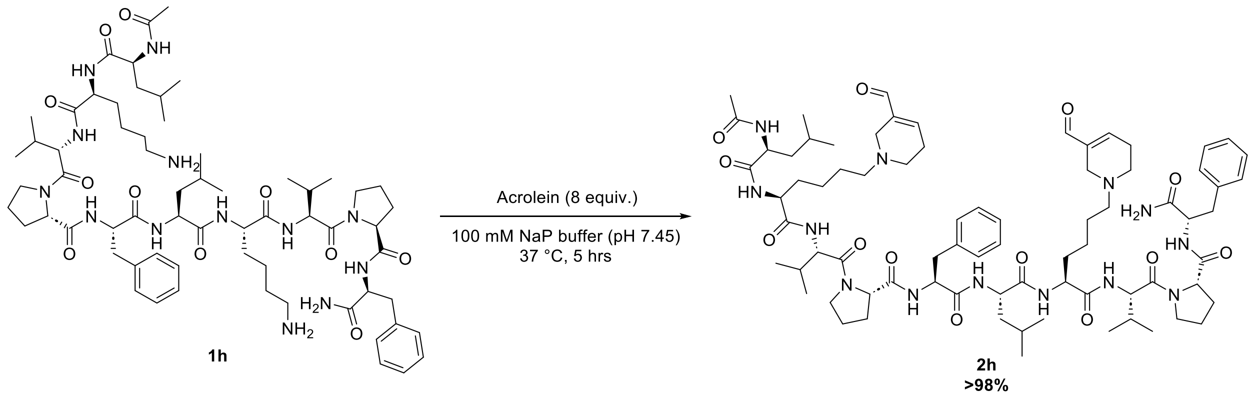

Ac-LKVPFLKVPF **1h** (2 mg, 1.6 µmol, 1 equiv.) was dissolved in 580 µL of 100 mM NaP buffer (pH 7.45) in a 1” dram vial. Acrolein (0.87 µL, 13.0 µmol, 8 equiv.) was added to the vial from a freshly prepared stock solution (20 µL). The mixture was stirred at 37 °C for 5 hours, after which the reaction was analyzed using **HPLC Method A**, revealing >98% conversion to the doubly FDP-modified product **2h**.

FDP Peptide 2h. LCMS, m/z 1416.8650 (calcd. [M+H$^+$] = 1416.8654), m/z 1438.8463 (calcd. [M+Na$^+$] = 1438.8473), m/z 708.9365 (calcd. [(M+2H$^+$)/2] = 708.9363), Purity: >99% (HPLC analysis at 220 nm). Retention time in HPLC: 17.1 min.

HPLC Trace for Reaction Forming Doubly Modified FDP Product 2h

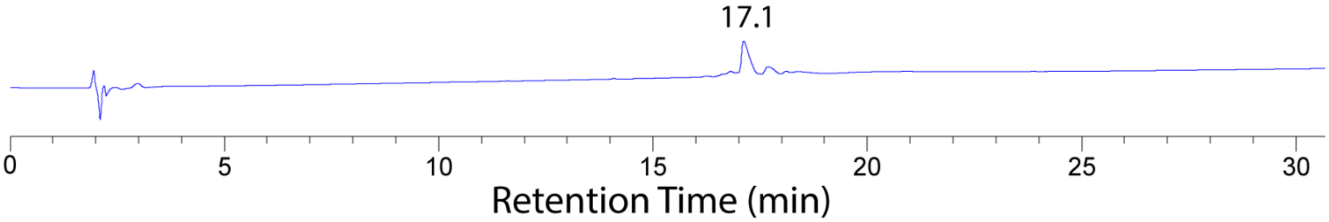

HRMS of Doubly Modified FDP Product 2h

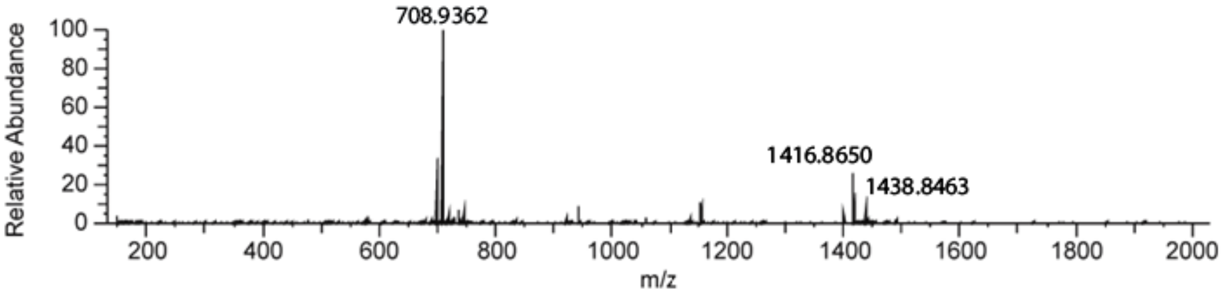

Formation of Singly Modified FDP Product 2h'

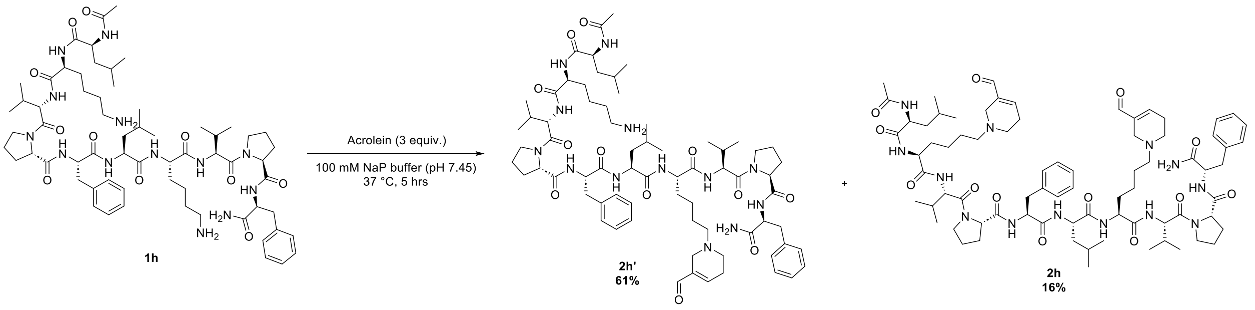

Ac-LKVPFLKVPF **1h** (2 mg, 1.6 µmol, 1 equiv.) was dissolved in 580 µL of 100 mM NaP buffer (pH 7.45) in a 1" dram vial. Acrolein (0.33 µL, 4.9 µmol, 3 equiv.) was added to the vial from a freshly prepared stock solution (20 µL). The mixture was stirred at 37 °C for 5 hours, after which the reaction was analyzed using **HPLC Method A**, revealing 61% conversion to the singly FDP-modified product **2h**', 16% conversion to the doubly FDP-modified product **2h**, and 23% starting peptide **1h**.

Ac-LKVPFLKVPF-CONH$_2$ (1h) peptide. LCMS, m/z 614.8947 (calcd. [(M+2H$^+$)/2] = 614.8945), Purity: >99% (HPLC analysis at 220 nm). Retention time in HPLC: 16.0 min.

FDP Peptide 2h'. LCMS, m/z 1322.8236 (calcd. [M+H$^+$] = 1322.8235), m/z 661.9152 (calcd. [(M+2H$^+$)/2] = 661.9154), Purity: >99% (HPLC analysis at 220 nm). Retention time in HPLC: 16.5 min.

FDP Peptide 2h. LCMS, m/z 708.9365 (calcd. [(M+2H$^+$)/2] = 708.9363), Purity: >99% (HPLC analysis at 220 nm). Retention time in HPLC: 17.1 min.

HPLC Trace for Reaction Forming Singly Modified FDP Product 2h'

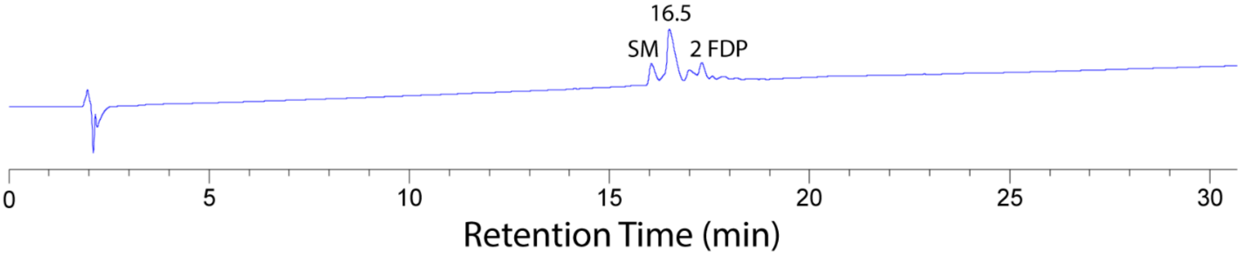

HRMS of Singly Modified FDP Product 2h'

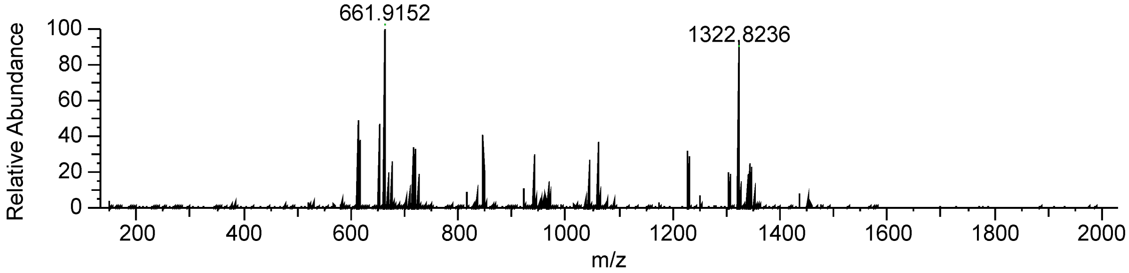

Formation of FDP Product 2i

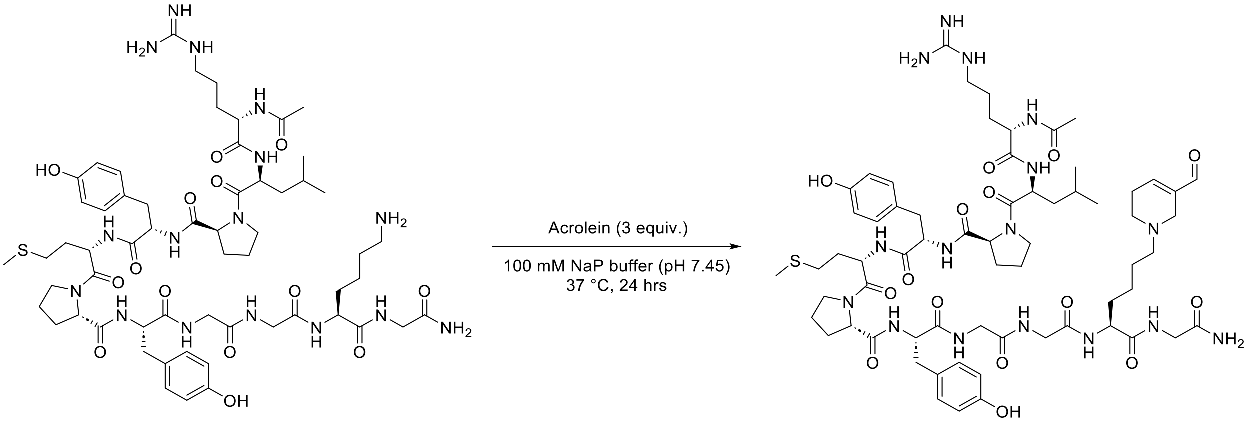

Ac-RLPYMPYGGKG **1i** (2 mg, 1.6 µmol, 1 equiv.) was dissolved in 580 µL of 100 mM NaP buffer (pH 7.45) in a 1" dram vial. Acrolein (0.31 µL, 4.7 µmol, 3 equiv.) was added to the vial from a freshly prepared stock solution (20 µL). The mixture was stirred at 37 °C for 5 hours, after which the reaction was analyzed using **HPLC Method A**, revealing >98% conversion to the FDP-modified product **2i**.

FDP Peptide 2i. LCMS, m/z 1373.7029 (calcd. $[(M+2H^+)/2]$ = 1373.7035), m/z 687.3557 (calcd. $[(M+2H^+)/2]$ = 687.3554), Purity: >99% (HPLC analysis at 220 nm). Retention time in HPLC: 11.8 min.

HPLC Trace for Reaction Forming FDP Product 2i

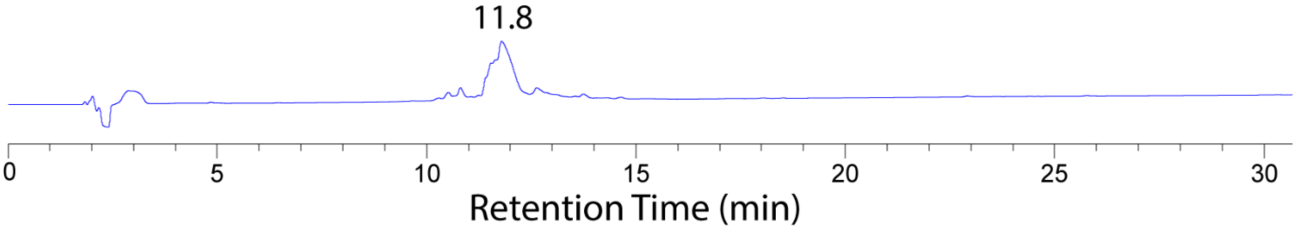

HRMS of FDP Product 2i

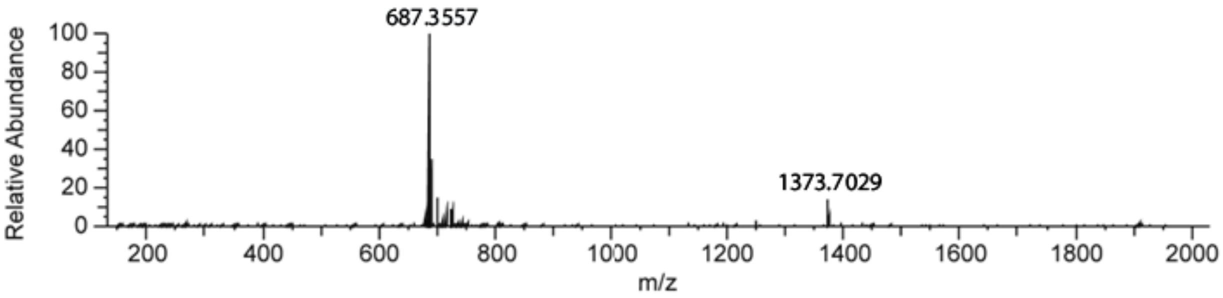

Fig. S14 – Tagging FDP lysine with hydroxylamine

Formation of Oxime Peptide 4a (1.2 Equiv. Hydroxylamine)

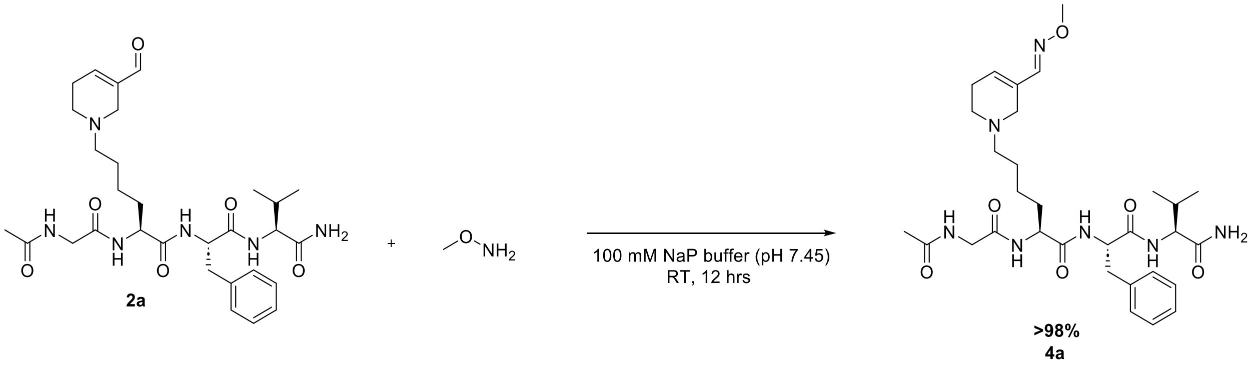

FDP Peptide **2a** (1 mg, 1.7 µmol, 1 equiv.), purified via analytical HPLC, was dissolved in 480 µL of 100 mM NaP buffer (pH 7.45) in a 1” dram vial. O-methylhydroxylamine hydrochloride (0.17 mg, 2.1 µmol, 1.2 equiv.) was added to the vial from a freshly prepared stock solution (20 µL). The mixture was stirred at room temperature for 12 hours, after which the reaction was analyzed using **HPLC Method A**, revealing >98% conversion to homogeneous product **4a** with a single hydroxylamine addition.

Oxime Peptide 4a (Single Hydroxylamine Addition). LCMS, m/z 614.3657 (calcd. $[M+H^+]$ = 614.3661), Purity: >99% (HPLC analysis at 220 nm). Retention time in HPLC: 8.0 min.

HPLC Trace for Reaction Forming Oxime Peptide 4a (1.2 Equiv. Hydroxylamine)

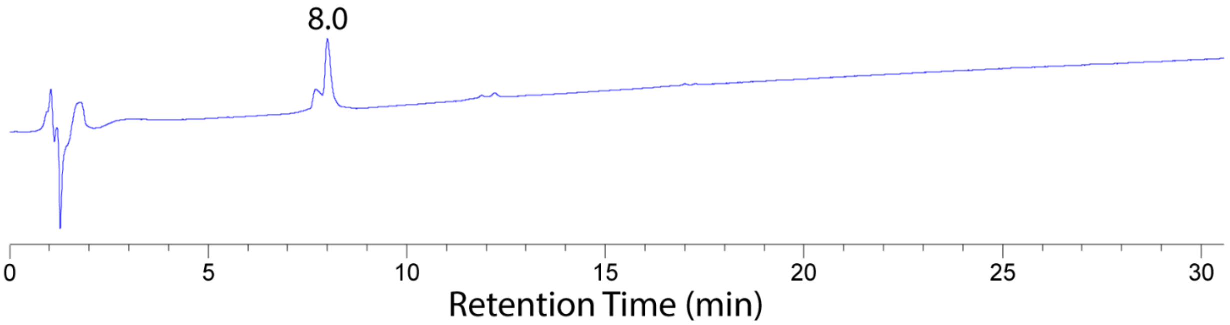

HRMS of Oxime Peptide 4a (Single Hydroxylamine Addition)

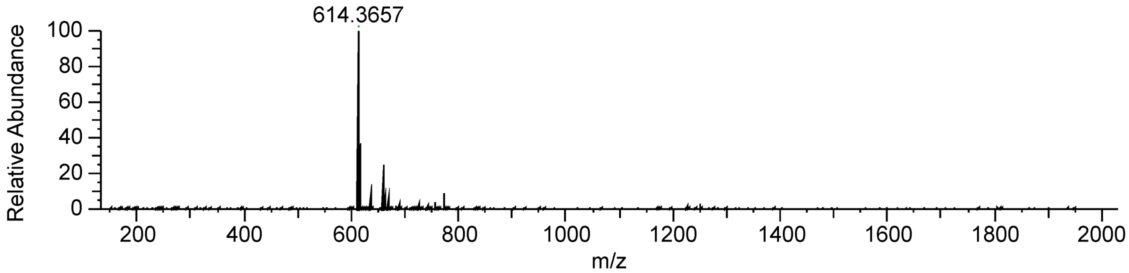

Formation of Oxime Peptide 4a (5 Equiv. Hydroxylamine)

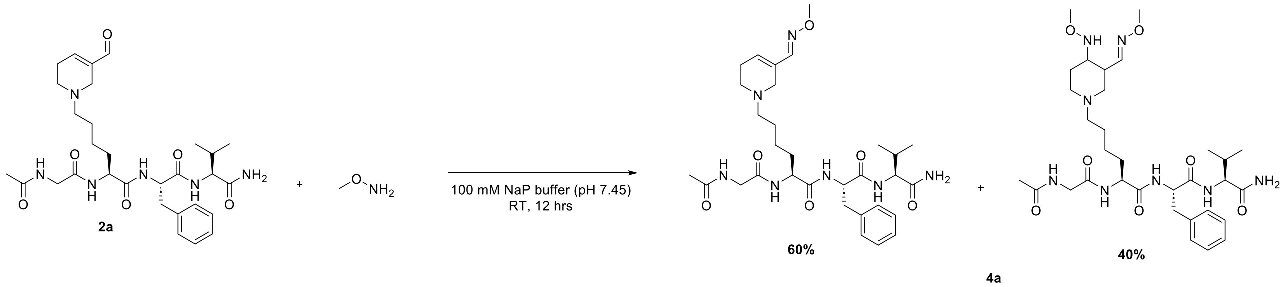

FDP Peptide **2a** (1 mg, 1.7 µmol, 1 equiv.), purified via analytical HPLC, was dissolved in 480 µL of 100 mM NaP buffer (pH 7.45) in a 1” dram vial. O-methylhydroxylamine hydrochloride (0.71 mg, 8.6 µmol, 5 equiv.) was added to the vial from a freshly prepared stock solution (20 µL). The mixture was stirred at room temperature for 12 hours, after which the reaction was analyzed using **HPLC Method A**, revealing >98% conversion to oxime peptide **4a** (60% with a single hydroxylamine addition and 40% with two hydroxylamine additions).

Oxime Peptide 4a (Single Hydroxylamine Addition). LCMS, m/z 614.3657 (calcd. $[M+H^+]$ = 614.3661), Purity: >99% (HPLC analysis at 220 nm). Retention time in HPLC: 9.5 min.

Oxime Peptide 4a (Double Hydroxylamine Addition). LCMS, m/z 661.4026 (calcd. $[M+H^+]$ = 661.4032), Purity: >99% (HPLC analysis at 220 nm). Retention time in HPLC: 9.1 min.

HPLC Trace for Reaction Forming Oxime Peptide 4a (5 Equiv. Hydroxylamine)

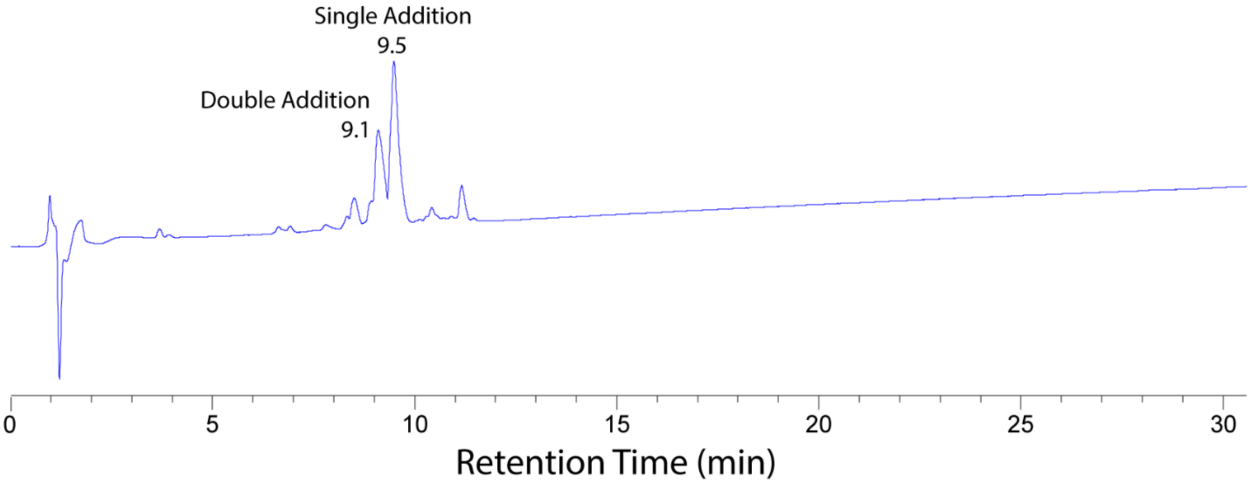

HRMS of Oxime Peptide 4a (Double Hydroxylamine Addition)

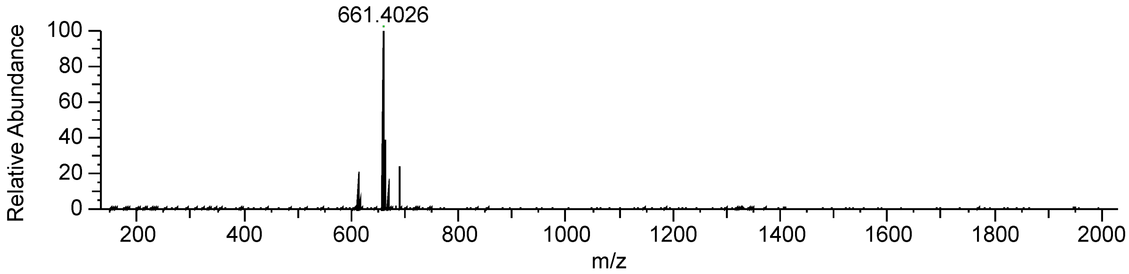

Formation of Oxime Peptide 4f

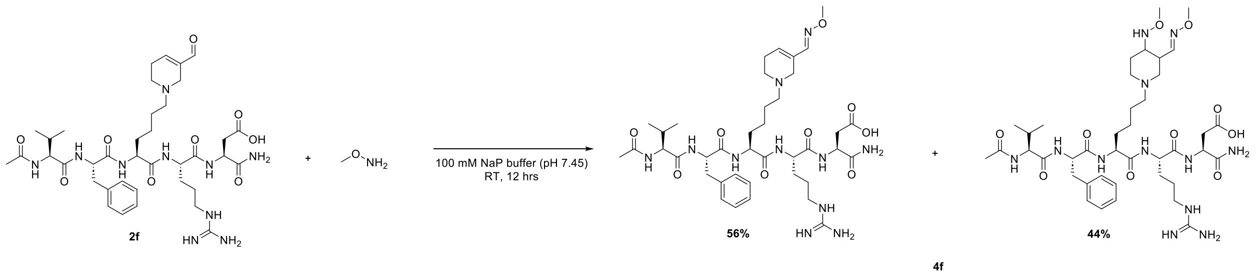

FDP Peptide **4f** (1 mg, 1.3 µmol, 1 equiv.), purified via analytical HPLC, was dissolved in 480 µL of 100 mM NaP buffer (pH 7.45) in a 1” dram vial. O-methylhydroxylamine hydrochloride (0.52 mg, 6.3 µmol, 5 equiv.) was added to the vial from a freshly prepared stock solution (20 µL). The mixture was stirred at room temperature for 12 hours, after which the reaction was analyzed using **HPLC Method A**, revealing >98% conversion to oxime peptide **4f** (56% with a single hydroxylamine addition and 44% with two hydroxylamine additions).

Oxime Peptide 4f (Double Hydroxylamine Addition). LCMS, m/z 875.5092 (calcd. $[M+H^+]$ = 875.5098), m/z 438.2582 (calcd. $[(M+2H^+)/2]$ = 438.2585), >99% (HPLC analysis at 220 nm). Retention time in HPLC: 9.4 min.

Oxime Peptide 4f (Single Hydroxylamine Addition). LCMS, m/z 828.4722 (calcd. $[M+H^+]$ = 828.4726), m/z 414.7397 (calcd. $[(M+2H^+)/2]$ = 414.7400), Purity: >99% (HPLC analysis at 220 nm). Retention time in HPLC: 10.0 min.

HPLC Trace for Reaction Forming Oxime Peptide 4f

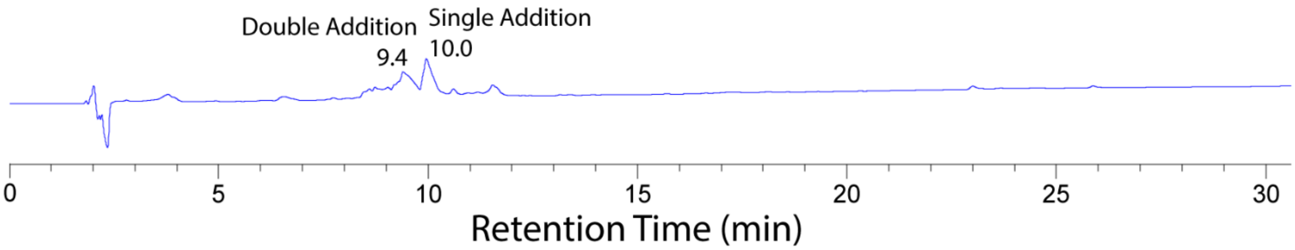

HRMS of Oxime Peptide 4f (Single Hydroxylamine Addition)

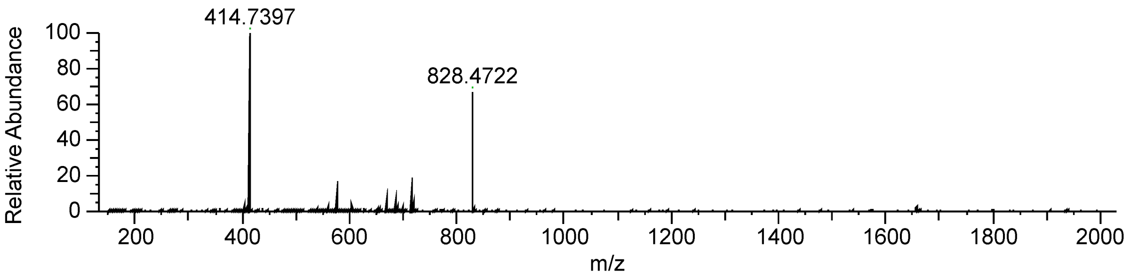

HRMS of Oxime Peptide 4f (Double Hydroxylamine Addition)

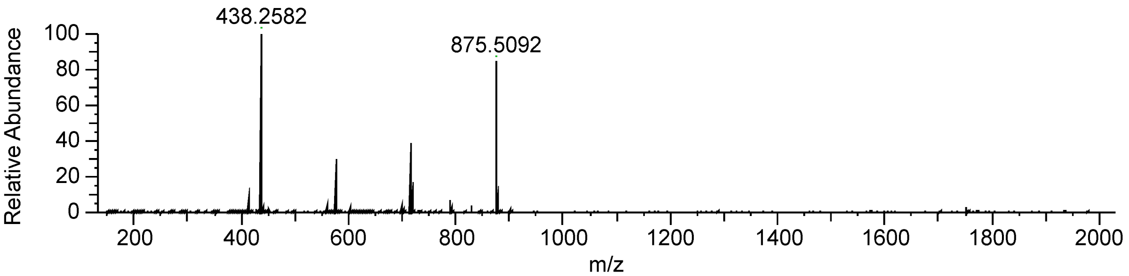

Formation of Oxime Peptide 4g

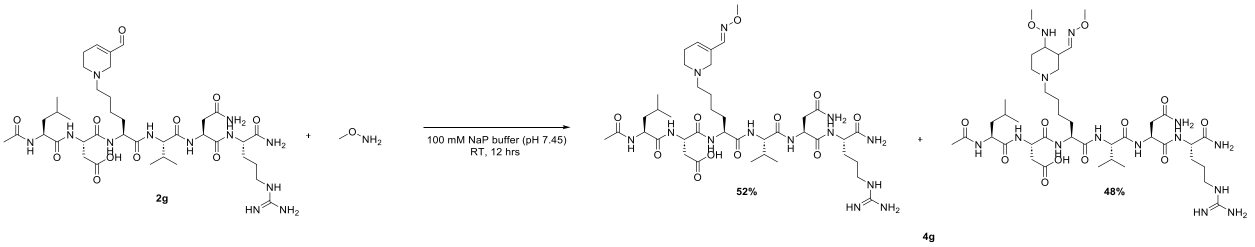

FDP peptide **2g** (1 mg, 1.1 µmol, 1 equiv.), purified via analytical HPLC, was dissolved in 480 µL of 100 mM NaP buffer (pH 7.45) in a 1" dram vial. O-methylhydroxylamine hydrochloride (0.48 mg, 5.7 µmol, 5 equiv.) was added to the vial from a freshly prepared stock solution (20 µL). The mixture was stirred at room temperature for 12 hours, after which the reaction was analyzed using

HPLC Method A, revealing >98% conversion to oxime peptide **4g** (52% with a single hydroxylamine addition and 48% with two hydroxylamine additions).

Oxime Peptide 4g (Double Hydroxylamine Addition). LCMS, m/z 955.5679 (calcd. $[M+H^+]$ = 955.5683), m/z 478.2875 (calcd. $[(M+2H^+)/2]$ = 478.2878), >99% (HPLC analysis at 220 nm). Retention time in HPLC: 10.2 min.

Oxime Peptide 4g (Single Hydroxylamine Addition). LCMS, m/z 908.5313 (calcd. $[M+H^+]$ = 908.5312), m/z 454.7693 (calcd. $[(M+2H^+)/2]$ = 454.7693), Purity: >99% (HPLC analysis at 220 nm). Retention time in HPLC: 10.6 min.

HPLC Trace for Reaction Forming Oxime Peptide 4g

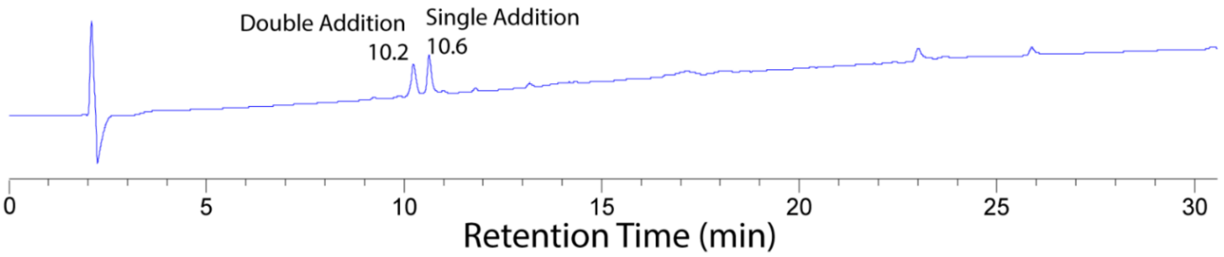

HRMS of Oxime Peptide 4g (Single Hydroxylamine Addition)

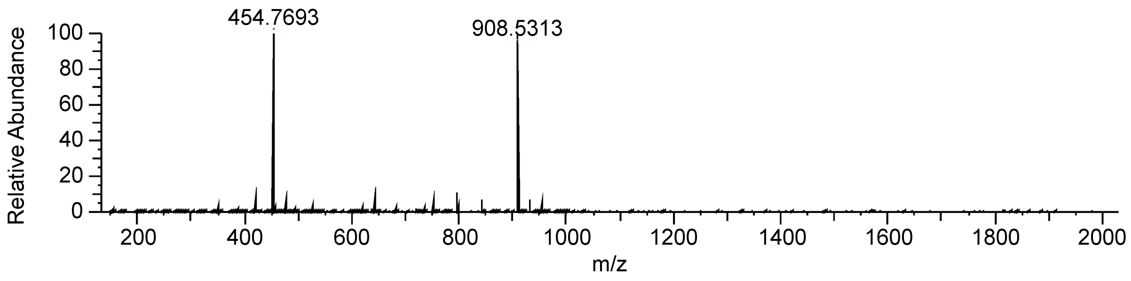

HRMS of Oxime Peptide 4g (Double Hydroxylamine Addition)

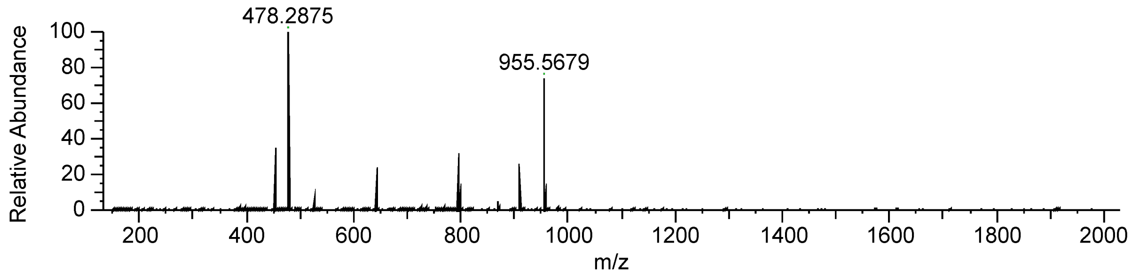

Formation of Oxime Peptide 4h (from Doubly Tagged 2h)

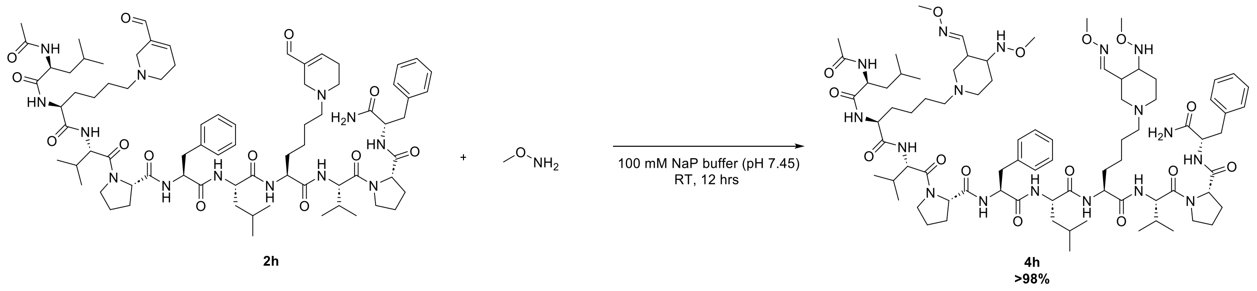

FDP peptide **2h** (1 mg, 0.71 µmol, 1 equiv.), purified via analytical HPLC, was dissolved in 480 µL of 100 mM NaP buffer (pH 7.45) in a 1” dram vial. O-methylhydroxylamine hydrochloride (0.29 mg, 3.5 µmol, 5 equiv.) was added to the vial from a freshly prepared stock solution (20 µL). The mixture was stirred at room temperature for 12 hours, after which the reaction was analyzed using **HPLC Method A**, revealing >98% conversion to homogeneous oxime peptide **4h** with two hydroxylamine additions to both FDP lysines.

Oxime Peptide 4h (Double Hydroxylamine Addition to Both FDP Lysines). LCMS, m/z 1568.9912 (calcd. $[M+H^+]$ = 1568.9927), m/z 785.0000 (calcd. $[(M+2H^+)/2]$ = 785.0000), > 99 % (HPLC analysis at 220 nm). Retention time in HPLC: 17.7 min.

HPLC Trace for Reaction Forming Oxime Peptide 4h

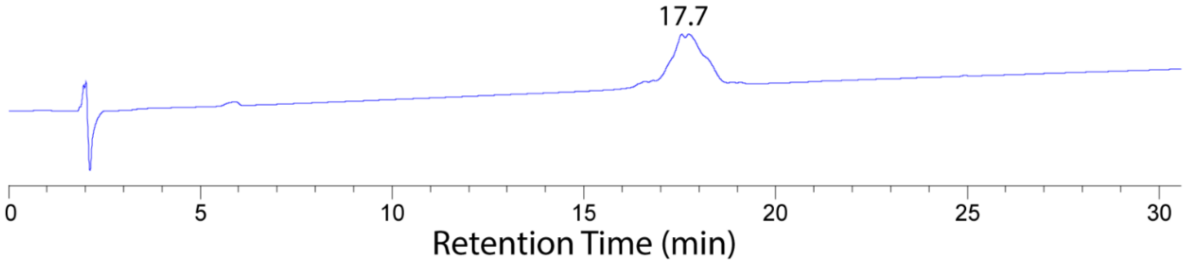

HRMS of Oxime Peptide 4h (Double Hydroxylamine Addition to Both FDP Lysines)

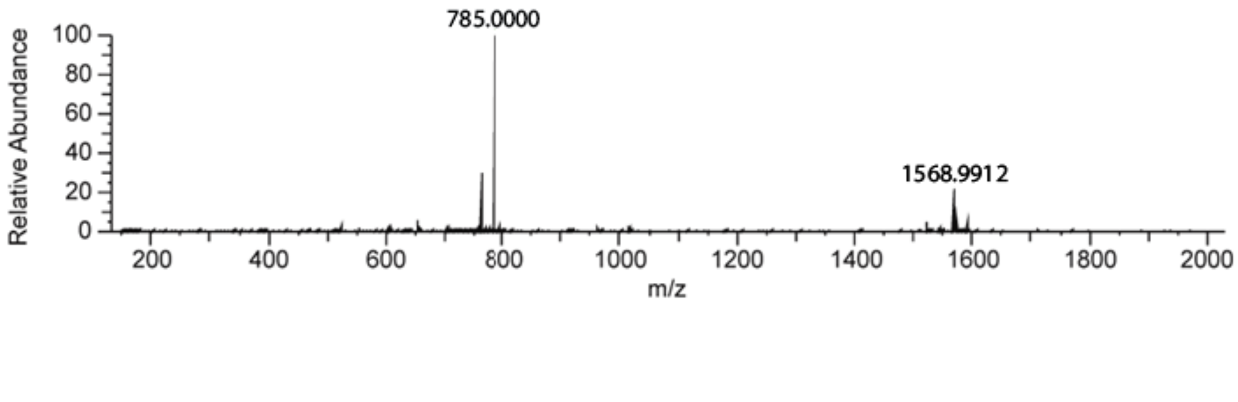

Formation of Oxime Peptide 4h' (from Singly Tagged 2h')

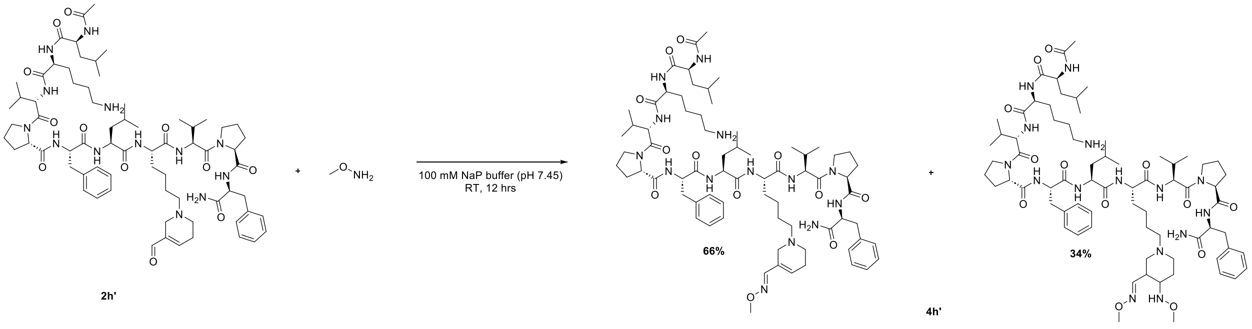

FDP peptide **2h'** (1 mg, 0.76 µmol, 1 equiv.), purified via analytical HPLC, was dissolved in 480 µL of 100 mM NaP buffer (pH 7.45) in a 1" dram vial. O-methylhydroxylamine hydrochloride (0.32 mg, 3.8 µmol, 5 equiv.) was added to the vial from a freshly prepared stock solution (20 µL). The mixture was stirred at room temperature for 12 hours, after which the reaction was analyzed using **HPLC Method A**, revealing >98% conversion to oxime peptide **4h'** (66% with a single hydroxylamine addition and 34% with two hydroxylamine additions).

Oxime Peptide 4h' (Double Hydroxylamine Addition). LCMS, m/z 1398.8867 (calcd. $[M+H^+]$ = 1398.8872), m/z 1420.8685 (calcd. $[M+Na^+]$ = 1420.8691), m/z 699.9473 (calcd. $[(M+2H^+)/2]$ = 699.9472), >99% (HPLC analysis at 220 nm). Retention time in HPLC: 16.8 min.

Oxime Peptide 4h' (Single Hydroxylamine Addition). LCMS, m/z 1351.8495 (calcd. $[M+H^+]$ = 1351.8500), m/z 1373.8307 (calcd. $[M+Na^+]$ = 1373.8320), m/z 676.4285 (calcd. $[(M+2H^+)/2]$ = 676.4287), Purity: >99% (HPLC analysis at 220 nm). Retention time in HPLC: 17.0 min.

HPLC Trace for Reaction Forming Oxime Peptide 4h'

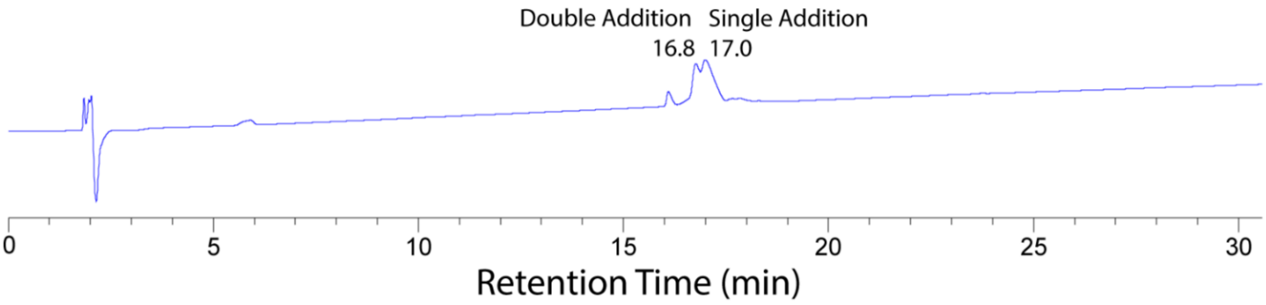

HRMS of Oxime Peptide 4h' (Single Hydroxylamine Addition)

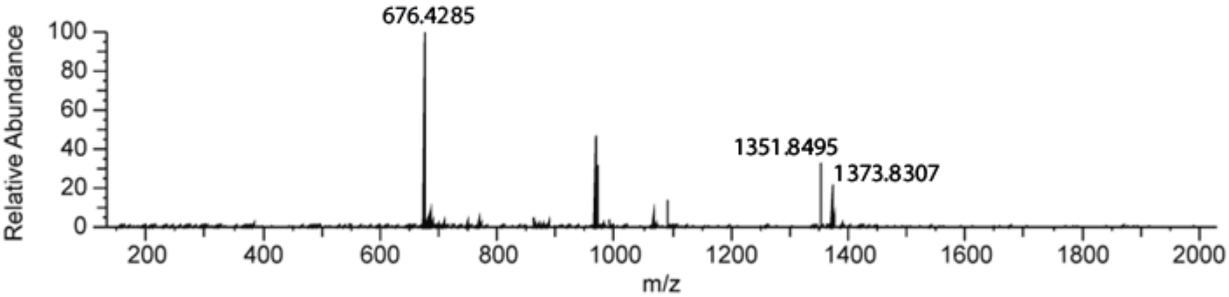

HRMS of Oxime Peptide 4h' (Double Hydroxylamine Addition)

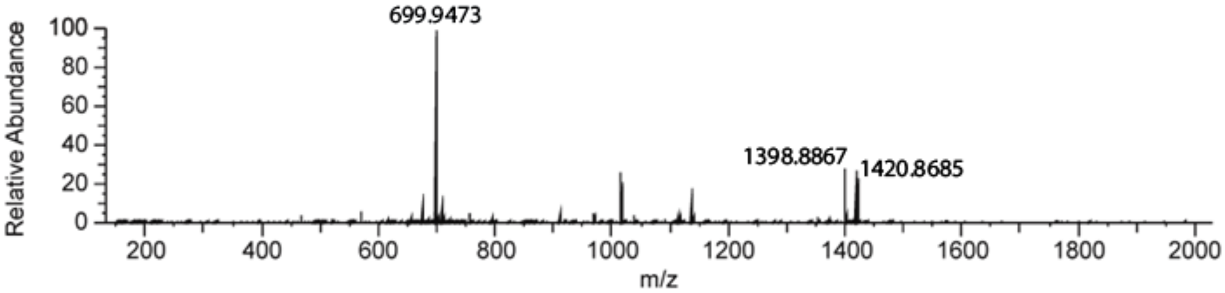

Formation of Oxime Peptide 4i

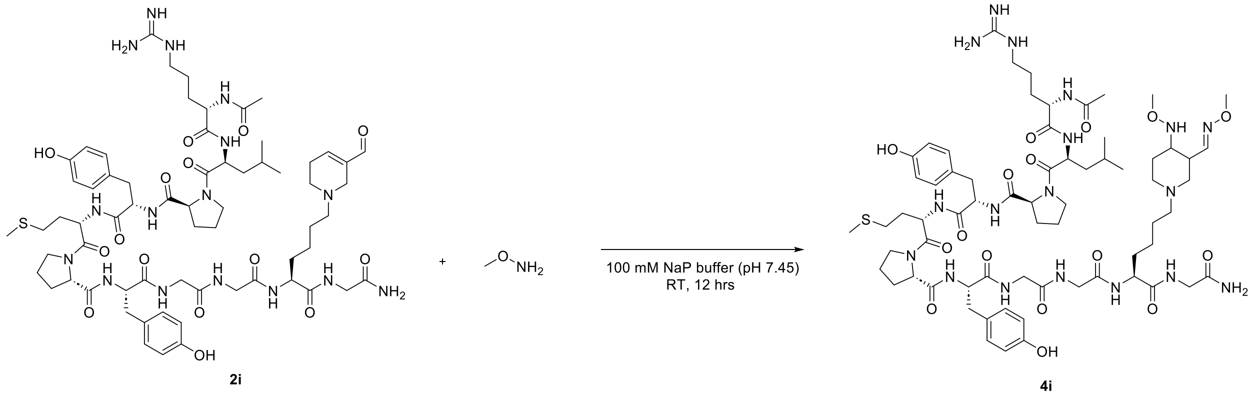

FDP peptide **2i** (1 mg, 0.73 µmol, 1 equiv.), purified via analytical HPLC, was dissolved in 480 µL of 100 mM NaP buffer (pH 7.45) in a 1" dram vial. O-methylhydroxylamine hydrochloride (0.30 mg, 3.6 µmol, 5 equiv.) was added to the vial from a freshly prepared stock solution (20 µL). The mixture was stirred at room temperature for 12 hours, after which the reaction was analyzed using **HPLC Method A**, revealing >98% conversion to homogeneous oxime peptide **4i** with two hydroxylamine additions.

Oxime Peptide 4i (Double Hydroxylamine Addition). LCMS, m/z 1449.7666 (calcd. [M+H$^+$] = 1449.7671), m/z 725.3873 (calcd. [(M+2H$^+$)/2] = 725.3872), >99% (HPLC analysis at 220 nm). Retention time in HPLC: 9.1 min.

HPLC Trace for Reaction Forming Oxime Peptide 4i

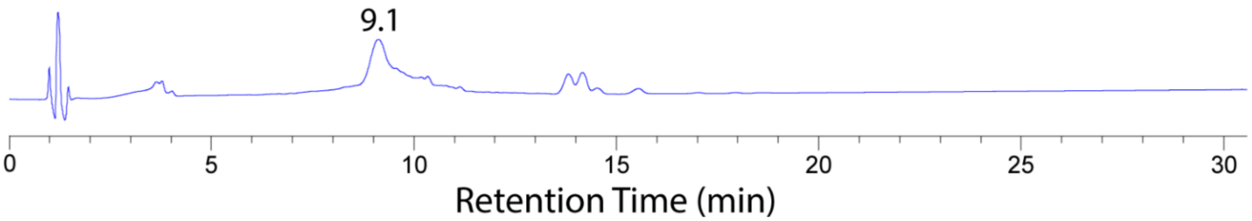

HRMS of Oxime Peptide 4i (Double Hydroxylamine Addition)

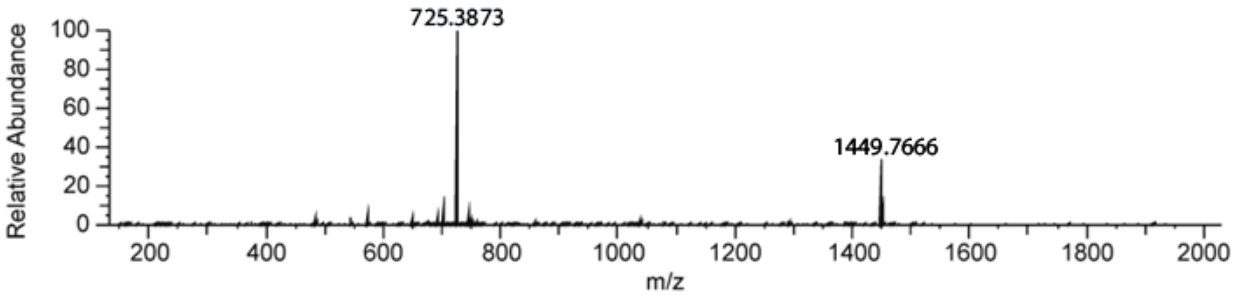

Fig. S15 – Formation of MP lysine on bioactive peptides 1f-1i

Formation of MP Product 3f

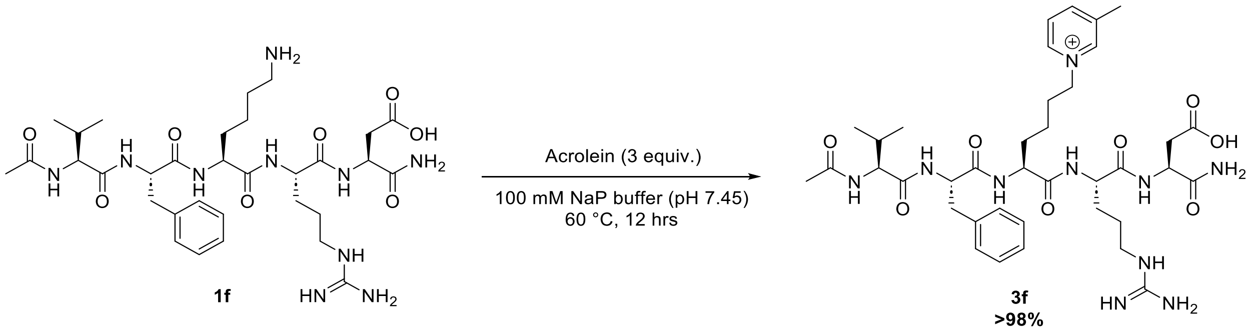

Ac-VFKRD **1f** (2 mg, 2.8 µmol, 1 equiv.) was dissolved in 580 µL of 100 mM NaP buffer (pH 7.45) in a 1” dram vial. Acrolein (0.57 µL, 8.5 µmol, 3 equiv.) was added to the vial from a freshly prepared stock solution (20 µL). The mixture was stirred at 60 °C for 12 hours, after which the reaction was analyzed using **HPLC Method A**, revealing >98% conversion to the MP-modified product **3f**.

MP Peptide 3f. LCMS, m/z 781.4373 (calcd. [M] = 781.4355), Purity: >99% (HPLC analysis at 220 nm). Retention time in HPLC: 10.7 min.

HPLC Trace for Reaction Forming MP Product 3f

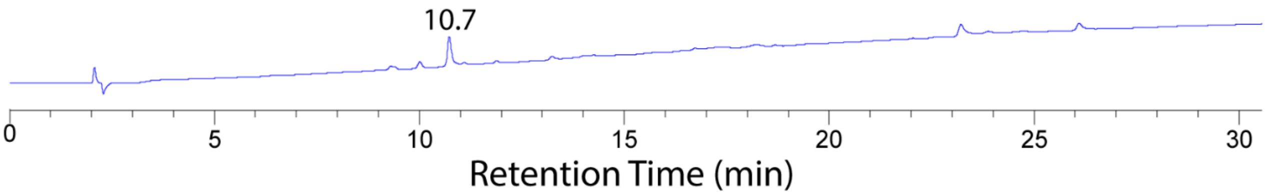

HRMS of MP Product 3f

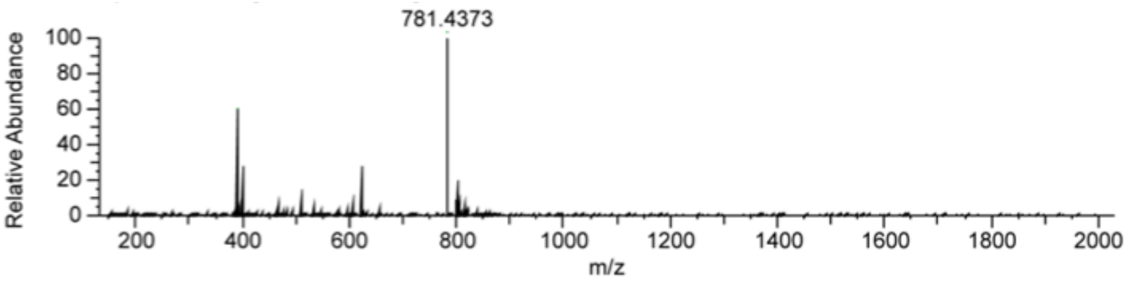

Formation of MP Product 3g

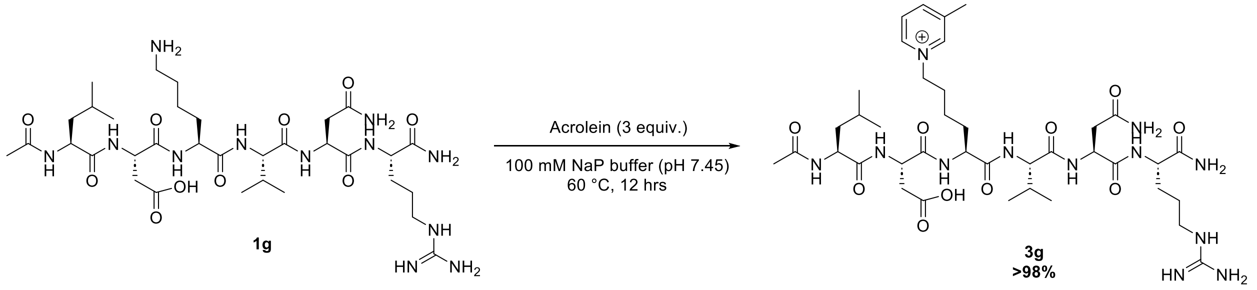

Ac-LDKVNR **1g** (2 mg, 2.5 µmol, 1 equiv.) was dissolved in 580 µL of 100 mM NaP buffer (pH 7.45) in a 1" dram vial. Acrolein (0.51 µL, 7.6 µmol, 3 equiv.) was added to the vial from a freshly prepared stock solution (20 µL). The mixture was stirred at 60 °C for 12 hours, after which the reaction was analyzed using **HPLC Method A**, revealing >98% conversion to the MP-modified product **3g**.

MP Peptide 3g. LCMS, m/z 861.4939 (calcd. [M] = 869.4941), m/z 431.2505 (calcd. [(M+H$^+$)/2] = 431.2507), Purity: >99% (HPLC analysis at 220 nm). Retention time in HPLC: 9.0 min.

HPLC Trace for Reaction Forming MP Product 3g

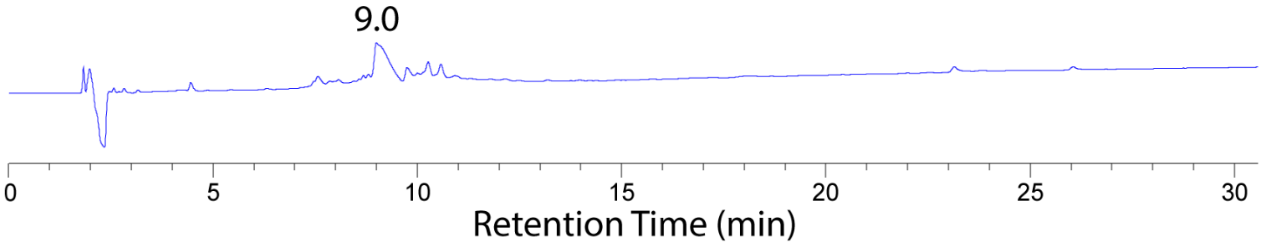

HRMS of MP Product 3g

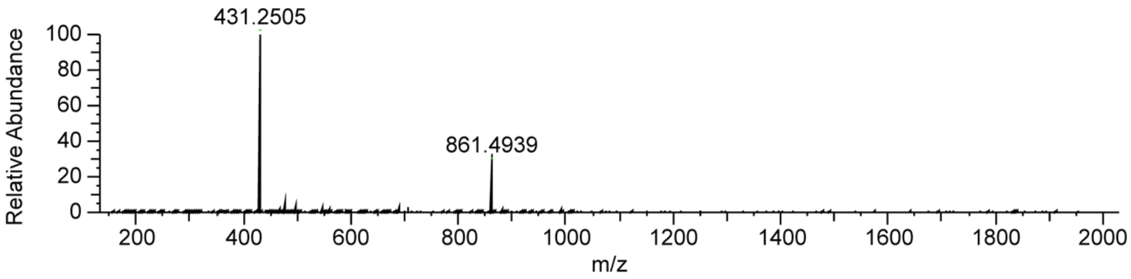

Formation of MP Product 3h (Doubly Modified)

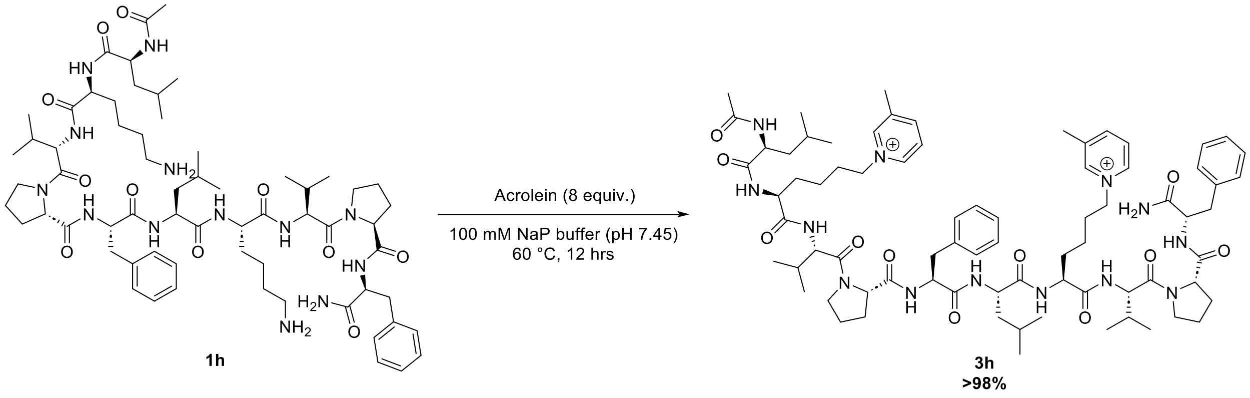

Ac-LKVPFLKVPF **1h** (2 mg, 1.6 µmol, 1 equiv.) was dissolved in 580 µL of 100 mM NaP buffer (pH 7.45) in a 1" dram vial. Acrolein (0.87 µL, 13.0 µmol, 8 equiv.) was added to the vial from a freshly prepared stock solution (20 µL). The mixture was stirred at 60 °C for 12 hours, after which the reaction was analyzed using **HPLC Method A**, revealing >98% conversion to the doubly MP-modified product **3h**.

MP Peptide 3h. LCMS, m/z 690.9258 (calcd. [M/2] = 690.9258), Purity: >99% (HPLC analysis at 220 nm). Retention time in HPLC: 18.3 min.

HPLC Trace for Reaction Forming MP Product 3h (Doubly Modified)

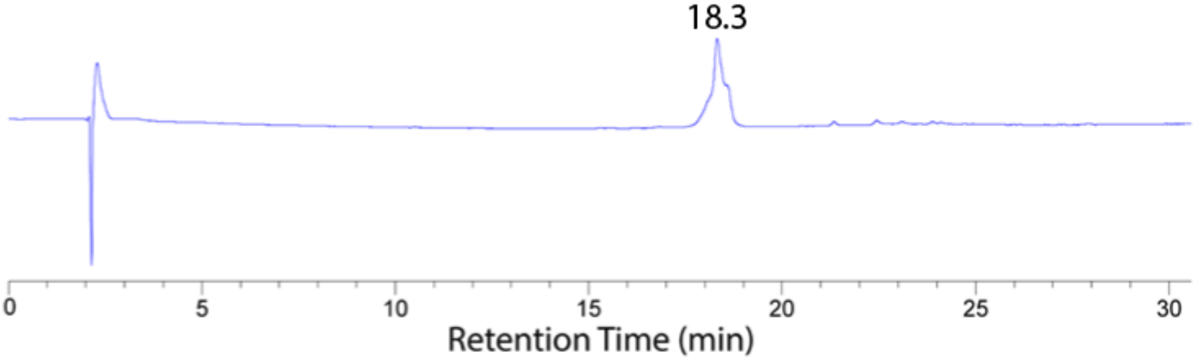

HRMS of MP Product 3h (Doubly Modified)

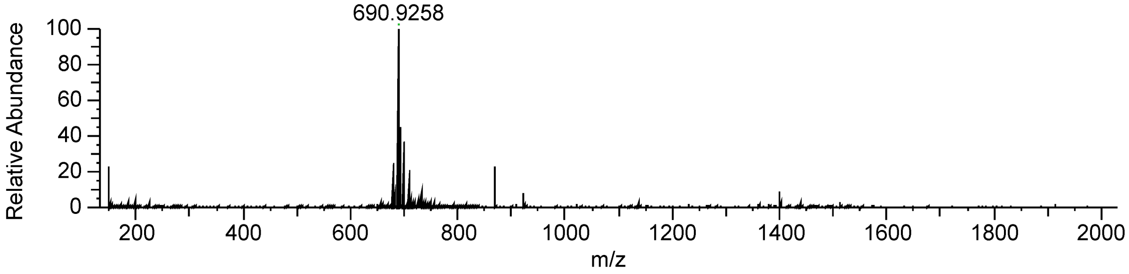

Formation of MP Product 3h' (Singly Modified)

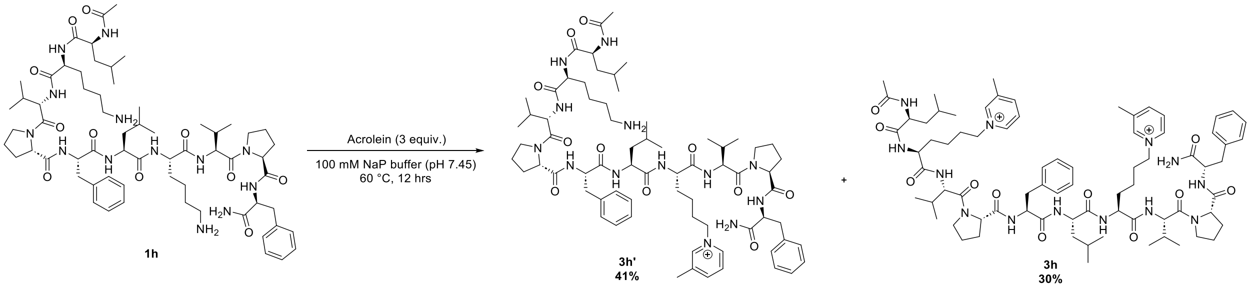

Ac-LKVPFLKVPF **1h** (2 mg, 1.6 µmol, 1 equiv.) was dissolved in 580 µL of 100 mM NaP buffer (pH 7.45) in a 1" dram vial. Acrolein (0.33 µL, 4.9 µmol, 3 equiv.) was added to the vial from a freshly prepared stock solution (20 µL). The mixture was stirred at 60 °C for 12 hours, after which the reaction was analyzed using **HPLC Method A**, revealing 41% conversion to the singly MP-modified product **3h**', 30% conversion to the doubly MP-modified product **3h**, and 29% starting peptide **1h**.

Ac-LKVPFLKVPF-CONH$_2$ (1h) peptide. LCMS, m/z 614.8947 (calcd. [(M+2H$^+$)/2] = 614.8945), Purity: >99% (HPLC analysis at 220 nm). Retention time in HPLC: 15.8 min.

MP Peptide 3h'. LCMS, m/z 1304.8129 (calcd. [M] = 1304.8129), m/z 652.9103 (calcd. [(M+H$^+$)/2] = 652.9101), Purity: >99% (HPLC analysis at 220 nm). Retention time in HPLC: 16.3 min.

MP Peptide 3h. LCMS, m/z 690.9258 (calcd. [M/2] = 690.9258), Purity: >99% (HPLC analysis at 220 nm). Retention time in HPLC: 16.8 min.

HPLC Trace for Reaction Forming MP Product 3h' (Singly Modified)

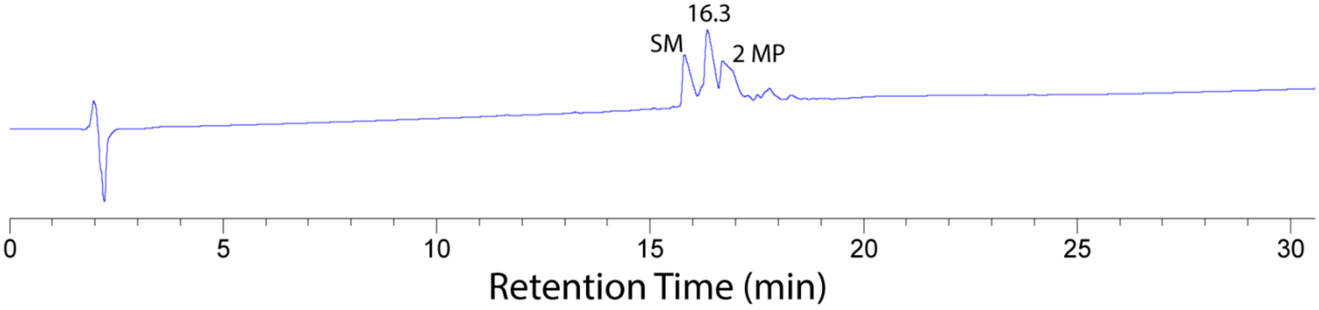

HRMS of MP Product 3h' (Singly Modified)

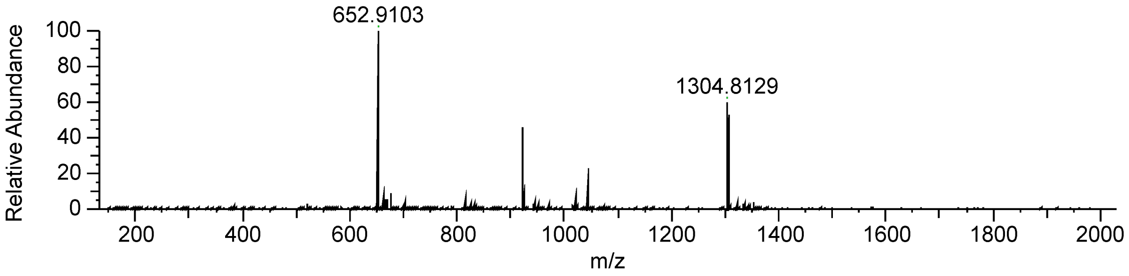

Formation of MP Product 3i

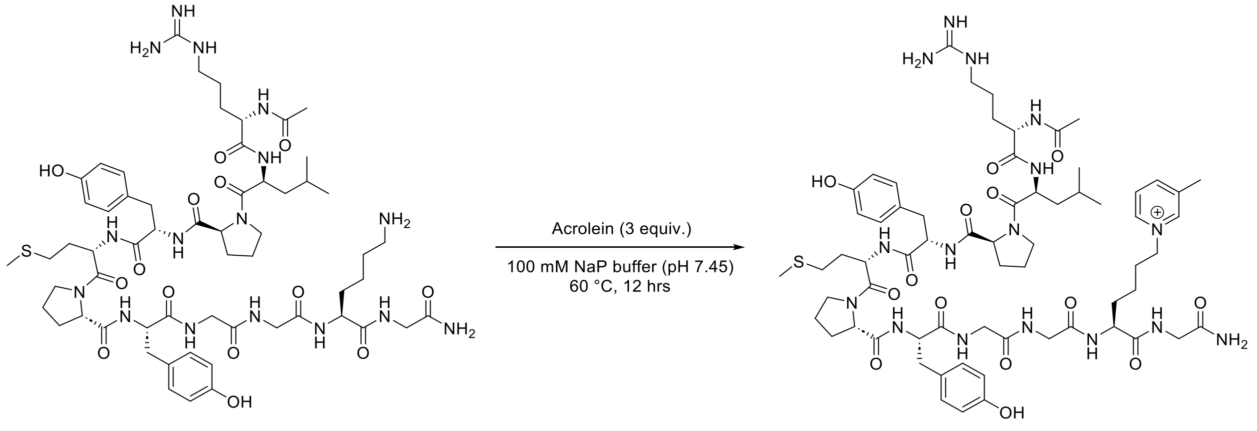

1i

2i
>98%

Ac-RLPYMPYGGKG **1i** (2 mg, 1.6 µmol, 1 equiv.) was dissolved in 580 µL of 100 mM NaP buffer (pH 7.45) in a 1" dram vial. Acrolein (0.31 µL, 4.7 µmol, 3 equiv.) was added to the vial from a freshly prepared stock solution (20 µL). The mixture was stirred at 60 °C for 12 hours, after which the reaction was analyzed using **HPLC Method A**, revealing >98% conversion to the MP-modified product **3i**.

MP Peptide 3i. LCMS, m/z 1355.6915 (calcd. [M] = 1355.6929), m/z 678.3499 (calcd. $[(M+H^+)/2]$ = 678.3501), Purity: >99% (HPLC analysis at 220 nm). Retention time in HPLC: 11.0 min.

HPLC Trace for Reaction Forming MP Product 3i

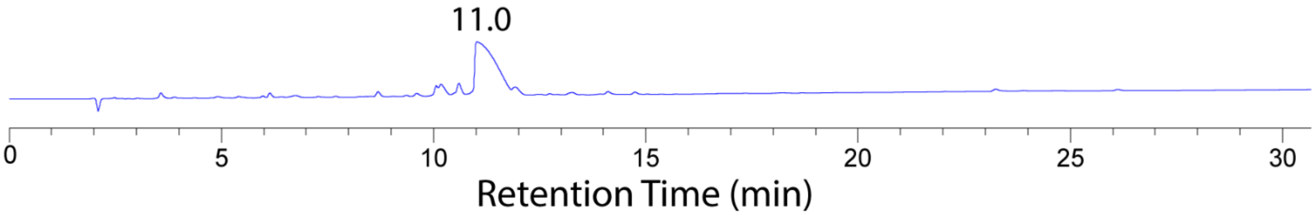

HRMS of MP Product 3i

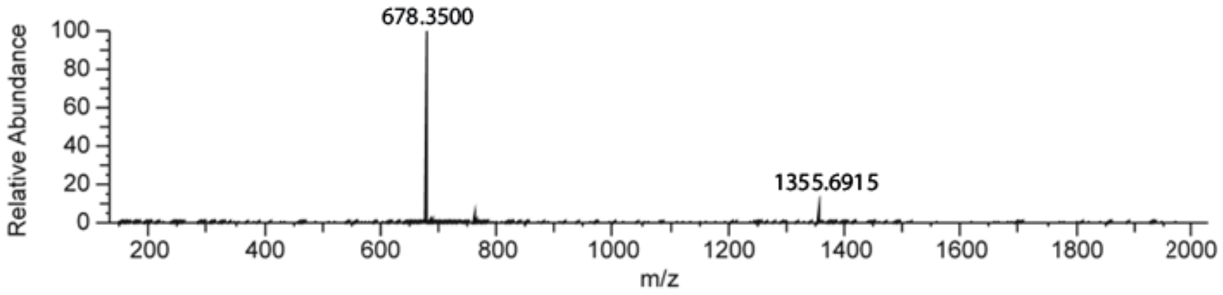

Fig. S16 – Homogeneous labeling of proteins

Optimization of Homogeneous Modification of Myoglobin

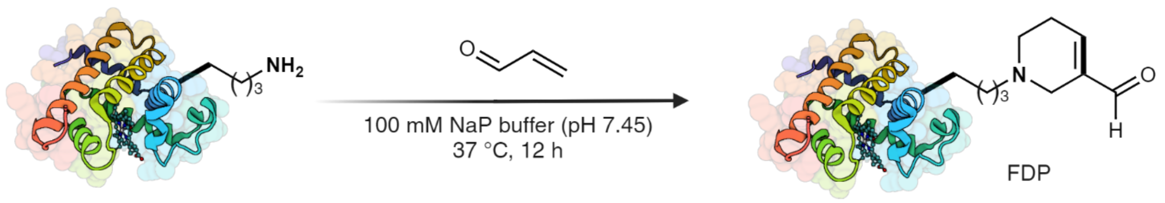

Myoglobin (2 mg, 0.235 mM) was dissolved in 500 µL of 100 mM NaP buffer (pH 7.45), and the designated amount of acrolein (see table below) was added to the mixture. The reaction was stirred at 37 °C for 12 hours, after which the crude reaction mixture was passed through Amicon Ultra 3 kDa spin-concentrator and washed with H_2O (5 x 500 µL) to remove the small molecule impurities. The labeled protein was redissolved in 0.1% formic acid in H_2O and analyzed using LC-MS. See conversion for each attempted condition in the table below. 8 equivalents of acrolein was found to be the optimized condition for obtaining homogeneous singly modified myoglobin.

Entry	Equivalents of Acrolein	Conversion				
		Unmodified	1 FDP	2 FDP	3 FDP	Overall
1	**3 equiv.** (0.024 µL, 0.708 mM)	68%	32%	N/A	N/A	32%
2	**8 equiv.** (0.063 µL,1.19 mM)	N/A	**69%**	31%	N/A	>95%
3	**10 equiv.** (0.079 µL, 2.36 mM)	N/A	55%	45%	N/A	>95%
4	**15 equiv.** (0.118 µL,3.54 mM)	N/A	32%	37%	31%	>95%

Intact MS Spectrum of Starting Myoglobin

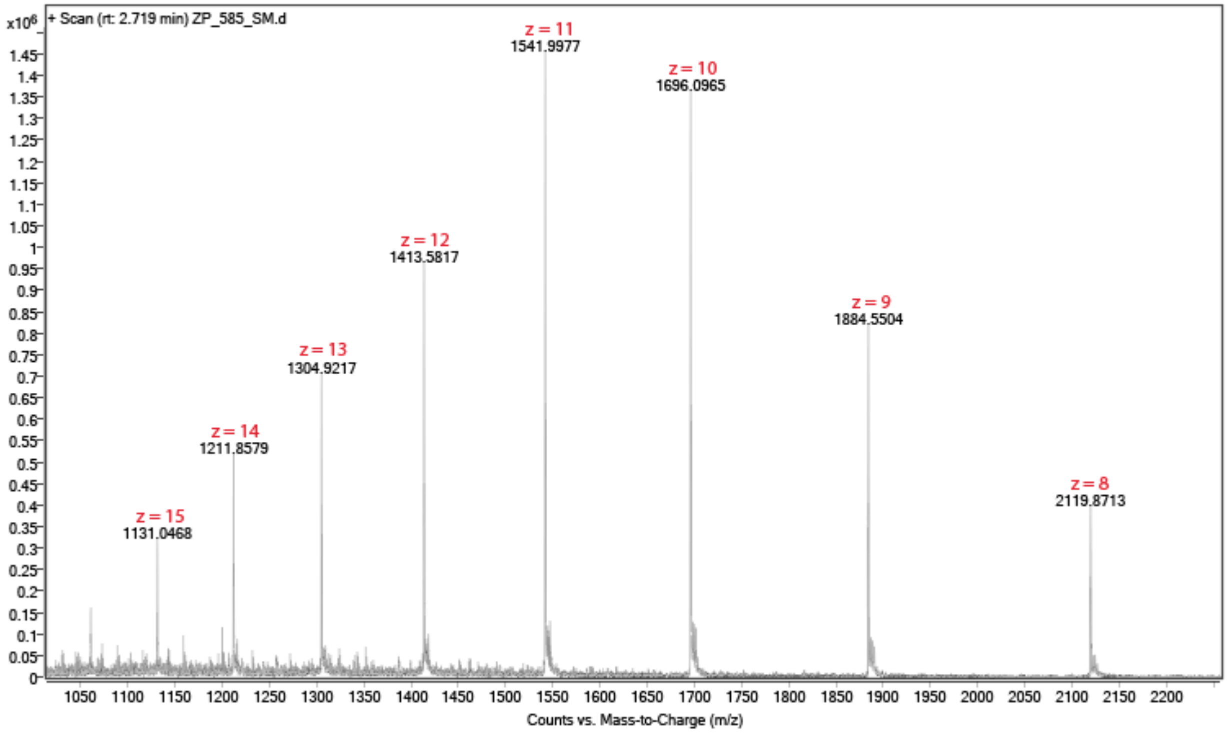

Deconvoluted MS Spectrum of Starting Myoglobin

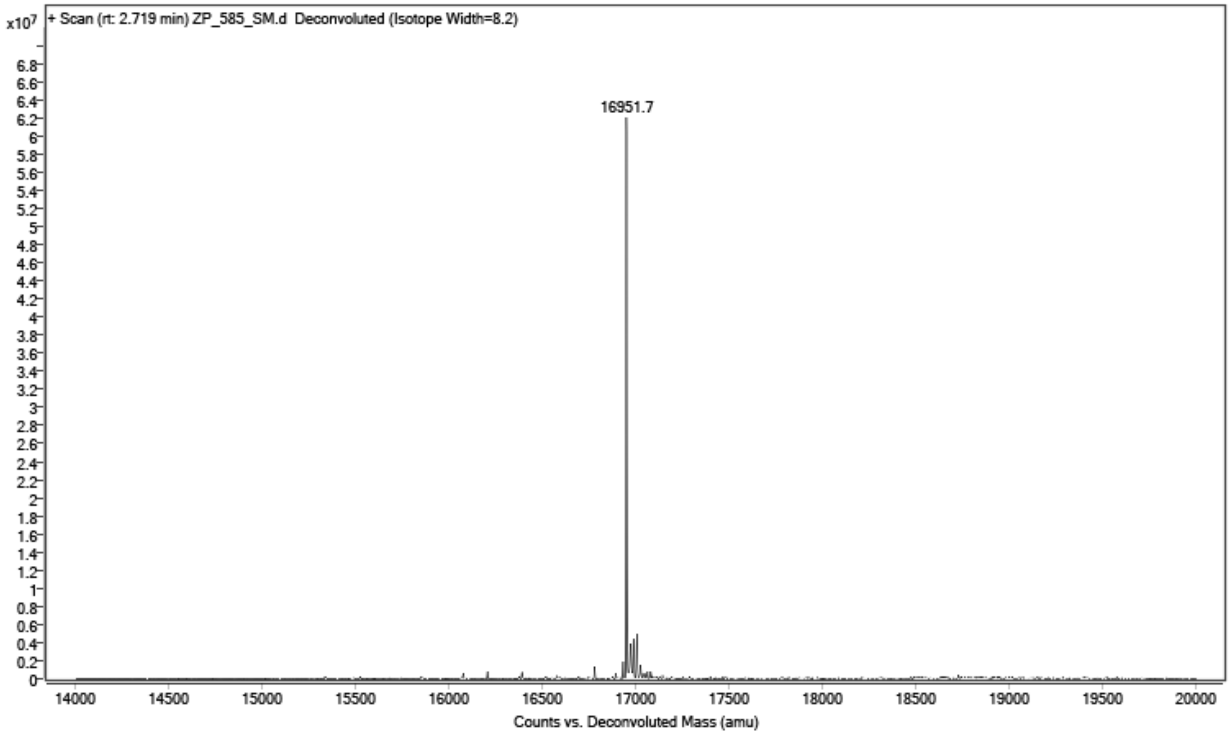

Optimized Conditions for Homogeneous 1 Myoglobin Modification (8 Equiv. Acrolein)

Modification	Mass	Conversion
Unmodified	16951.7	N/A
1 FDP	17045.5 (**+94**)	69%
2 FDP	17139.7 (**+94**)	31%

Intact MS Spectrum of Modified Myoglobin (8 Equiv. Acrolein)

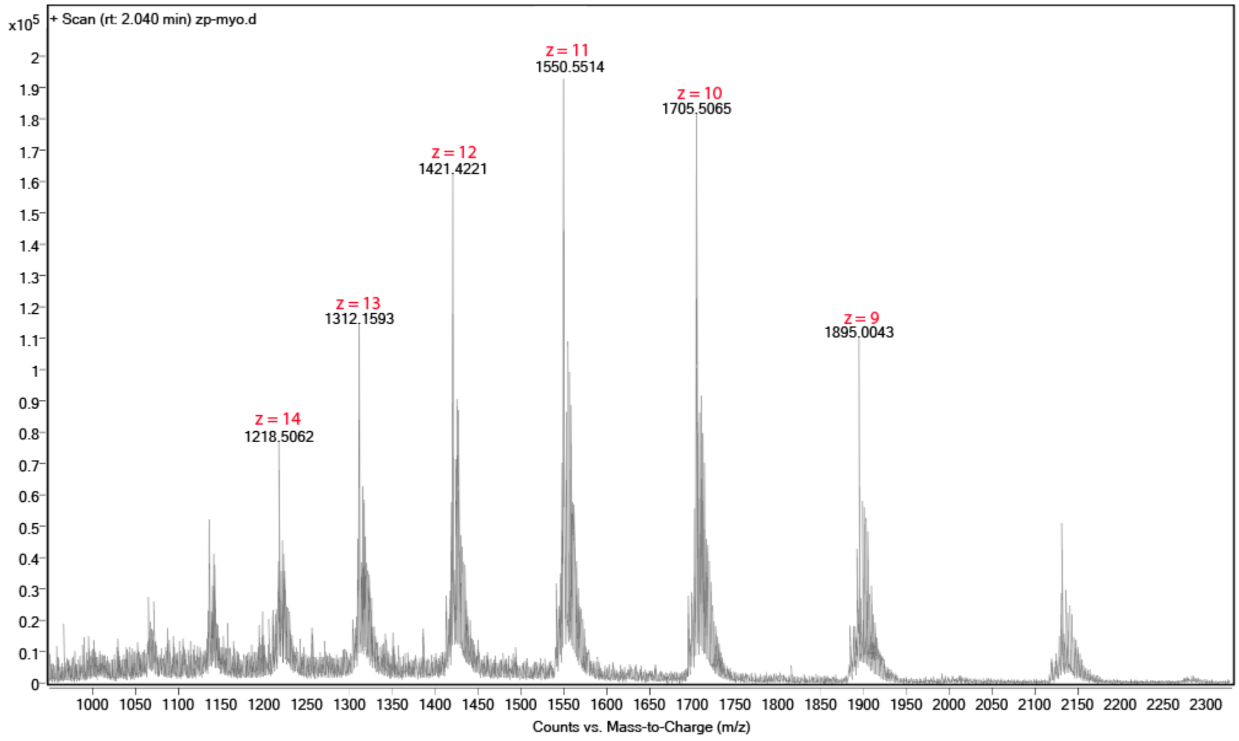

Deconvoluted MS Spectrum of Modified Myoglobin (8 Equiv. Acrolein)

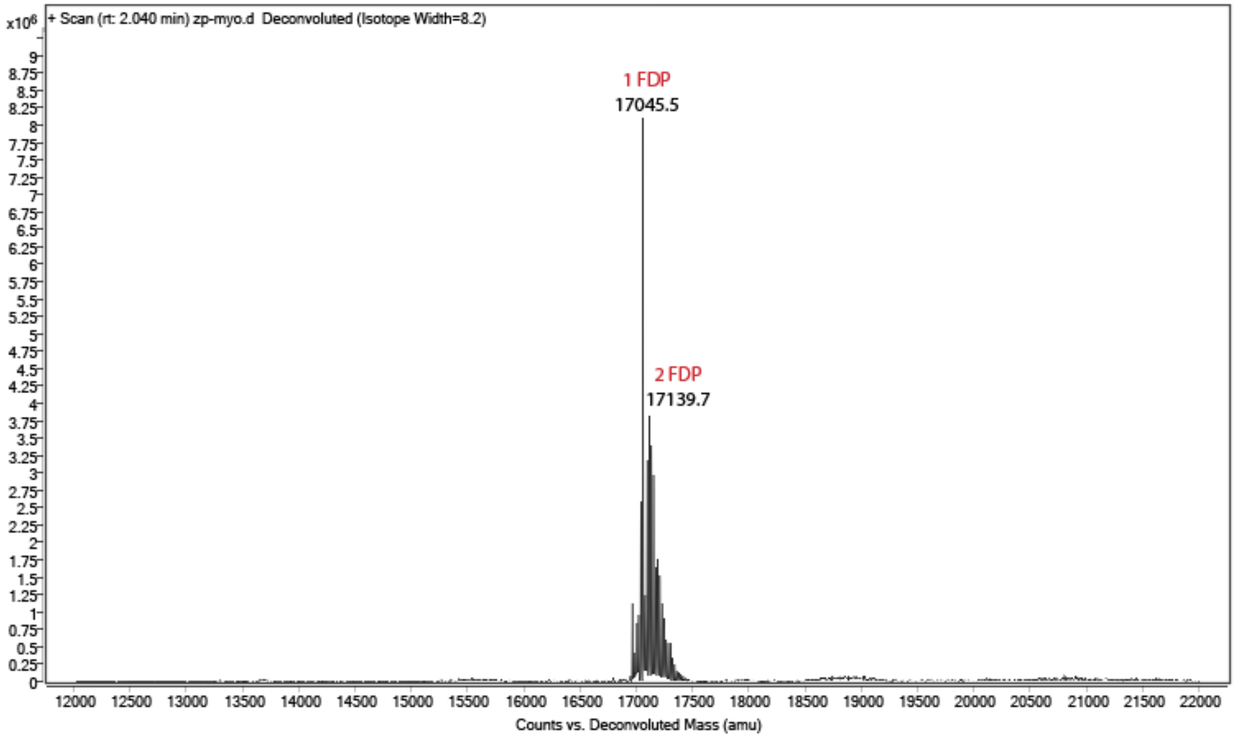

Intact MS Spectrum of Modified Myoglobin (3 Equiv. Acrolein)

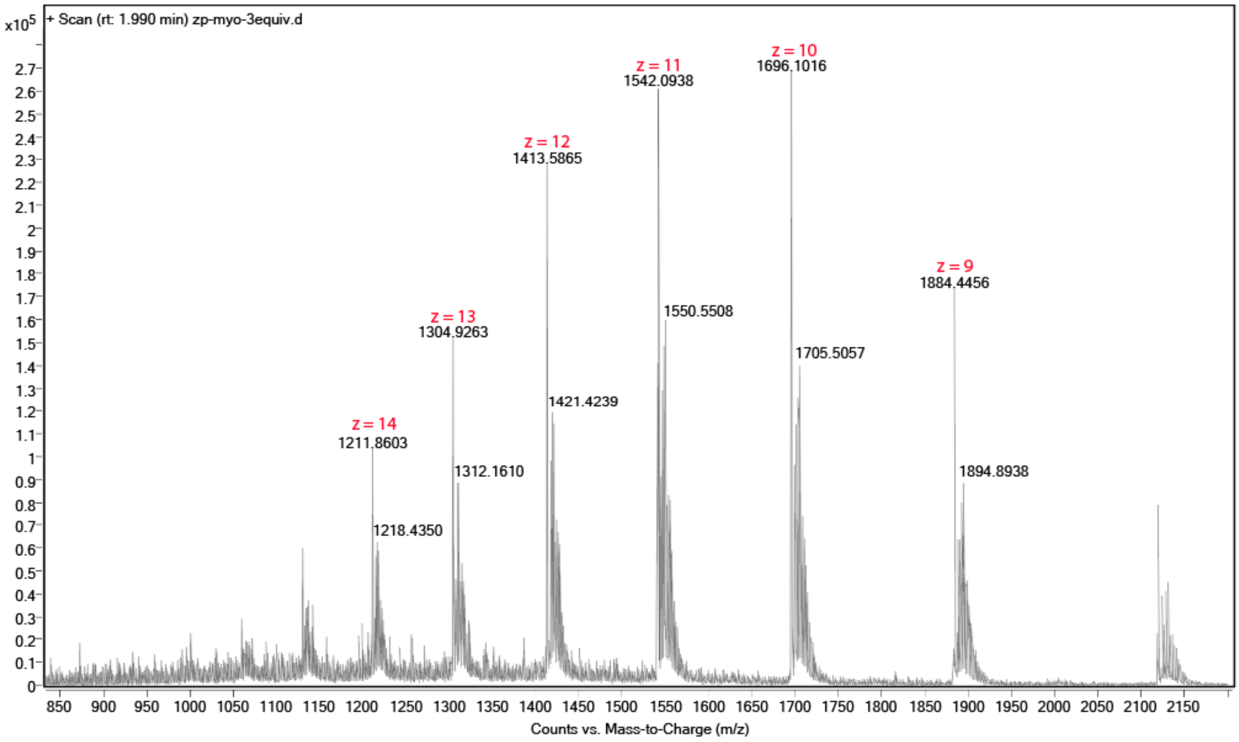

Deconvoluted MS Spectrum of Modified Myoglobin (3 Equiv. Acrolein)

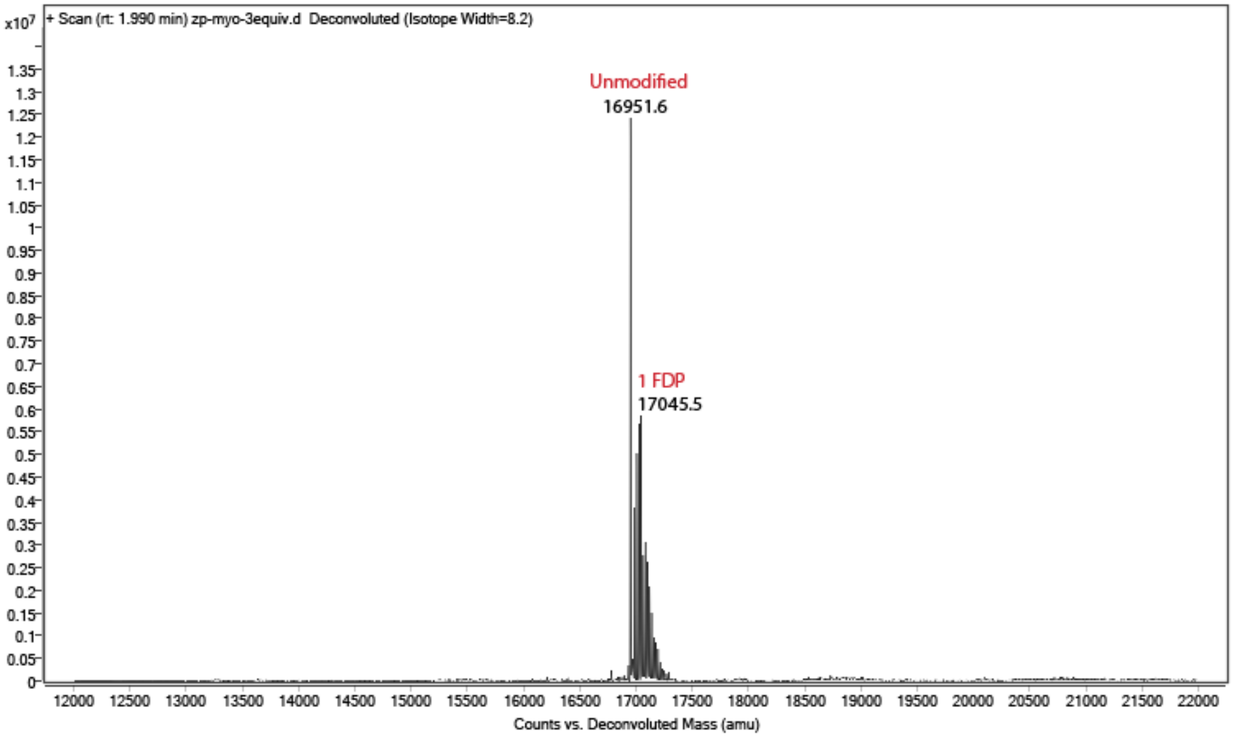

Intact MS Spectrum of Modified Myoglobin (10 Equiv. Acrolein)

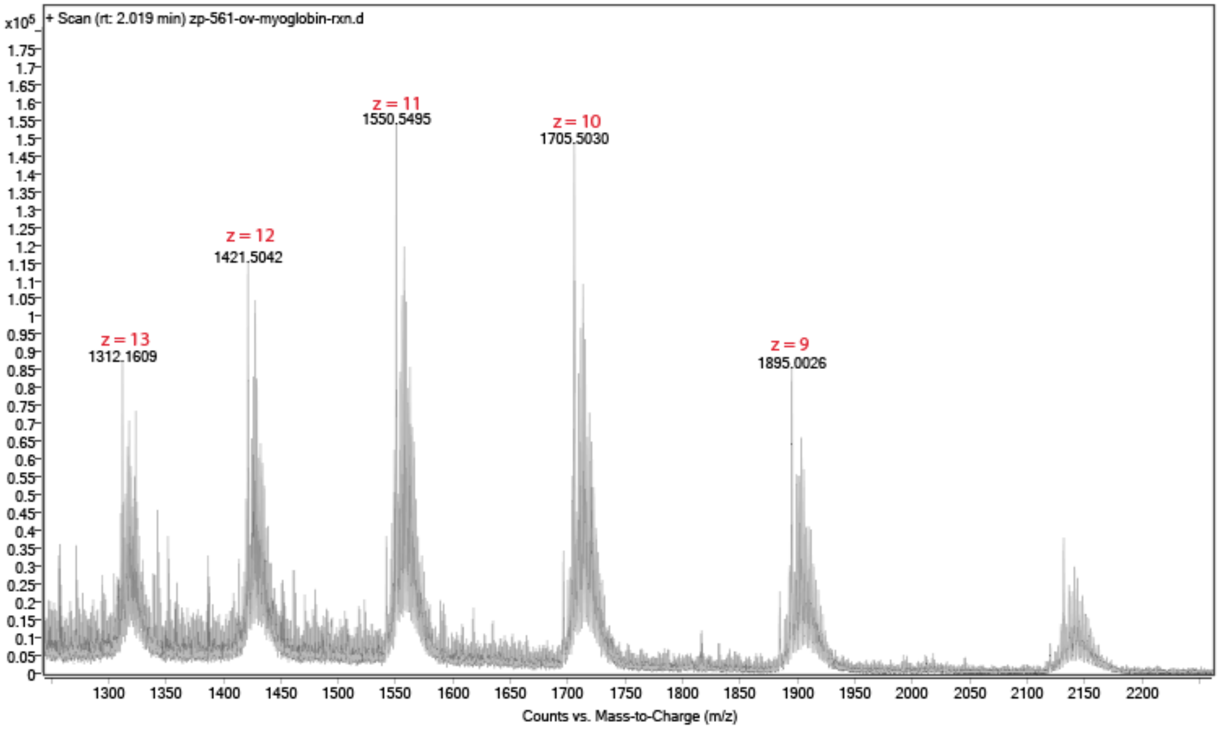

Deconvoluted MS Spectrum of Modified Myoglobin (10 Equiv. Acrolein)

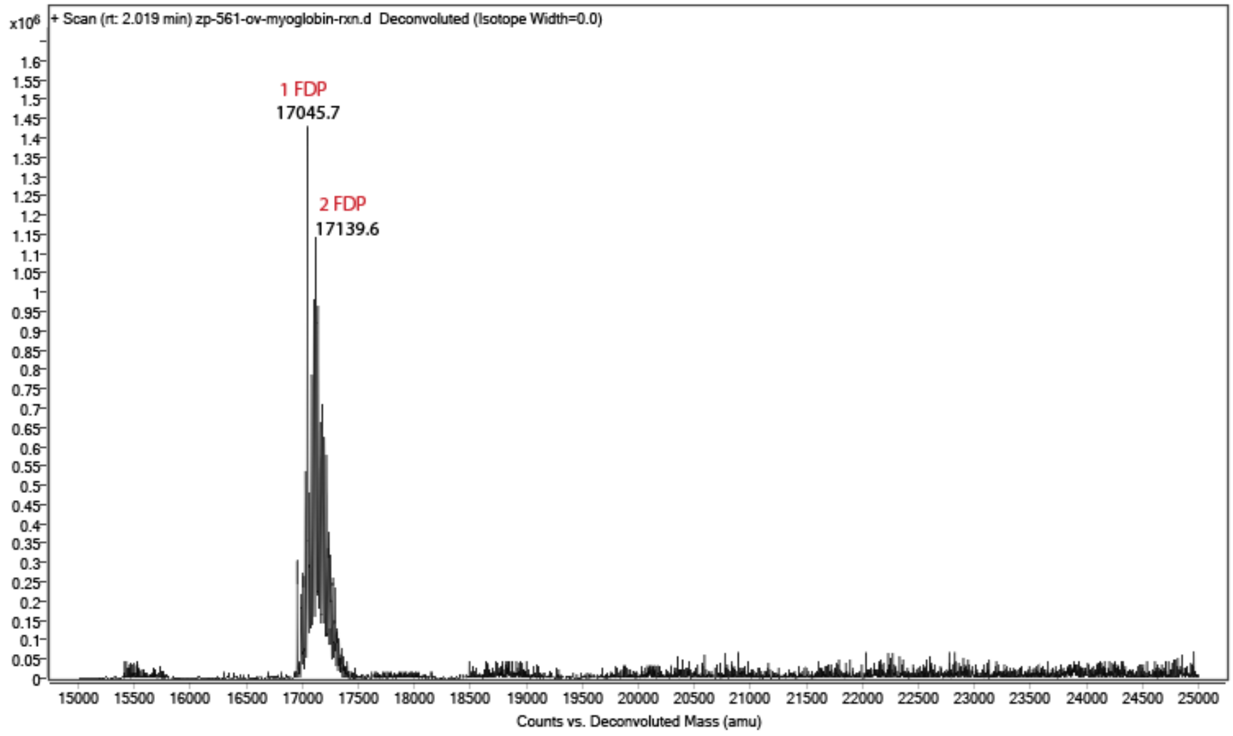

Intact MS Spectrum of Modified Myoglobin (15 Equiv. Acrolein)

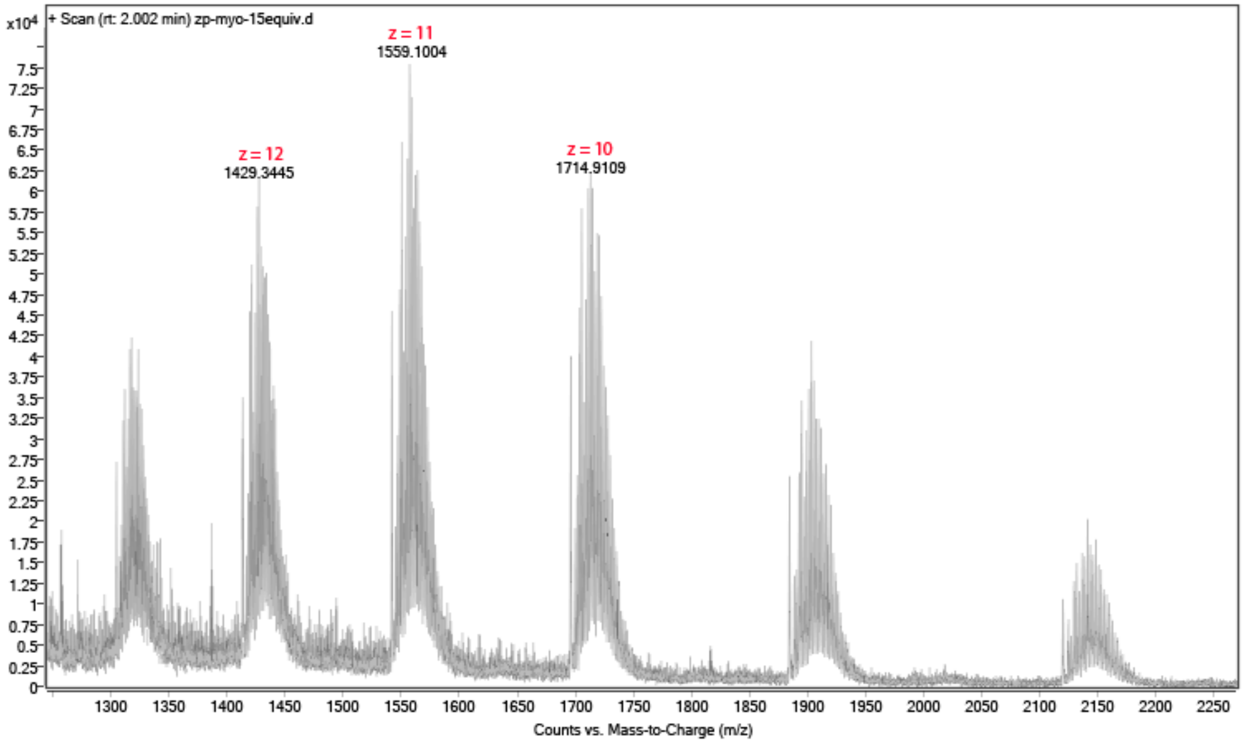

Deconvoluted MS Spectrum of Modified Myoglobin (15 Equiv. Acrolein)

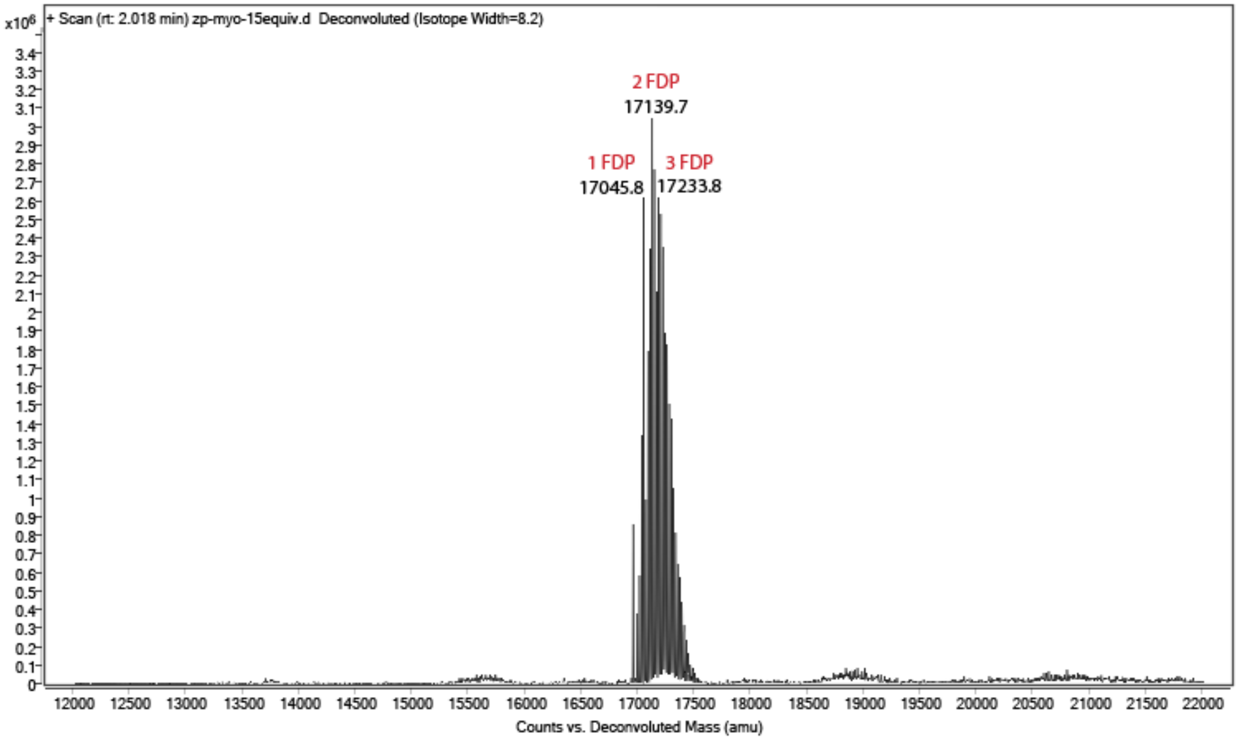

MSMS Analysis of Digested Modified Myoglobin (8 equiv. of Acrolein – Homogeneous Single Modification)

Identified Peptide Fragment: KHGTVVLTALGGILK: (Sequence: AA 63-77, K 63 – MP Lysine)

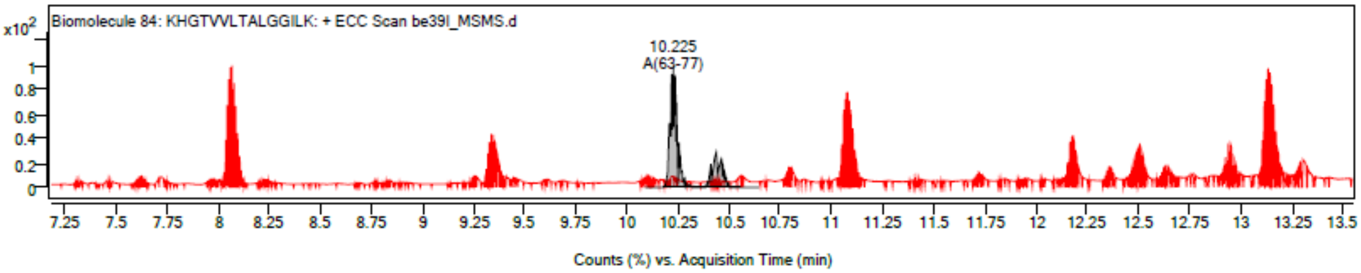

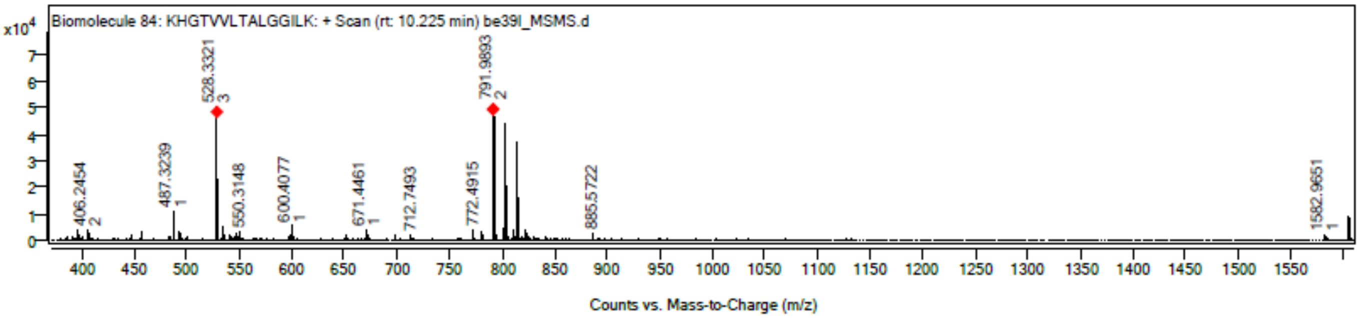

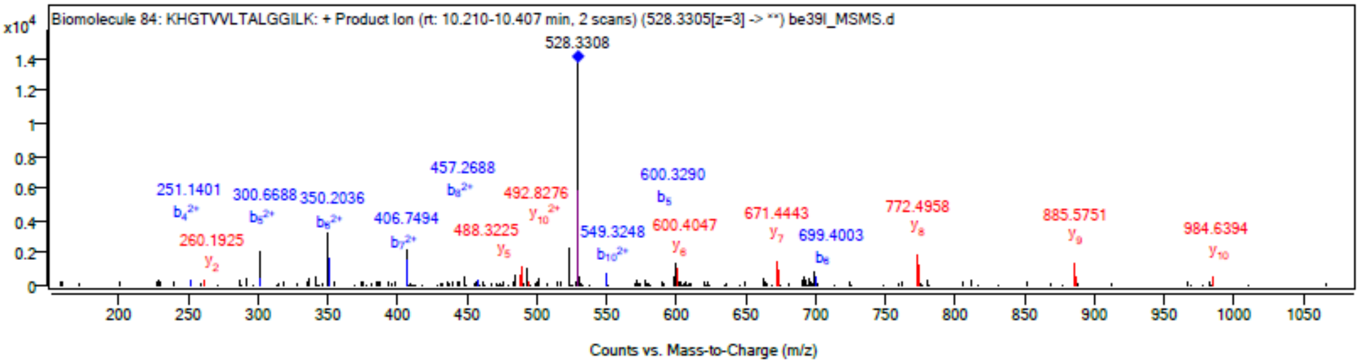

Fragment Spectrum Peaks

m/z	Diff (ppm)	Abund	Ion	Z
260.1925	16.83	380	y2	
488.3225	9.57	1205	y5	
600.4047	5.41	1018	y6	
671.4443	1.09	1449	y7	
772.4958	-4.03	1883	y8	
885.5751	1.92	1341	y9	
984.6394	5.87	563	y10	
492.8276	-2.75	276	y10	
600.3290	14.76	522	b5	
699.4003	8.50	504	b6	
251.1401	-7.14	373	b4	
300.6688	12.41	410	b5	
350.2036	9.07	1677	b6	
406.7494	-1.38	1563	b7	
457.2688	8.38	348	b8	
549.3248	15.30	804	b10	
528.6654	-3.62	5791	Precursor	

Modification of Ubiquitin

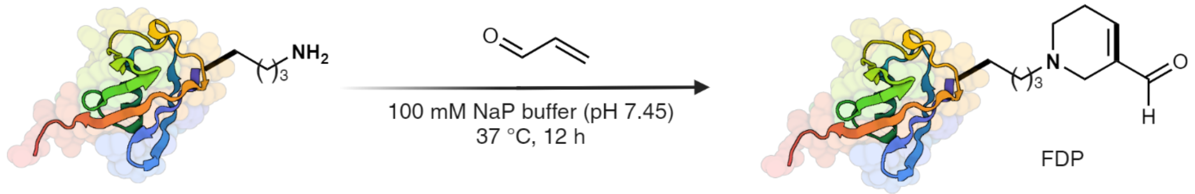

Ubiquitin (2 mg, 0.467 mM) was dissolved in 480 µL of 100 mM NaP buffer (pH 7.45), and acrolein (0.125 µL, 3.74 mM) was added to the mixture from a freshly prepared stock solution (20 µL). The reaction was stirred at 37 °C for 12 hours, after which the crude reaction mixture was passed through Amicon Ultra 3 kDa spin-concentrator and washed with H_2O (5 x 500 µL) to remove the small molecule impurities. The labeled protein was redissolved in 0.1% formic acid in H_2O and analyzed using LC-MS. The conversion was found to be 73% with 46% 1 FDP modification, 27% 2 FDP modifications, and 37% unmodified ubiquitin.

Modification	Mass	Conversion
Unmodified	8565.2	27%
1 FDP	8659.0 (**+94**)	46%
2 FDP	8753.2 (**+94**)	27%

Intact MS Spectrum of Starting Ubiquitin

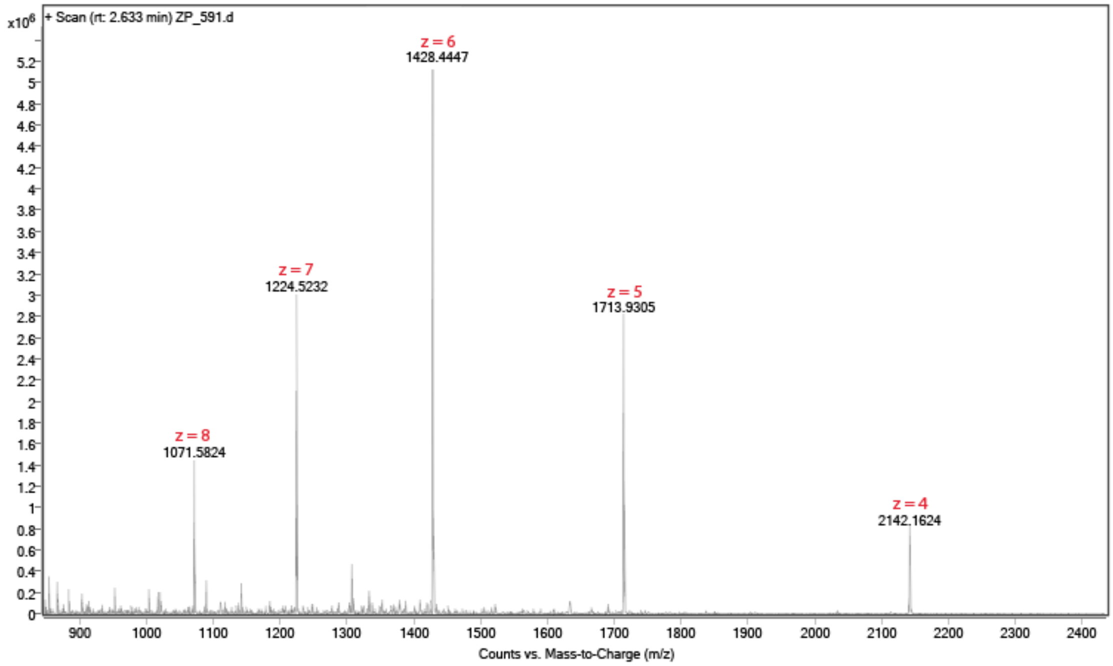

Deconvoluted MS Spectrum of Starting Ubiquitin

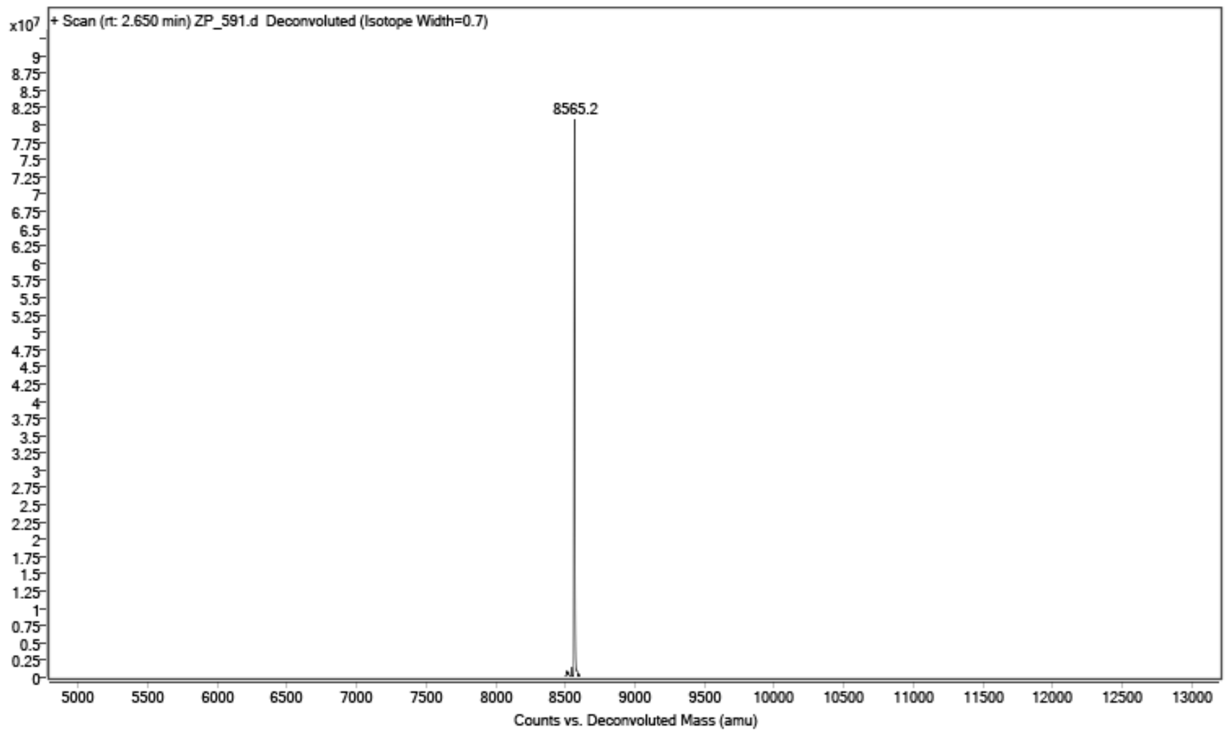

Intact MS Spectrum of Modified Ubiquitin

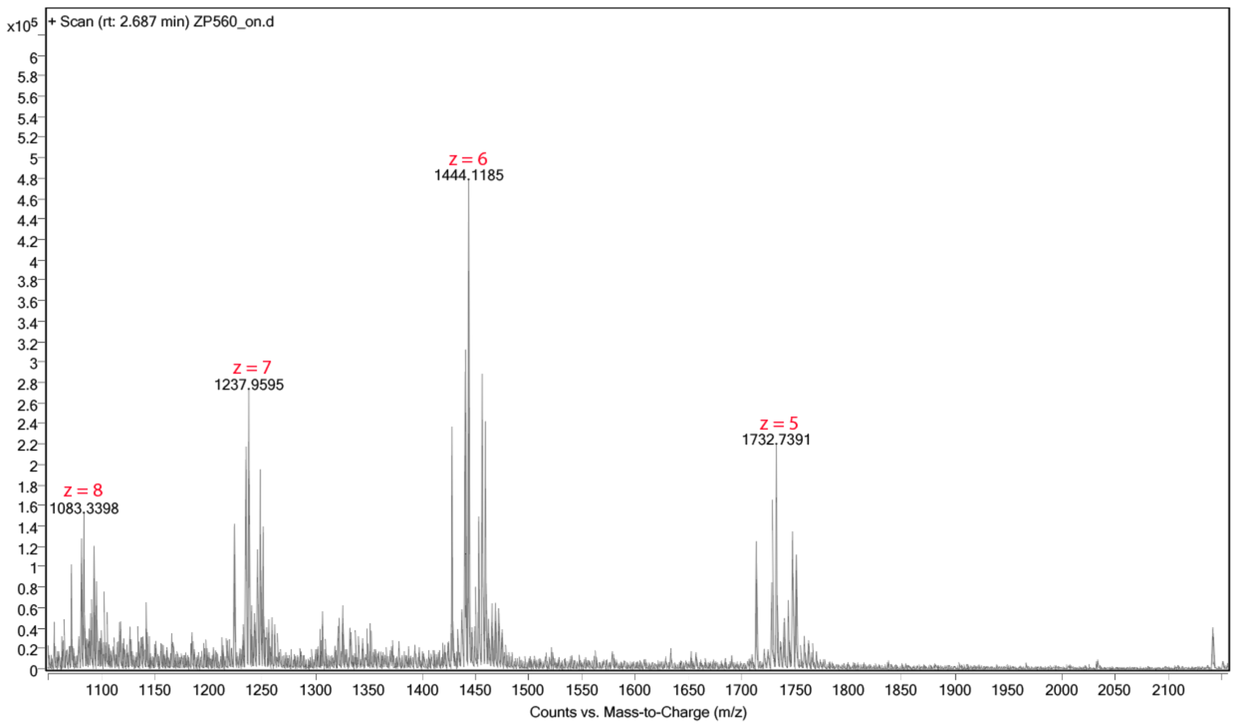

Deconvoluted MS Spectrum of Modified Ubiquitin

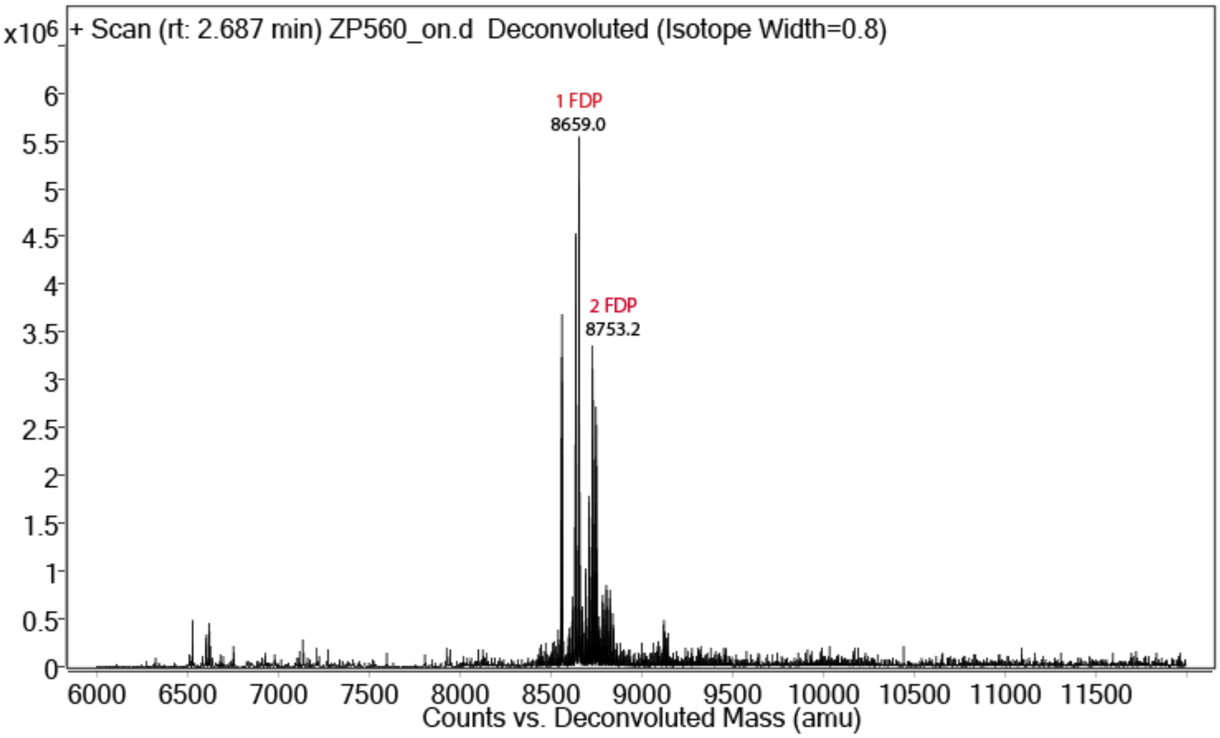

Modification of Lysozyme Chicken

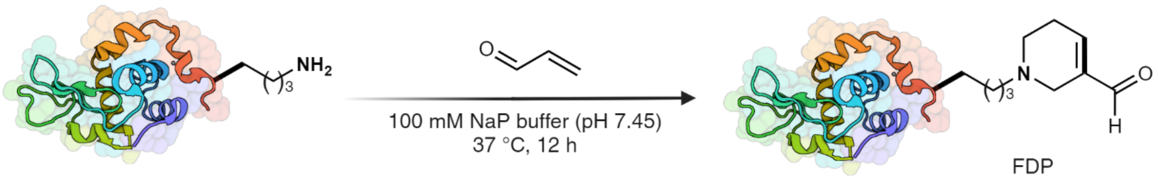

Lysozyme chicken (2 mg, 0.280 mM) was dissolved in 480 µL of 100 mM NaP buffer (pH 7.45), and acrolein (0.075 µL, 2.23 mM) was added to the mixture from a freshly prepared stock solution (20 µL). The reaction was stirred at 37 °C for 12 hours, after which the crude reaction mixture was passed through Amicon Ultra 3 kDa spin-concentrator and washed with H_2O (5 x 500 µL) to remove the small molecule impurities. The labeled protein was redissolved in 0.1% formic acid in H_2O and analyzed using LC-MS. The conversion was found to be >95% with 54% 1 FDP modification and 46% 2 FDP modifications.

Modification	Mass	Conversion
Unmodified	14305.6	N/A
1 FDP	14399.4 (**+94**)	54%
2 FDP	14493.5 (**+94**)	46%

Intact MS Spectrum of Starting Lysozyme Chicken

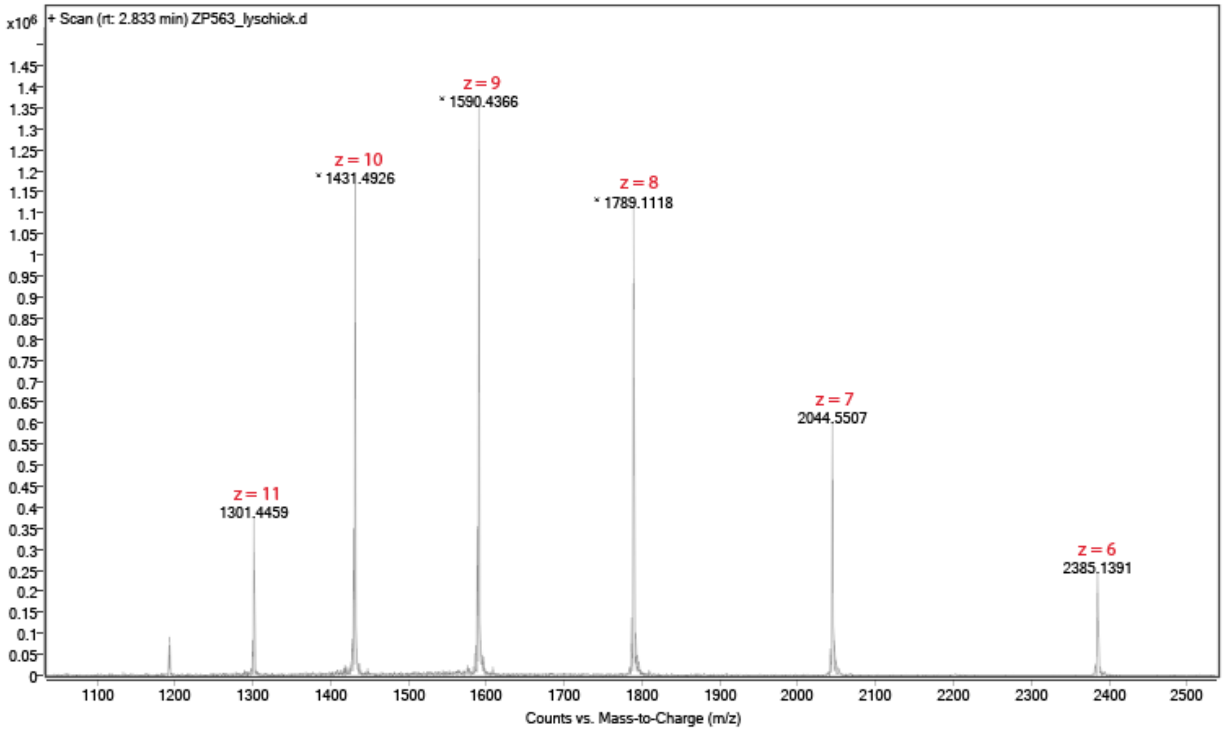

Deconvoluted MS Spectrum of Starting Lysozyme Chicken

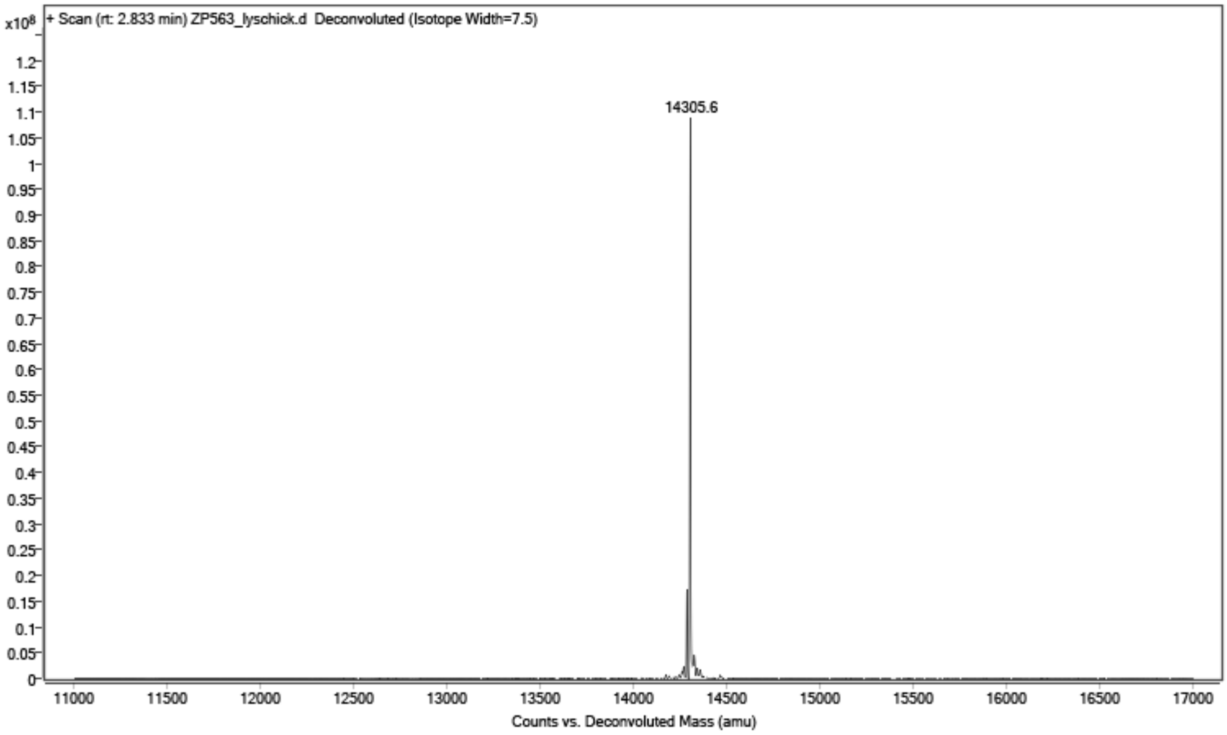

Intact MS Spectrum of Modified Lysozyme Chicken

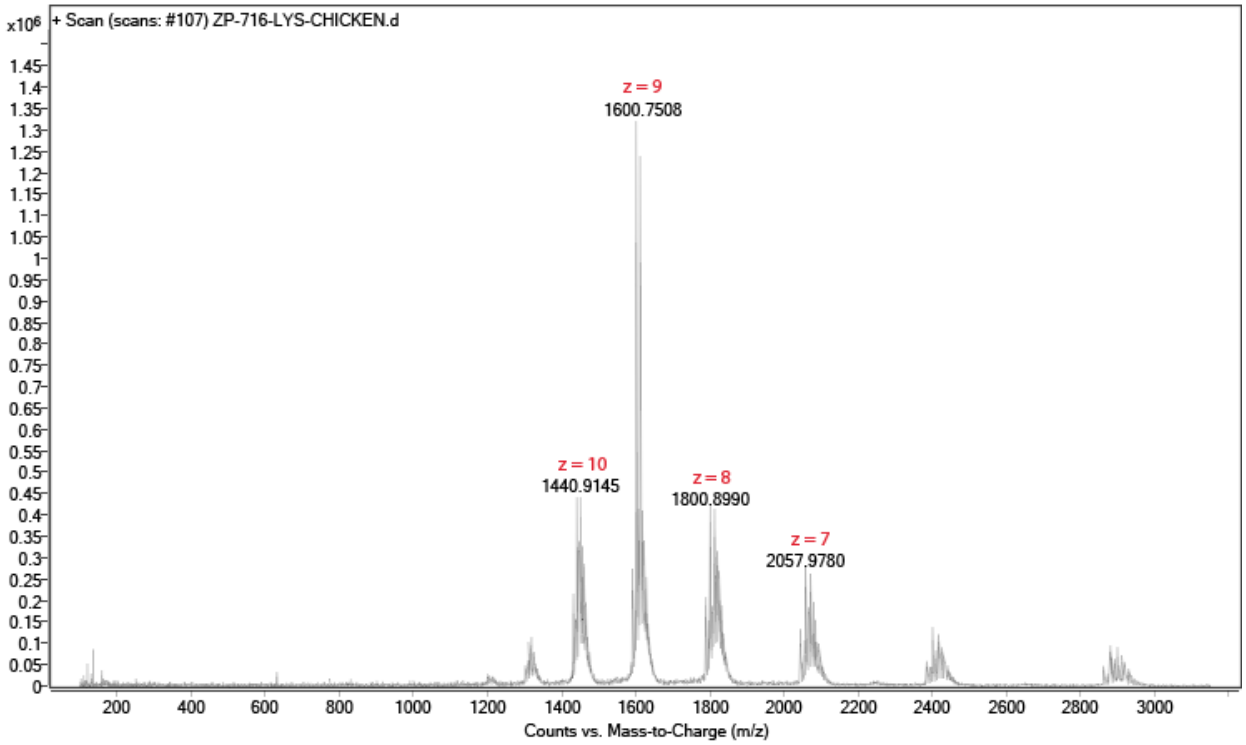

Deconvoluted MS Spectrum of Modified Lysozyme Chicken

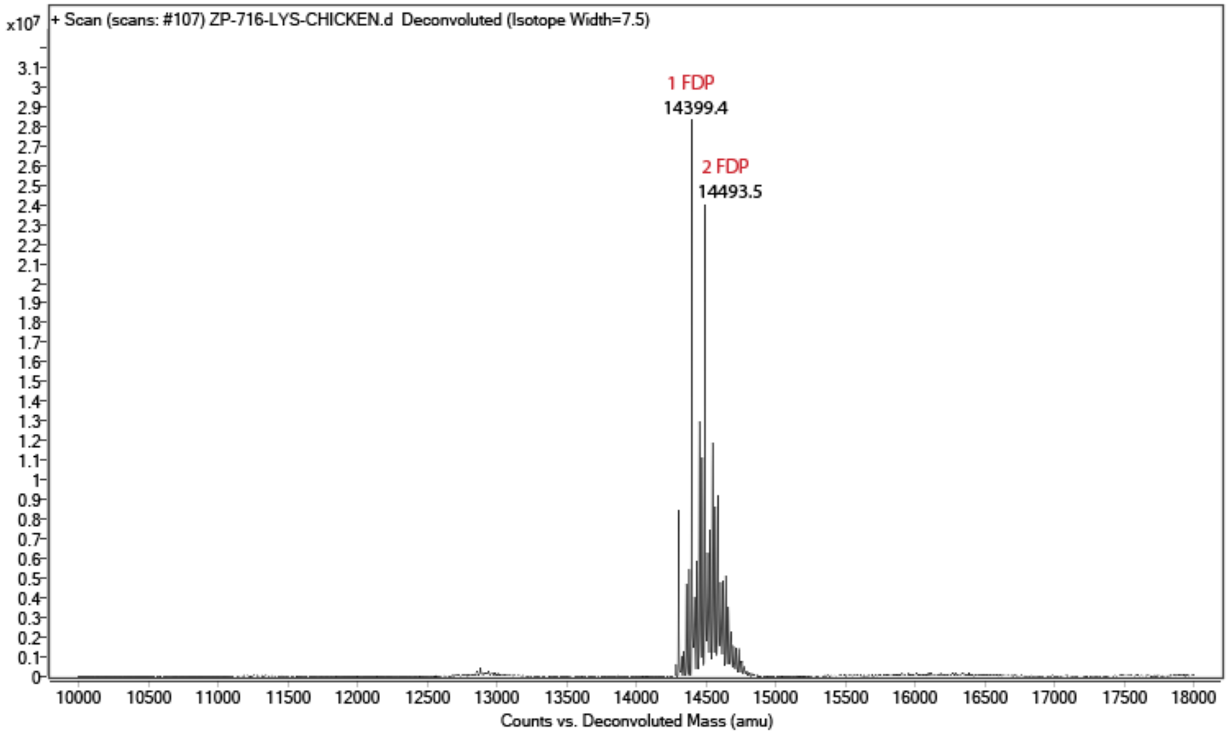

MSMS Analysis of Digested Modified Lysozyme Chicken

Identified Peptide Fragment: KIVSDGNGMNAWVAWR: (Sequence: AA 97-112, K97 – MP Lysine)

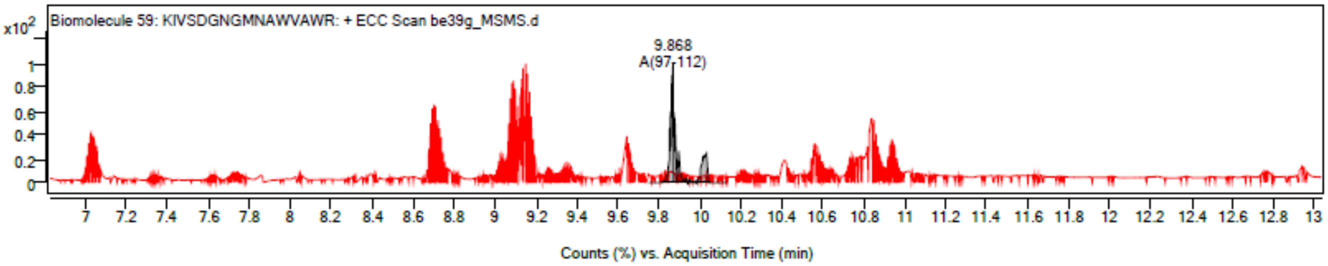

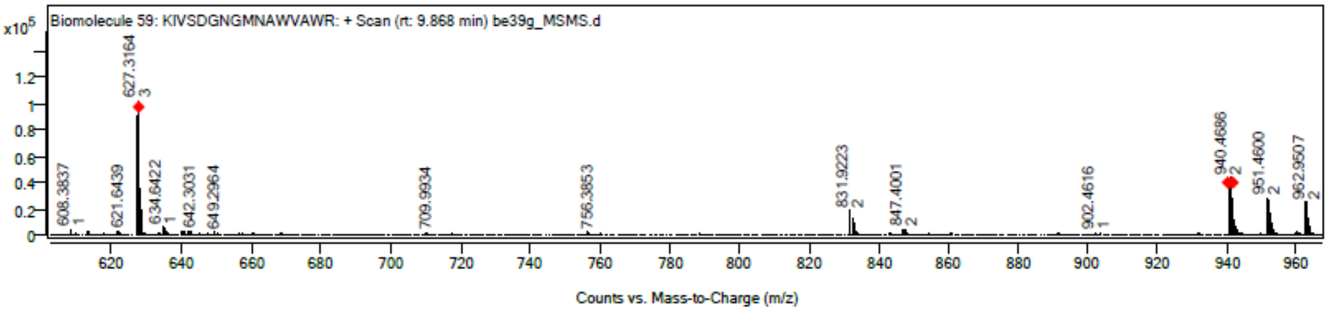

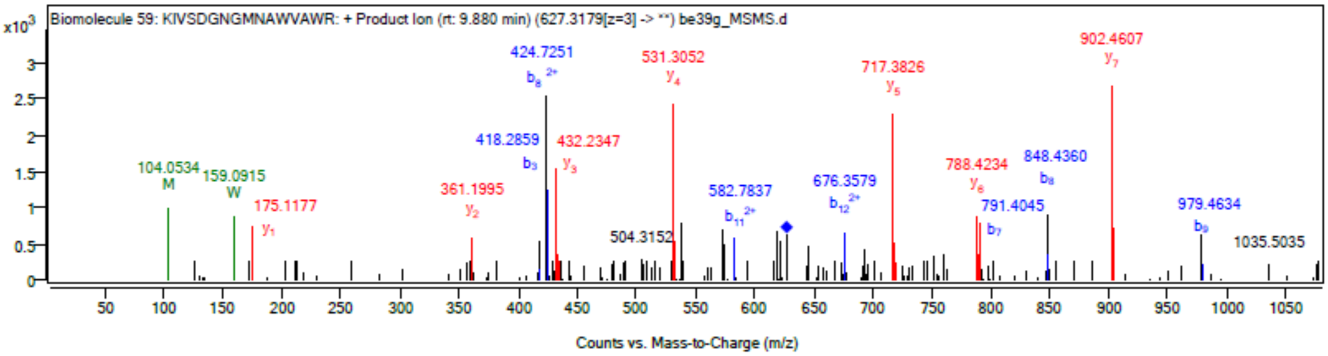

Fragment Spectrum Peaks

m/z	Diff (ppm)	Abund	Ion	Z
175.1177	6.95	740	y1	
361.1995	-3.53	585	y2	
432.2347	1.56	1539	y3	
531.3052	-2.63	2421	y4	
717.3826	0.72	2288	y5	
788.4234	-3.99	873	y6	
902.4607	2.72	2670	y7	
418.2859	18.96	147	b3	
791.4045	32.53	259	b7	
848.4360	3.17	357	b8	
979.4634	16.13	216	b9	
424.7251	-5.02	1245	b8	
582.7837	-0.81	586	b11	
676.3579	-49.24	652	b12	
104.0534	-5.61	998	M	
159.0915	1.25	883	W	

Identified Peptide Fragment: KIVSDGNGMNAWVAWR: (Sequence: AA 97-112, K97 – MP Lysine)

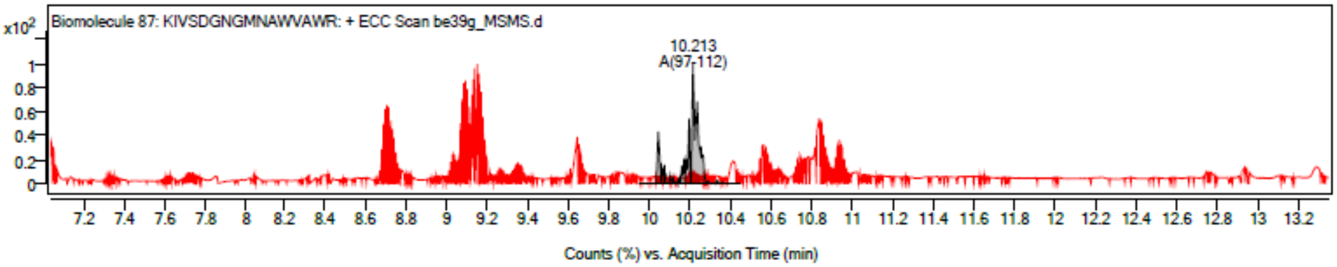

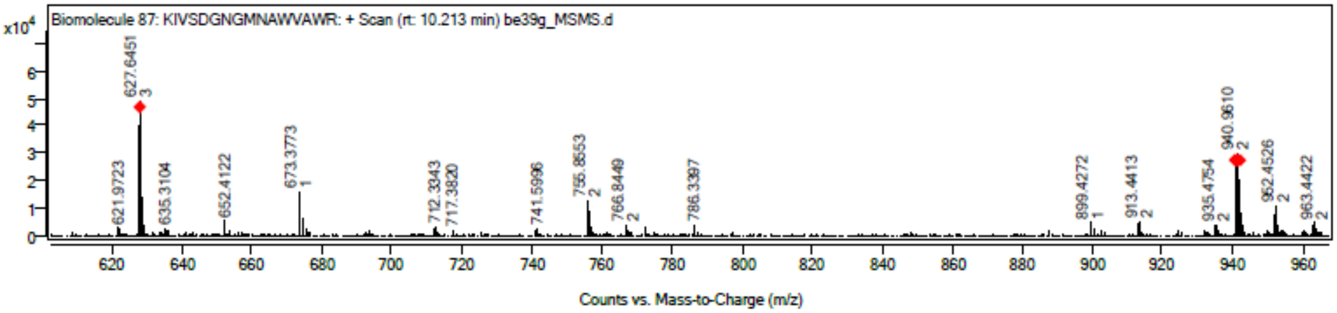

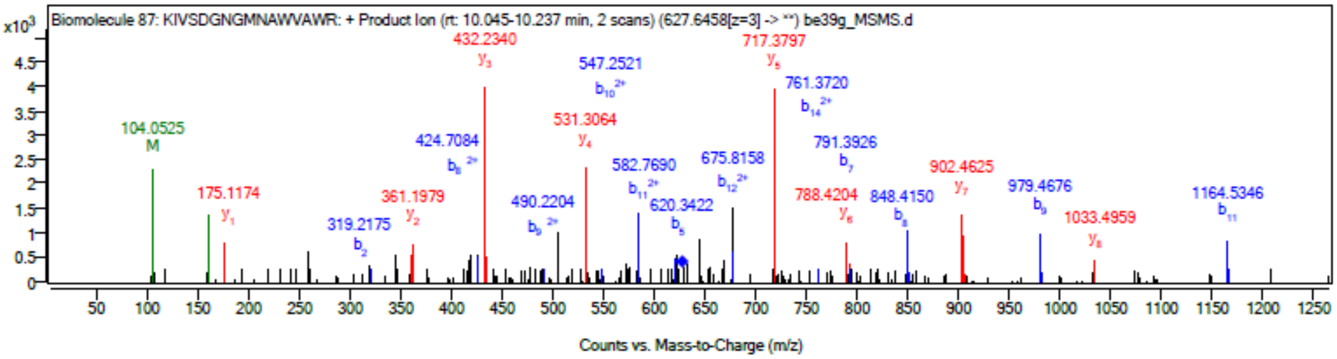

Fragment Spectrum Peaks

m/z	Diff (ppm)	Abund	Ion	Z
175.1174	9.08	799	y1	
361.1979	1.01	769	y2	
432.2340	3.15	3969	y3	
531.3064	-4.89	2315	y4	
717.3797	4.68	3947	y5	
788.4204	-0.24	797	y6	
902.4625	0.70	1356	y7	
1033.4959	7.47	432	y8	
359.1973	-5.83	523	y5	
319.2175	24.98	261	b2	
620.3422	17.12	469	b5	
791.3926	47.61	368	b7	
848.4150	27.90	1048	b8	
979.4676	11.77	958	b9	
1164.5346	21.14	833	b11	
424.7084	34.27	497	b8	
490.2204	46.53	266	b9	
547.2521	23.00	266	b10	
582.7690	24.51	1384	b11	
675.8158	10.49	625	b12	
761.3720	7.03	263	b14	
104.0525	3.65	2298	M	
159.0912	2.77	1357	W	

Modification of Lysozyme Human

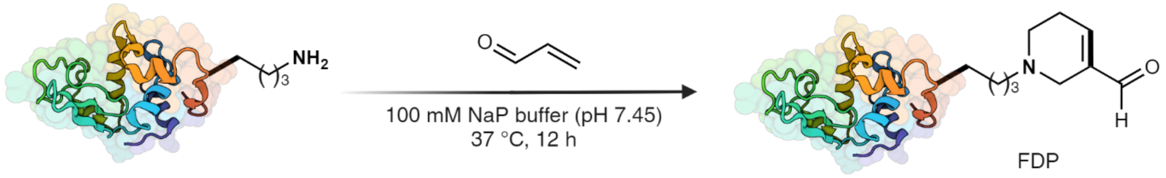

Lysozyme human (2 mg, 0.272 mM) was dissolved in 480 µL of 100 mM NaP buffer (pH 7.45), and acrolein (0.073 µL, 2.18 mM) was added to the mixture from a freshly prepared stock solution (20 µL). The reaction was stirred at 37 °C for 12 hours, after which the crude reaction mixture was passed through Amicon Ultra 3 kDa spin-concentrator and washed with H_2O (5 x 500 µL) to remove the small molecule impurities. The labeled protein was redissolved in 0.1% formic acid in H_2O and analyzed using LC-MS. The conversion was found to be >95% with 56% 1 FDP modification and 44% 2 FDP modifications.

Modification	Mass	Conversion
Unmodified	14692.7	N/A
1 FDP	14786.8 (+**94**)	56%
2 FDP	14880.9 (+**94**)	44%

Intact MS Spectrum of Starting Lysozyme Human

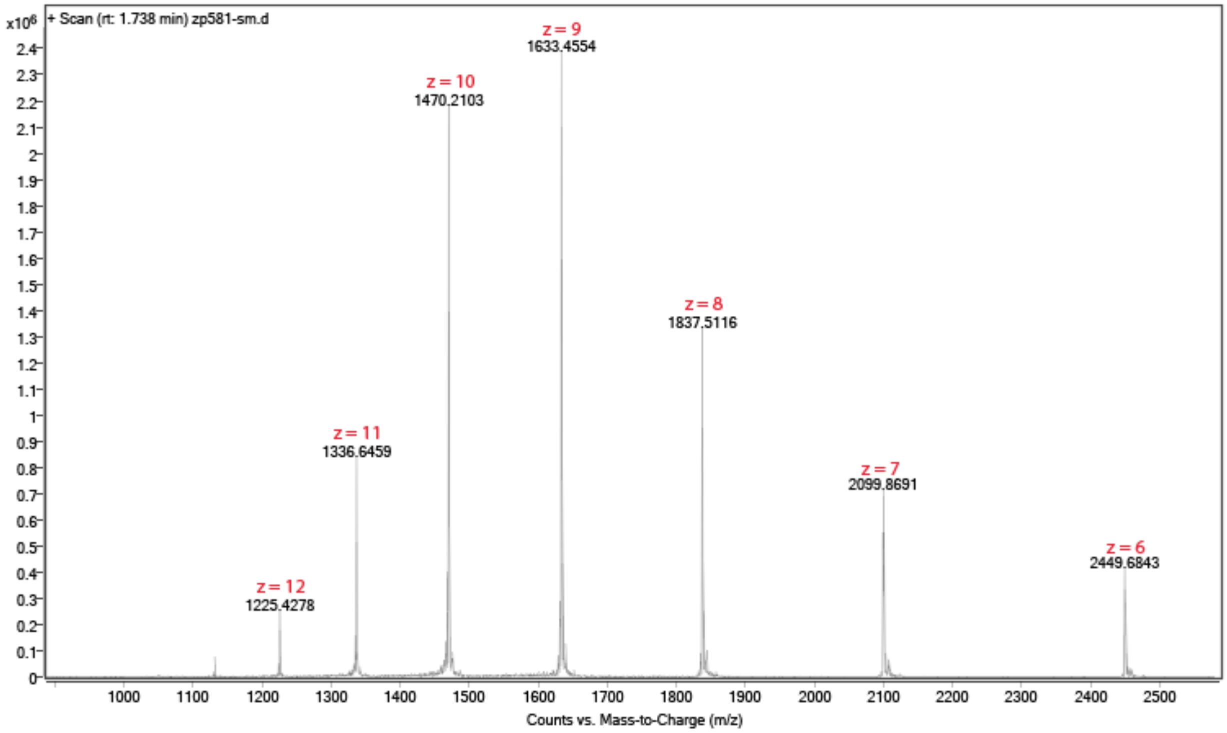

Deconvoluted MS Spectrum of Starting Lysozyme Human

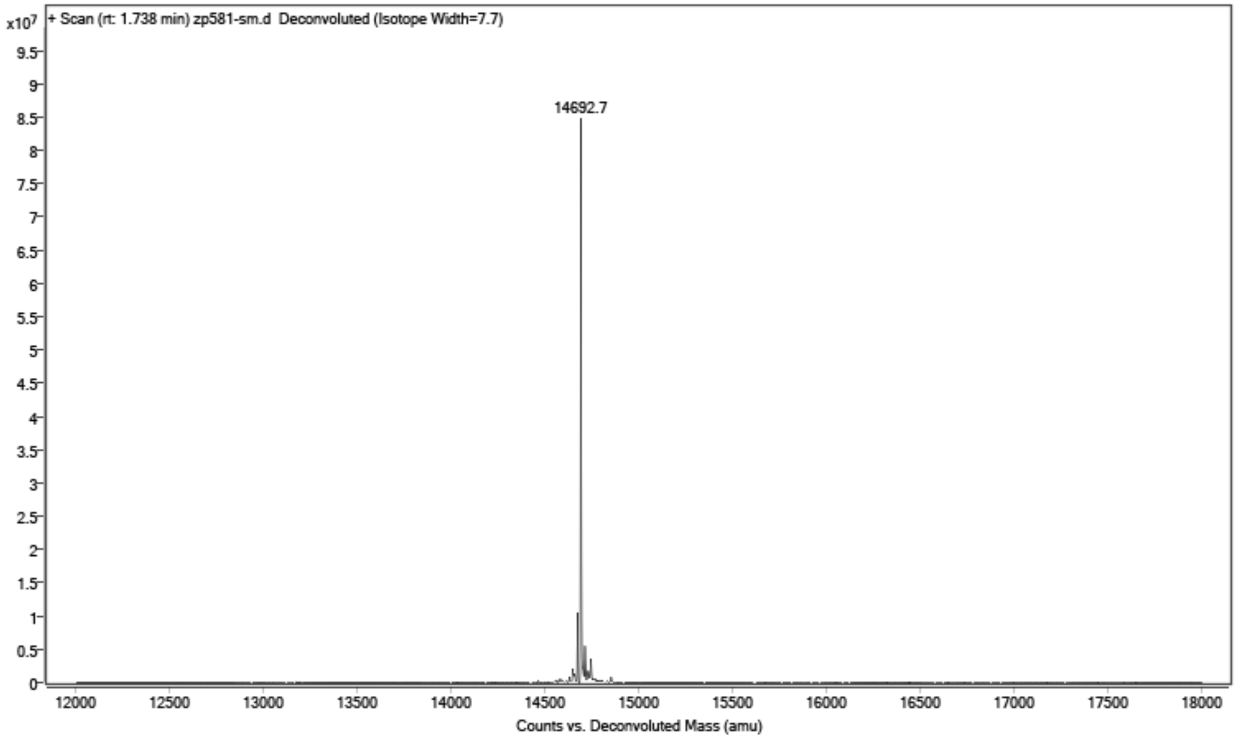

Intact MS Spectrum of Modified Lysozyme Human

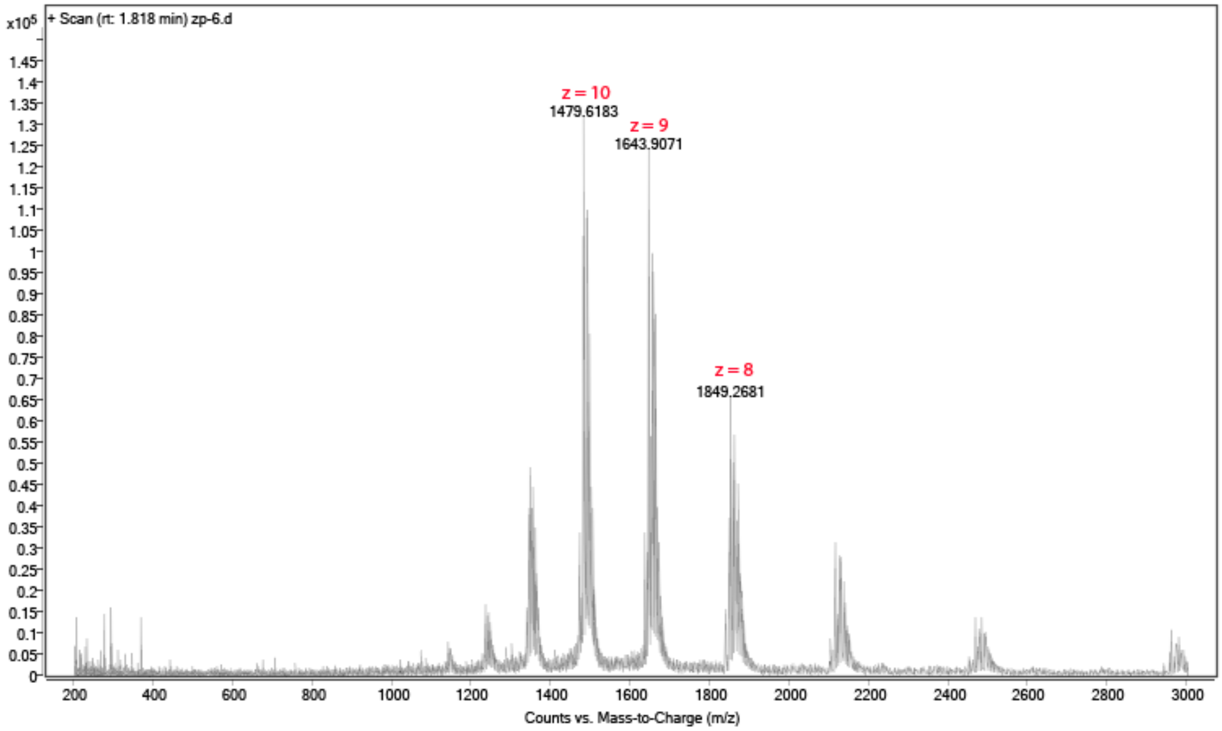

Deconvoluted MS Spectrum of Modified Lysozyme Human

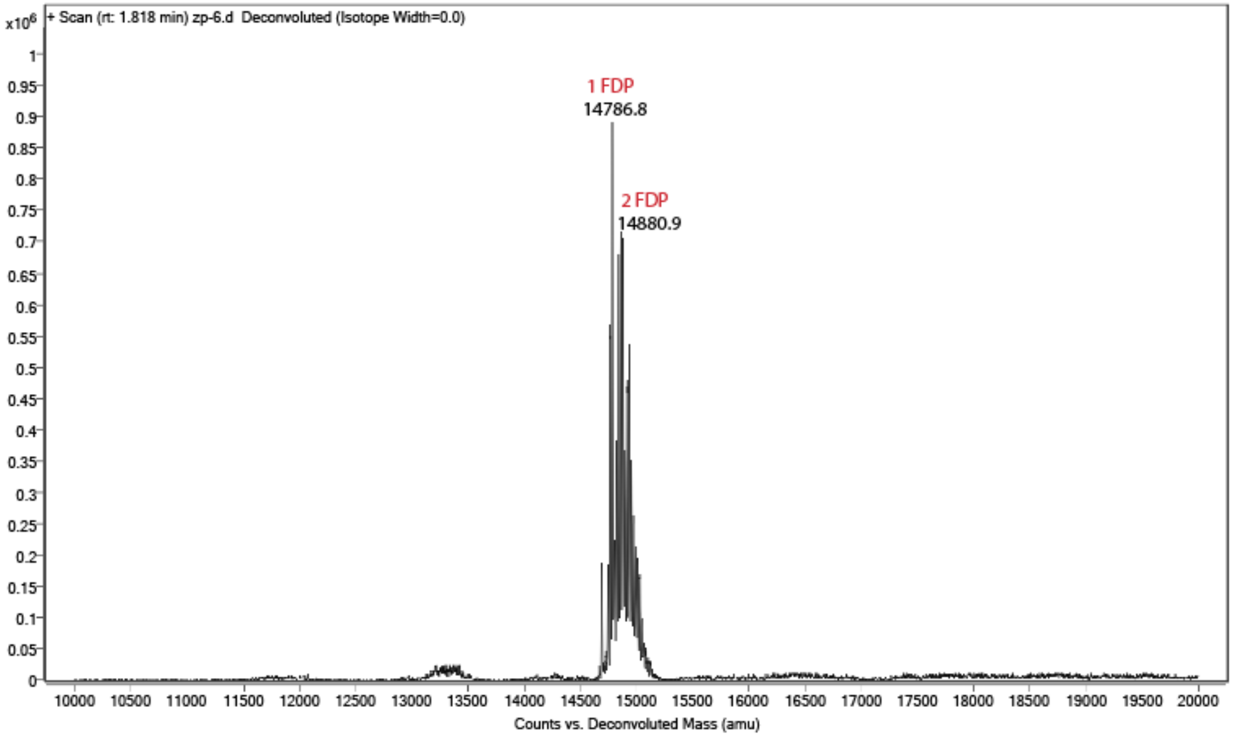

Modification of Aprotinin

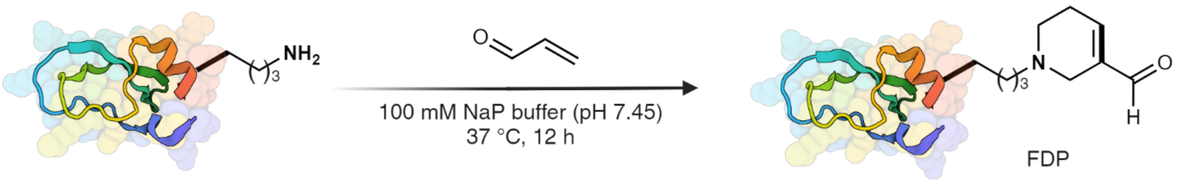

Aprotinin (2 mg, 0.614 mM) was dissolved in 480 µL of 100 mM NaP buffer (pH 7.45), and acrolein (0.164 µL, 4.91 mM) was added to the mixture from a freshly prepared stock solution (20 µL). The reaction was stirred at 37 °C for 12 hours, after which the crude reaction mixture was passed through Amicon Ultra 3 kDa spin-concentrator and washed with H_2O (5 x 500 µL) to remove the small molecule impurities. The labeled protein was redissolved in 0.1% formic acid in H_2O and analyzed using LC-MS. The conversion was found to be >95% with 27% 2 FDP modification, 42% 3 FDP modifications, and 31% for 4 FDP.

Modification	Mass	Conversion
Unmodified	6511.1	N/A
2 FDP	6699.9 (**+186**)	27%
3 FDP	6793.9 (**+94**)	42%
4 FDP	6887.6 (**+94**)	31%

Intact MS of Starting Aprotinin

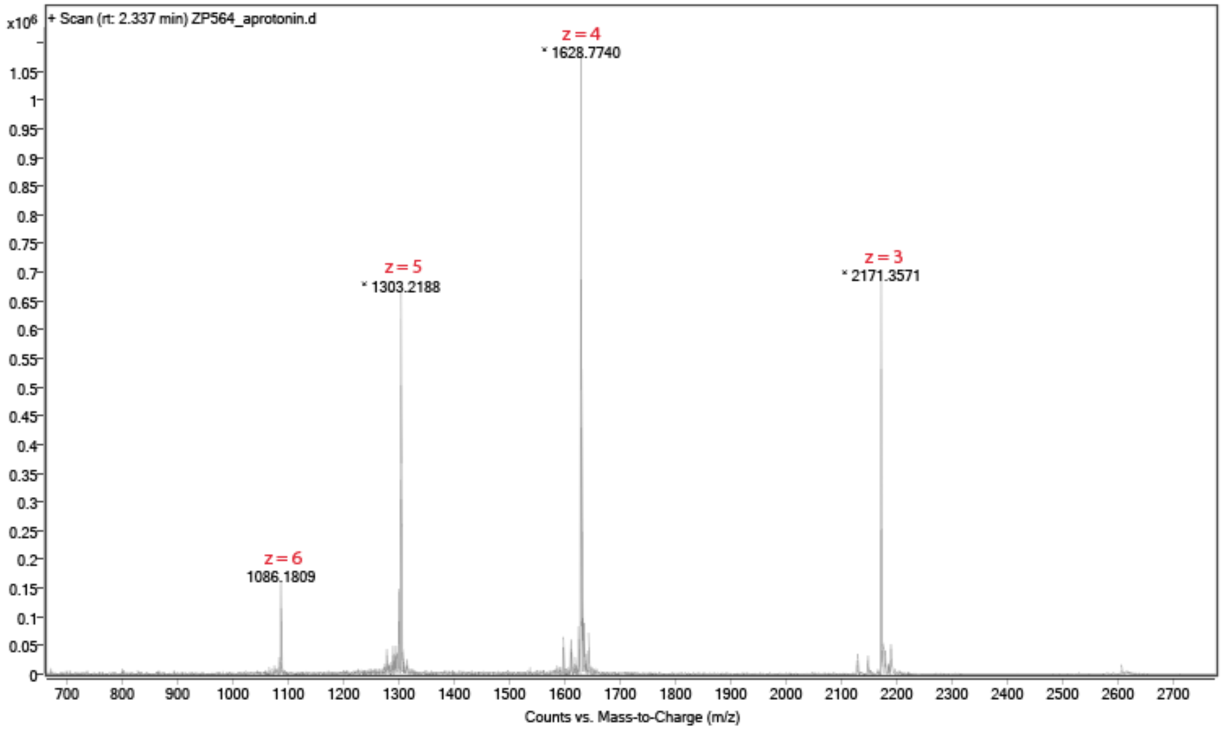

Deconvoluted MS of Starting Aprotinin

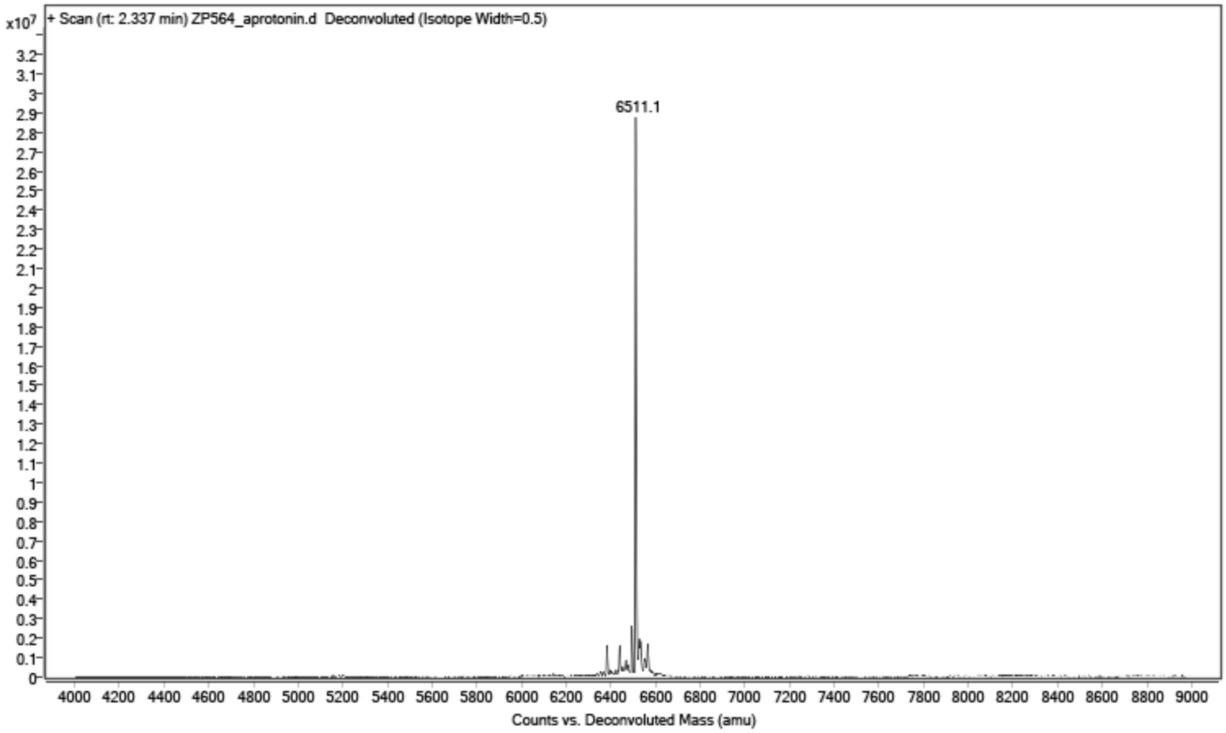

Intact MS of Modified Aprotinin

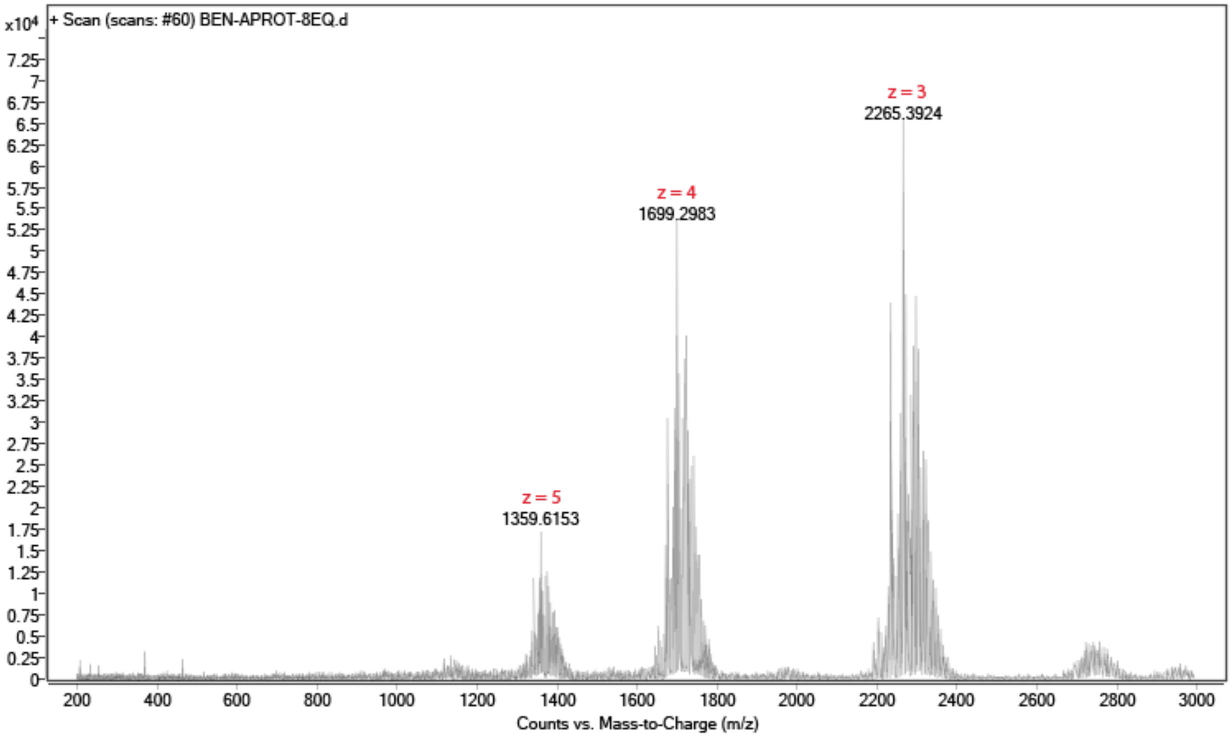

Deconvoluted MS of Modified Aprotinin

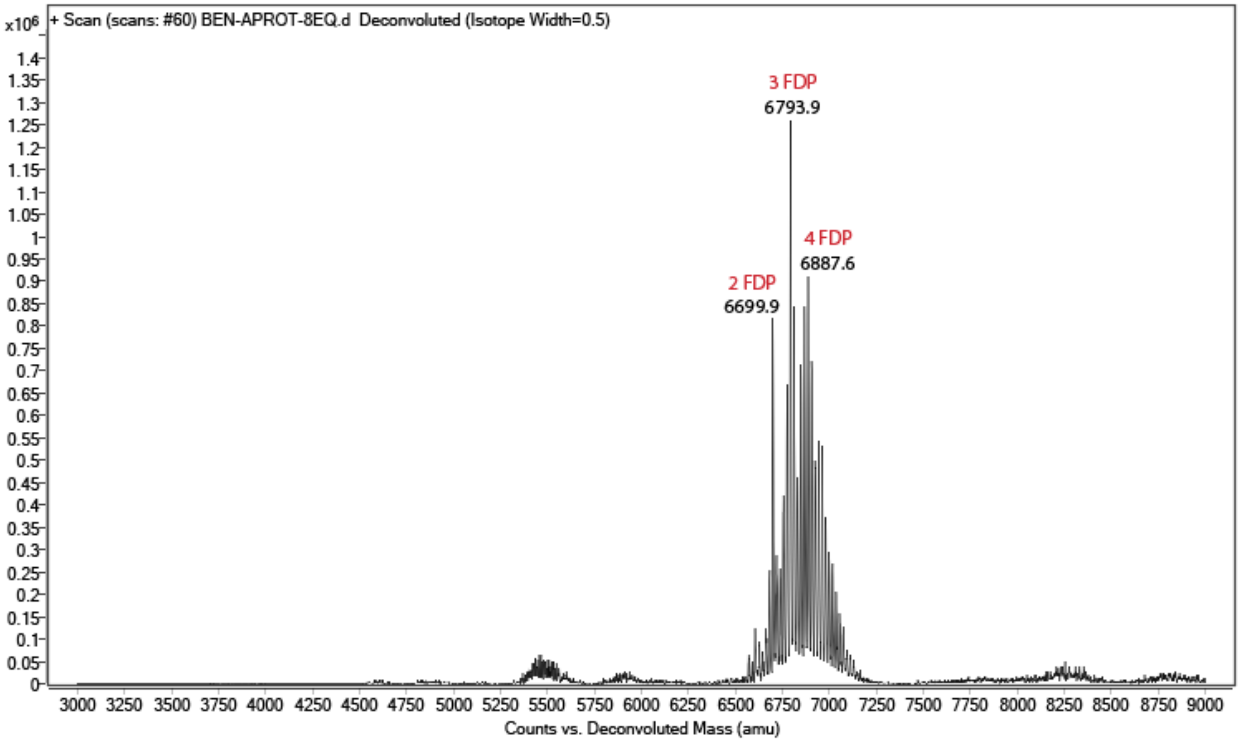

Modification of β-lactoglobulin

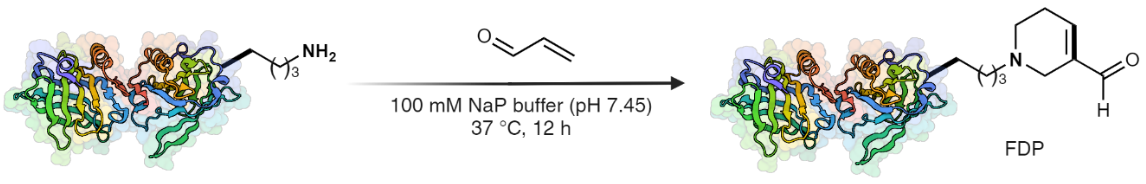

β-lactoglobulin (2 mg, 0.218 mM) was dissolved in 480 µL of 100 mM NaP buffer (pH 7.45), and acrolein (0.058 µL, 1.74 mM) was added to the mixture from a freshly prepared stock solution (20 µL). The reaction was stirred at 37 °C for 12 hours, after which the crude reaction mixture was passed through Amicon Ultra 3 kDa spin-concentrator and washed with H_2O (5 x 500 µL) to remove the small molecule impurities. The labeled protein was redissolved in 0.1% formic acid in H_2O and analyzed using LC-MS. The conversion was found to be >95% with full conversion to 1 FDP modification.

Modification	Mass	Conversion
Unmodified	18363.5	N/A
1 FDP	18457.5 (**+94**)	>95%

Intact MS of Starting β-lactoglobulin

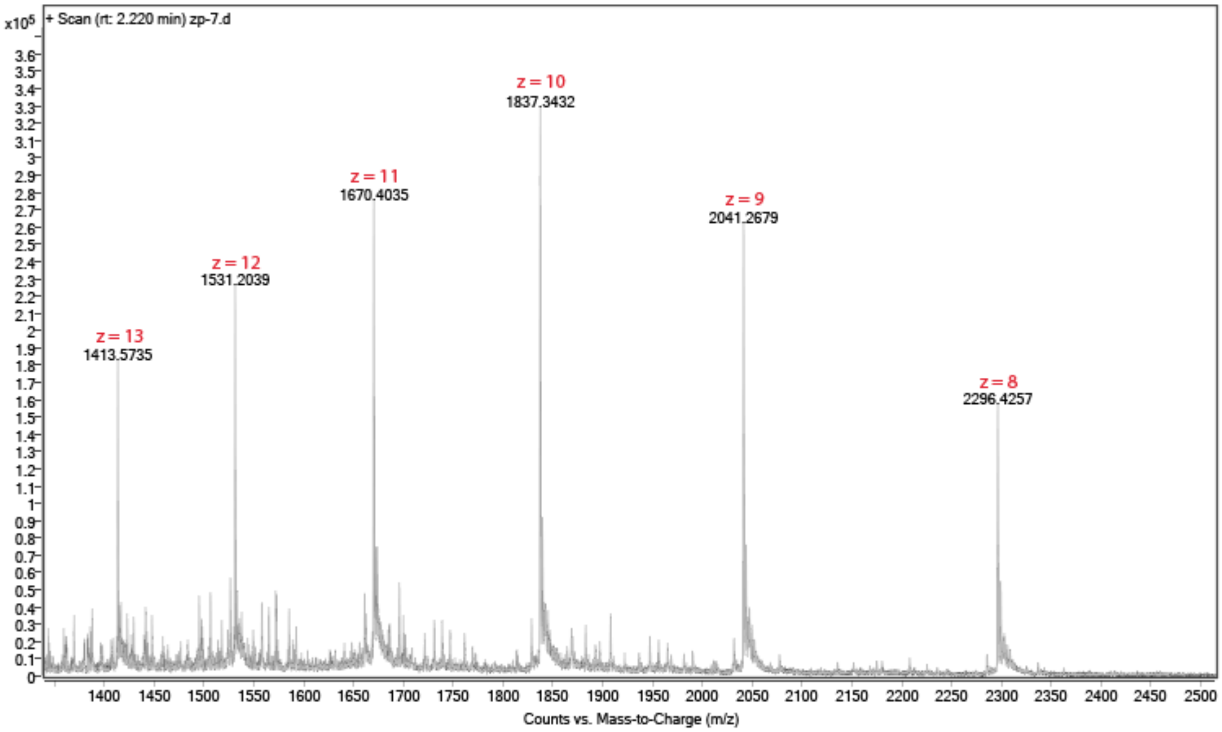

Deconvoluted MS of Starting β-lactoglobulin

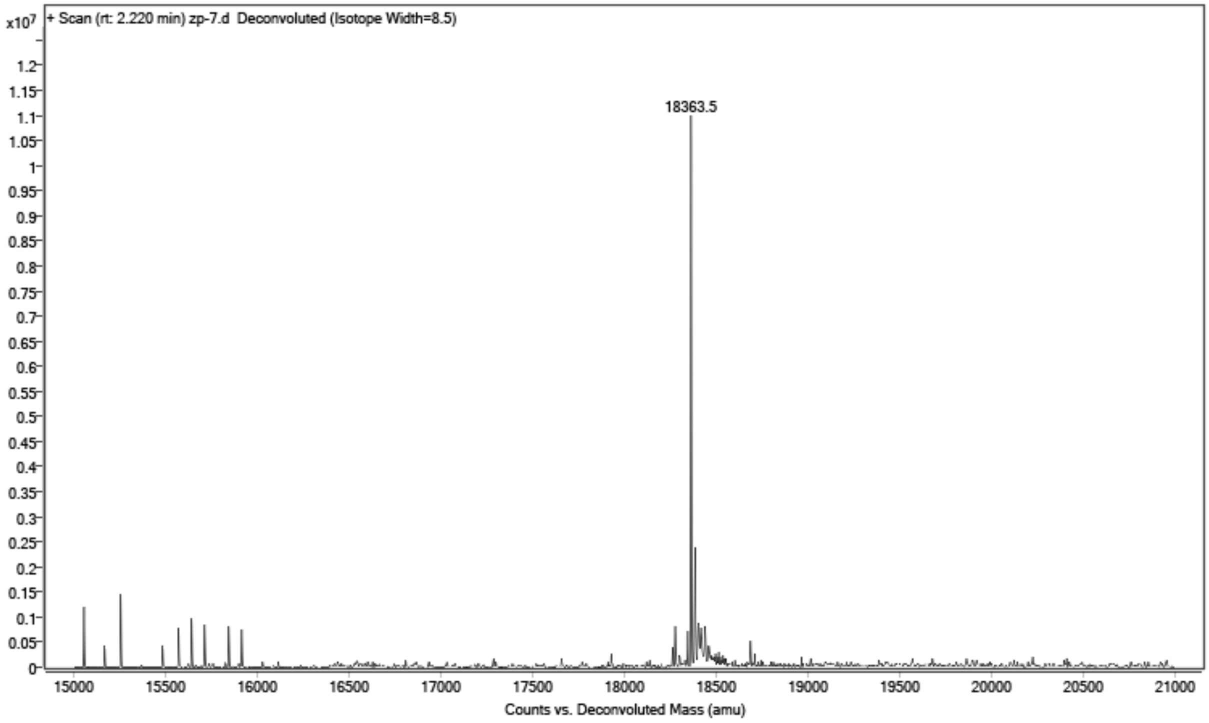

Intact MS of Modified β-lactoglobulin

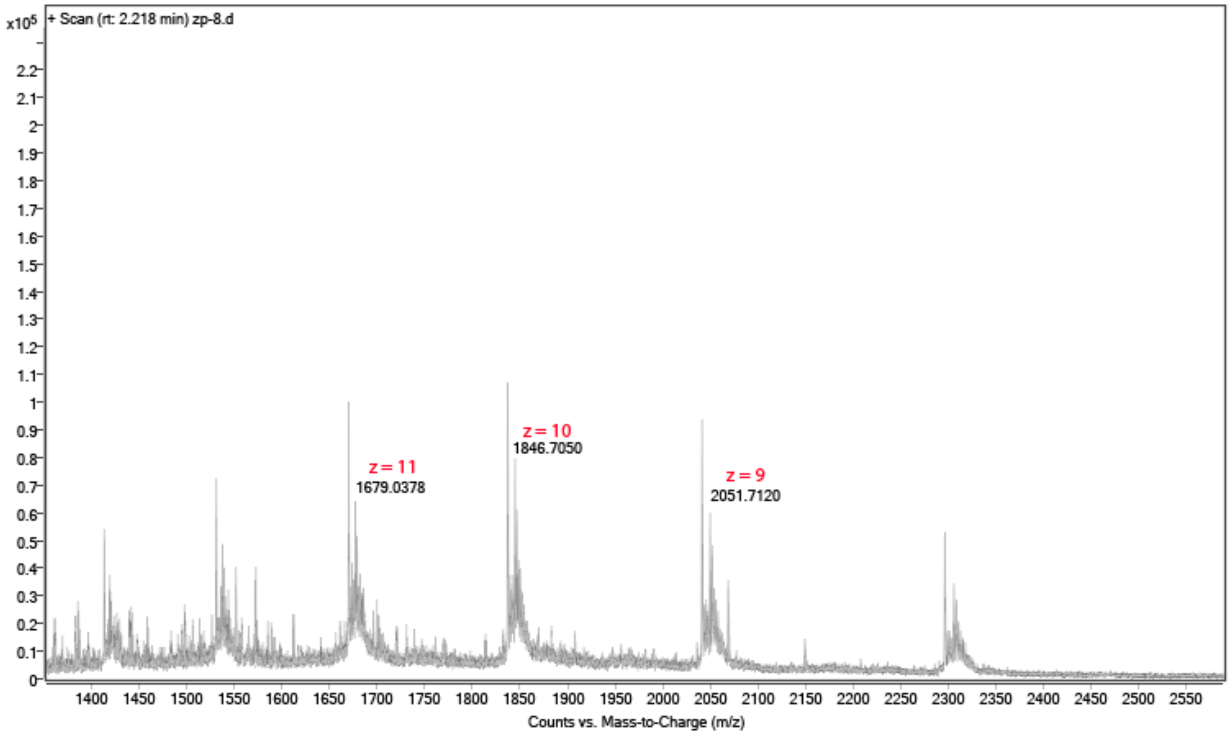

Deconvoluted MS of Modified β-lactoglobulin

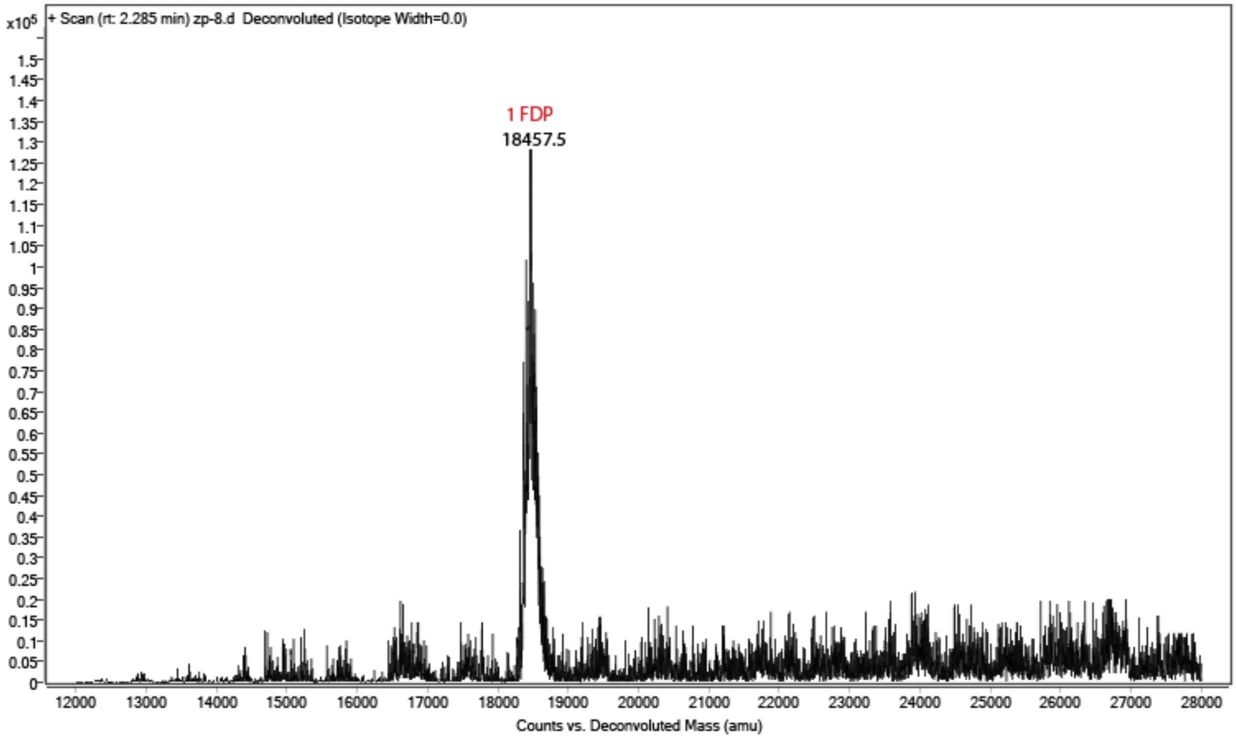

MSMS Analysis of Digested Modified β-lactoglobulin

Identified Peptide Fragment: KIDALNENKVLVLDTDYK: (Sequence: AA 99-116, K99 – MP Lysine)

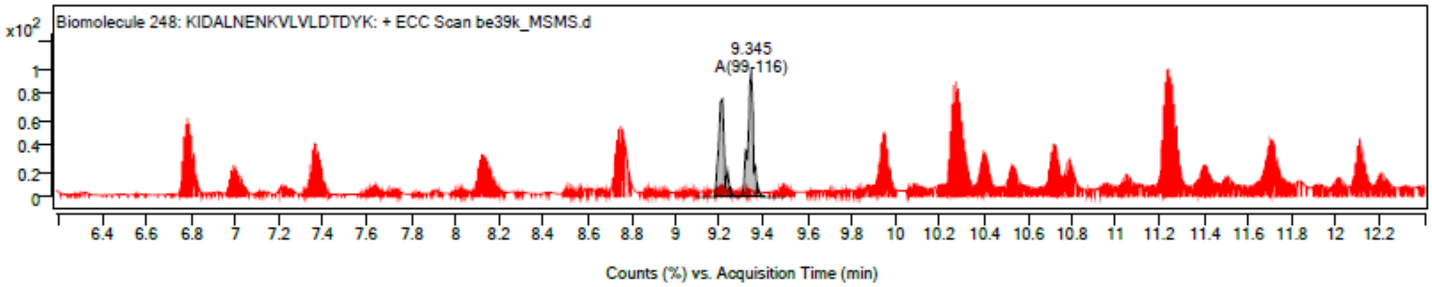

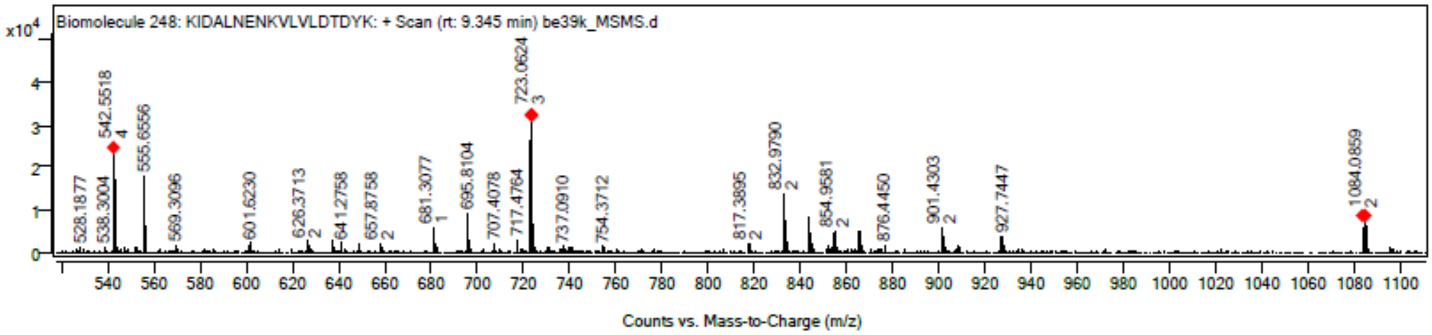

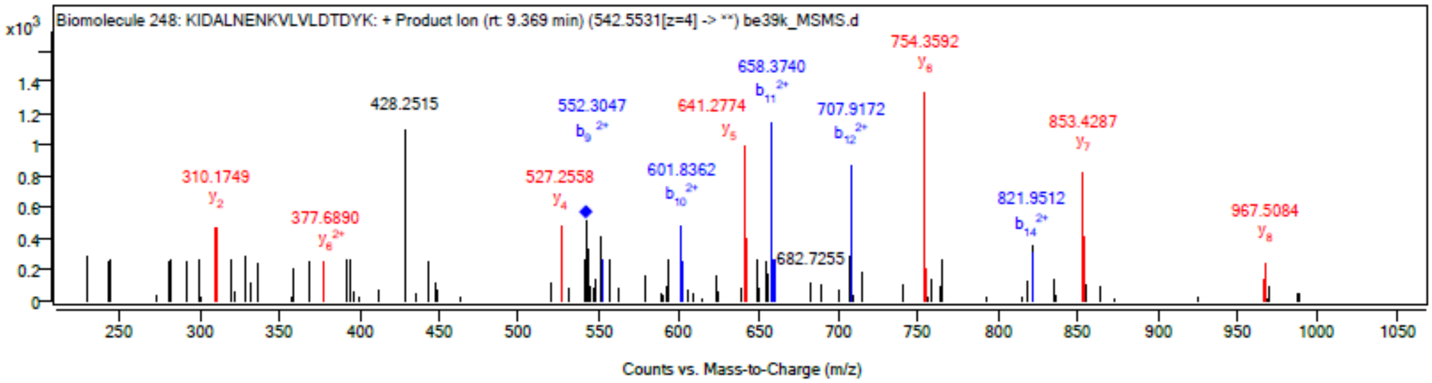

Fragment Spectrum Peaks

m/z	Diff (ppm)	Abund	Ion	Z
310.1749	4.13	468	y2	
527.2558	-3.22	477	y4	
641.2774	0.46	994	y5	
754.3592	3.37	1327	y6	
853.4287	1.68	823	y7	
967.5084	9.47	246	y8	
377.6890	-11.95	257	y6	
552.3047	-4.66	263	b9	
601.8362	0.19	477	b10	
658.3740	6.60	1137	b11	
707.9172	-6.51	868	b12	
821.9512	20.49	304	b14	

Identified Peptide Fragment: KYLLFCMENSAEPEQSLACQCLV: (Sequence: AA 117-139, K117 – FDP Lysine)

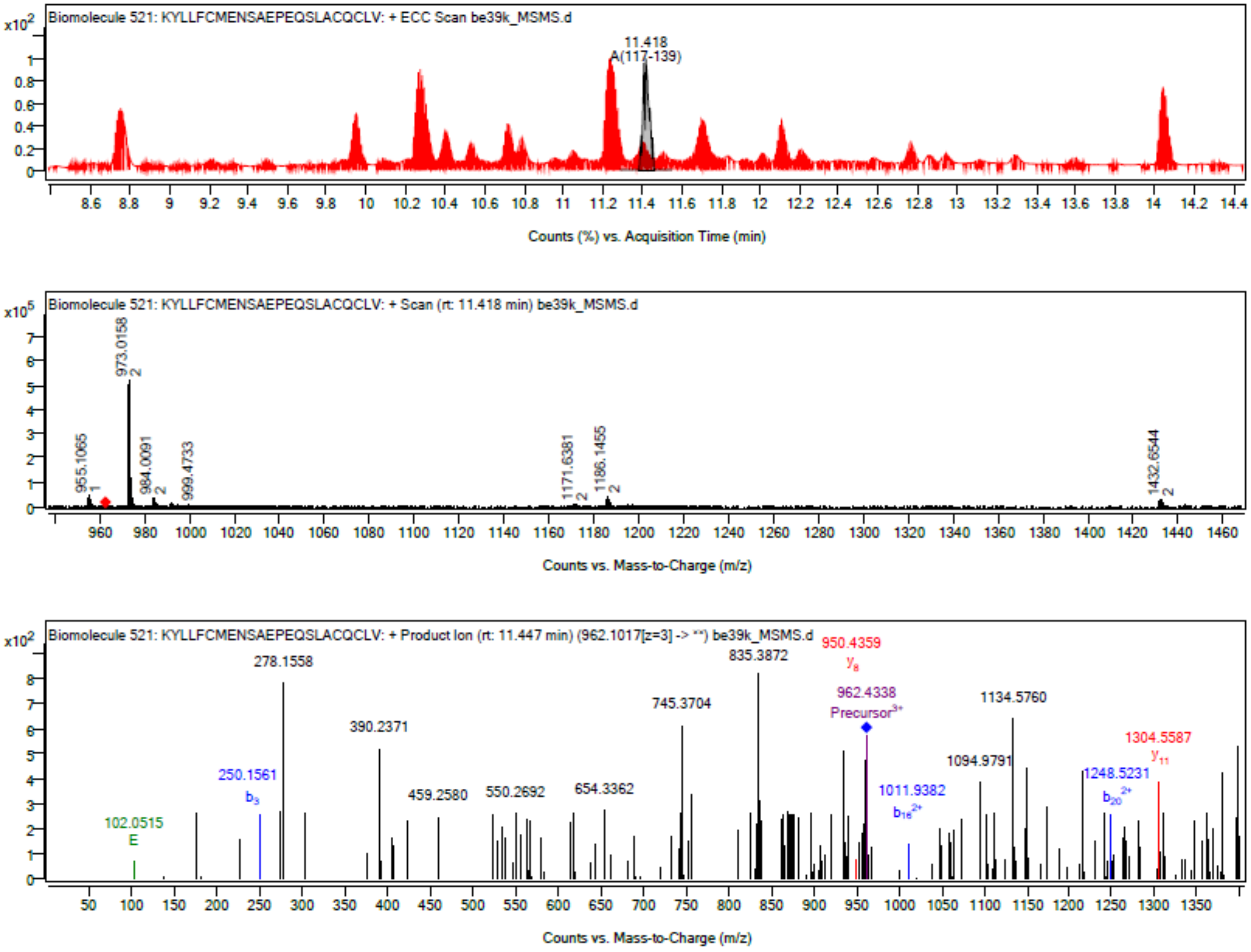

Fragment Spectrum Peaks

m/z	Diff (ppm)	Abund	Ion	Z
950.4359	7.85	77	y8	
1304.5587	29.64	385	y11	
250.1561	-26.97	255	b3	
1011.9382	16.11	137	b16	
1248.5231	30.66	258	b20	
102.0515	33.62	70	E	
962.4338	6.55	574	Precursor	

Identified Peptide Fragment: KYLLFCMENSAEPEQSLACQCLVR: (Sequence: AA 117-140, K117 – MP Lysine)

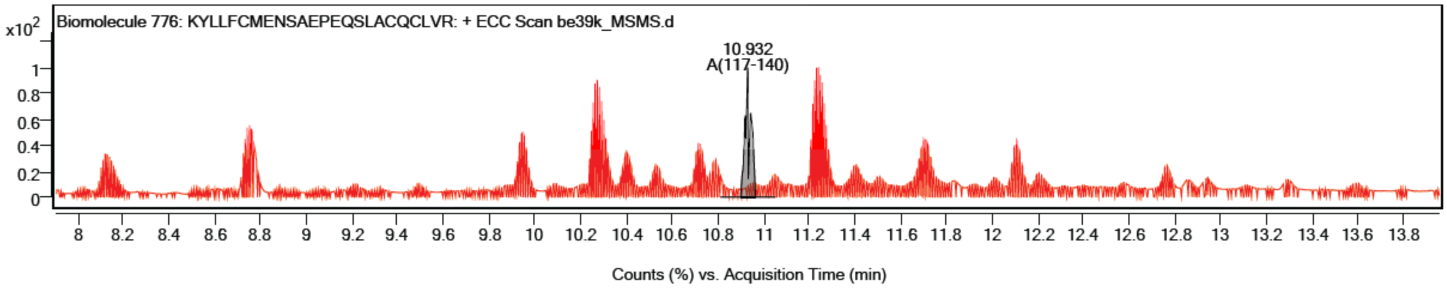

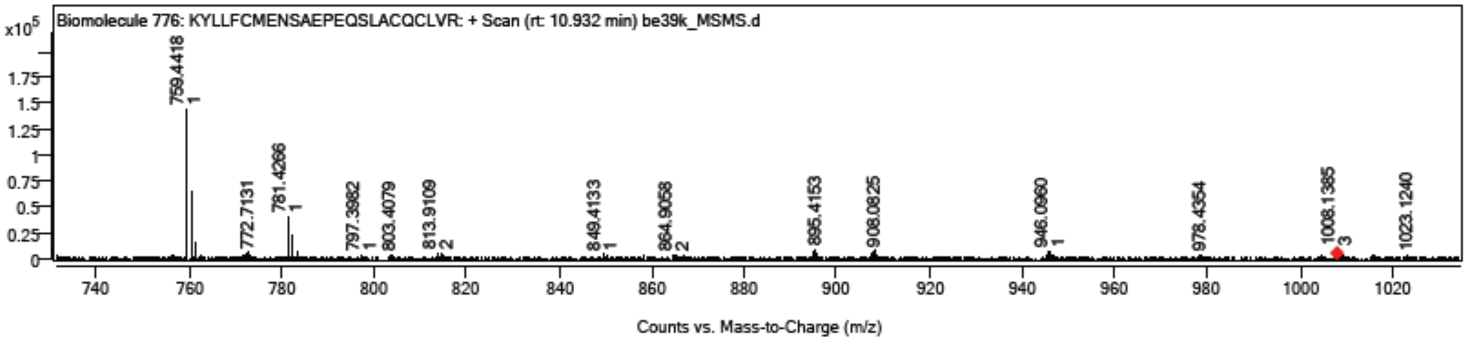

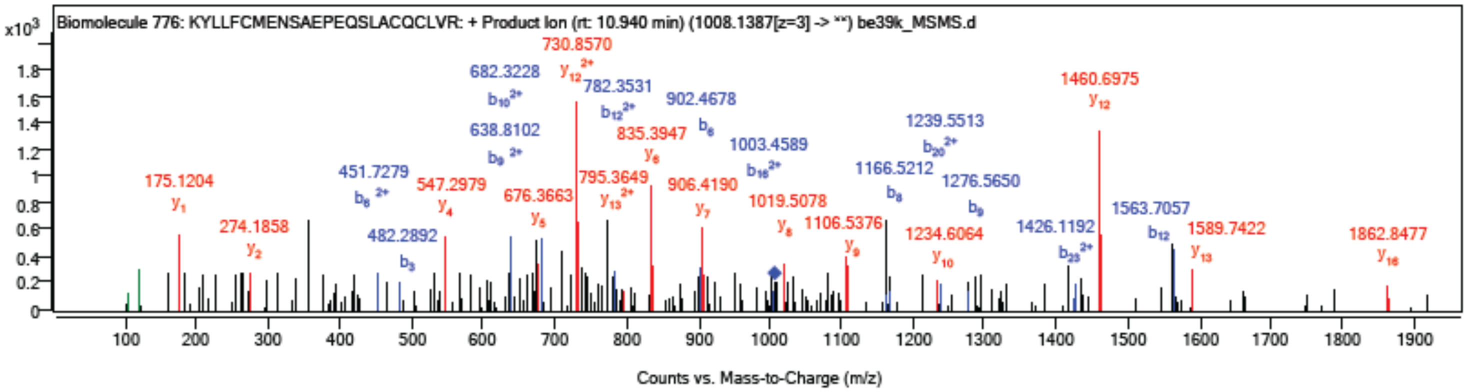

m/z	Diff (ppm)	Abund	Ion	Z
175.1204	-8.34	564	y1	
274.1858	5.79	264	y2	
547.2979	7.58	543	y4	
676.3663	-3.38	341	y5	
835.3947	-4.10	933	y6	
906.4190	10.39	609	y7	
1019.5078	4.58	344	y8	
1106.5376	6.28	392	y9	
1234.6064	-2.71	212	y10	
1460.6975	0.64	1348	y12	
1589.7422	-0.70	303	y13	
1862.8477	4.69	171	y16	
730.8570	-5.65	1564	y12	
795.3649	11.67	130	y13	
482.2892	-0.94	203	b3	
902.4678	4.51	304	b6	
1166.5212	40.41	135	b8	
1276.5650	25.80	137	b9	
1563.7057	2.49	451	b12	
451.7279	25.95	272	b6	
638.8102	-11.98	541	b9	
682.3228	-6.15	528	b10	
782.3531	6.80	283	b12	
1003.4589	-5.73	127	b16	
1239.5513	5.68	183	b20	
1426.1192	22.71	186	b23	
104.0509	18.35	120	M	
120.0825	-13.98	304	F	

Modification of Ribonuclease A

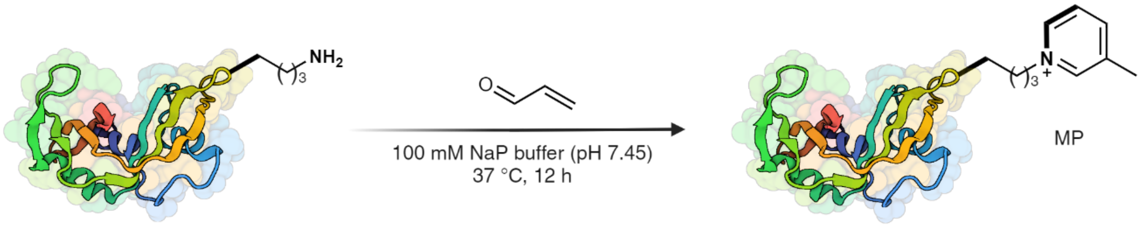

Ribonuclease A (2 mg, 0.292 mM) was dissolved in 480 µL of 100 mM NaP buffer (pH 7.45), and acrolein (0.078 µL, 2.34 mM) was added to the mixture from a freshly prepared stock solution (20 µL). The reaction was stirred at 37 °C for 12 hours, after which the crude reaction mixture was passed through Amicon Ultra 3 kDa spin-concentrator and washed with H_2O (5 x 500 µL) to remove the small molecule impurities. The labeled protein was redissolved in 0.1% formic acid in H_2O and analyzed using LC-MS. The conversion was found to be >95% with full conversion to 1 MP modification.

Modification	Mass	Conversion
Unmodified	13682.7	N/A
1 MP	13757.1 (**+76**)	>95%

Intact MS of Starting Ribonuclease A

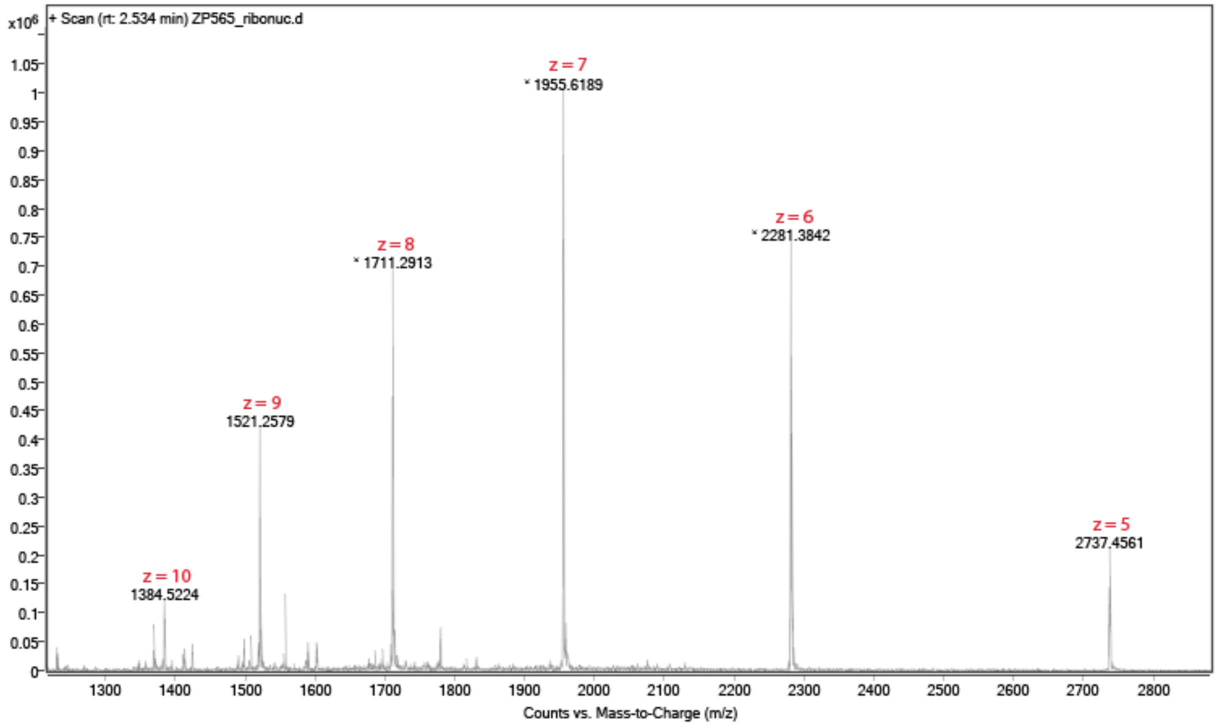

Deconvoluted MS of Starting Ribonuclease A

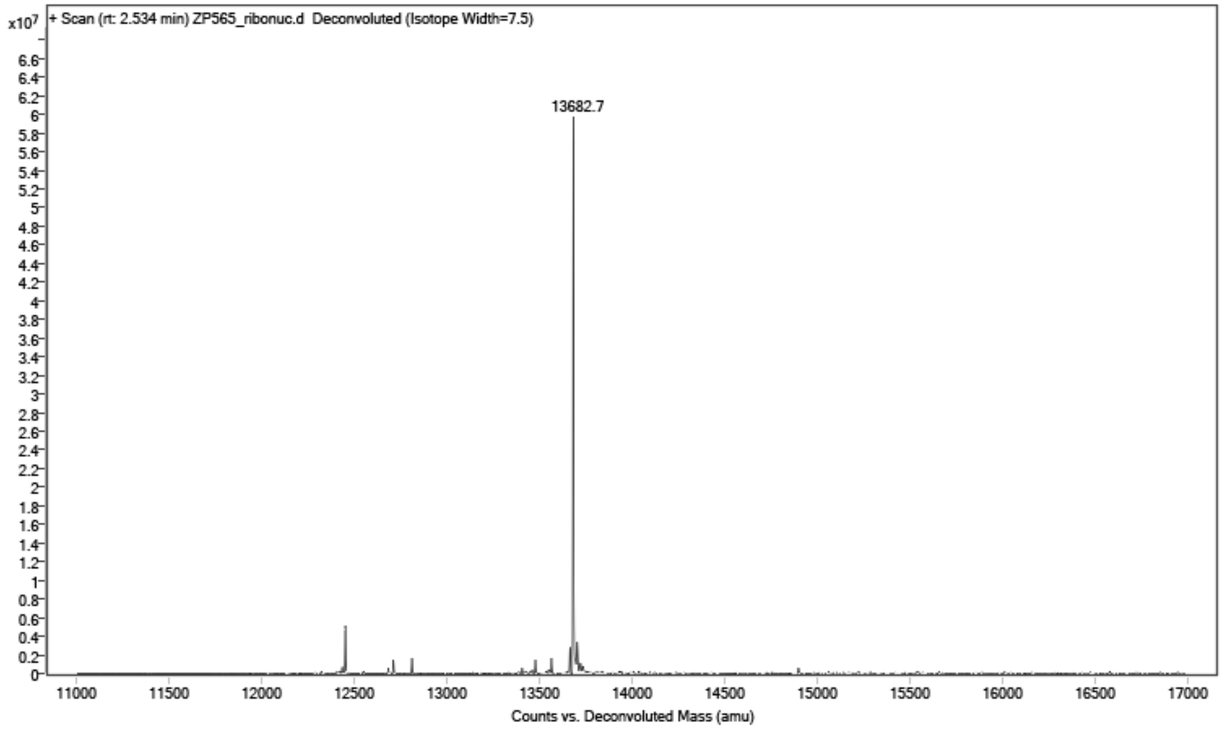

Intact MS of Modified Ribonuclease A

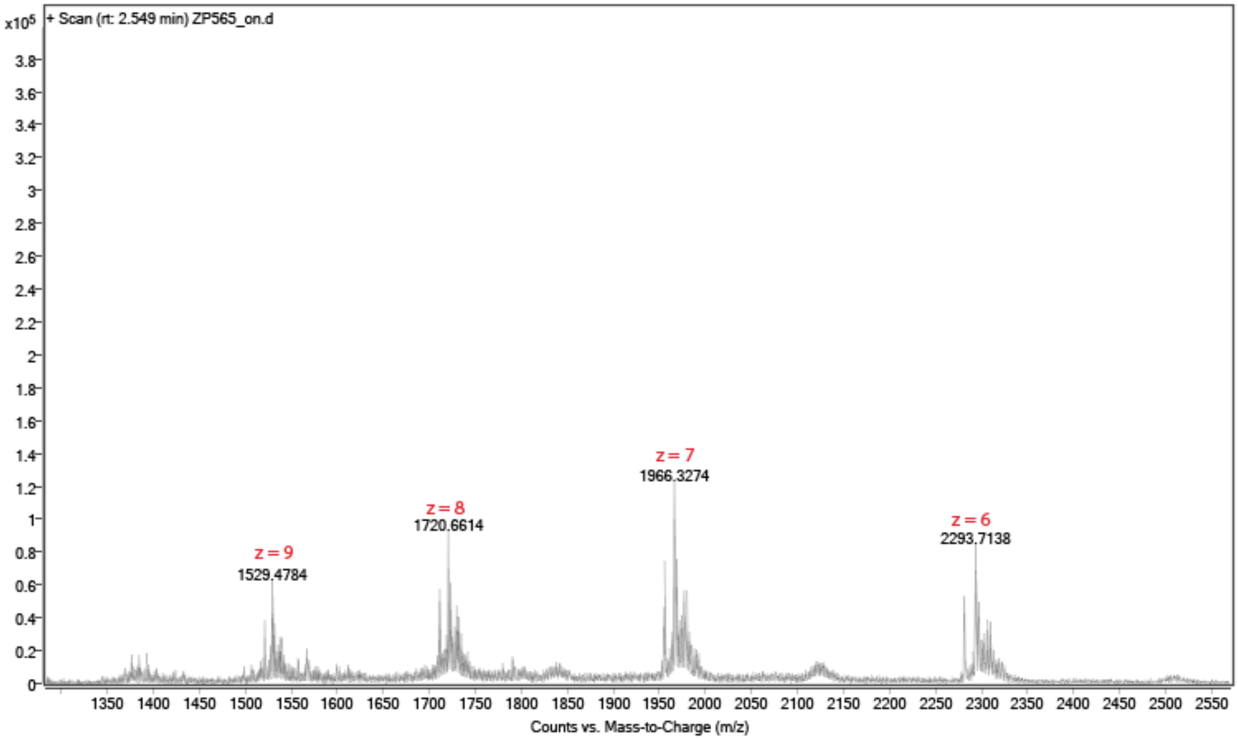

Deconvoluted MS of Modified Ribonuclease A

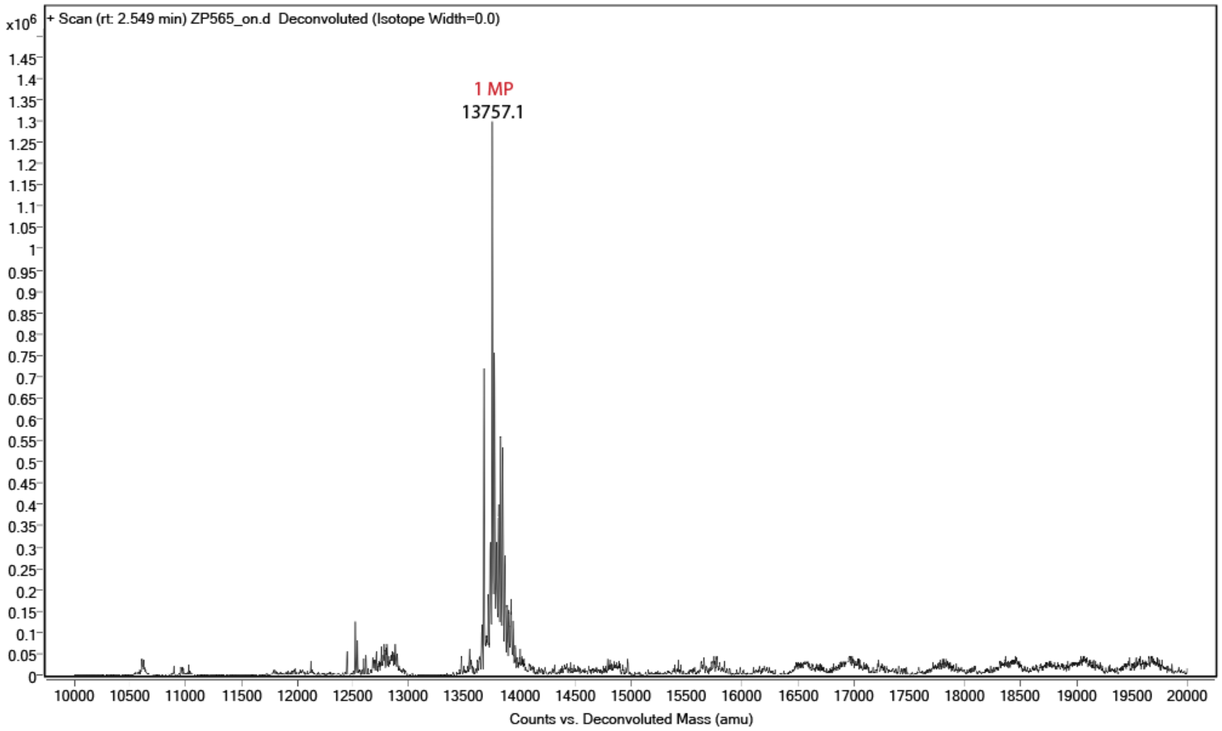

Modification of Cytochrome C

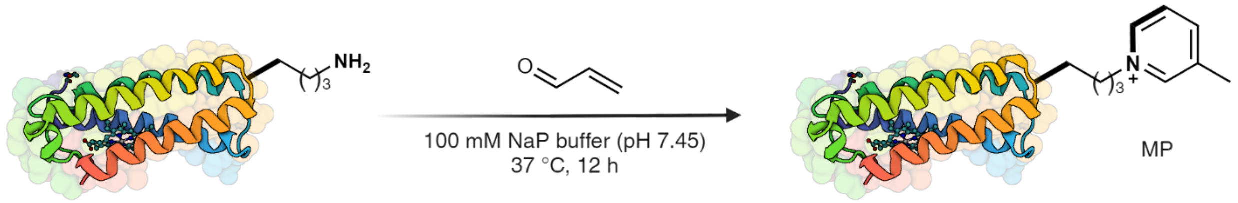

Cytochrome C (2 mg, 0.324 mM) was dissolved in 480 µL of 100 mM NaP buffer (pH 7.45), and acrolein (0.086 µL, 2.59 mM) was added to the mixture from a freshly prepared stock solution (20 µL). The reaction was stirred at 37 °C for 12 hours, after which the crude reaction mixture was passed through Amicon Ultra 3 kDa spin-concentrator and washed with H_2O (5 x 500 µL) to remove the small molecule impurities. The labeled protein was redissolved in 0.1% formic acid in H_2O and analyzed using LC-MS. The conversion was found to be >95% with full conversion to 1 MP modification.

Modification	Mass	Conversion
Unmodified	12359.5	N/A
1 MP	12436.4 (+**76**)	>95%

Intact MS of Starting Cytochrome C

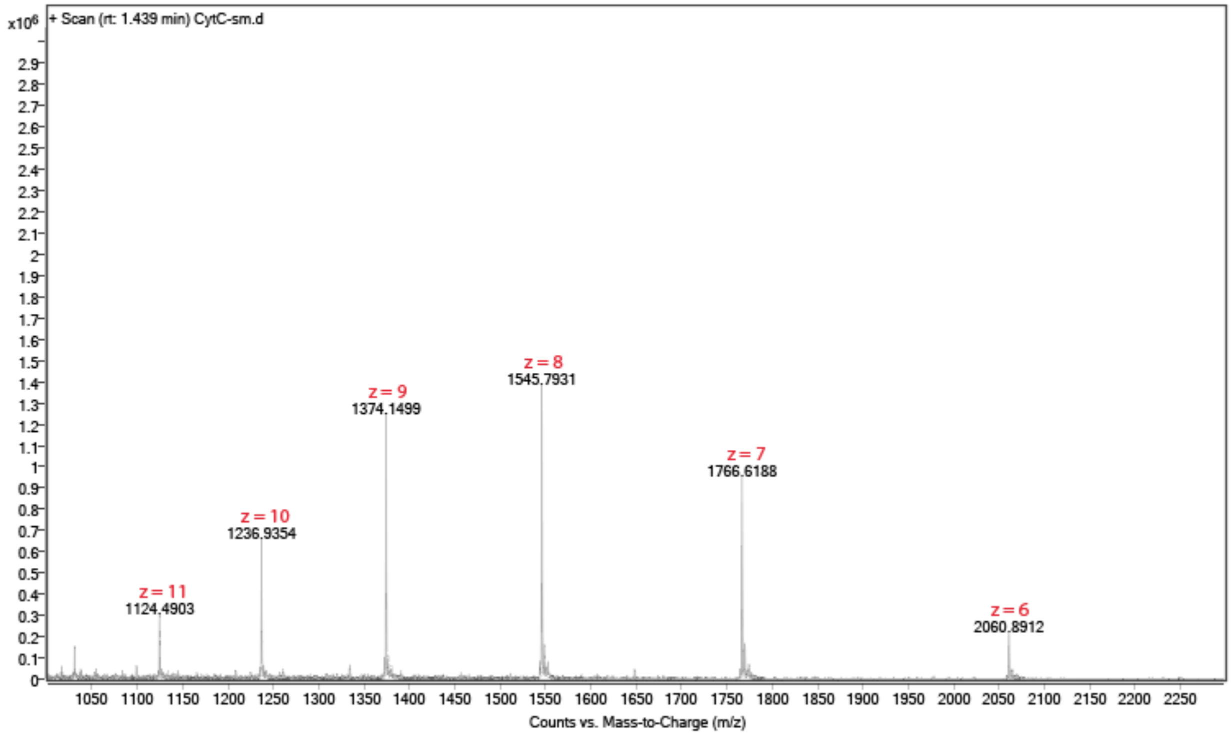

Deconvoluted MS of Starting Cytochrome C

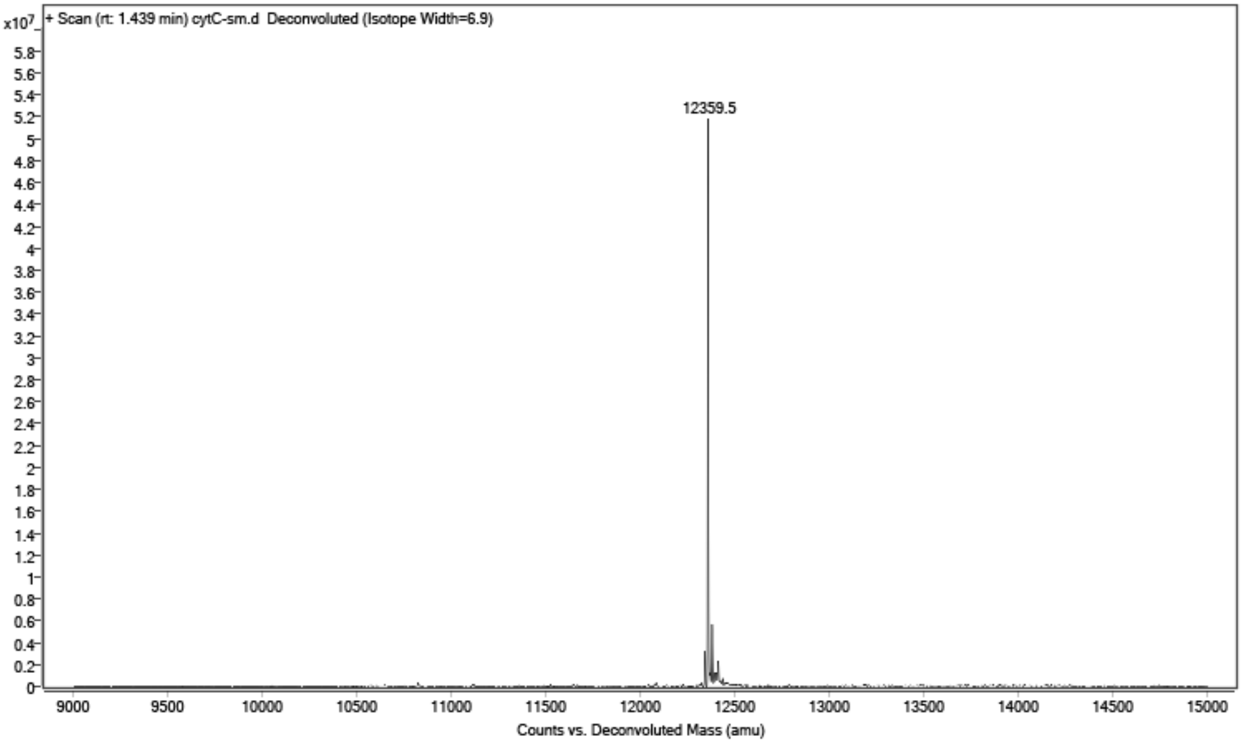

Intact MS of Modified Cytochrome C

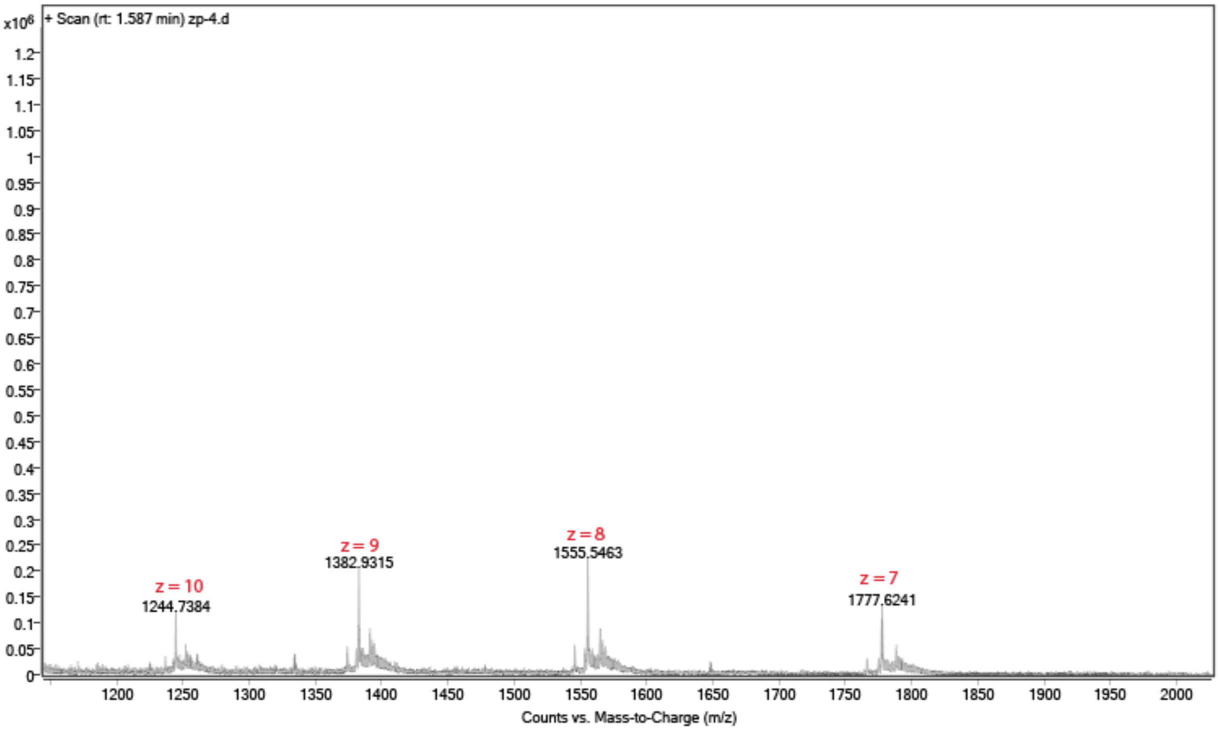

Deconvoluted MS of Modified Cytochrome C

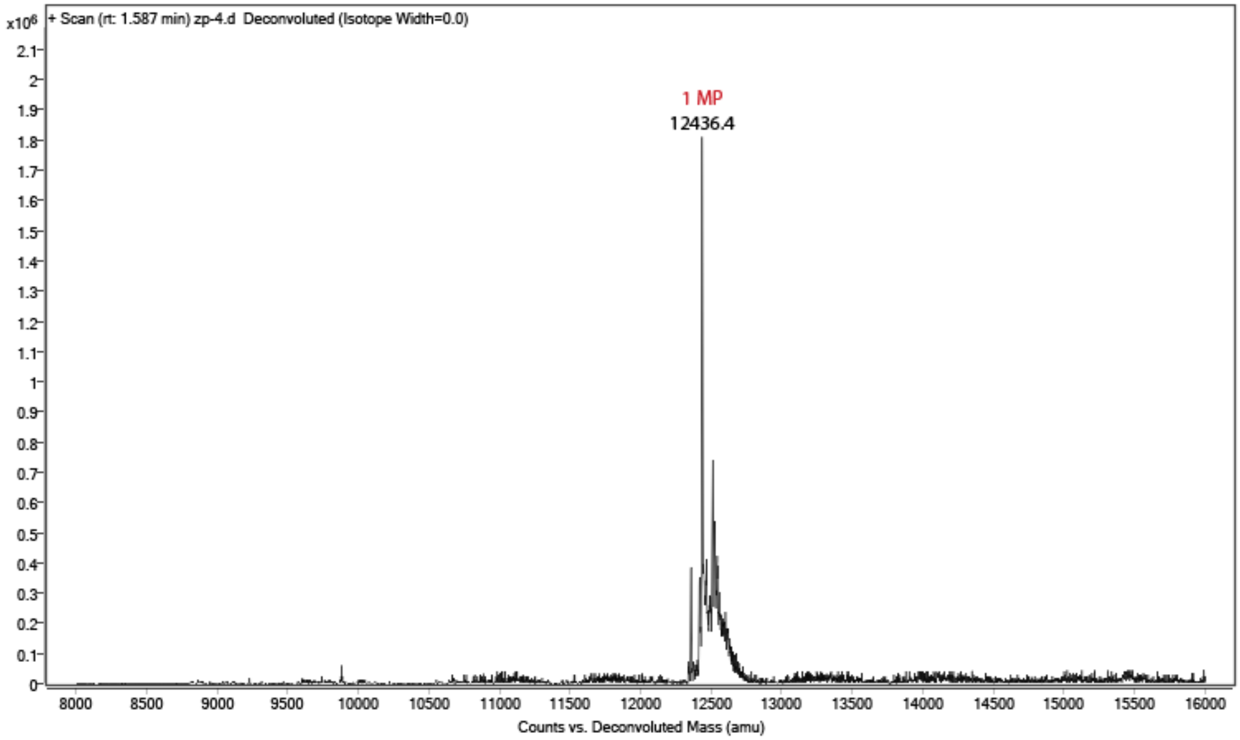

MSMS Analysis of Digested Modified Cytochrome C

Identified Peptide Fragment: KTGQAPGFTYTDANK: (Sequence: AA 39-53, K39 – MP Lysine)

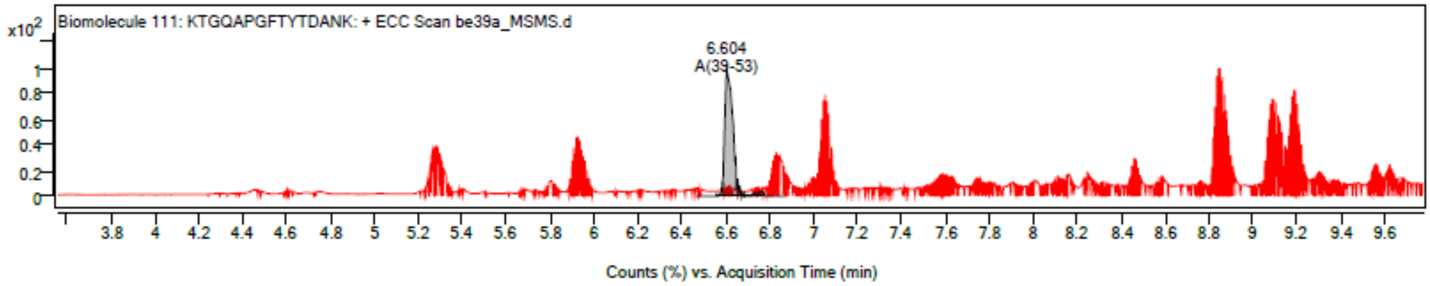

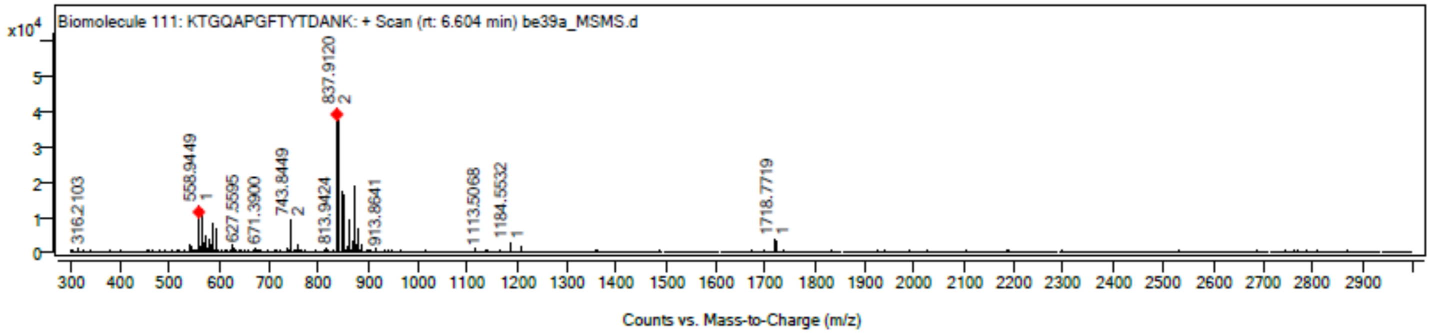

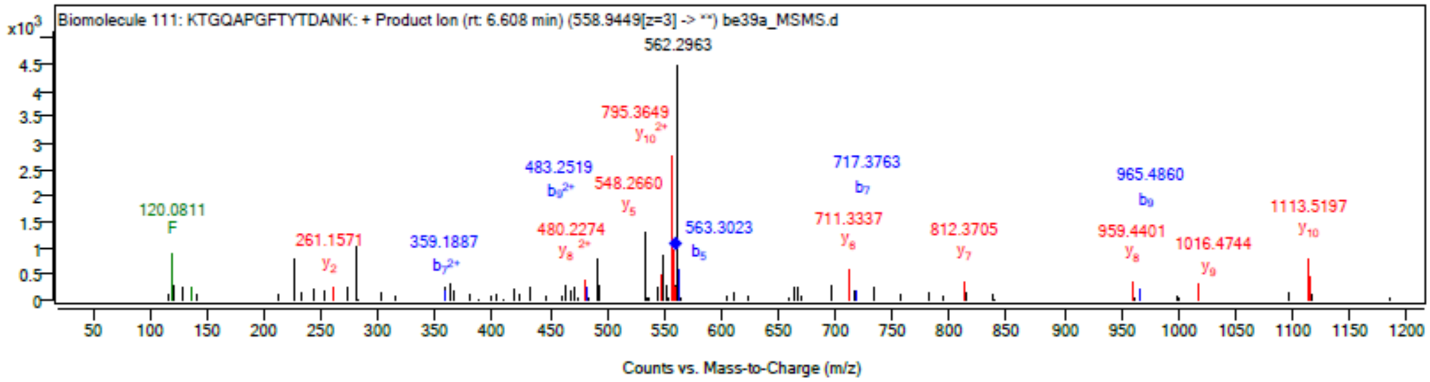

Fragment Spectrum Peaks

m/z	Diff (ppm)	Abund	Ion	Z
559.2787	-13.88	256	Precursor	
261.1571	-5.16	257	y2	
548.2660	2.64	491	y5	
711.3337	-4.12	575	y6	
812.3705	9.86	359	y7	
959.4401	7.10	346	y8	
1016.4744	-5.90	299	y9	
1113.5197	1.24	798	y10	
480.2274	-0.66	374	y8	
557.2648	-1.02	2736	y10	
563.3023	7.00	575	b5	
717.3763	5.69	194	b7	
965.4860	10.94	210	b9	
359.1887	14.21	165	b7	
483.2519	-0.03	257	b9	
120.0811	-2.40	901	F	
136.0792	-25.50	262	Y	

Identified Peptide Fragment: KCAQCHTVEKGGK: (Sequence: AA 13-25, K13 – FDP Lysine)

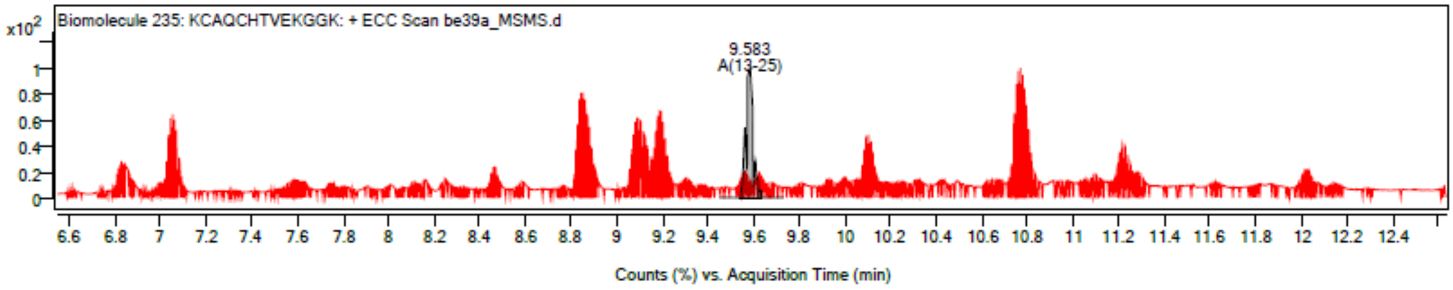

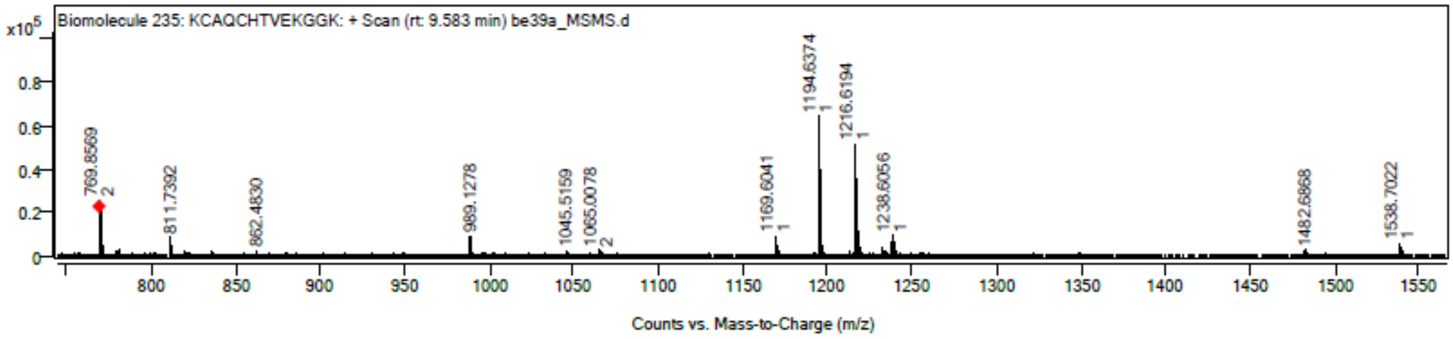

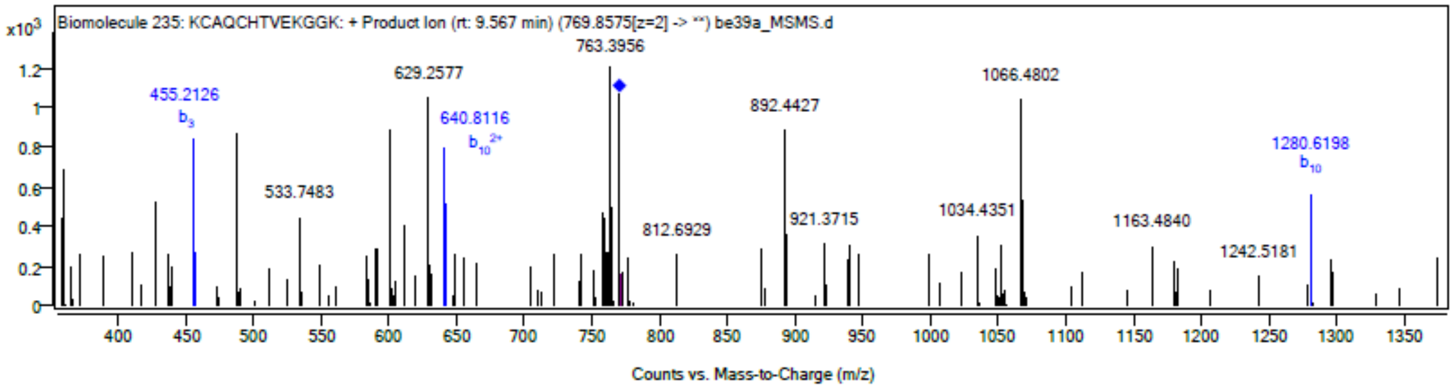

Fragment Spectrum Peaks

m/z	Diff (ppm)	Abund	Ion	Z
455.2126	-36.70	843	b3	
1280.6198	-34.06	560	b10	
640.8116	-31.06	796	b10	
770.8681	-2.83	161	Precursor	

Modification of Insulin

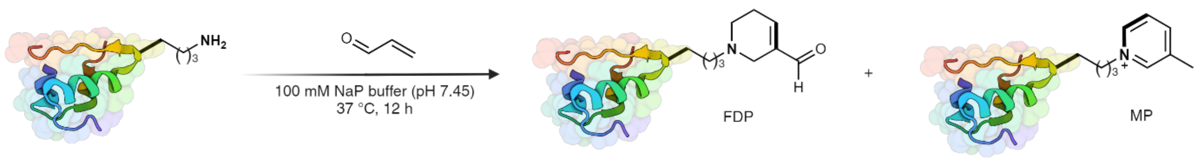

Insulin (2 mg, 0.689 mM) was dissolved in 480 µL of 100 mM NaP buffer (pH 7.45), and acrolein (0.184 µL, 5.51 mM) was added to the mixture from a freshly prepared stock solution (20 µL). The reaction was stirred at 37 °C for 12 hours, after which the crude reaction mixture was passed through Amicon Ultra 3 kDa spin-concentrator and washed with H_2O (5 x 500 µL) to remove the small molecule impurities. The labeled protein was redissolved in 0.1% formic acid in H_2O and analyzed using LC-MS. The conversion was found to be >95% with full conversion to a homogeneous product with 1 FDP and 1 MP modification.

Modification	Mass	Conversion
Unmodified	5807.7	N/A
1 FDP + 1 MP	5977.3 (+170)	>95%

Intact MS of Starting Insulin

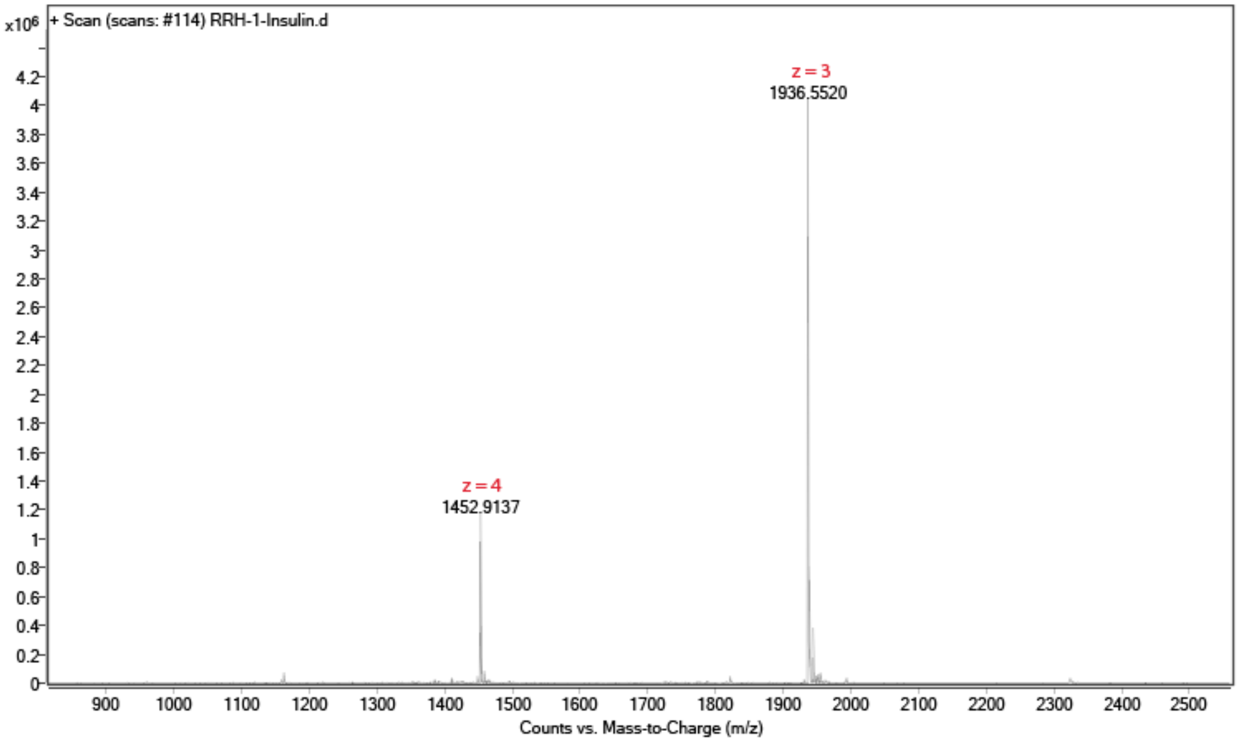

Deconvoluted MS of Starting Insulin

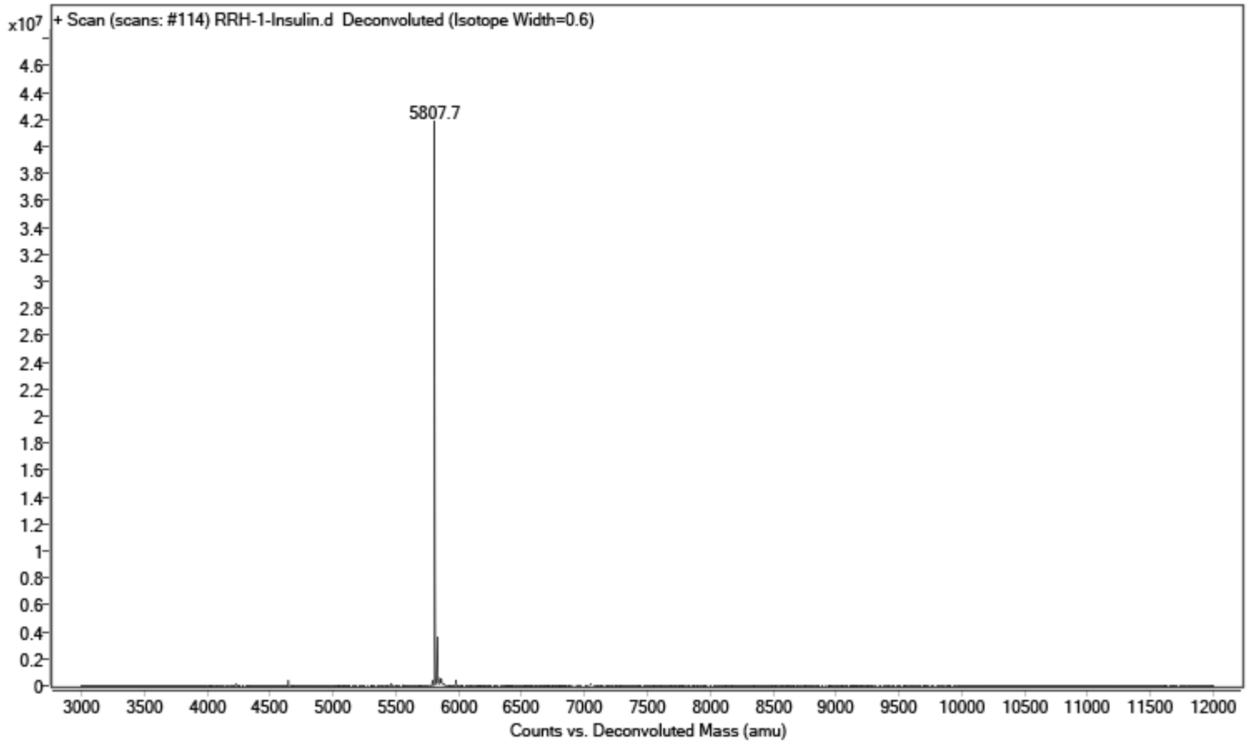

Intact MS of Modified Insulin

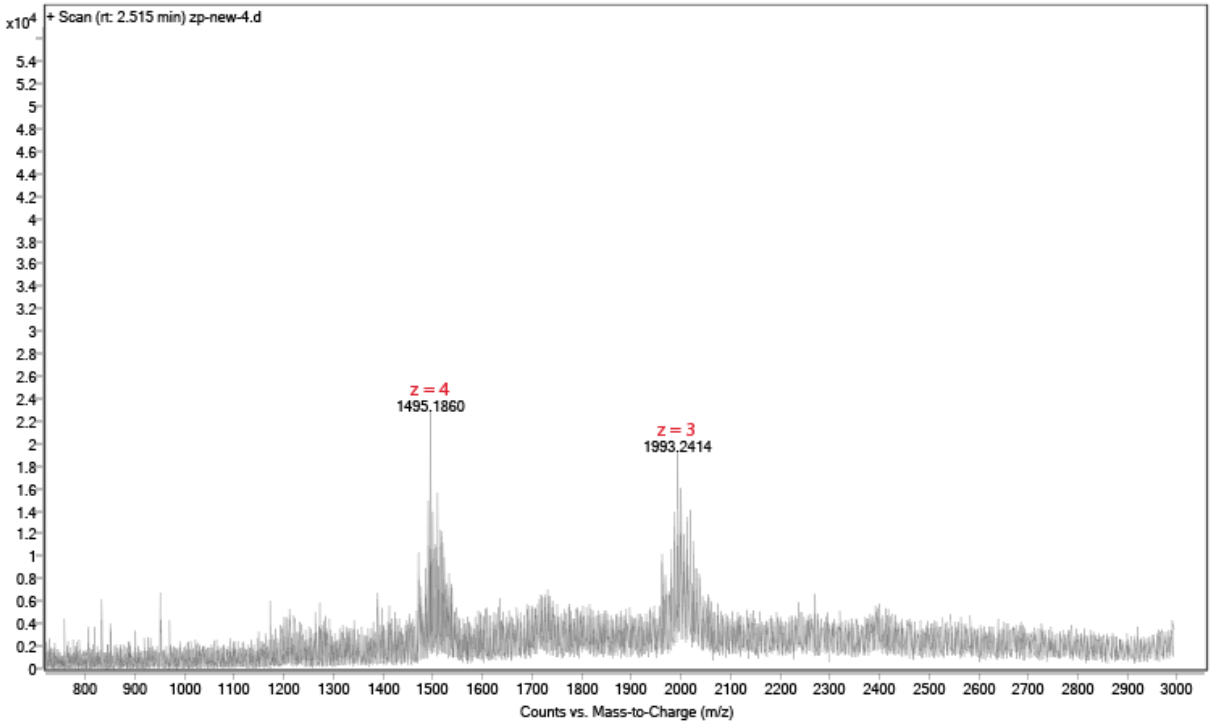

Deconvoluted MS of Modified Insulin

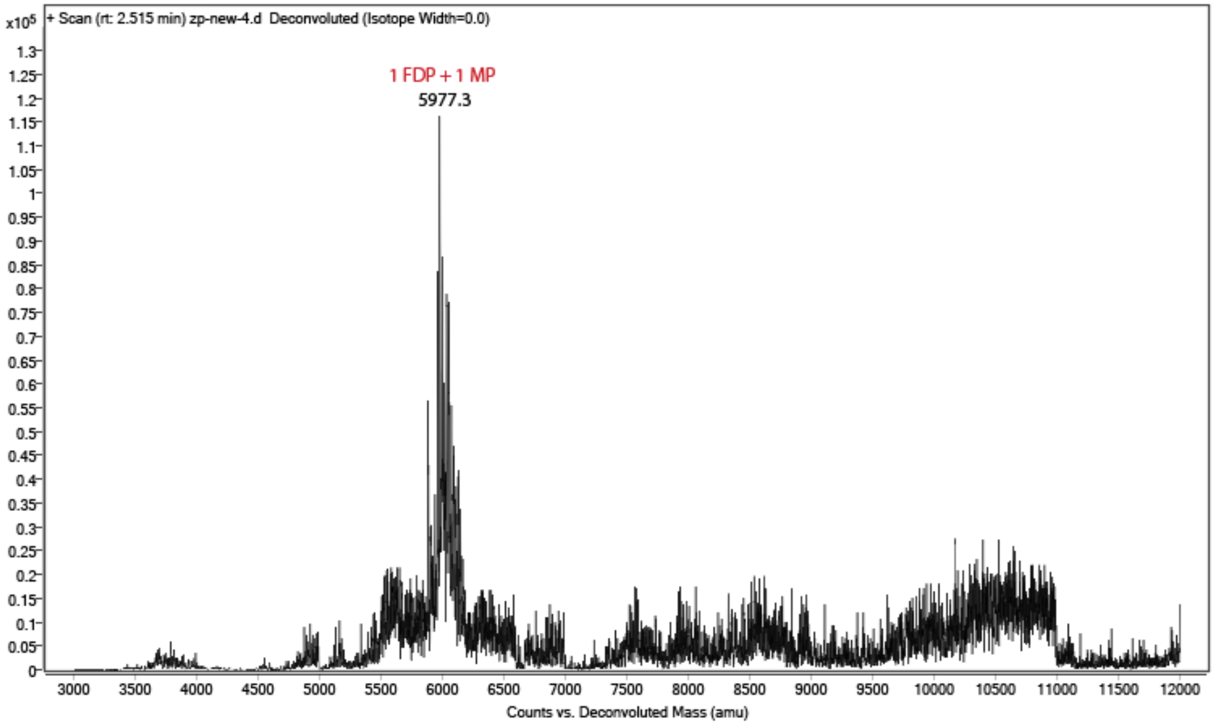

Modification of Apo-Transferrin Bovine

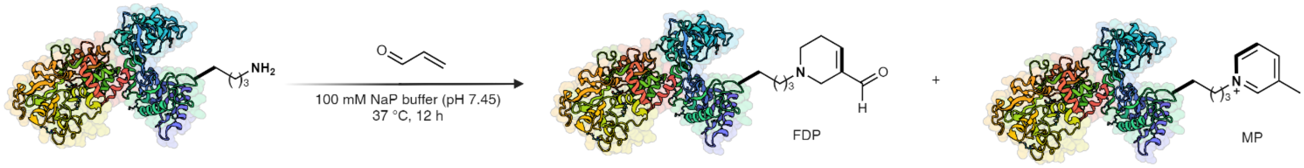

Apo-Transferrin bovine (2 mg, 0.050 mM) was dissolved in 480 µL of 100 mM NaP buffer (pH 7.45), and acrolein (0.013 µL, 0.754 mM) was added to the mixture from a freshly prepared stock solution (20 µL). The reaction was stirred at 37 °C for 12 hours, after which the crude reaction mixture was passed through Amicon Ultra 3 kDa spin-concentrator and washed with H_2O (5 x 500 µL) to remove the small molecule impurities. The labeled protein was redissolved in 0.1% formic acid in H_2O and analyzed using LC-MSMS to determine sites of FDP and MP modification.

MSMS Analysis of Digested Modified Apo-Transferrin Bovine

Identified Peptide Fragment: KCGLVPVLAENYKTEGESCK: (Sequence: AA 423-442, K423 – MP Lysine)

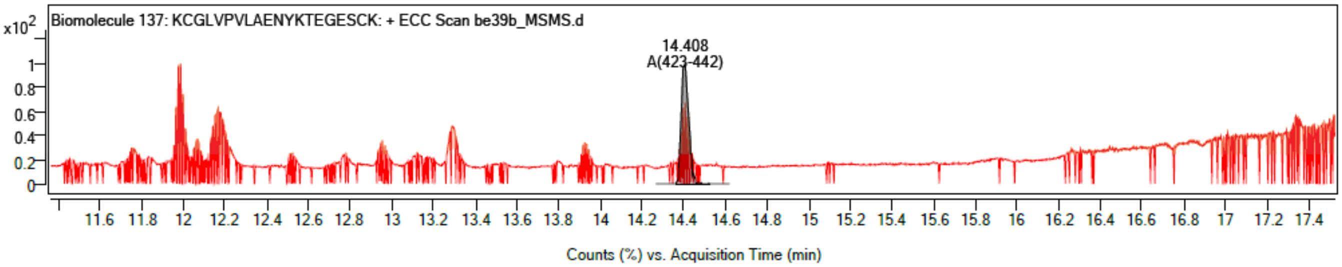

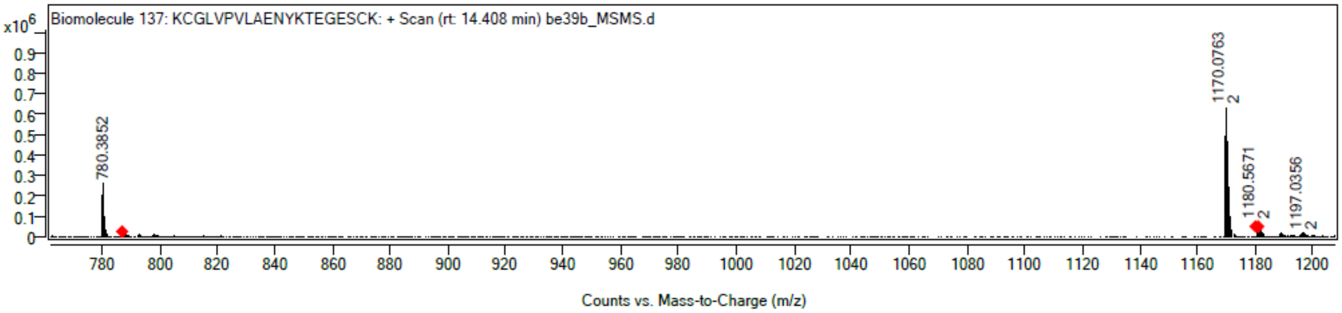

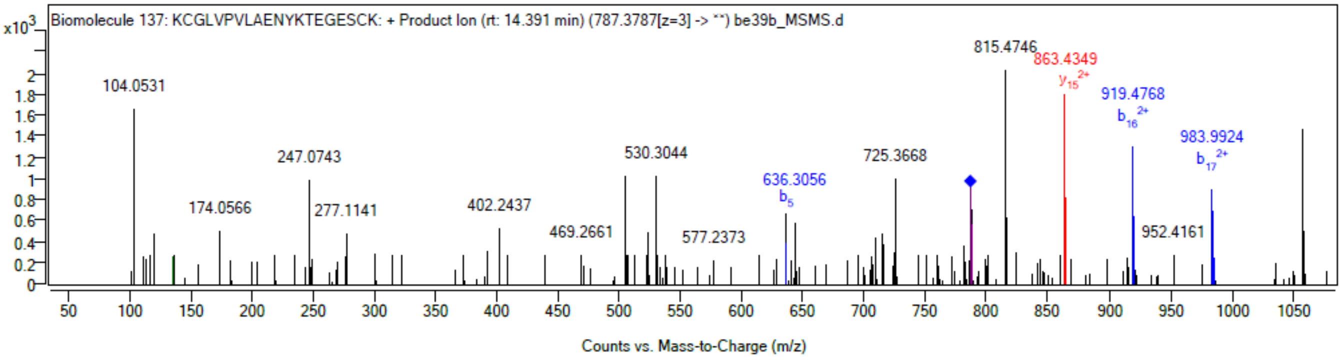

Fragment Spectrum Peaks

m/z	Diff (ppm)	Abund	Ion	Z
863.4349	-25.05	1790	y15	
636.3056	38.36	375	b5	
919.4768	-9.99	1292	b16	
983.9924	-3.62	884	b17	
136.0745	8.92	255	Y	
787.3779	8.23	909	Precursor	

Identified Peptide Fragment: KTYDSYLGDDYVR: (Sequence: AA 670-682, K670 – MP Lysine)

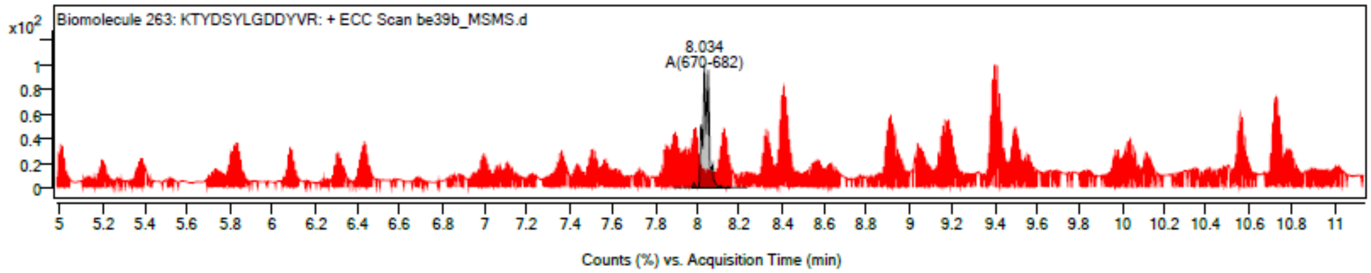

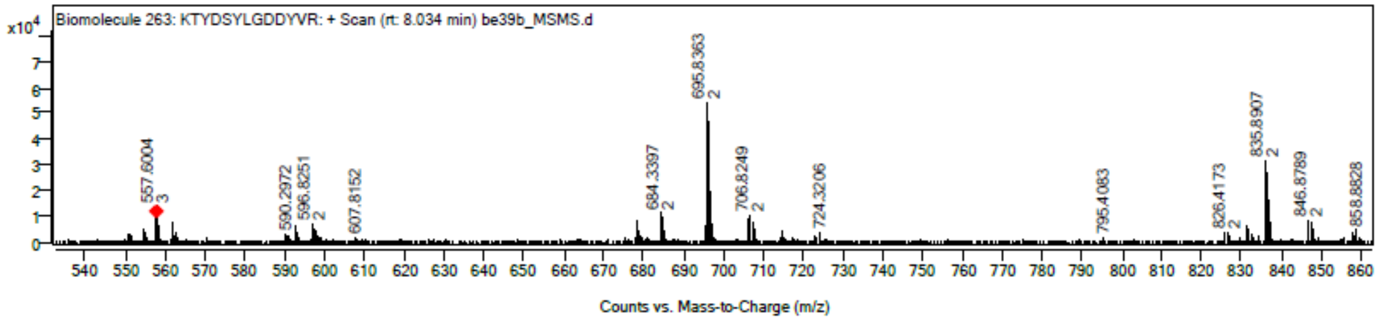

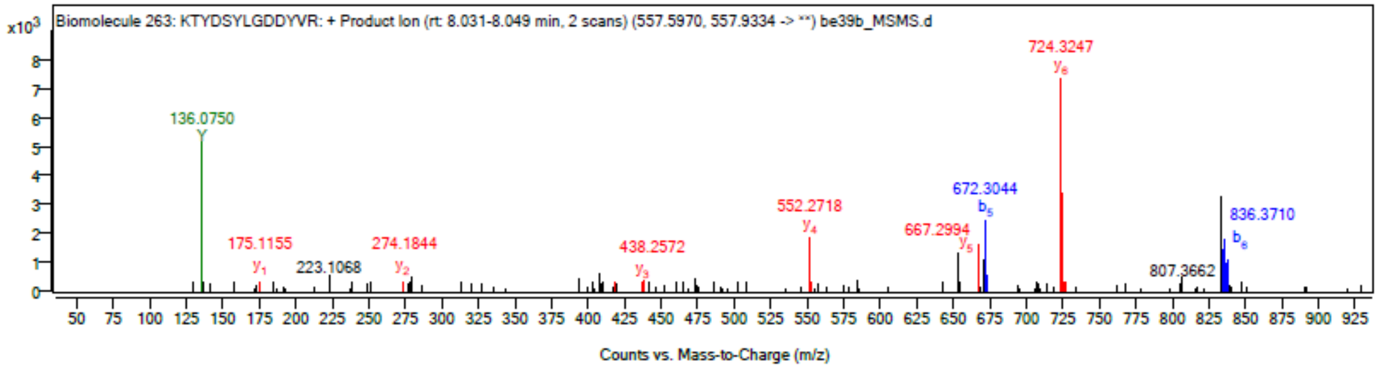

Fragment Spectrum Peaks

m/z	Diff (ppm)	Abund	Ion	Z
175.1155	19.44	298	y1	
274.1844	10.82	265	y2	
438.2572	-7.17	344	y3	
552.2718	10.49	1828	y4	
667.2994	7.74	1546	y5	
724.3247	1.92	7335	y6	
419.2114	-6.47	266	y7	
672.3044	10.29	2416	b5	
836.3710	8.45	1736	b6	
136.0750	4.84	5110	Y	

Identified Peptide Fragment: KNYELLCGDNTR: (Sequence: AA 244-255, K244 – MP Lysine)

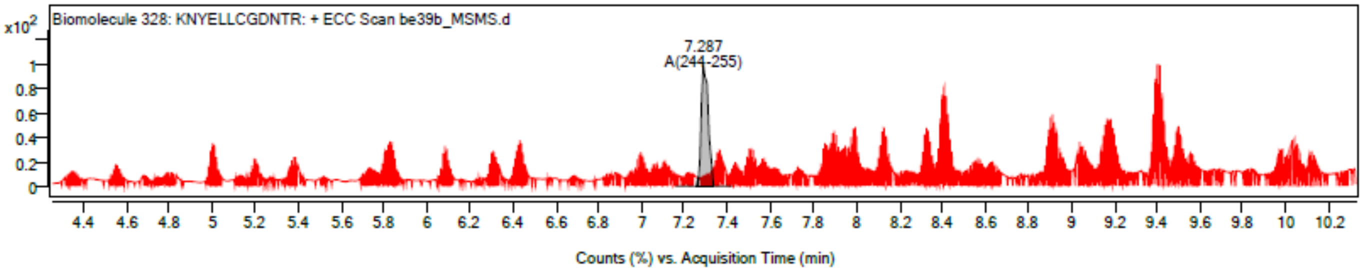

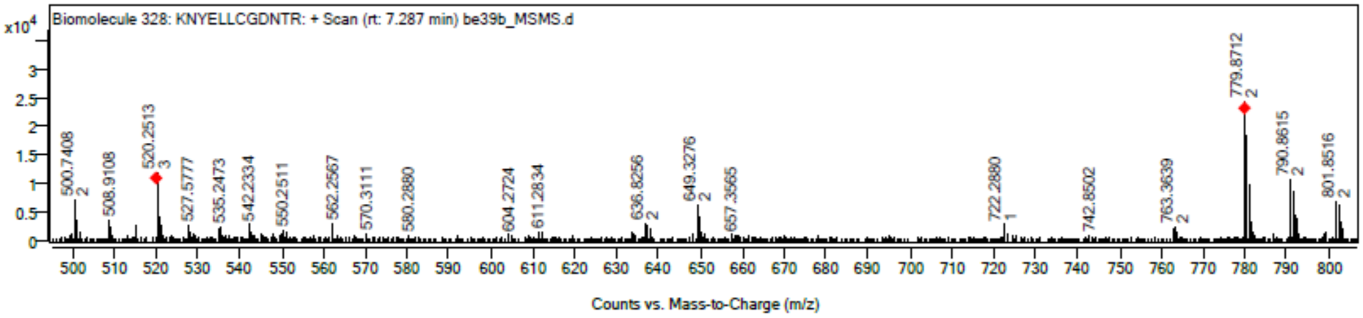

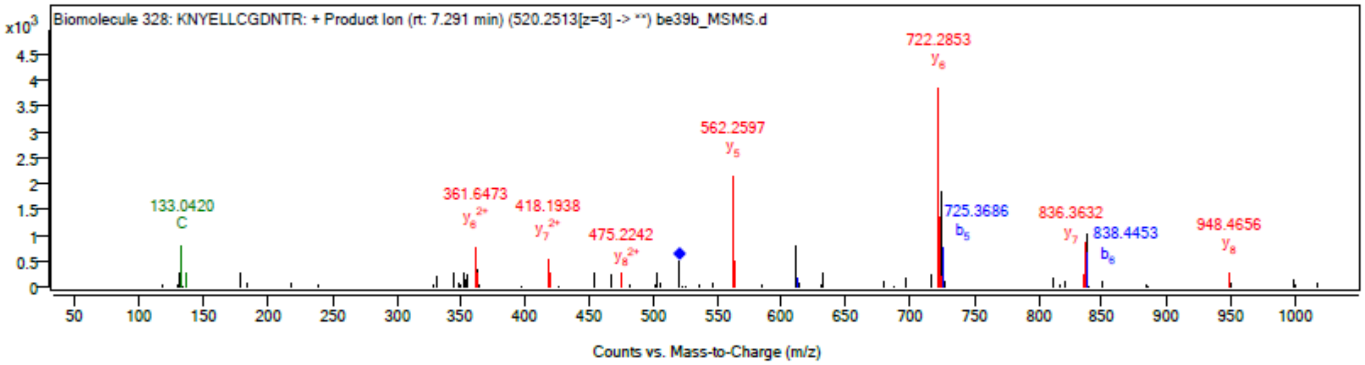

Fragment Spectrum Peaks

m/z	Diff (ppm)	Abund	Ion	Z
562.2597	-3.03	2114	y5	
722.2853	4.65	3813	y6	
836.3632	15.35	857	y7	
948.4656	-9.37	255	y8	
361.6473	1.73	743	y6	
418.1938	-9.15	522	y7	
475.2242	19.97	258	y8	
612.2871	5.09	155	b4	
725.3686	7.89	751	b5	
838.4453	15.62	653	b6	
133.0420	7.53	777	C	
136.0756	0.50	260	Y	

Identified Peptide Fragment: KSCHTAVDRTAGWNIPMGLL: (Sequence: AA 471-490, K471 – FDP Lysine)

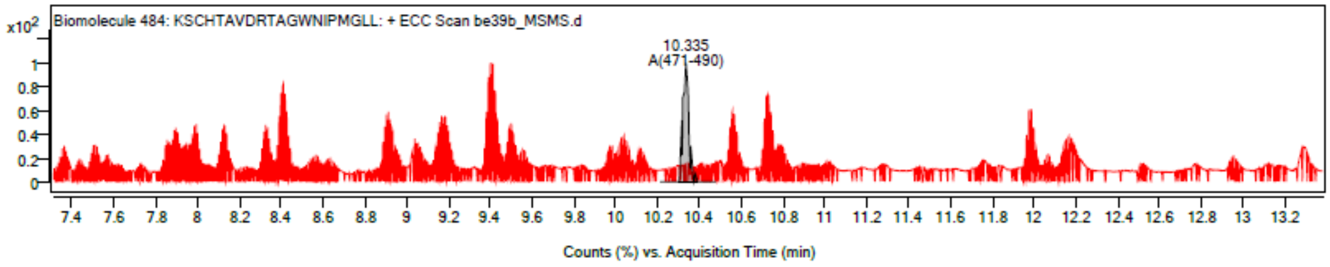

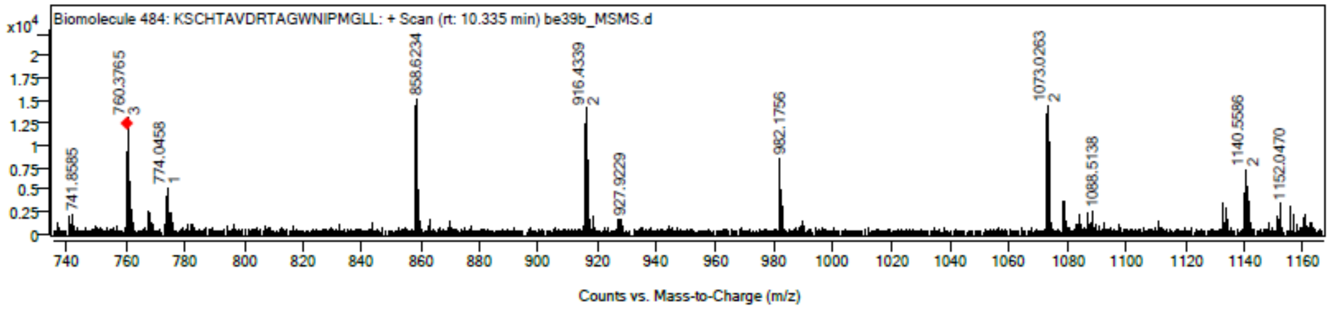

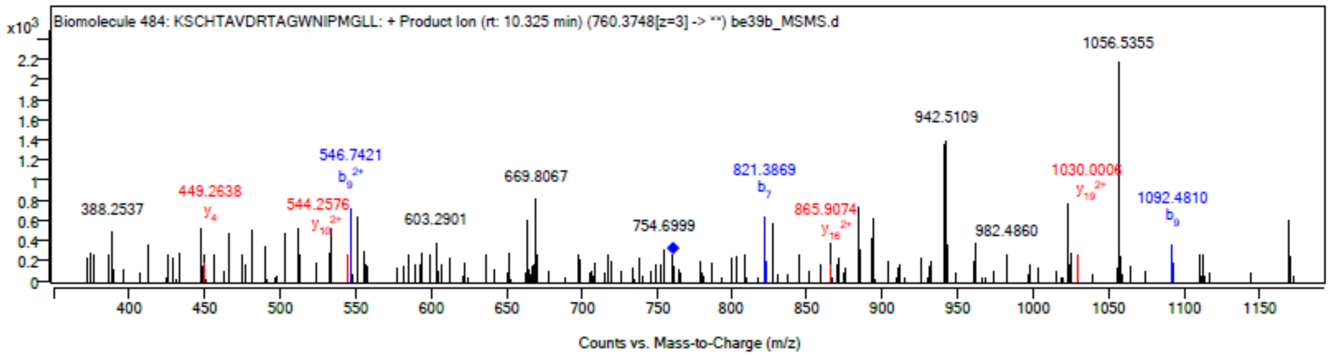

Fragment Spectrum Peaks

m/z	Diff (ppm)	Abund	Ion	Z
449.2638	-46.65	177	y4	
544.2576	48.23	255	y10	
865.9074	47.24	163	y16	
1030.0006	-0.46	257	y19	
821.3869	12.86	626	b7	
1092.4810	40.71	346	b9	
546.7421	44.41	721	b9	

Identified Peptide Fragment: KPVTDAENCHLARG: (Sequence: AA 595-608, K595 – MP Lysine)

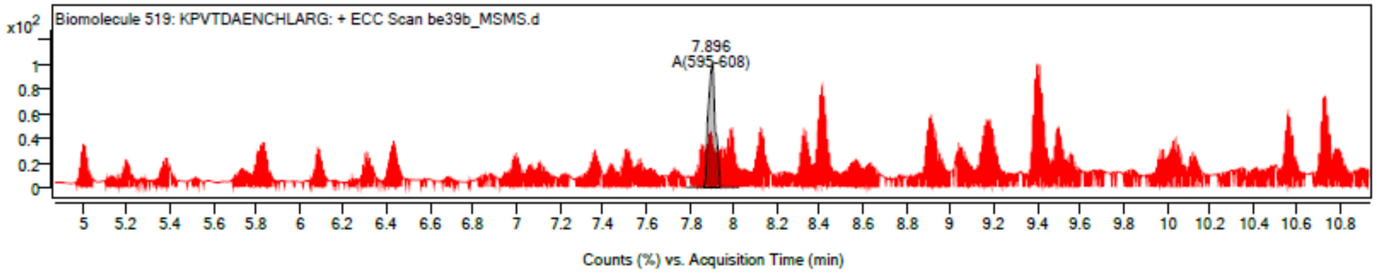

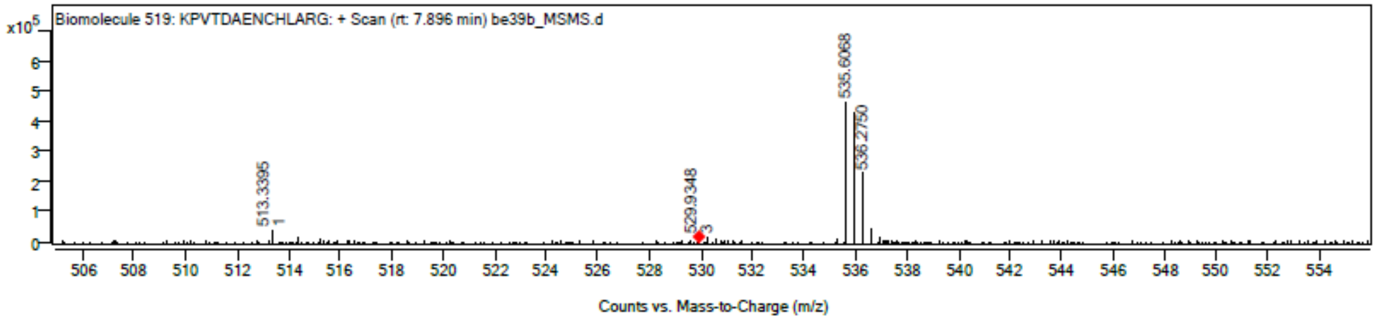

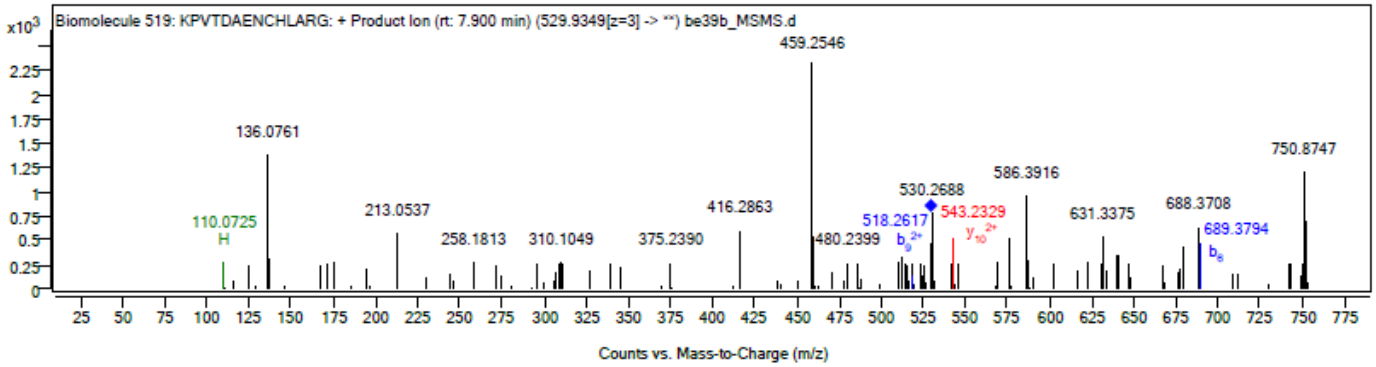

Fragment Spectrum Peaks

m/z	Diff (ppm)	Abund	Ion	Z
543.2329	19.03	519	y10	
689.3794	-7.42	473	b6	
518.2617	-45.52	126	b9	
110.0725	-11.04	268	H	

Fig. S17 – Stability studies using FDP myoglobin

Incubation of FDP Myoglobin at pH 3

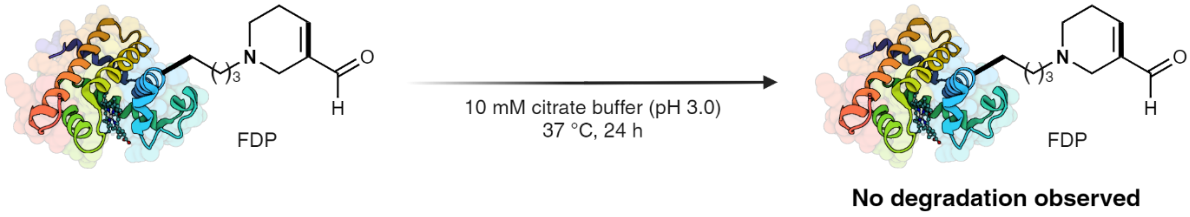

FDP-modified myoglobin (0.5 mg, 0.0983 mM), synthesized using the optimized conditions for homogeneous modification (8 equiv. acrolein), was dissolved in 300 µL of 10 mM citrate buffer (pH 3.0). The FDP myoglobin was heated at 37 °C for 24 hours, after which the protein was directly analyzed using LC-MS, which showed no degradation of the homogeneous FDP-modified product in the acidic conditions.

Incubation of FDP Myoglobin at pH 4

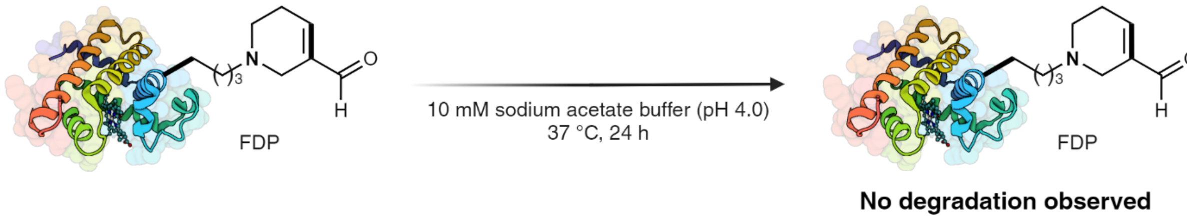

FDP-modified myoglobin (0.5 mg, 0.0983 mM), synthesized using the optimized conditions for homogeneous modification (8 equiv. acrolein), was dissolved in 300 µL of 10 mM sodium acetate buffer (pH 4.0). The FDP myoglobin was heated at 37 °C for 24 hours, after which the protein was directly analyzed using LC-MS, which showed no degradation of the homogeneous FDP-modified product in the acidic conditions.

Incubation of FDP Myoglobin at pH 10.8

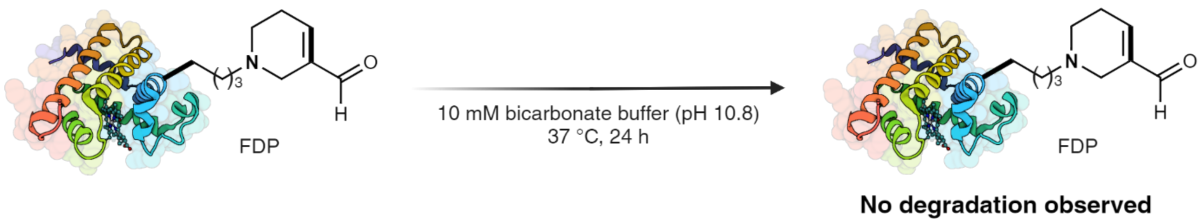

FDP-modified myoglobin (0.5 mg, 0.0983 mM), synthesized using the optimized conditions for homogeneous modification (8 equiv. acrolein), was dissolved in 300 µL of 10 mM bicarbonate buffer (pH 10.8). The FDP myoglobin was heated at 37 °C for 24 hours, after which the protein was directly analyzed using LC-MS, which showed no degradation of the homogeneous FDP-modified product in the basic conditions.

Fig. S18 – Conversion of FDP to MP on protein

Myoglobin: FDP to MP Conversion

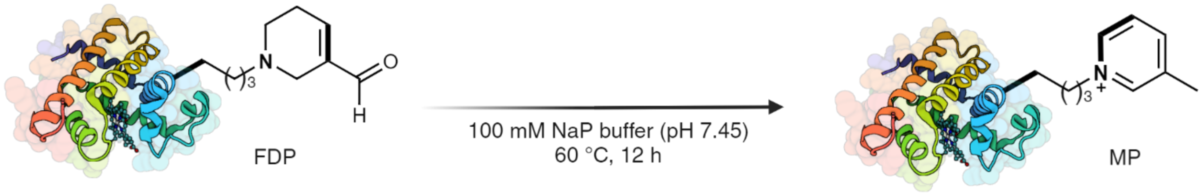

FDP-modified myoglobin (1 mg, 0.118 mM), synthesized using the optimized conditions for homogeneous modification (8 equiv. acrolein), was dissolved in 500 µL of 100 mM NaP buffer (pH 7.45). The reaction was stirred at 60 °C for 12 hours, after which the protein was directly analyzed using LC-MS. The conversion was found to be >95% with 37% 1 MP modification, 39% 2 MP modifications, and 24% 3 MP modifications.

Modification	Mass	Conversion
Unmodified	16951.7	N/A
1 MP	17026.6 (**+76**)	37%
2 MP	17102.3 (**+76**)	39%
3 MP	17178.1 (**+76**)	24%

Intact MS of Myoglobin Converted to MP

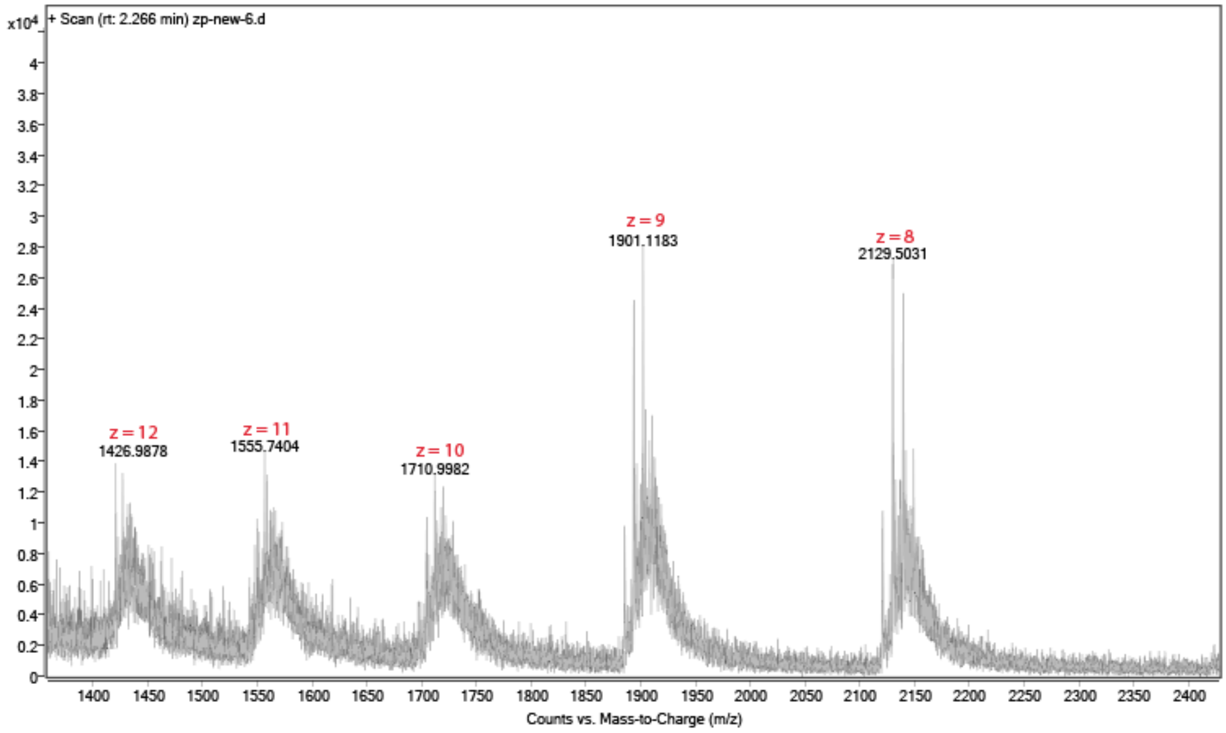

Deconvoluted MS of Myoglobin Converted to MP

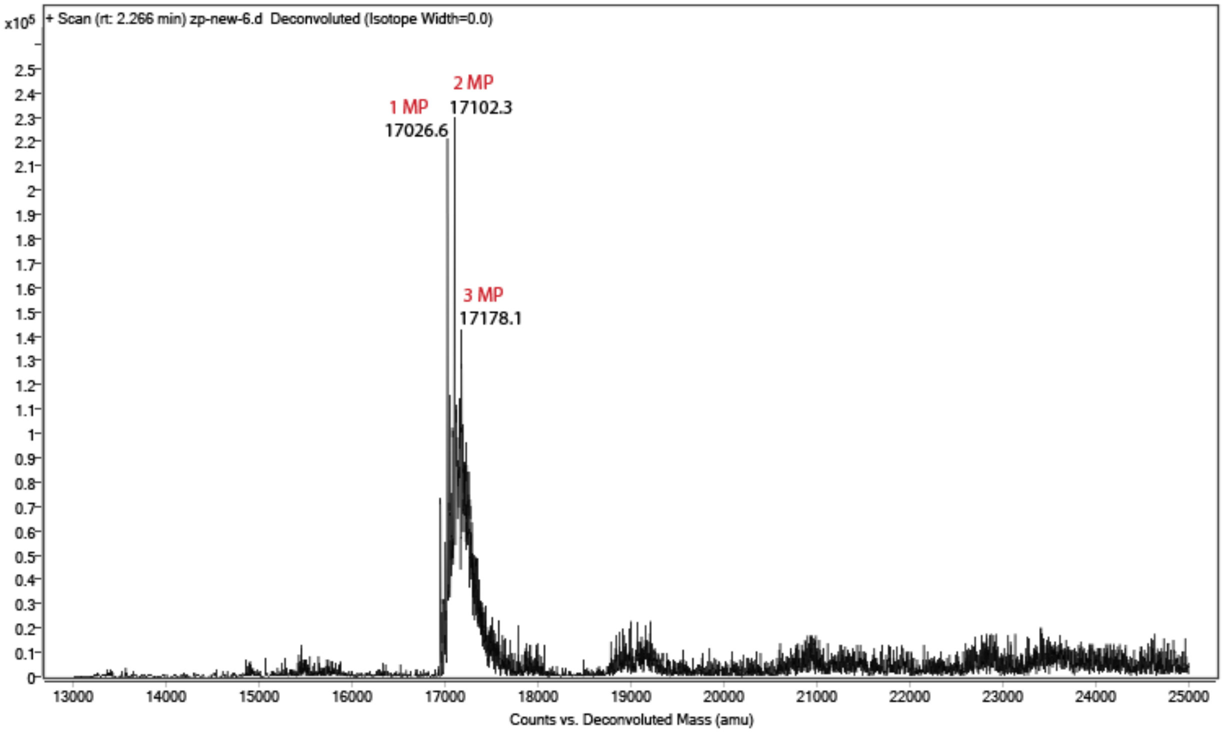

Ubiquitin: FDP to MP Conversion

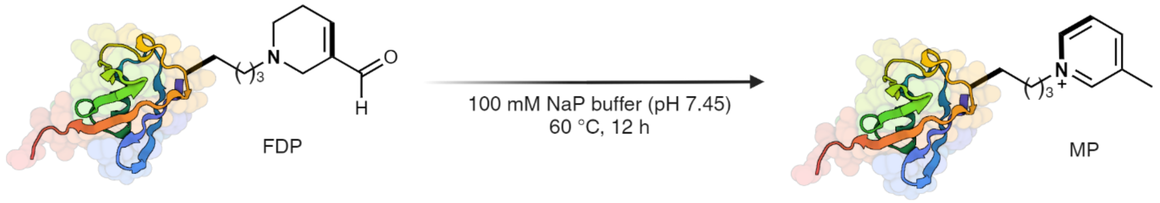

FDP-modified ubiquitin (1 mg, 0.234 mM), synthesized using the optimized conditions for homogeneous modification (8 equiv. acrolein), was dissolved in 500 μL of 100 mM NaP buffer (pH 7.45). The reaction was stirred at 60 °C for 12 hours, after which the protein was directly analyzed using LC-MS. The conversion was found to be >95% with 31% 1 MP modification, 50% 2 MP modifications, and 19% 3 MP modifications.

Modification	Mass	Conversion
Unmodified	8565.2	N/A
1 MP	8640.9 (+**76**)	31%
2 MP	8716.3 (+**76**)	50%
3 MP	8791.8 (+**76**)	19%

Intact MS of Ubiquitin Converted to MP

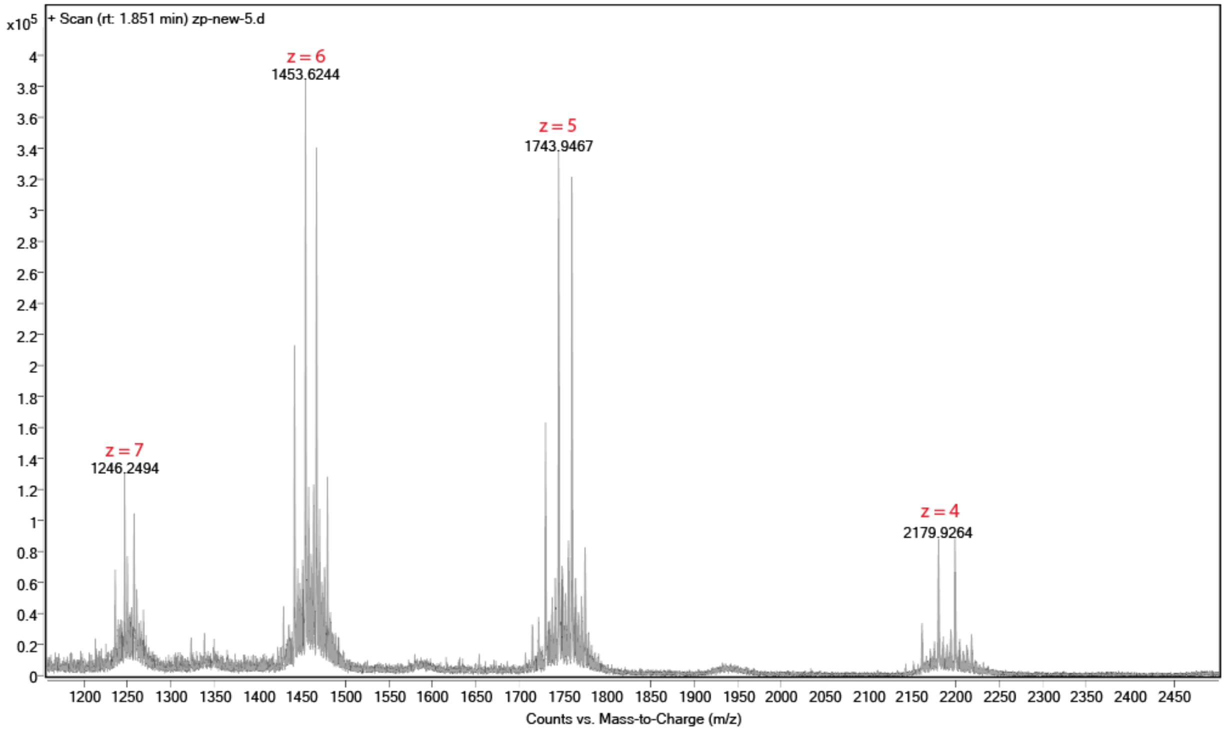

Deconvoluted MS of Ubiquitin Converted to MP

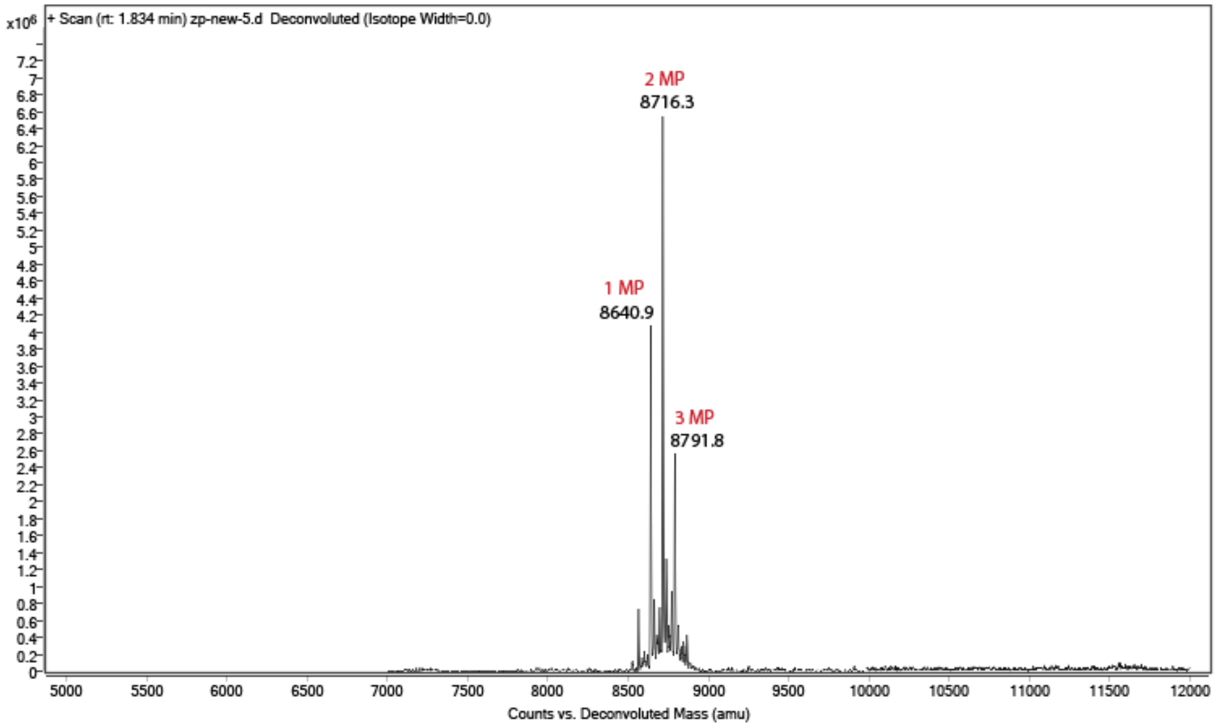

Aprotinin: FDP to MP Conversion

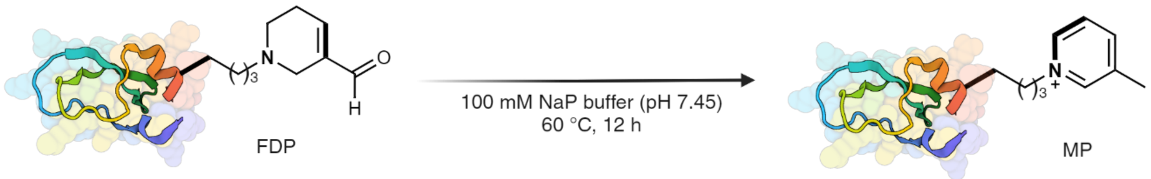

FDP-modified aprotinin (1 mg, 0.307 mM), synthesized using the optimized conditions for homogeneous modification (8 equiv. acrolein), was dissolved in 500 µL of 100 mM NaP buffer (pH 7.45). The reaction was stirred at 60 °C for 12 hours, after which the protein was directly analyzed using LC-MS. The conversion was found to be >95% with 57% 1 MP modification, and 43% 2 MP modifications.

Modification	Mass	Conversion
Unmodified	6511.1	N/A
1 MP	6587.4 (**+76**)	57%
2 MP	6663.0 (**+76**)	43%

Intact MS of Aprotinin Converted to MP

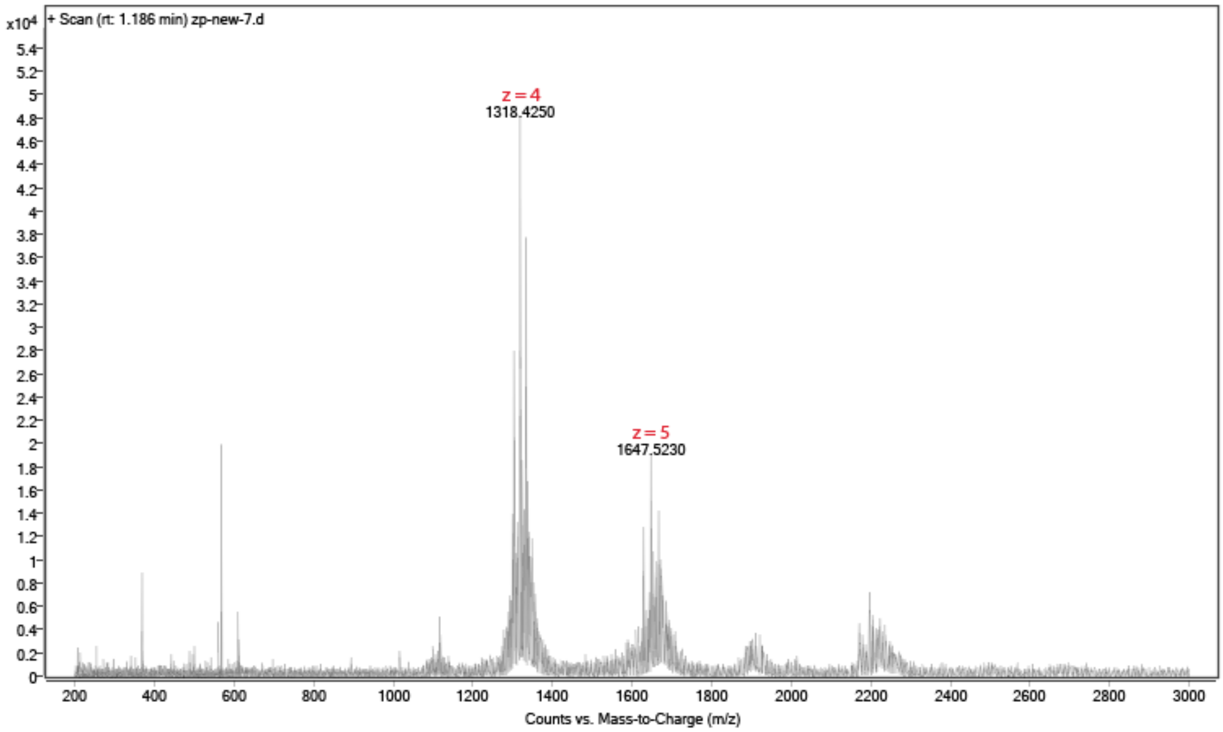

Deconvoluted MS of Aprotinin Converted to MP

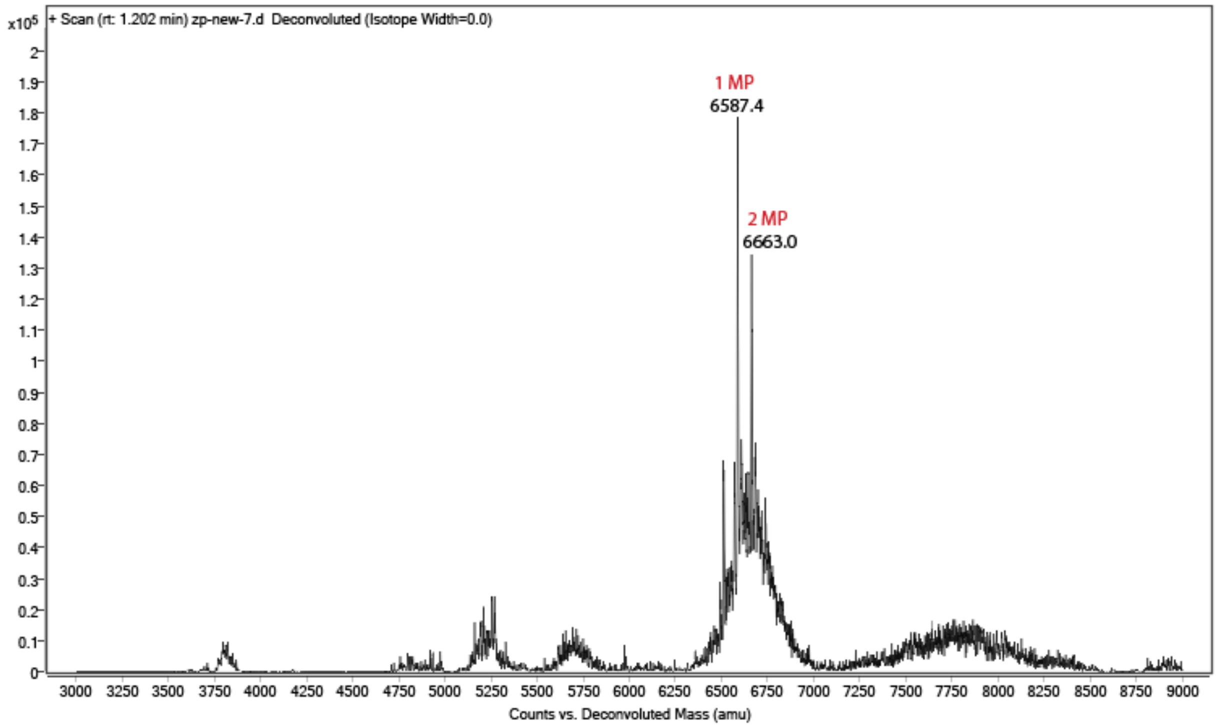

β-lactoglobulin: FDP to MP Conversion

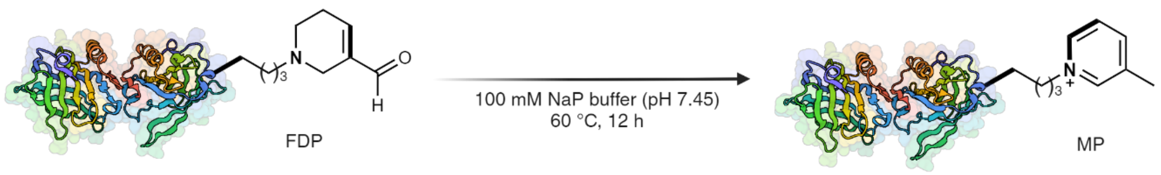

FDP-modified β-lactoglobulin (1 mg, 0.109 mM), synthesized using the optimized conditions for homogeneous modification (8 equiv. acrolein), was dissolved in 500 µL of 100 mM NaP buffer (pH 7.45). The reaction was stirred at 60 °C for 12 hours, after which the protein was directly analyzed using LC-MS. The conversion was found to be >95% with full conversion to a homogeneous product with 1 MP modification.

Modification	Mass	Conversion
Unmodified	18363.5	N/A
1 MP	18438.0 (+**76**)	>95%

Intact MS of β-lactoglobulin Converted to MP

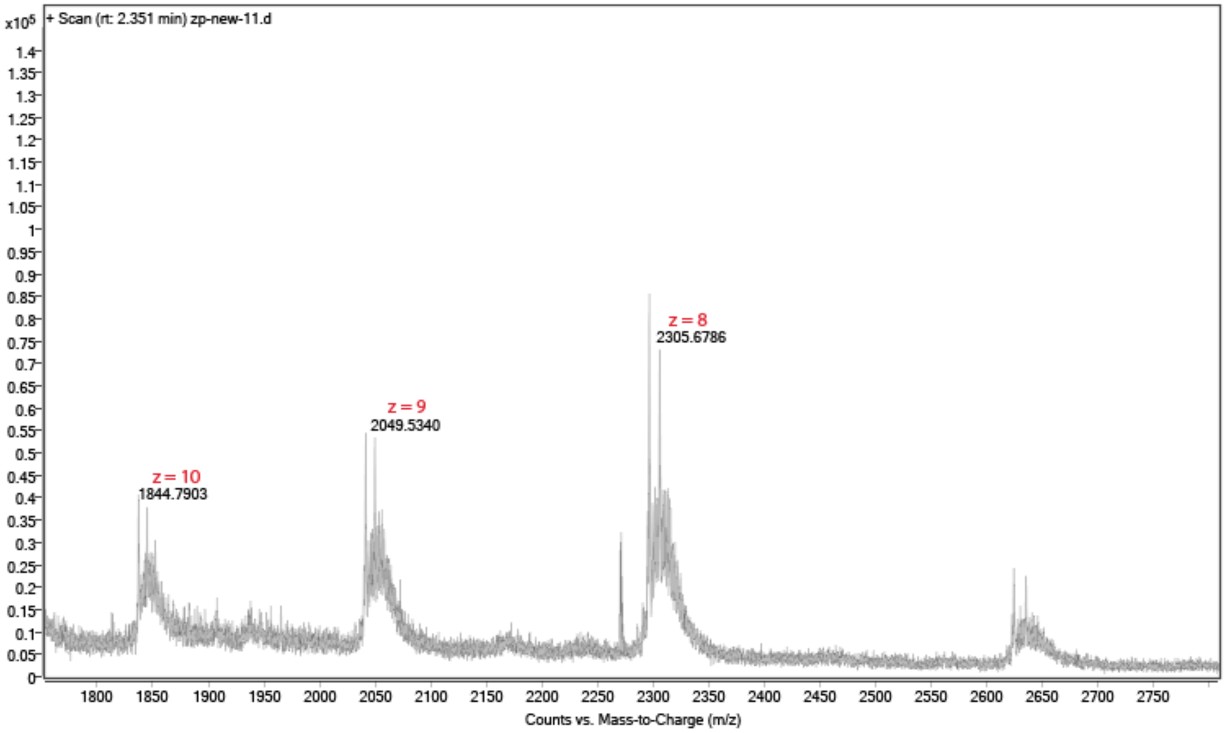

Deconvoluted MS of β-lactoglobulin Converted to MP

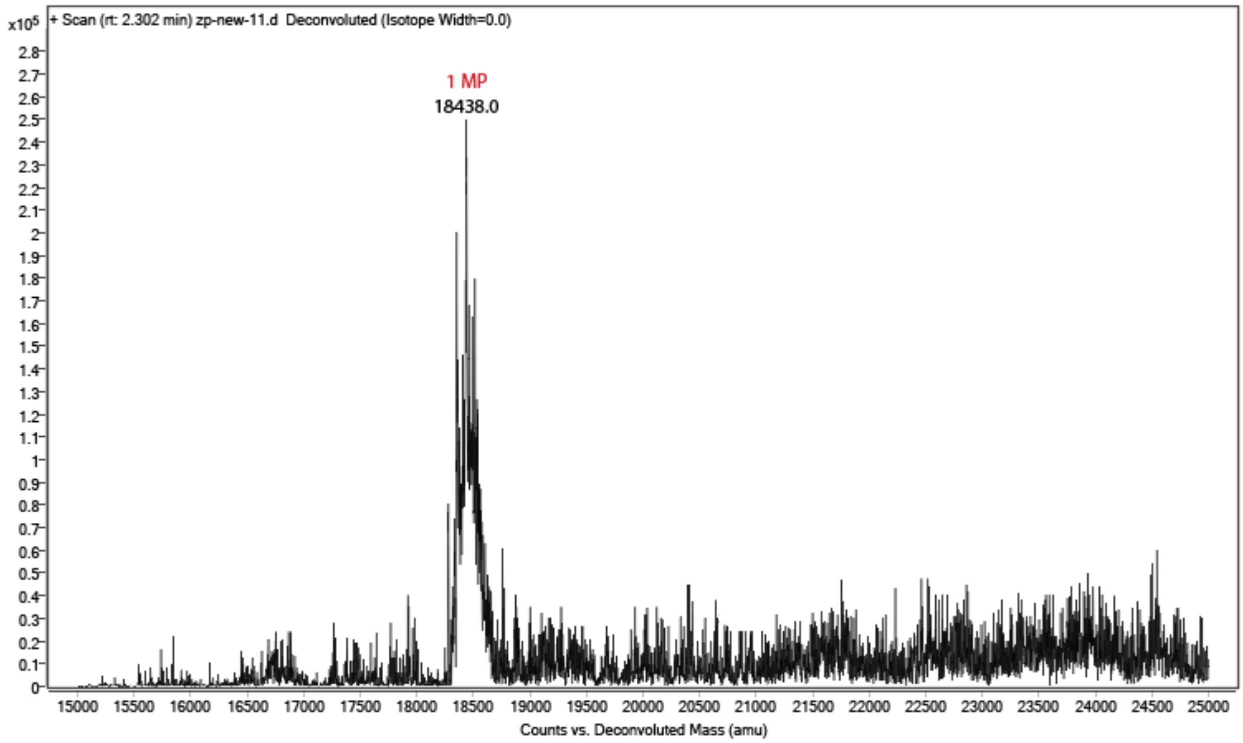

Fig. S19 – Bioactivity assay of FDP and MP modified myoglobin in oxidation of o-phenylenediamine with hydrogen peroxide.

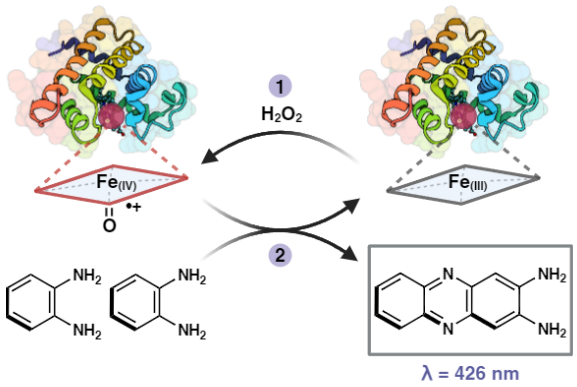

Enzymatic assay of myoglobin activity before and after labeling with acrolein to generate FDP and MP was evaluated by oxidation of *o*-phenylenediamine with hydrogen peroxide[3]. Oxidation of *o*-phenylenediamine to 2,3-diaminophenazine was monitored at 426 nm using Agilent Cary UV-Vis

Compact. Citric acid-Na_2HPO_4 buffer was prepared by mixing 0.1 M citric acid and 0.2 M Na2HPO4. 1 mg of FDP labeled myoglobin obtained by addition of 8 equiv. of acrolein was dissolved in 1 mL of citric acid- Na_2HPO_4 buffer separately in cuvette. To the reaction mixture in cuvette, 20 µL of 0.1 M *o*-phenylenediamine and 2 µL of 1 M hydrogen peroxide were added to the reaction mixture. The reaction was fully mixed by gentle pipetting up and down in the cuvette. The cuvettes were placed in Agilent Cary UV-Vis Compact and absorbance was measured at 426 nm every 5 min for a period of 1 h. To monitor the activity of MP labeled myoglobin, 1 mg of MP modified myoglobin was analyzed using the same procedure described above for FDP-myoglobin. To make a blank, 20 µL of 0.1 M *o*-phenylenediamine and 2 µL of 1 M hydrogen peroxide were added to 1 mL of citric acid- Na_2HPO_4 buffer and absorbance was measured at 426 nm every 5 min for a period of 1 h. This experiment was performed twice with (n=2).

Unmodified Myoglobin (2 replicates):

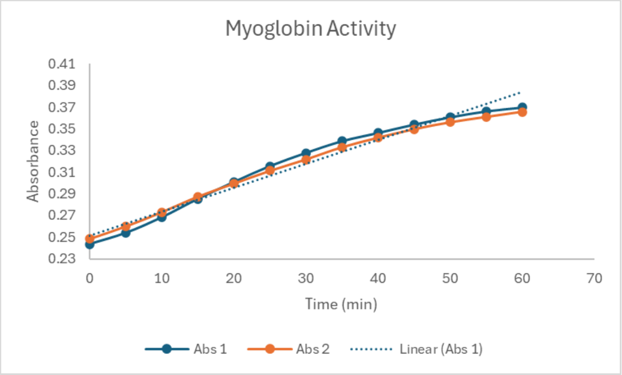

FDP Myoglobin (2 replicates):

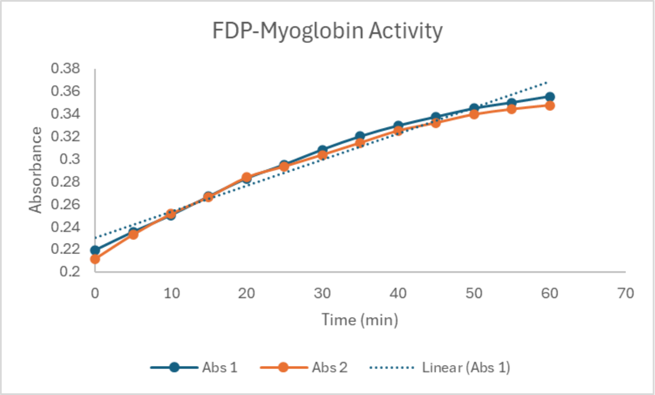

MP Myoglobin (2 replicates):

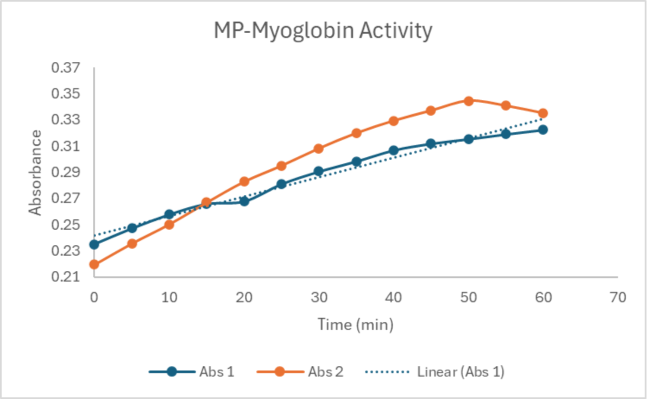

Summary of average activity for myoglobin, FDP-Myoglobin, and MP-Myoglobin.

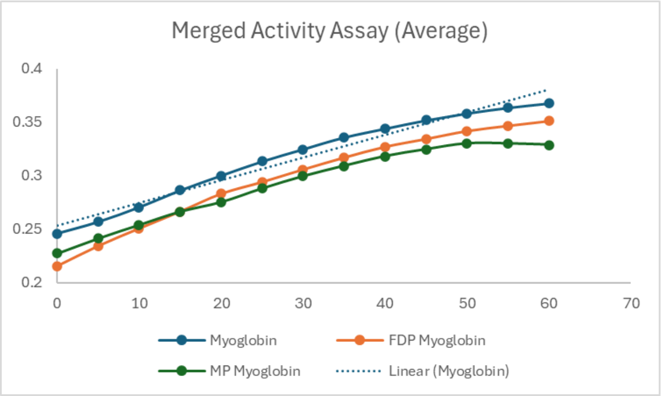

Fig. S20. Derivatization of FDP-aprotinin with aminoxy affinity handles.

Aminoxy-alkyne functionalization of aprotinin:

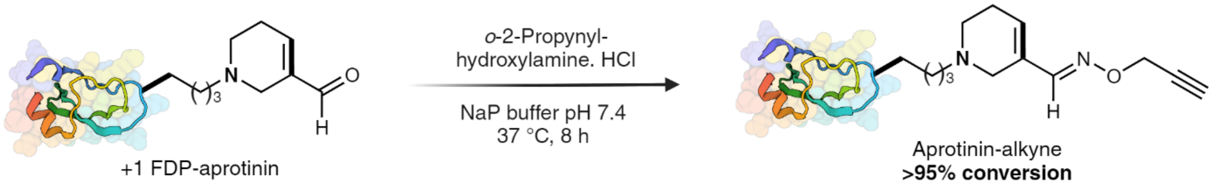

Single-FDP modified aprotinin (2 mg, 0.614 mM) was dissolved in 480 µL of 100 mM NaP buffer (pH 7.45), and *o*-2-Propynylhydroxylamine hydrochloride (0.164 mg, 5.84 mM) was added to the mixture. The reaction was stirred at 37 °C for 8 hours, after which the crude reaction mixture was

passed through Amicon Ultra 3 kDa spin-concentrator and washed with H_2O (5 x 500 μL) to remove the small molecule impurities. The labeled protein was redissolved in 0.1% formic acid in H_2O and analyzed using LC-MS. The conversion was found to be >95% to alkyne labeled aprotinin.

Deconvoluted MS of Single-FDP aprotinin labeled with alkyne handle

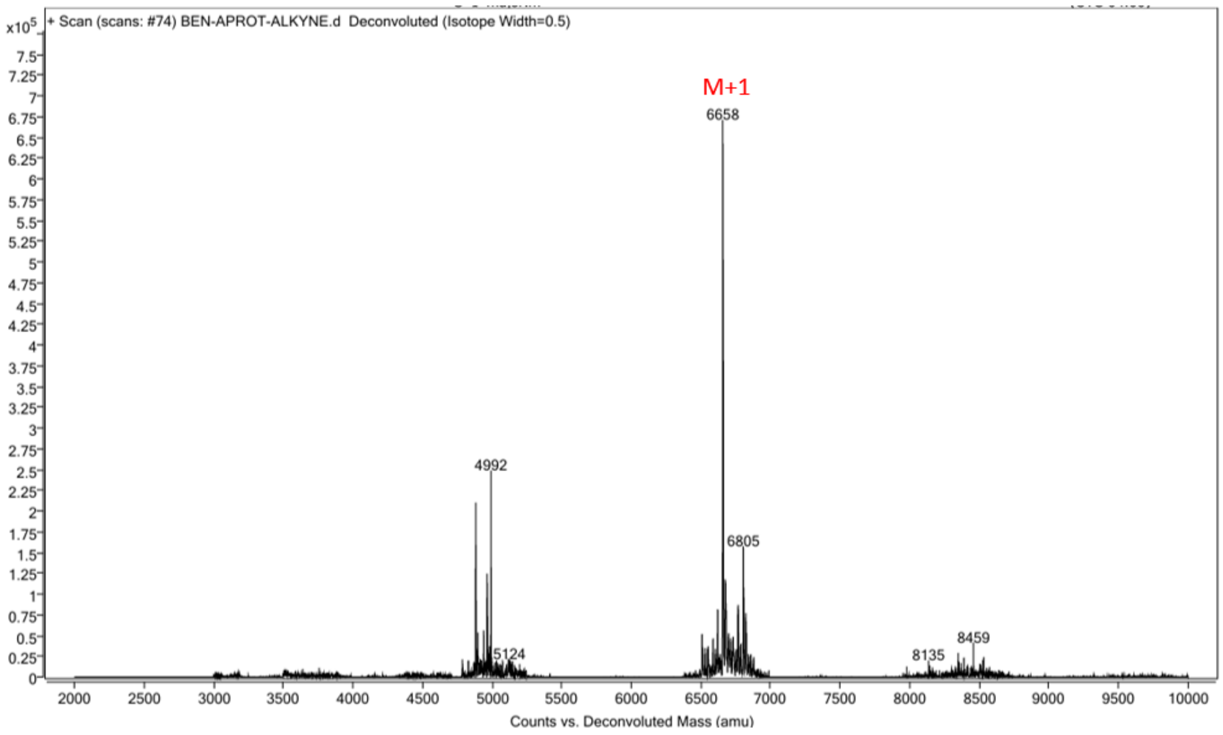

Aminoxy-biotin functionalization of aprotinin:

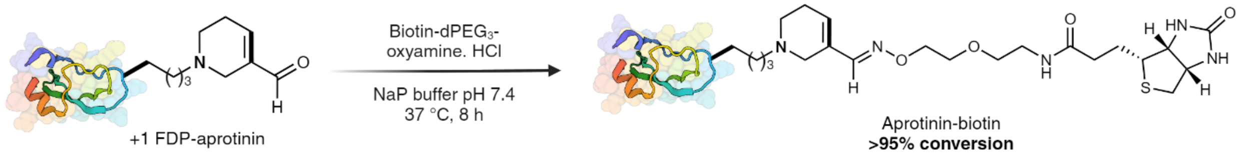

Single-FDP modified aprotinin (2 mg, 0.614 mM) was dissolved in 480 μL of 100 mM NaP buffer (pH 7.45), and biotin-dPEG$_3$-oxyamine hydrochloride (1.42 mg, 6.28 mM) was added to the mixture. The reaction was stirred at 37 °C for 8 hours, after which the crude reaction mixture was passed through Amicon Ultra 3 kDa spin-concentrator and washed with H_2O (5 x 500 μL) to remove the small molecule impurities. The labeled protein was redissolved in 0.1% formic acid in H_2O and analyzed using LC-MS. The conversion was found to be >95% to alkyne labeled aprotinin.

Deconvoluted MS of Single-FDP aprotinin labeled with biotin handle

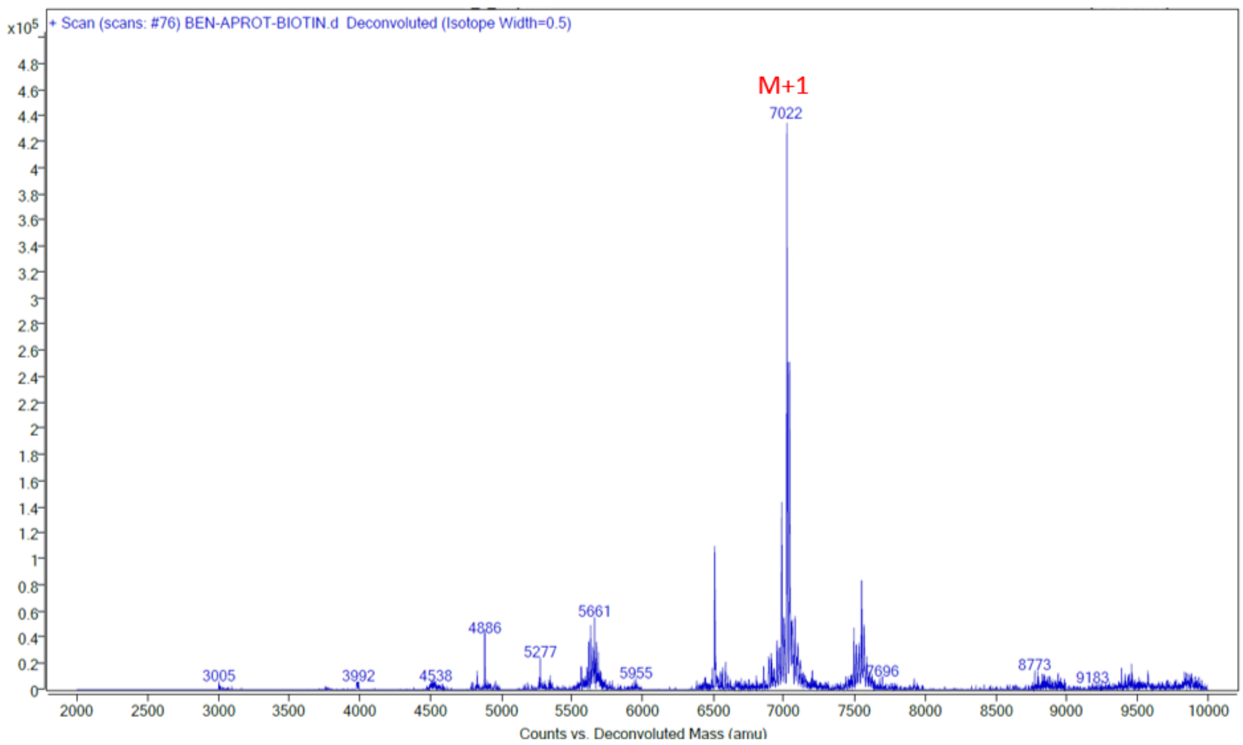

Fig. S21. Fluorophore labeling of proteins.

Thiol-FITC Probe Synthesis:

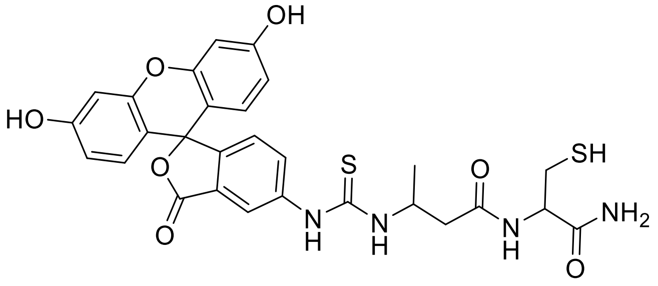

The **Thiol FITC Probe** was synthesized using solid-phase peptide synthesis techniques. 250 mg of Rink Amide Resin (0.15 mmol) was added to the solid-phase peptide synthesis tube. Fmoc-Cys(trt)-OH was coupled to the resin first, followed by Fmoc-β-ala. To couple the fluorophore, fluorescein 5-isothiocyanate (88 mg, 1.5 equiv., 0.23 mmol) was dissolved in 1 mL of DMSO, to which 9 mL of DCM was added. This FITC coupling solution was added to the solid phase synthesis tube and left for 1 hour. After coupling FITC, the probe was cleaved from resin and purified via preparatory HPLC to obtain the product as a bright orange powder (14 mg yield).

Thiol FITC Probe. LCMS, m/z 595.1317 (calcd. [M+H$^+$] = 595.1316), Purity: >99% (HPLC analysis at 220 nm). Retention time in HPLC: 14.9 min.

HPLC Trace of Thiol FITC Probe

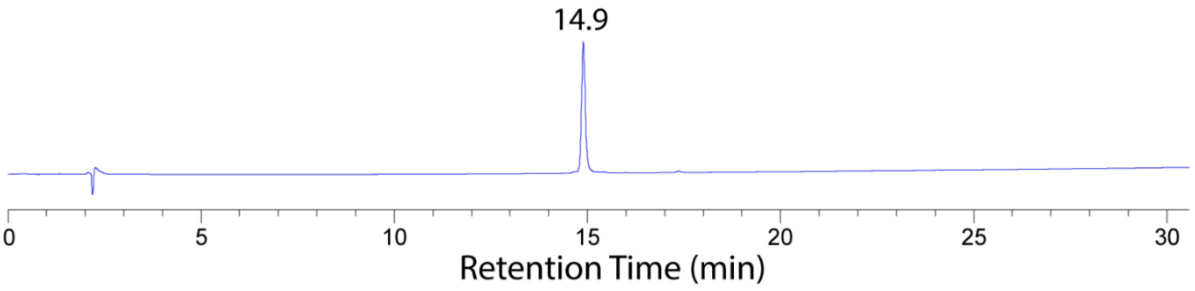

HRMS of Thiol FITC Probe

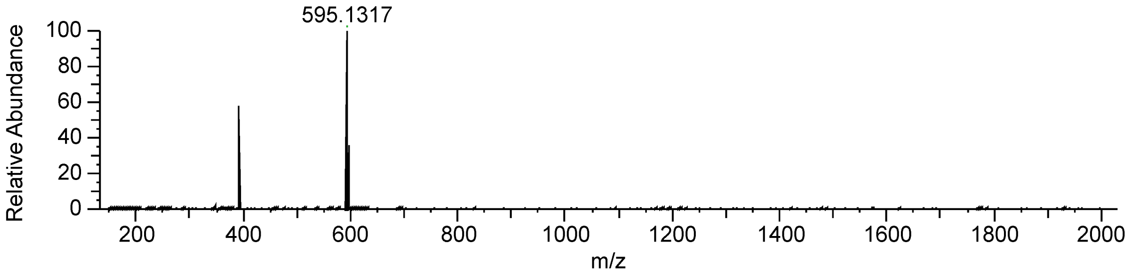

Fluorophore labeling of proteins:

To 120 µM solutions of proteins (Apo-transferrin, Bovine serum albumin, Myoglobin) in NaP buffer (pH 7.45) was added 3 mM of acrolein. The reaction was stirred at room temperature for 12 h. Samples were filtered using a 3 kDa molecular weight cut-off filter to obtain pure proteins. FDP labelled proteins were dissolved in 300 µL of NaP buffer (pH 7.45), followed by the addition of 5 µL of 16 mM Thiol-FITC dye in DMSO and 50 µL of 100 mM TCEP solution in water. The reaction was stirred for 5 h and filtered with a 3kDa filter, followed by analysis of proteins through in gel fluorescence imaging and coomassie blue staining. Samples were loaded on a Novex WedgeWell 4-20% Tris-Glycine gel. Gel was run in Tris-glycine running buffer at 180V. The gel was then stained with coomassie brilliant blue for 1 h and destained overnight.

Uncropped gel data for apo-transferrin:

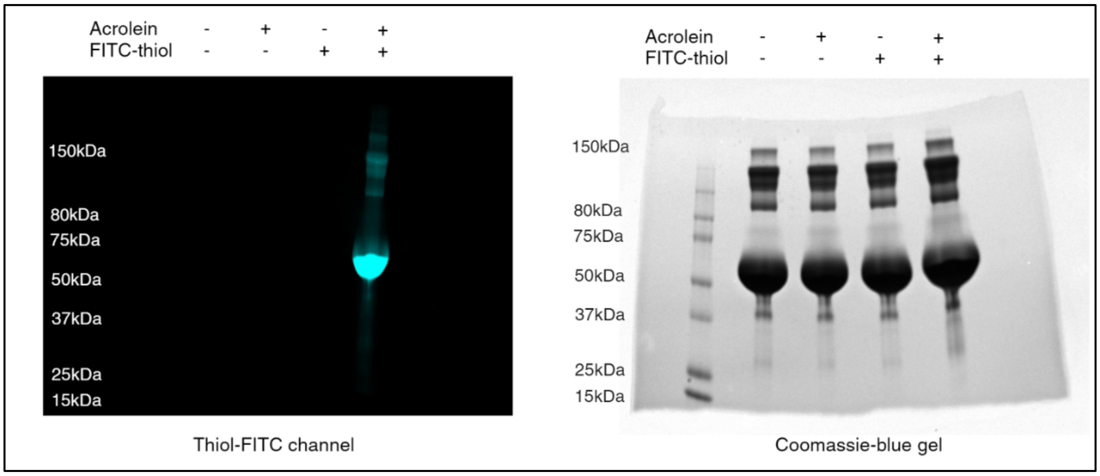

Uncropped gel data for bovine serum albumin:

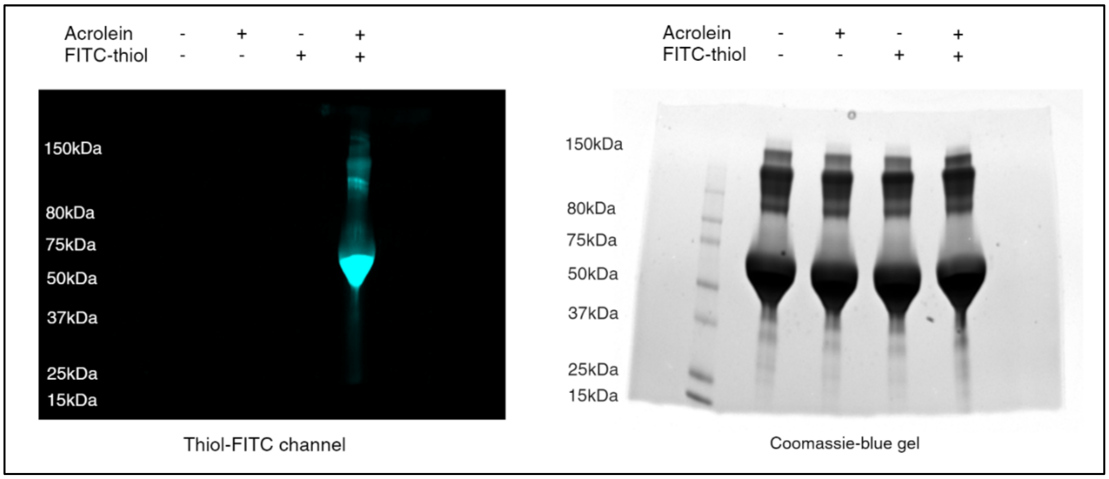

Uncropped gel data for myoglobin:

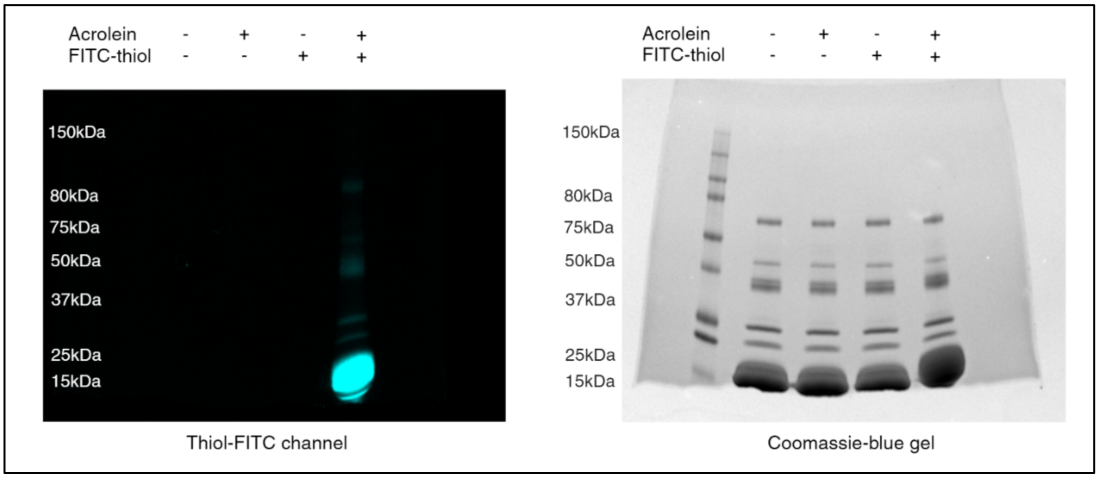

Fig. S22. Dual labeling of acrolein treated T47D cell lysate

Cell Culture and Drugs. Cells were maintained at 37 °C and 5% CO_2. T47D cells were cultured in RPMI supplemented with 10% (V/V) fetal bovine serum (FBS) and 1% (V/V) penicillin/streptomycin (100 µg/mL).

Cell Lysis. Whole cell lysate was generated by lysing cells on ice in RIPA buffer (50 mM TrisHCl [pH 8], 150 mM NaCl, 1% NP-40, 0.5% sodium deoxycholate, 0.1% SDS) supplemented with protease and phosphatase inhibitors. Lysates were centrifuged 6,500 x g, 10 m at 4°C, and soluble lysate was collected. Whole cell lysate proteins were separated using 16% SDS-PAGE. SDS-PAGE gels were stained with Coomassie brilliant blue dye.

Dose-dependent acrolein modification of lysates and dual labeling with aminoxy and thiol fluorophore. To 5 tubes (individual reactions) of 100 µg of lysate in NaP buffer pH 7.45 were treated with freshly prepared 10 µM, 50 µM, 100 µM, 500 µM, and 1000 µM of acrolein. The reaction was stirred at room temperature for 12 h. Upon completion of reaction, samples were acetone precipitated, followed by treatment with 2 µL of 10 mM hydroxylamine-647 (HA-647) fluorophore in DMSO. The reaction was stirred for 3 h and acetone precipitated, followed by analysis of proteins through in gel fluorescence imaging and coomassie blue staining. For dual labeling of lysate proteins, samples were first modified with acrolein and labeled with hydroxylamine-647 dye, followed by treatment with thiol-FITC fluorophore. To achieve this, acetone precipitated HA-647 labeled proteins were resuspended in NaP buffer (pH 7.45) and treated with 5 µL of 16 mM Thiol-FITC dye in DMSO and 50 µL of 100 mM TCEP solution in water. The reaction was stirred for 5 h and acetone precipitated. Samples were loaded on a Novex WedgeWell 4-20% Tris-Glycine gel. Gel was run in Tris-glycine running buffer at 180V. The gel was then stained with coomassie brilliant blue for 1 h and destained overnight.

Uncropped gel data for dual labeling of T47D lysate:

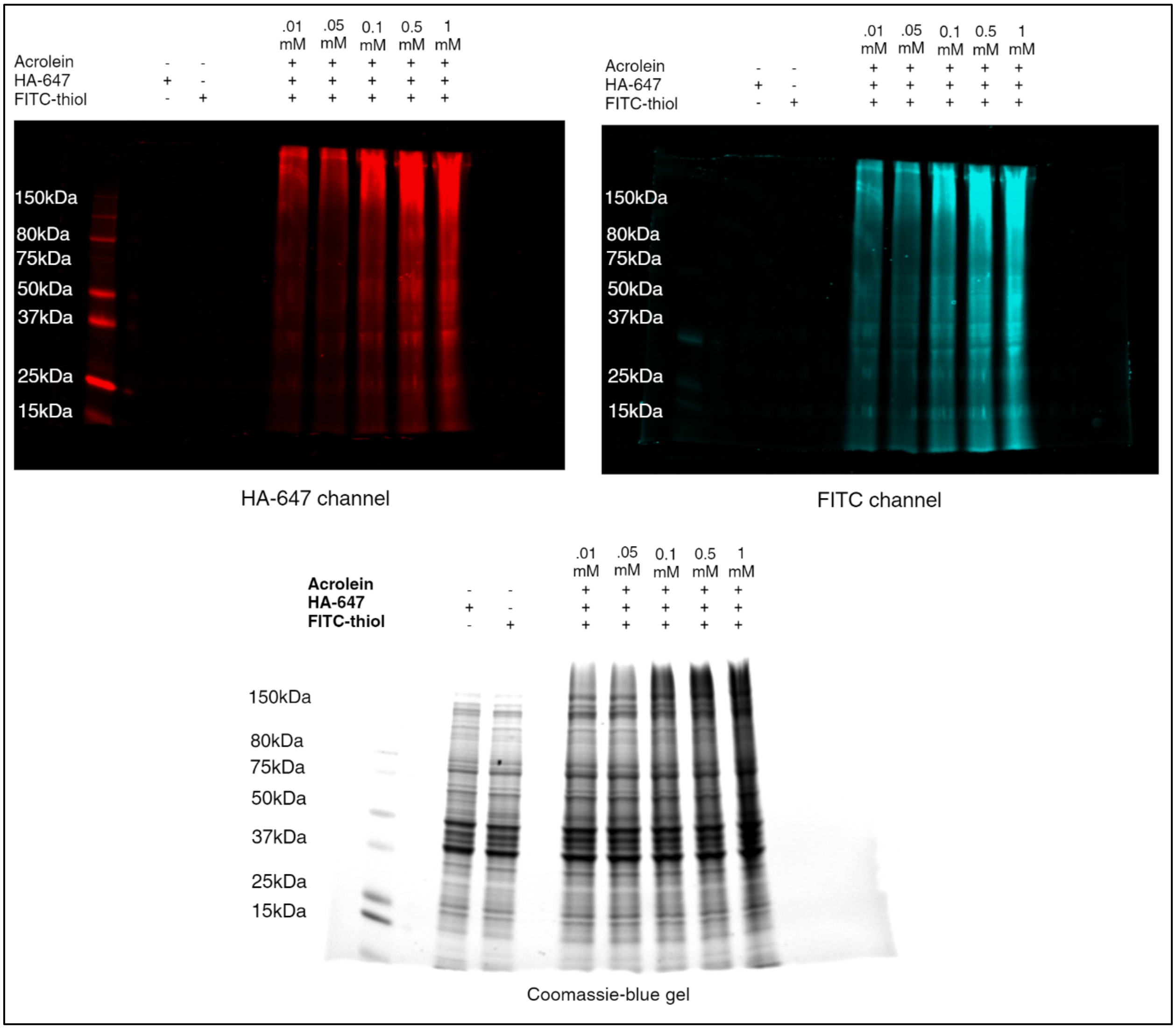

Fig. S23. Acrolein-mediated activity-based protein profiling.

Dose-dependent acrolein modification of lysates. To 5 tubes (individual reactions) of 100 µg of lysate in NaP buffer pH 7.45 were treated with freshly prepared 10 µM, 50 µM, 250 µM, 500 µM, and 1000 µM of acrolein. The reaction was stirred at room temperature for 12 h. Upon completion of reaction, modified lysate samples were acetone precipitated and digested using Glu C cleavage Kit by Promega. This experiment was performed with (n= 2 biological samples).

LC-MS/MS. Digested samples were resuspended in Buffer A (0.1% FA in water) and the peptide amount was determined by Pierce™ Quantitative Peptide Assays & Standards (Thermo Fisher Scientific) according to manufactures instructions. Samples were injected into a nanoElute UPLC

autosampler (Bruker Daltonics) coupled to a timsTOF Pro2 massspectrometer (Bruker Daltonics). The peptides were loaded on a 25 cm Aurora ultimate CSI C18 column (IonOpticks) and chromatographic separation was achieved using a linear gradient starting with a flow rate of 250 nl/min from 2% Buffer B (0.1% FA in MeCN) and increasing to 13% in 42 min, followed by an increase to 23% B in 65 min, 30% B in 70 min, then the flow rate was increased to 300 nl/min and 80% B in 85 min, this was kept for 5 min. The mass-spectrometer operated in positive polarity for data collection using a data-dependent acquisition (ddaPASEF) mode. The cycle time was 1.17 s and consisted of one full scan followed by 10 PASEF/MSMS scans. Precursors with intensity of over 2500 (arbitrary units) were picked for fragmentation and precursors over the target value of 20,000 were dynamically excluded for 1 min. Precursors below 700 Da were isolated with a 2 Th window and ones above with 3 Th. All spectra were acquired within an m/z range of 100 to 1700 and fragmentation energy was set to 20 eV at 0.6 1/K0 and 59 eV at 1.60 1/K0.

Database search (MSFragger). MS raw files were searched FragPipe GUI version 20 with MSFragger (version 3.8) as the search algorithm. Protein identification was performed with the human Swisspot database (20′456 entries) with acetylation (N-terminus), and oxidation on methionine was set variable modification. To account for the mass shift introduced by the different chemical handles a variable mass shift of 94.1130 Da, 76.0326 Da, and 112.128 Da on Lysine, 56.0263 Da on cysteine and 56.0262 Da on histidine with a maximal occurrence of 2 for each modification. Carbamidomethylation of cysteine residues was considered a fixed modification. Glu C was set as the enzyme with up to two missed cleavages. The peptide length was set to 7–50, and the peptide mass range of 500–5000 Da. For MS2-based experiments, the precursor tolerance was set to 20 ppm and fragment tolerance to 20 ppm. Peptide spectrum matches (PSMs) were adjusted to a 1% false discovery rate using Percolator as part of the Philosopher toolkit (v5). For label-free quantification, match-between-runs were enabled. All downstream analysis was performed in R (version 2023.03.0). Individual samples were normalized to the mean of all quantified peptides.

Excel sheet of analysis is attached Supplementary Data 1. The mass spectrometry proteomics data (Data 1) generated in this study have been deposited to the ProteomeXchange Consortium via the PRIDE partner repository with the dataset identifier PXD054410.

Heatmap analysis of hyperreactive lysine sites

Heatmap analysis of the dose-dependent labelling of lysates with acrolein identified a dose-dependent labeling of lysine residues as intensity of modified peptides increased as concentration of acrolein increased.

Sequence specificity of acrolein on a proteome-wide scale

To assess the selectivity for lysine, we performed a database search of proteomics data with the FDP or MP on Lysine, Michael addition modification on Cysteine, and Michael addition modification on His. 835 modifications were observed on lysine, 7 modifications on cysteine and no modification on histidine.

Sequence Motif analysis

Sequence motif of modified lysine sites: To identify the sequence motif of modified lysine sites, Excel list containing the acrolein modified lysine sites were utilized. Sequences containing 4 residues from the left and 4 residues from the right of modified lysine sites were utilized, with lysine as the fixed positions. Motif analysis was done using a p-value <0.05. The sequence motif was generated using “probability logo generator for biological sequence motif “ plogo v1.2.0.[4]

Evaluation of E-D electrostatic interaction with K

To evaluate E-D electrostatic interaction with lysine, 100 randomized FDP and MP modified peptide sequences were analyzed. The PDB structures of proteins containing these sites were evaluated and E-D lysine interactions within (< 3.2 Å) considered as positive (presence of interaction), with greater distances considered as negative (no interaction). 77% of analyzed peptides led to a positive E-D lysine interaction.

Excel sheet of analysis is attached Supplementary Data 2.

Gene Ontology (GO) analysis

For Gene Ontology (GO) analysis, gene list of FDP and MP modified proteins were utilized as input in metascape[5] and gProfiler, with input and analysis species set to *H. sapiens*. Pathway and process enrichment analysis was carried out with the following ontology sources: KEGG Pathway, GO Biological Processes, Reactome Gene Sets, Canonical Pathways, CORUM, WikiPathways, and PANTHER Pathway. All genes in the human genome were used as the enrichment background. Terms with a p-value < 0.01, a minimum count of 3, and an enrichment factor > 1.5 were utilized. p-values were calculated based on the cumulative hypergeometric distribution, and q-values were calculated using the Benjamini-Hochberg procedure.

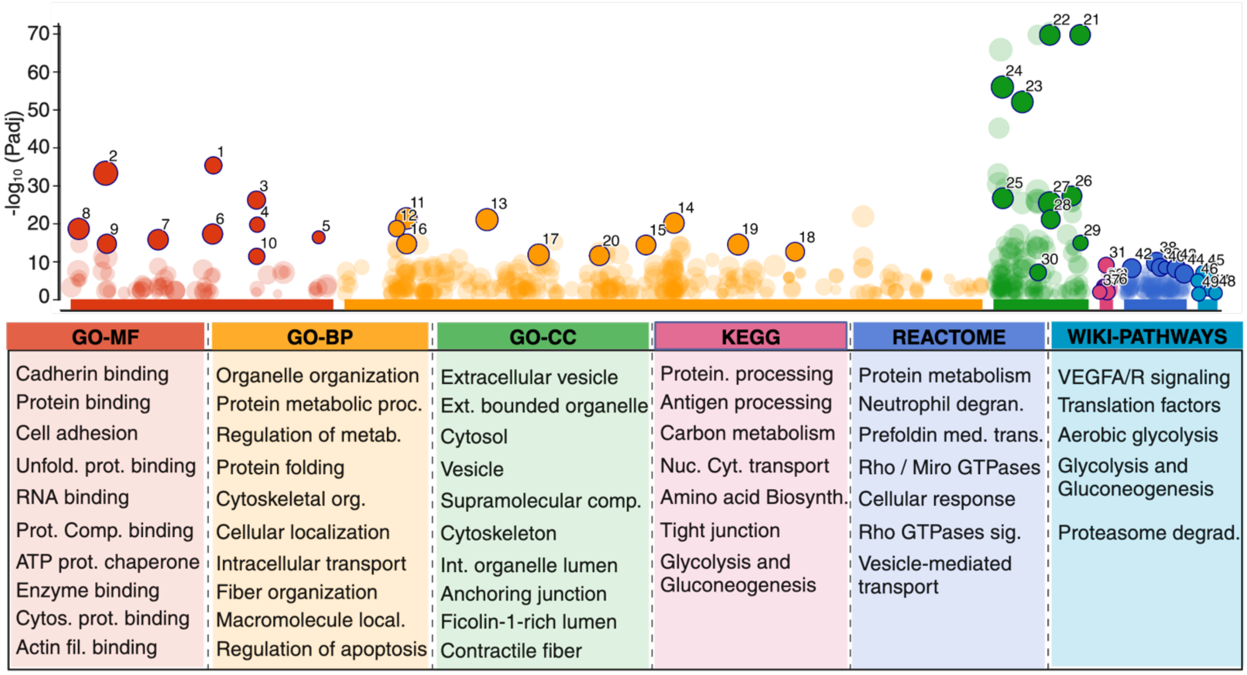

Fig. S24 – MP lysine mass booster data

Mass Booster Analysis Procedure:

Peptide 1a (0.98 mg, 2 mM) and MP analogue 3a (1.14 mg, 2 mM), purified via analytical HPLC, were dissolved in 1 mL of H_2O at equal concentration. 3 µL of this sample was injected into a Thermo scientific LTQ Orbitrap Velos mass spectrometer, and the maximum absolute ion counts for the masses corresponding to peptide **1a** and MP analogue **3a** were recorded. This sample was diluted serially to obtain the concentrations listed in the table below, and the 3 µL injection volume was kept constant. This serial dilution was repeated twice more using the initial 2 mM sample to obtain 3 replicates for each concentration; the compiled absolute ion count data can be found in the table below. MP analogue **3a** displayed significantly greater ionizability than peptide **1a**, remaining detectable (absolute ion count >1000) at a concentration of 500 fM. For comparison, peptide **1a** was no longer detectable (absolute ion count >1000) at a concentration less than 100 nM.

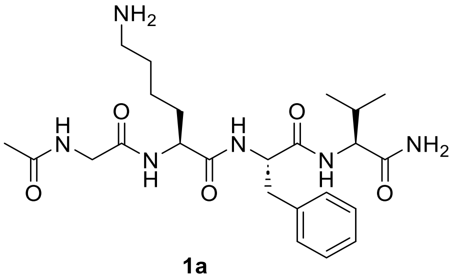

1a

vs.

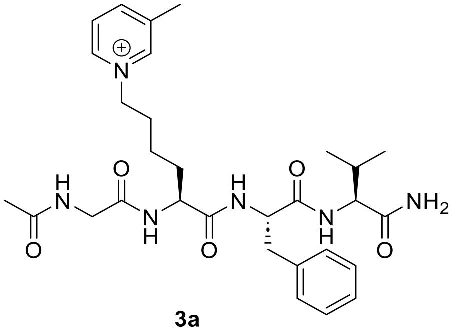

3a

Ion Count Data for Peptide 1a

	Absolute Ion Count			
Concentration	Trial 1	Trial 2	Trial 3	Average
2 mM	6404351.5	9973281	11294728	9224120
1 mM	7390636	9298345	6949882	7879621
500 µM	3289149.8	2896254	3106934	3097446
250 µM	3207345.8	3141130	2665730	3004735
100 µM	1866347.6	2343882	2329829	2180019
50 µM	1542149.3	1349665	996662	1296159
25 µM	909235	525987.5	775791.6	737004.7
10 µM	295571.28	489317.6	189719	324869.3
5 µM	153312.92	124239.7	133735.6	137096.1
2.5 µM	74618.06	66313.04	66496.9	69142.67
1 µM	49910.67	55052.08	40075.9	48346.22
500 nM	20609.97	15312.6	18999.6	18307.39
250 nM	14187.95	8323.7	9207.75	10573.13
100 nM	9597.02	4807.68	5196.68	6533.793

Ion Count Data for MP Lysine Peptide 3a

	Absolute Ion Count			
Concentration	Trial 1	Trial 2	Trial 3	Average
2 mM	25439812	34431440	42912012	34261088
1 mM	22152066	30805280	28257066	27071471
500 µM	11409084	15332078	15383908	14041690
250 µM	10081418	11071189	14188551	11780386
100 µM	8149616	7699284	4893051	6913984
50 µM	4484861.5	6193262	6228968	5635697
25 µM	4280016	4594638	3530854	4135169
10 µM	1931391.25	3491368	1943530	2455430
5 µM	895717.06	1152869	1187162	1078583
2.5 µM	746400.81	662229.7	675384.4	694671.6
1 µM	474989.12	477757.9	463097.4	471948.1
500 nM	157474.98	164131.1	202015.5	174540.5
250 nM	132246.72	74169.64	87415.6	97943.99
100 nM	83628.58	58056.49	50766.21	64150.43
1 nM	26636.39	21222.09	19438.48	22432.32
500 fM	2290.92	1319.35	2833.53	2147.933

Bar Chart of Compiled Average Ion Count Data for Mass Booster Assay

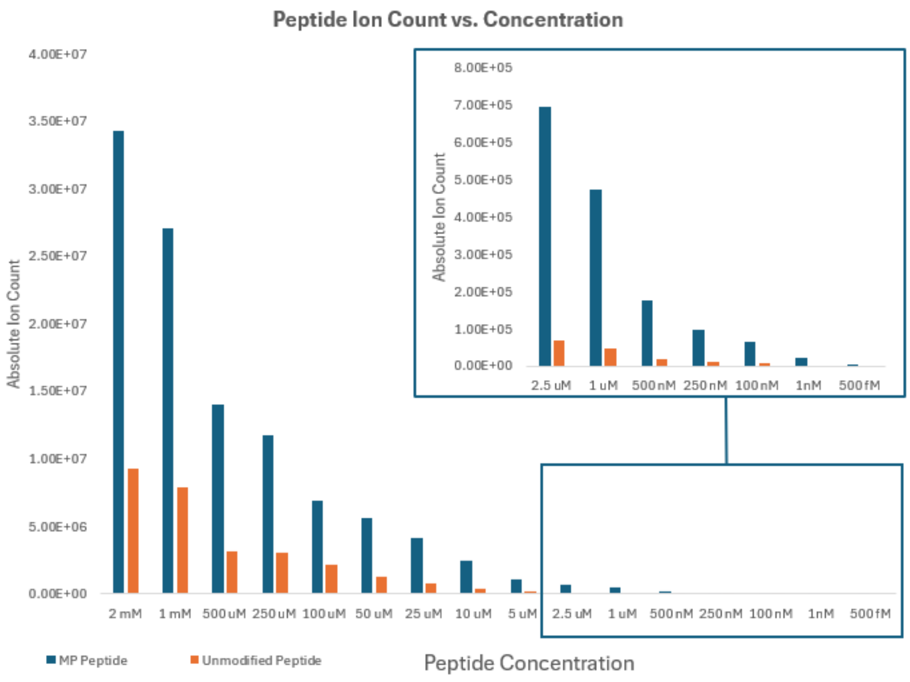

Evaluating Ability of Oxime-Tagged FDP to Convert to MP Upon Heating

To explore the potential to enrich FDP-labeled proteins using oxime chemistry and then heat the captured proteins to convert the oxime product to MP with mass boosting properties, FDP-modified aprotinin was tagged with alkyne hydroxylamine, and then the sample was heated. However, the oxime-tagged FDP is stable and does not aromatize under heating conditions.

Tagging FDP Aprotinin with Alkyne Hydroxylamine

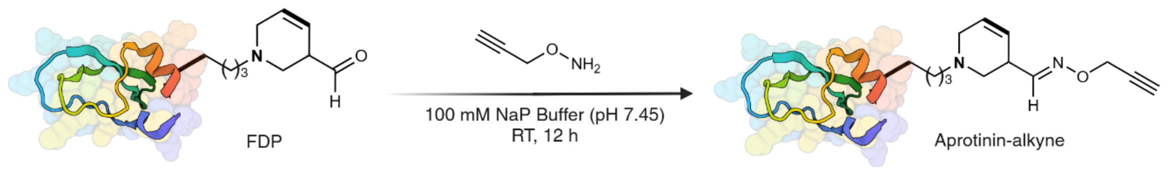

The FDP-modified aprotinin (1 mg, 0.307 mM) was dissolved in 480 µL of 100 mM NaP buffer (pH 7.45), and *o*-2-Propynylhydroxylamine hydrochloride (0.165 mg, 3.07 mM) was added to the mixture from a freshly prepared stock solution (20 µL). The reaction was stirred at RT for 12 hours, after which the crude reaction mixture was passed through Amicon Ultra 3 kDa spin-concentrator

and washed with H_2O (5 x 500 µL) to remove the small molecule impurities. The labeled protein was redissolved in 0.1% formic acid in H_2O and analyzed using LC-MS. The conversion was found to be >95% to alkyne labeled aprotinin (HA tag) with 52% 1 FDP with alkyne labeling and 48% 2 FDP with alkyne labeling.

Deconvoluted MS of FDP Aprotinin Tagged with Alkyne Hydroxylamine (HA tag)

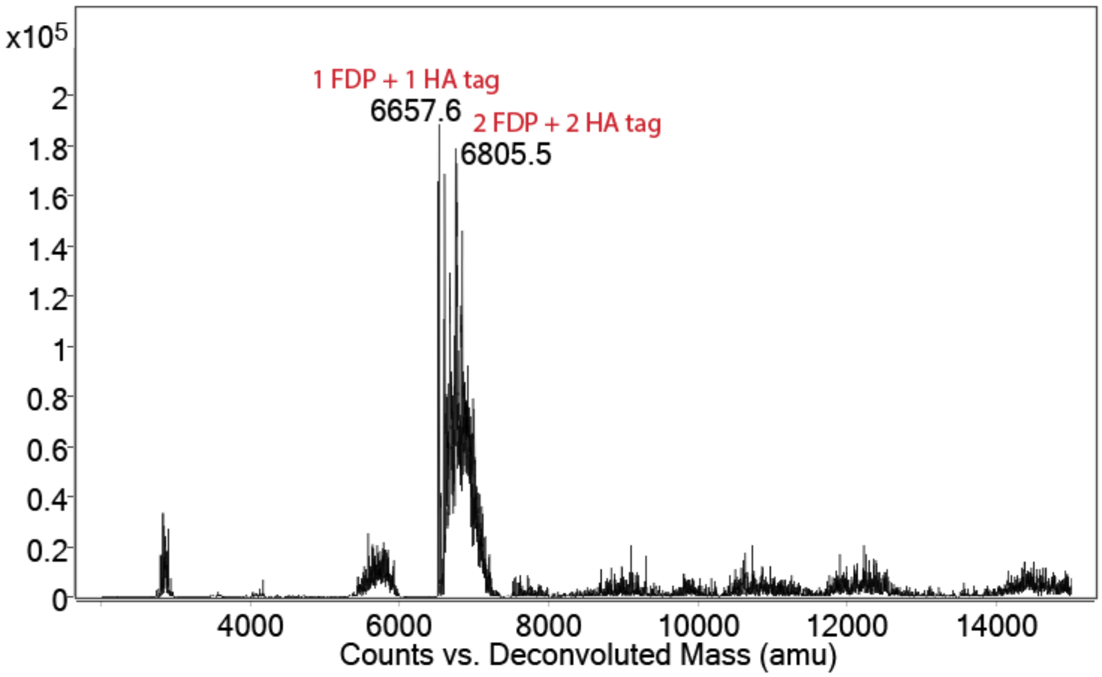

Heating Oxime-Tagged FDP to Evaluate Conversion to MP

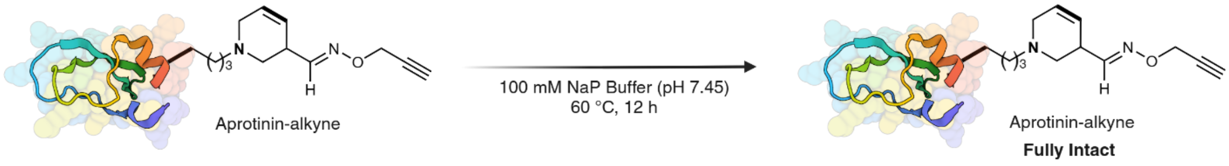

Alkyne-labeled aprotinin (1 mg, 0.300 mM) was dissolved in 500 µL of 100 mM NaP buffer (pH 7.45) and the reaction was stirred at 60 °C for 12 hours, after which the crude reaction mixture was passed through Amicon Ultra 3 kDa spin-concentrator and washed with H_2O (5 x 500 µL) to remove any impurities. The labeled protein was redissolved in 0.1% formic acid in H_2O and

analyzed using LC-MS. No aromatization/formation of the MP product was observed. The oxime product was stable under heating conditions.

Deconvoluted MS of Unmodified FDP Aprotinin Tagged with Hydroxylamine After Heating

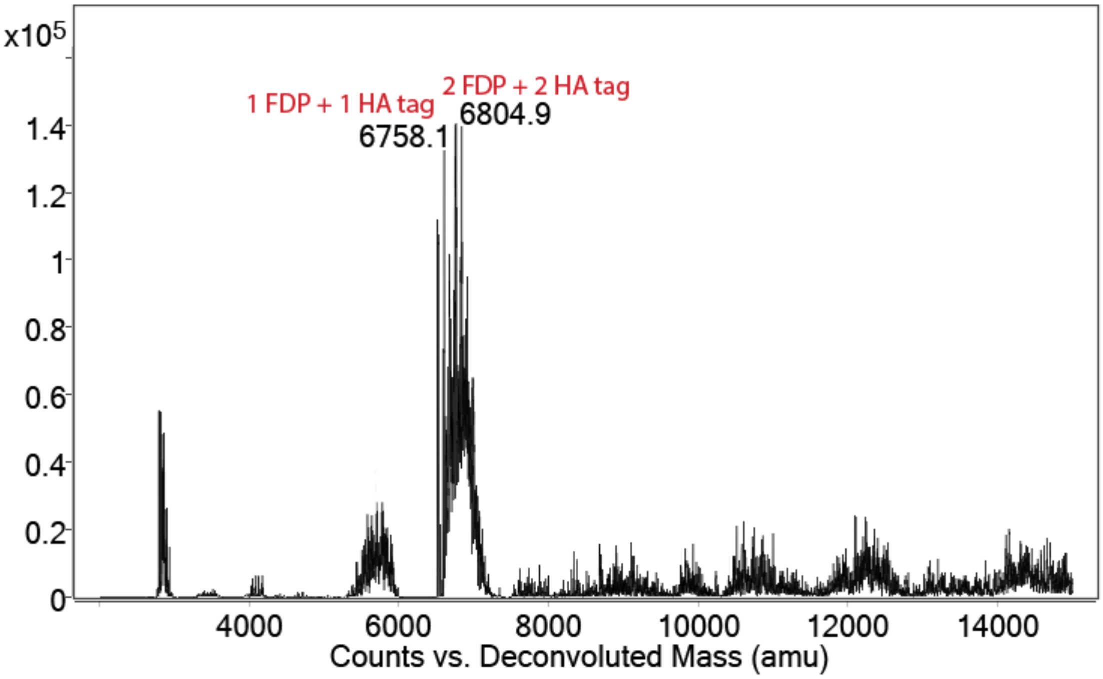

Reversible Enrichment of FDP Using Hydrazide: MS Proof at Single Protein Level

FDP-modified aprotinin was tagged with biotin hydrazide, and then hydrazone product was placed in an acidified and heated mixture to reverse the tagging and form MP lysine. The reversible nature of hydrazide chemistry offers the potential to enrich FDP-modified proteins, reform FDP upon acidification, and then heat the proteins to form MP, offering a potential proteomics application for the mass-boosting properties of MP lysine.

Tagging FDP Aprotinin with Biotin Hydrazide

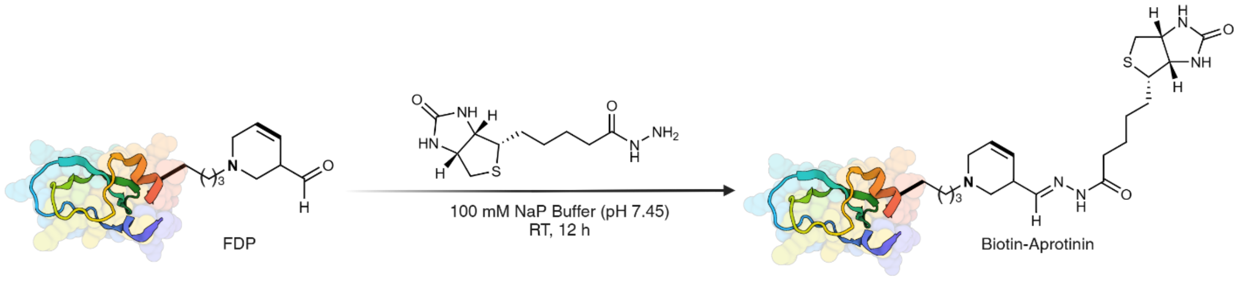

The FDP-modified aprotinin (1 mg, 0.307 mM) was dissolved in 480 µL of 100 mM NaP buffer (pH 7.45), and (+)-biotin hydrazide (0.40 mg, 3.07 mM) was added to the mixture from a freshly prepared stock solution (20 µL). The reaction was stirred at RT for 12 hours, after which the crude reaction mixture was passed through Amicon Ultra 3 kDa spin-concentrator and washed with H_2O (5 x 500 µL) to remove the small molecule impurities. The labeled protein was redissolved in 0.1% formic acid in H_2O and analyzed using LC-MS. The conversion from FDP-modified aprotinin was found to be 75% to the hydrazone product with 30% 1 FDP with 1 hydrazone tag and 45% 2 FDP with 1 hydrazone tag.

Deconvoluted MS of FDP Aprotinin Tagged with Hydrazide

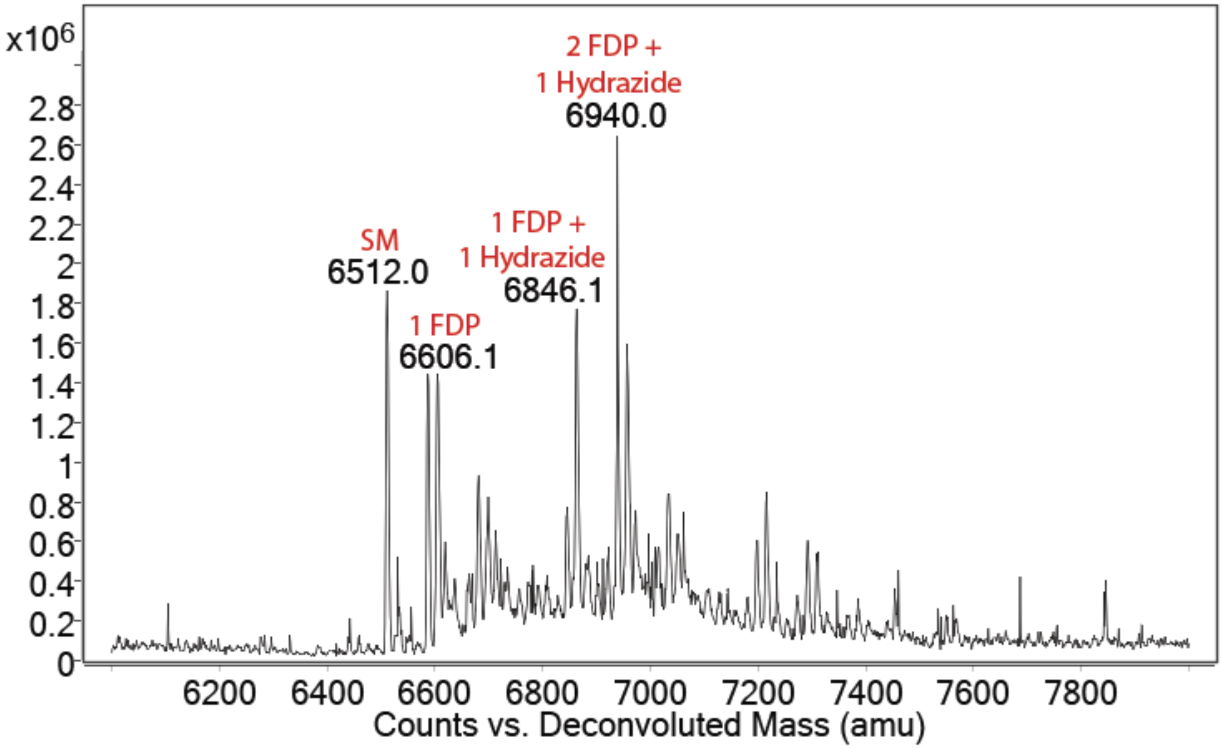

Reversal of Hydrazide Tagging of FDP Aprotinin

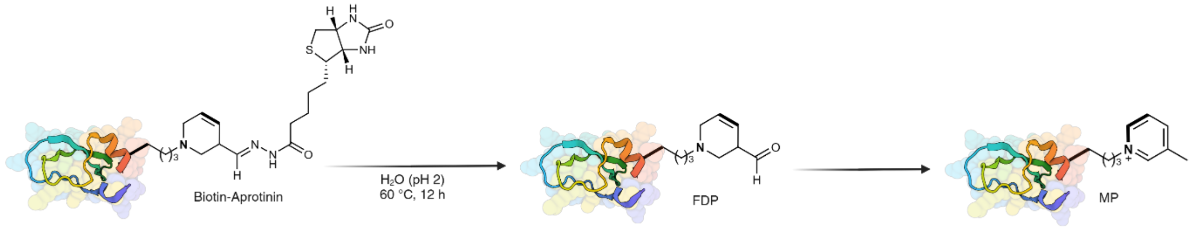

Hydrazide-modified FDP aprotinin (1 mg, 0.288 mM) was dissolved in 500 µL of H_2O and 1 M HCl was added to bring the pH of the solution to 2. Next, the mixture was heated at 60 °C for 12 hours, after which the crude reaction mixture was passed through Amicon Ultra 3 kDa spin-

concentrator and washed with H_2O (5 x 500 µL) to remove any molecule impurities. The labeled protein was redissolved in 0.1% formic acid in H_2O and analyzed using LC-MS. The conversion from the hydrazide-modified product to mass-boosted MP lysine was found to be 61%.

Deconvoluted MS for Reversal of Hydrazide Tagging for FDP Aprotinin

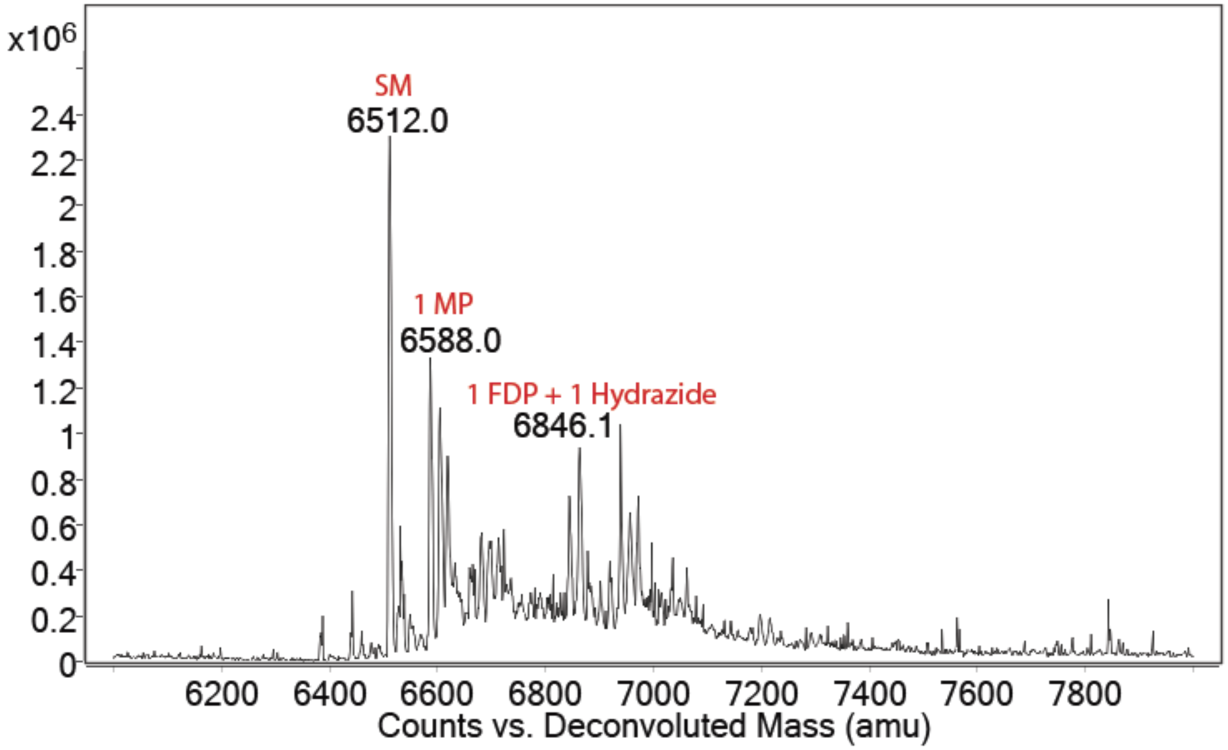

Proteome-Wide Mass-booting application of MP sites

1000 µg of lysate in NaP buffer pH 7.45 was treated with freshly prepared 250 µM of acrolein. The reaction was stirred at room temperature for 12 h. Upon completion of reaction, lysate was acetone precipitated and resuspended in 500 µL of NaP buffer pH 4.5 and treated with 200 µM of biotin hydrazide for 6 h, followed by acetone precipitation and resuspension in PBS buffer pH 7.4. Biotinylated lysates were incubated with streptavidin agarose bead for 1 h and eluted using 8 M guanidine hydrochloride (pH 1.5). Enriched proteins were desalted and converted to MP containing proteins through heating of lysates at 60 °C for 12 h. Heated and non-heated released proteins were incubated with Glu C cleavage Kit by Promega, followed by LCMS-MS and proteomics analysis. For gel analysis of eluates, samples were loaded on a Novex WedgeWell 4-20% Tris-Glycine gel. Gel was run in Tris-glycine running buffer at 180V. The gel was then stained with coomassie brilliant blue for 1 h and destained overnight.

Excel sheet of analysis is attached Supplementary Data 3.

Uncropped gel data for eluates of FDP and MP modified lysates

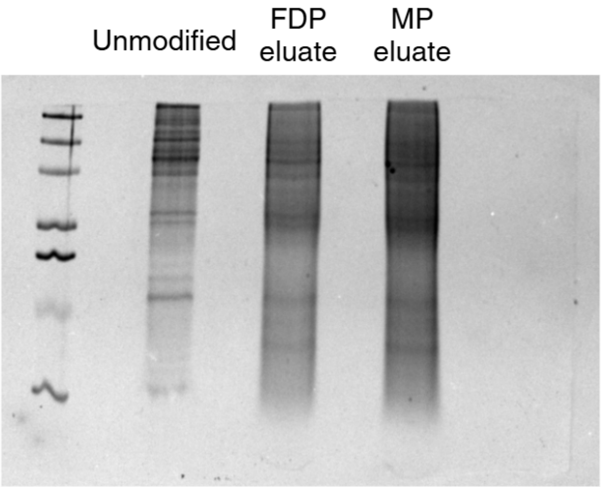

Fig. S25. Identification of protein crosslinking partners of FDP-modified proteins.

FDP Modification of Aprotinin

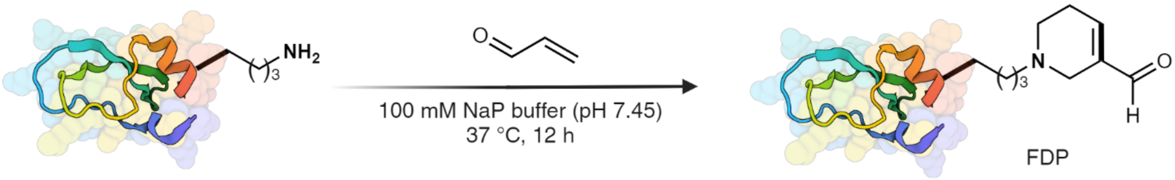

Aprotinin (2 mg, 0.614 mM) was dissolved in 480 µL of 100 mM NaP buffer (pH 7.45), and acrolein (0.164 µL, 4.91 mM) was added to the mixture from a freshly prepared stock solution (20 µL). The reaction was stirred at 37 °C for 12 hours, after which the crude reaction mixture was passed through Amicon Ultra 3 kDa spin-concentrator and washed with H_2O (5 x 500 µL) to remove the small molecule impurities. The labeled protein was redissolved in 0.1% formic acid in H_2O and analyzed using LC-MS. The conversion was found to be >95% with 30% 1 FDP modification, 41% 2 FDP modifications, and 24% for 3 FDP.

Modification	Mass	Conversion
1 FDP	6605.3 (**+94**)	30%
2 FDP	6700.0 (**+94**)	41%
3 FDP	6794.0 (**+94**)	24%

Deconvoluted MS of FDP aprotinin

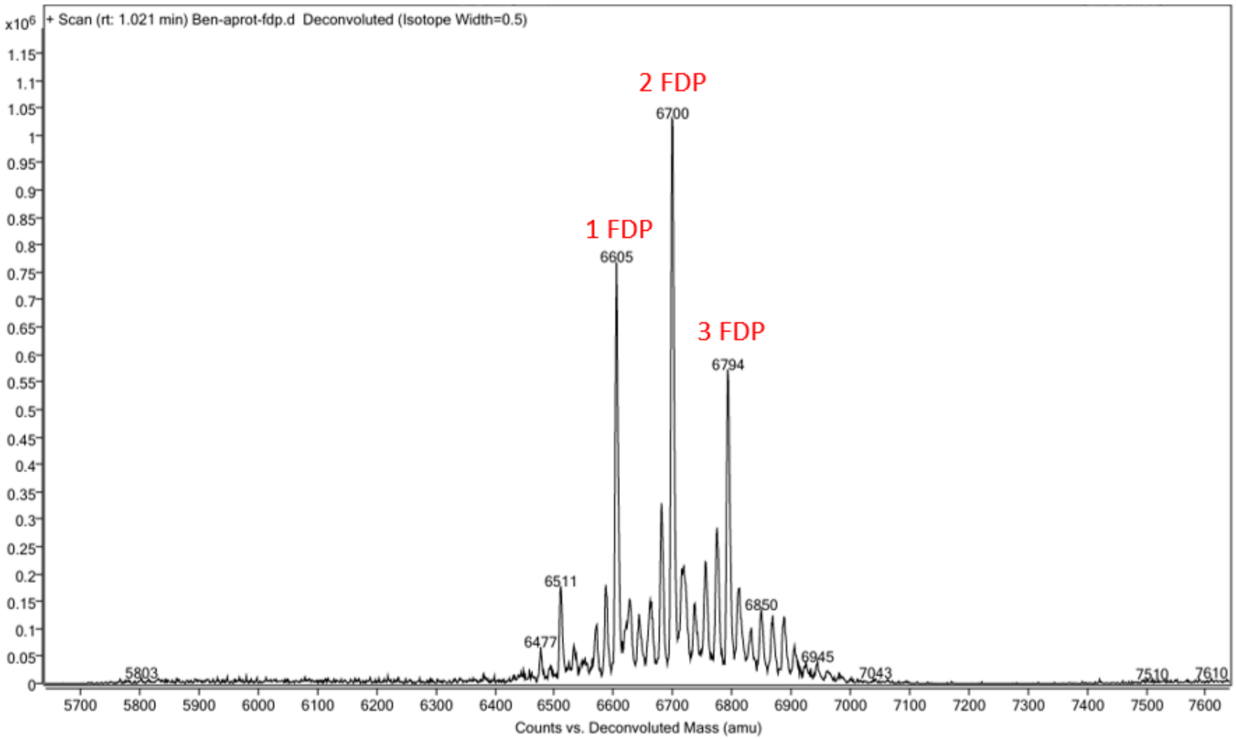

MS Validation of Reductive Amination Using FDP Aprotinin

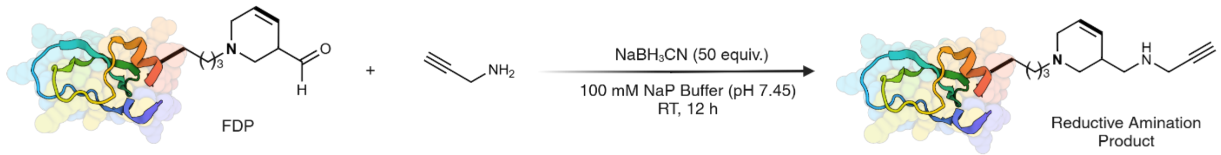

The FDP-modified aprotinin (1 mg, 0.307 mM) was dissolved in 460 µL of 100 mM NaP buffer (pH 7.45). $NaBH_3CN$ (0.165 mg, 3.07 mM) was added to the mixture from a freshly prepared stock solution (20 µL). Next, propargylamine (0.165 mg, 3.07 mM) was added to the mixture from a freshly prepared stock solution (20 µL). The reaction was stirred at RT for 12 hours, after which the crude reaction mixture was passed through Amicon Ultra 3 kDa spin-concentrator and washed with H_2O (5 x 500 µL) to remove the small molecule impurities. The labeled protein was redissolved in 0.1% formic acid in H_2O and analyzed using LC-MS. The conversion was found to be >95% to the reductive amination product with 53% 1 FDP with propargylamine attached and 47% 2 FDP with propargylamine attached.

Deconvoluted MS of FDP Aprotinin Reductive Amination Product

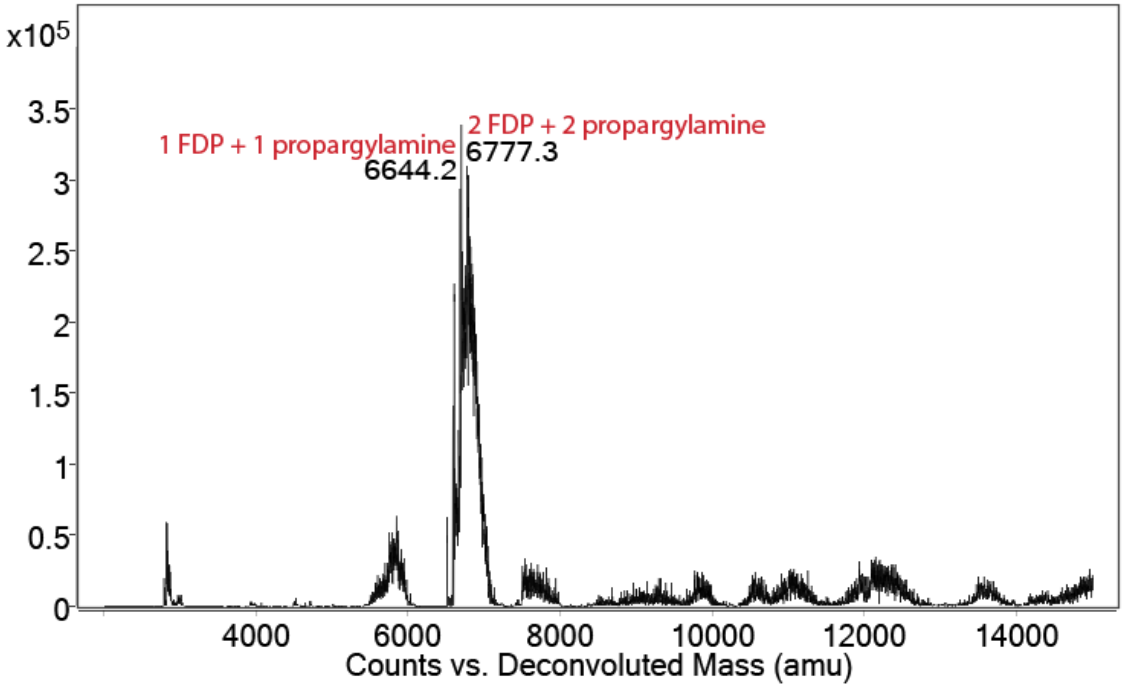

Incubation of FDP-aprotinin with chymotrypsin

2 mg of FDP-aprotinin was dissolved in 400 µL of 100 mM NaP buffer (pH 5.8), and 2 mg of α-chymotrypsin dissolved in 400 µL of NaP buffer was added to the mixture. The reaction was stirred at 37 °C for 1 hour, followed by the addition of 200 µL of 600 mM solution of sodium cyanoborohydride. Reaction mixture was stirred for 12 h and the crude reaction mixture was passed through Amicon Ultra 3 kDa spin-concentrator and washed with H_2O (5 x 500 µL) to remove the small molecule impurities. The labeled proteins were redissolved followed by analysis of proteins through in gel fluorescence imaging and coomassie blue staining. Samples were loaded on a Novex WedgeWell 4-20% Tris-Glycine gel. Gel was run in Tris-glycine running buffer at 180V. The gel was then stained with Coomassie brilliant blue for 1 h and destained overnight.

Uncropped gel data for aprotinin-chymotrypsin crosslinks:

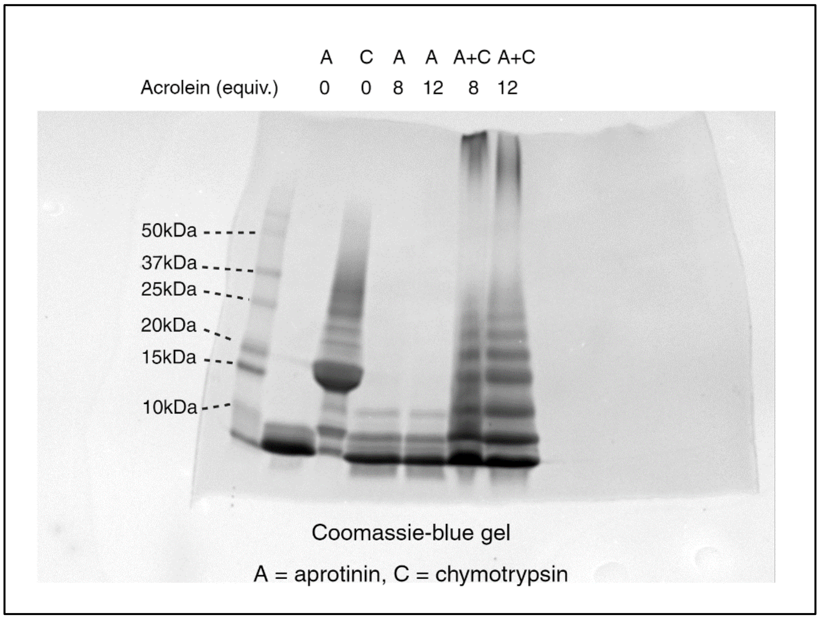

Identification of protein crosslinks

To identify the types of protein crosslinks, top sections of the gel (above chymotrypsin) and lower sections below chymotrypsin, were excised followed by in-gel digestion[6] and LCMS/MS analysis.

Peptide coverage mapping of top gel section

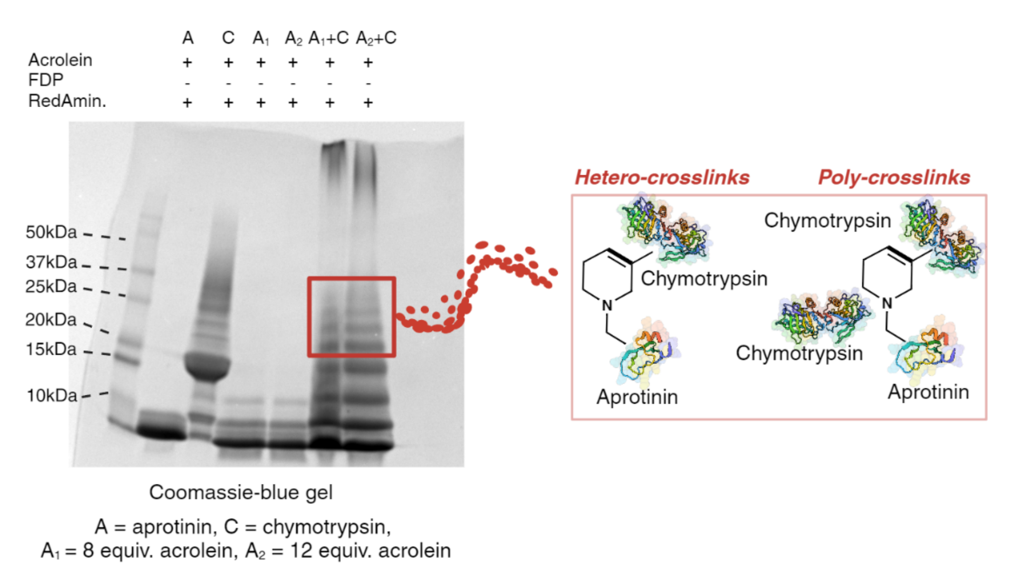

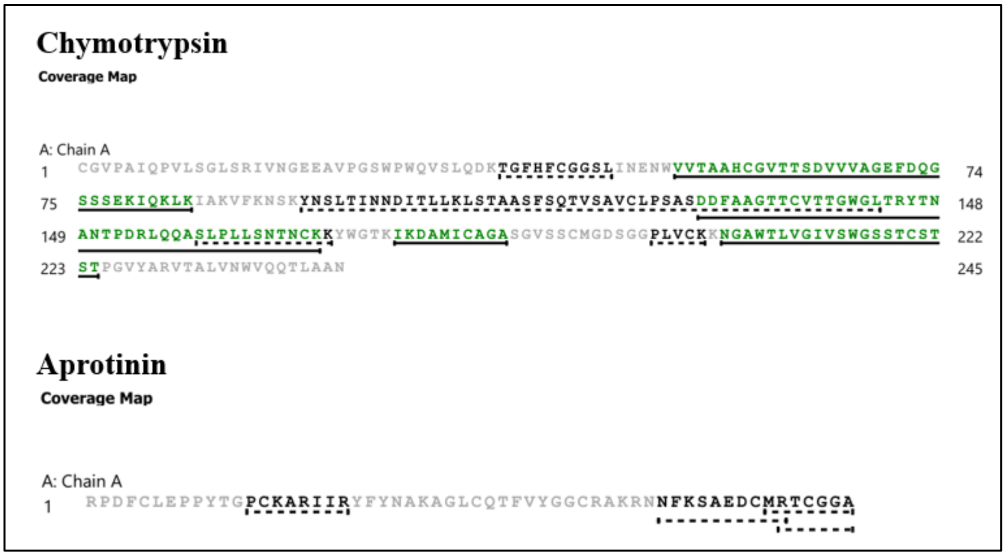

Peptide coverage mapping of lower gel section

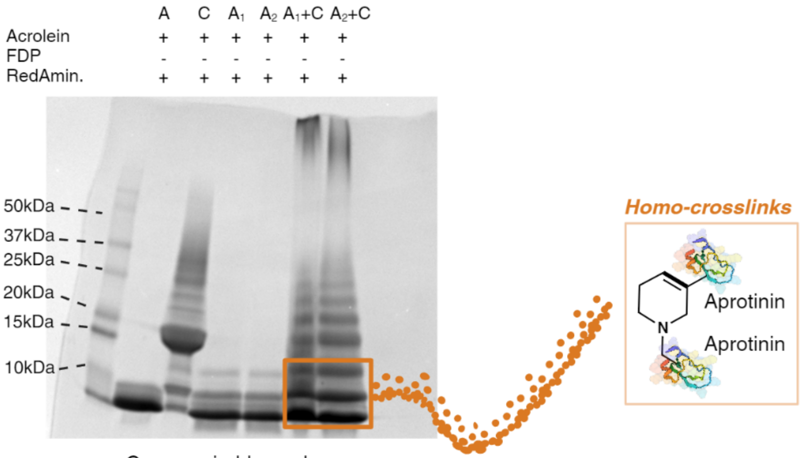

Coomassie-blue gel

A = aprotinin, C = chymotrypsin,
A_1 = 8 equiv. acrolein, A_2 = 12 equiv. acrolein

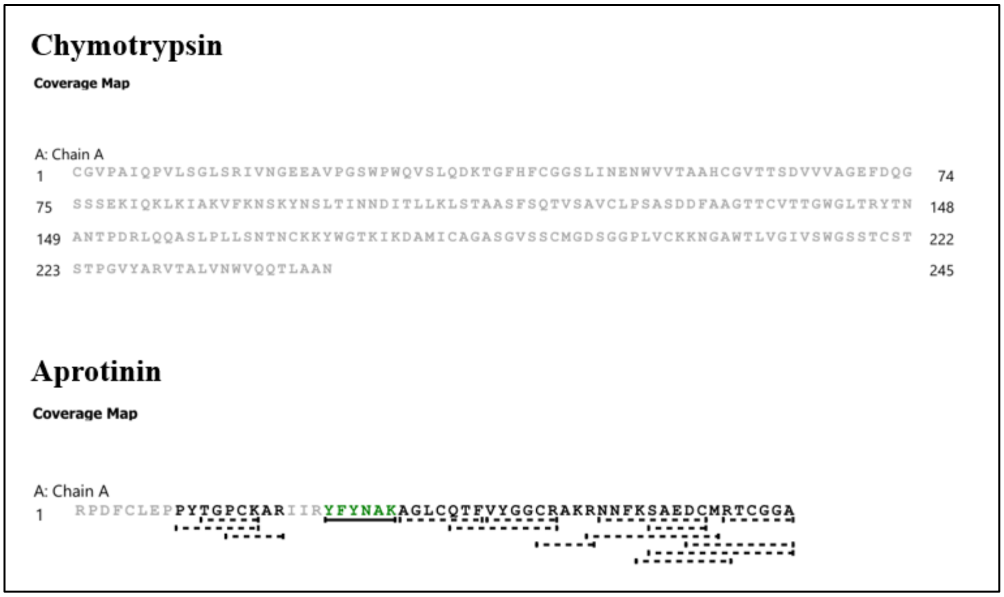

Evaluation of FDP-Lysozyme chicken crosslinking with chymotrypsin

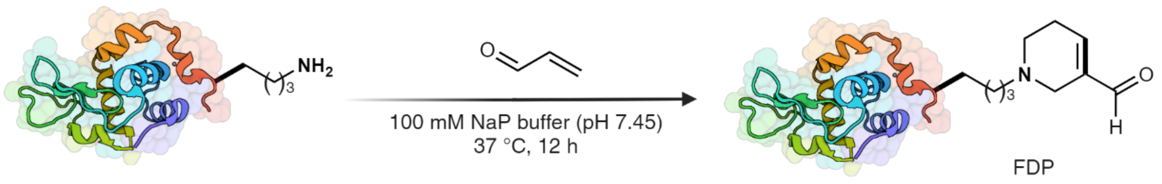

Lysozyme chicken (2 mg, 0.280 mM) was dissolved in 480 µL of 100 mM NaP buffer (pH 7.45), and acrolein (0.075 µL, 2.23 mM) was added to the mixture from a freshly prepared stock solution (20 µL). The reaction was stirred at 37 °C for 12 hours, after which the crude reaction mixture was passed through Amicon Ultra 3 kDa spin-concentrator and washed with H_2O (5 x 500 µL) to remove the small molecule impurities. The labeled protein was redissolved in 0.1% formic acid in H_2O and analyzed using LC-MS. The conversion was found to be 72% with 32% 1 FDP modification, 27% 2 FDP modifications, and 24% 3 FDP modifications.

Modification	Mass	Conversion
Unmodified	14305.3	18%
1 FDP	14399.4 (**+94**)	32%
2 FDP	14493.9 (**+94**)	27%
3 FDP	14588.1 (**+94**)	24%

Deconvoluted MS of FDP-Modified Lysozyme Chicken

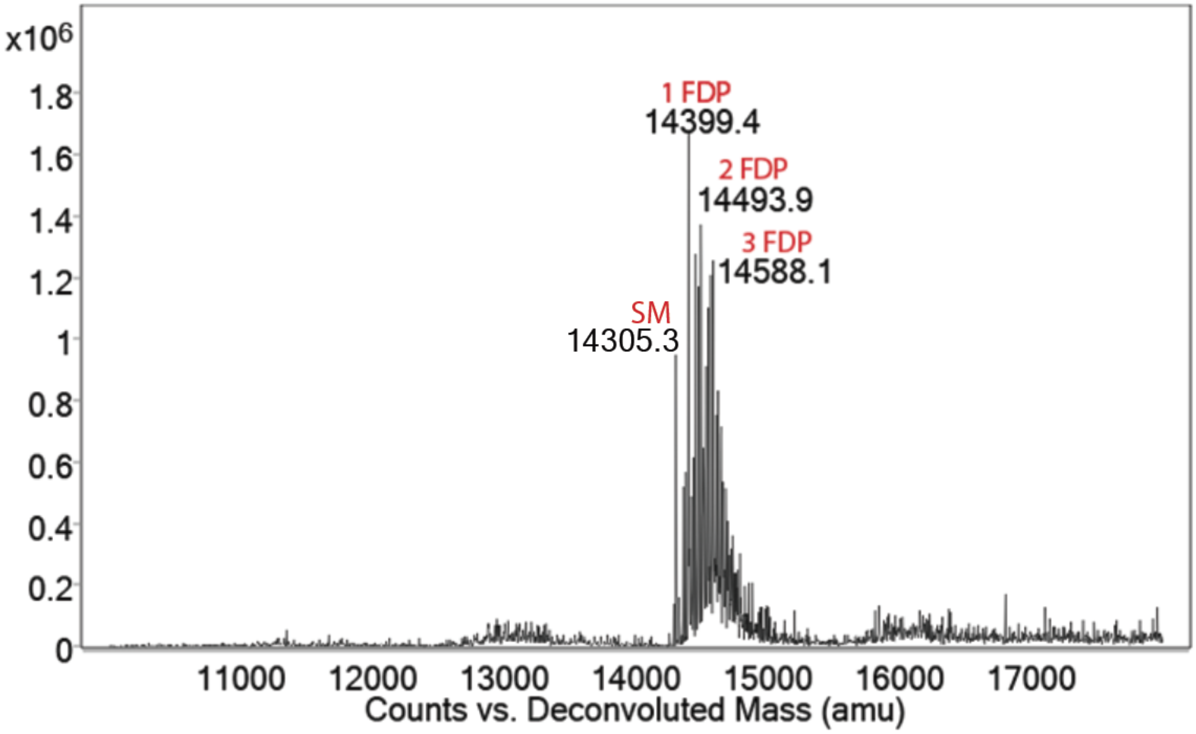

Incubation of FDP-Lysozyme with chymotrypsin

2 mg of FDP-Lysozyme was dissolved in 400 μL of 100 mM NaP buffer (pH 5.8), and 2 mg of α-chymotrypsin dissolved in 400 μL of NaP buffer was added to the mixture. The reaction was stirred at 37 °C for 1 hour, followed by the addition of 200 μL of 600 mM solution of sodium cyanoborohydride. Reaction mixture was stirred for 12 h and the crude reaction mixture was passed through Amicon Ultra 3 kDa spin-concentrator and washed with H_2O (5 x 500 μL) to remove the small molecule impurities. The labeled proteins were redissolved followed by analysis of proteins through in gel fluorescence imaging and coomassie blue staining. Samples were loaded on a Novex WedgeWell 4-20% Tris-Glycine gel. Gel was run in Tris-glycine running buffer at 180V. The gel was then stained with Coomassie brilliant blue for 1 h and destained overnight.

Identification of protein crosslinks

To identify the types of protein crosslinks, top sections of the gel (above chymotrypsin) and lower sections below chymotrypsin, were excised followed by in-gel digestion[6] and LCMS/MS analysis.

Peptide coverage mapping of top gel section

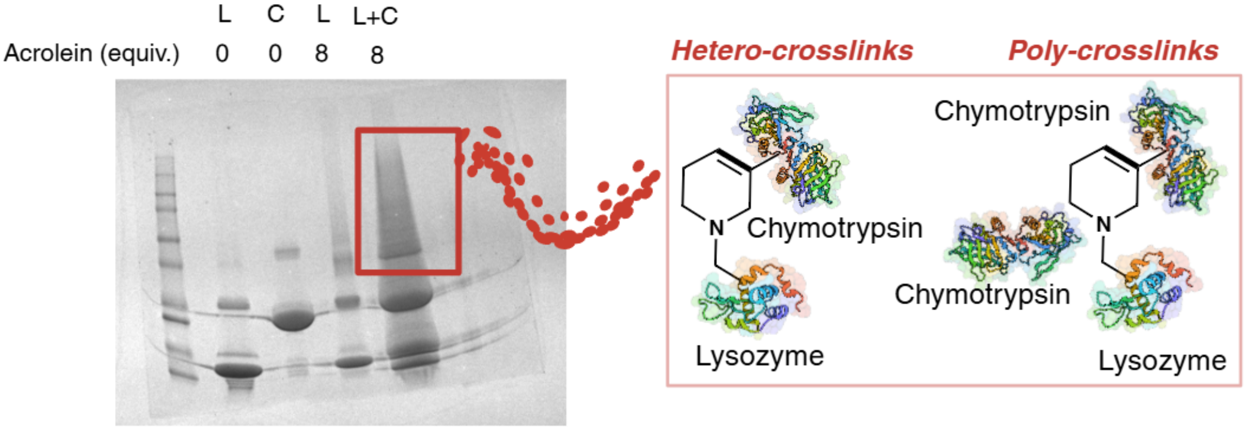

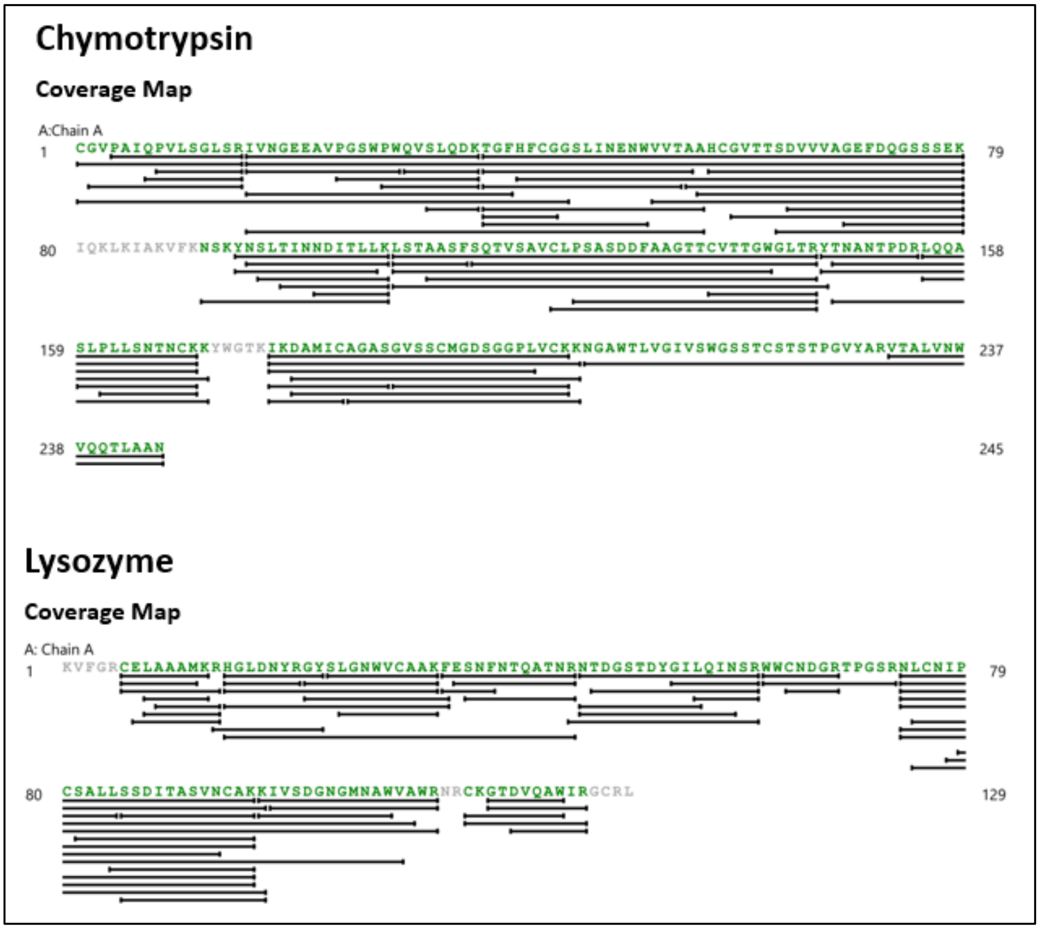

Peptide coverage mapping of lower gel section

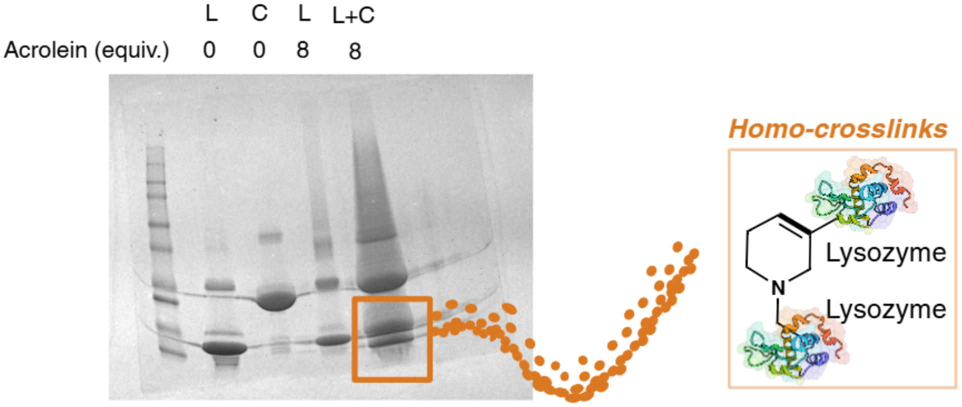

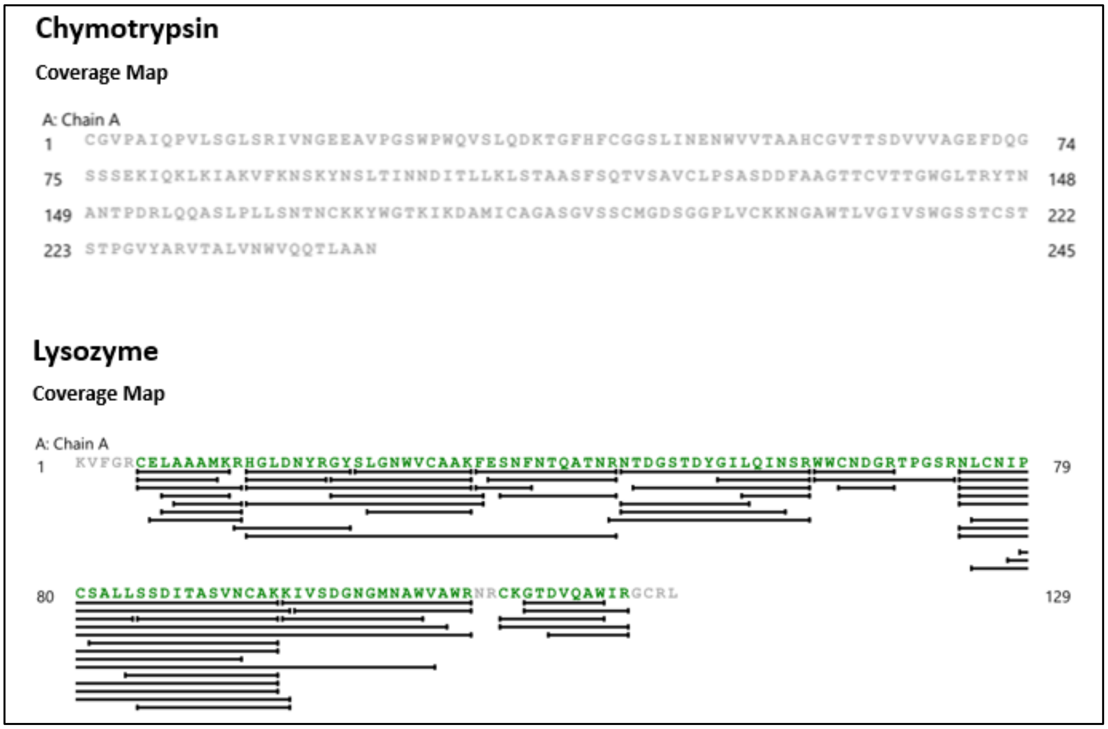

Fig. S26. Identification of protein binding partners of FDP-modified proteins from cell lysate.

Dose-dependent FDP Modification of Aprotinin and biotin conjugation.

4 batches of aprotinin (2 mg, 0.614 mM) dissolved in 480 µL of 100 mM NaP buffer (pH 7.45) were added different equivalence of acrolein (8, 12, 20, and 30). The reaction was stirred at 37 °C for 12 hours, after which crude reaction mixtures were passed through Amicon Ultra 3 kDa spin-

concentrator and washed with H_2O (5 x 500 µL) to remove the small molecule impurities. The labeled proteins were redissolved in NaP buffer (pH 5.8). Next, 1 equiv. of biotin-dPEG$_3$-oxyamine hydrochloride (1.42 mg, 6.28 mM) was added to each reaction mixture. The reaction was stirred at 37 °C for 8 hours, after which the crude reaction mixture was passed through Amicon Ultra 3 kDa spin-concentrator and washed with H_2O (5 x 500 µL). Control aprotinin-biotin sample was generated by reacting 2 mg of aprotinin in 500 µL of 100 mM NaP buffer with 2 equiv. of biotin-NHS ester. Reaction was stirred for 2 h and passed through Amicon Ultra 3 kDa spin-concentrator and washed with H_2O (5 x 500 µL).

Incubation of biotin tagged FDP-aprotinin samples with T47D lysates.

To each tube of biotin labeled FDP-aprotinin dissolved in 400 µL of NaP buffer (pH 5.8), was added 200 µg of T47D lysate and reaction stirred for 1 h, followed by the addition of 100 µL of 600 mM sodium cyanoborohydride solution. The reaction was stirred for 12 h and the crude reaction mixture was passed through Amicon Ultra 3 kDa spin-concentrator and washed with H_2O (5 x 500 µL). Proteins were redissolved in PBS and 20 µL of prepped Magbeads added to the reactions, followed by incubation (rocking) at 4 °C for 16 h. Samples were placed in a magnetic separation rack and washed 3 times with PBS. Next, 1X SDS sample buffer (50ul) was added to the beads followed by boiling for 10 minutes at 95 °C. Samples were loaded on a Novex WedgeWell 4-20% Tris-Glycine gel. Gel was run in Tris-glycine running buffer at 180V. The gel was then stained with Coomassie brilliant blue for 1 h and destained overnight.

Uncropped gel data for aprotinin-chymotrypsin crosslinks:

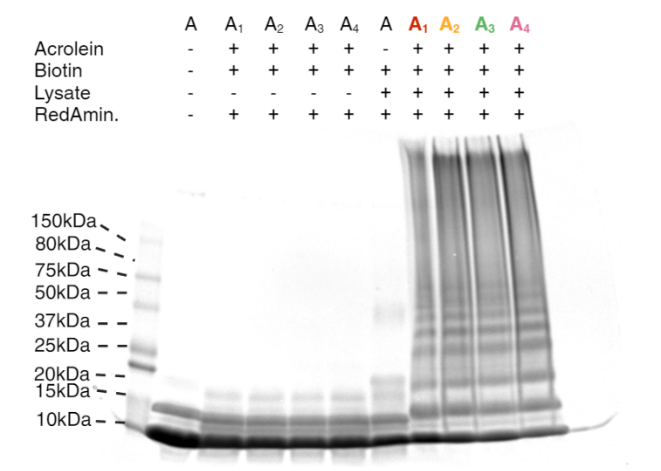

Coomassie-blue gel

A = aprotinin, A_1 = 8 equiv. acrolein, A_2 = 12 equiv. acrolein, A_3 = 20 equiv. acrolein, A_4 = 30 equiv. acrolein

Identification of protein crosslinks

To identify the types of protein crosslinks for each acrolein treated aprotinin samples, each well containing experimental samples (A1, A2, A3, A4) and control sample (A), were excised followed by in-gel digestion[6] and LCMS/MS analysis.

LC-MS/MS. Digested samples were resuspended in 0.1% formic acid (FA) in water, and the peptide amount was determined by Pierce™ Quantitative Peptide Assays & Standards (Thermo Fisher Scientific) according to manufacturer instructions. Samples from the site modifications experiment were injected into a nanoElute2 UPLC autosampler (Bruker Daltonics) coupled to a timsTOF Pro2 mass-spectrometer (Bruker Daltonics). The peptides were loaded on a 10 cm PepSep column (Bruker Daltonics). Chromatographic separation was achieved using a linear gradient over 90 min. The mass-spectrometer operated in positive polarity for data collection using a data-dependent acquisition (ddaPASEF) mode. The cycle time was 1.17 s and consisted of one full scan followed by 10 PASEF/MSMS scans. Precursors with intensity of over 2500 were selected for fragmentation and precursors with a target value over 20,000 were dynamically excluded for 1 min. Precursors below 700 Da were isolated with a 2 Th window and ones above with 3 Th. All spectra were acquired within an m/z range of 100 to 1700 and fragmentation energy was set to 20 eV at 0.6 1/K0 and 59 eV at 1.60 1/K0. The pulldown samples were loaded onto EvoTips and injected into an Evosep One system (EvoSep Biosystems) coupled to a timsTOF Pro2 mass-spectrometer (Bruker Daltonics). Samples were analyzed using the 20 SPD Zoom predefined gradient using a commercial analytical column (Aurora Elite, IonOpticks). Data acquisition was performed with the same settings, with the only expectation of a cycling time of 0.74 s, and consisted of one full scan followed by 6 PASEF/MSMS scans, accounting for the sharper peaks with this method.

Database search (MSFragger). MS raw files were searched FragPipe GUI version 20 with MSFragger (version 3.8) as the search algorithm. Protein identification was performed with the human Swisspot database (20′456 entries) with acetylation of N-terminus (+42.03670), and oxidation on methionine (+15.99492 Da) was set variable modification. Carbamidomethylation of cysteine residues (+57.021465 Da) was considered a fixed modification. Trypsin was set as the enzyme with up to two missed cleavages. The peptide length was set to 7–50, and the peptide mass range of 500–5000 Da. For MS2-based experiments, the precursor tolerance was set to 20 ppm and fragment tolerance to 20 ppm. Peptide spectrum matches (PSMs) were adjusted to a 1% false discovery rate using Percolator as part of the Philosopher toolkit (v5). For label-free quantification, match-between-runs were enabled. All downstream analysis was performed in R (version 2023.03.0). Individual samples were normalized to the mean of all quantified peptides.

Excel sheet of analysis is attached Supplementary Data 4. The mass spectrometry proteomics data (Data 2) generated in this study have been deposited to the ProteomeXchange Consortium via the PRIDE partner repository with the dataset identifier PXD054410. These experiments were performed with (n= 2 biological samples). Data is reported as Mean+SD.

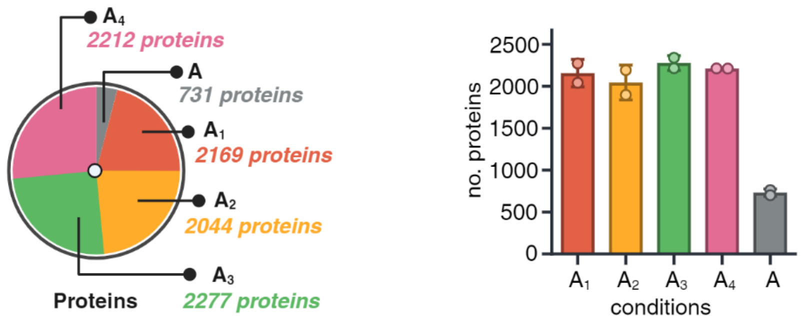

This experiment led to the identification of new protein partners of FDP-aprotinin, with 2169 proteins for 8 equiv. acrolein (A_1), 2044 proteins for 12 equiv. acrolein (A_2), 2277 proteins for 20 equiv. acrolein (A_3), 2212 proteins for 30 equiv. of acrolein (A_4), and 731 proteins for unmodified aprotinin. Number of proteins represents a mean value of protein count for n = 2 biological samples.

Fig. S27. Structural evaluation of FDP-aprotinin and unmodified aprotinin.

2 mg of wild-type aprotinin and FDP modified aprotinin were dissolved in 1 mL of water. 50 µL of each were used to perform Circular Dichroism analyses. Circular Dichroism was recorded on a Jasco-810 Spectropolarimeter. Samples were micro-pipetted onto a 50 µL Hellma Analytics quartz cell with a 0.1 mm path length (Model # 106-0.10-40). Spectra were measured by averaging three scans from 260-190 nm with a 0.2 nm data pitch and 100 nm s^{-1} scanning speed.

CD spectra of aprotinin and FDP-aprotinin

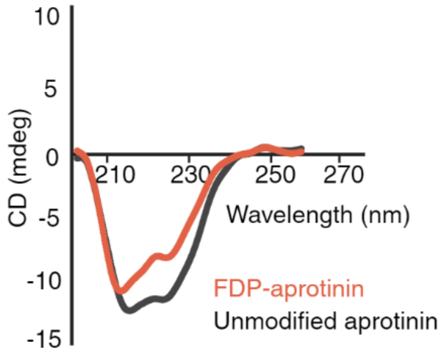

Fig. S28. Downstream analysis of FDP-aprotinin binders.

All downstream analysis was performed in R (version 2023.03.0). Individual samples were normalized to the mean of all quantified peptides. Analysis of peptide spectrum matches (PSMs)

for experimental samples (A_1, A_2, A_3, A_4) and control sample (A), were done using two sample t-test performed with FDR = 0.01. P-value cut off was set at <0.05

Excel sheet of analysis is attached Supplementary Data 5. The mass spectrometry proteomics data (Data 2) generated in this study have been deposited to the ProteomeXchange Consortium via the PRIDE partner repository with the dataset identifier PXD054410. These experiments were performed with (n= 2 biological samples).

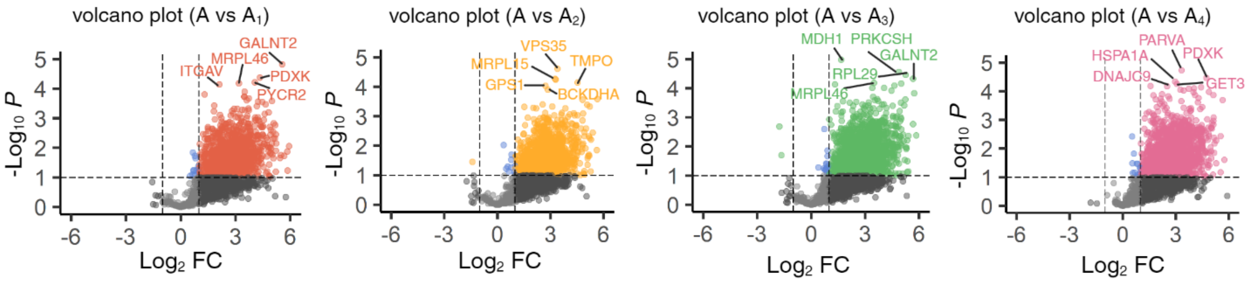

Gene	*P value*
GALNT2	1.51788E-05
PDXK	4.20901E-05
PYCR2	6.16736E-05
MRPL46	6.63566E-05
ITGAV	7.24788E-05

Gene	*P value*
VPS35	2.44373E-05
MRPL15	5.44138E-05
BCKDHA	5.66045E-05
TMPO	7.14679E-05
GPS1	9.32706E-05

Gene	*P value*
MDH1	1.06208E-05
PRKCSH	2.76696E-05
GALNT2	3.45797E-05
RPL29	4.76913E-05
MRPL46	6.55588E-05

Gene	*P value*
PARVA	1.86853E-05
PDXK	3.41588E-05
HSPA1A	4.58986E-05
GET3	5.52043E-05
RAB6A	6.59838E-05

Gene Ontology (GO) analysis

For Gene Ontology (GO) analysis, gene list of modified proteins was utilized as input in ShinyGO version 0.77.[7] FDR cut-off was set at 0.05%, with input and analysis species set to *H. sapiens*. All genes in the human genome were used as the enrichment background.

Inhibition of aprotinin-FDP crosslinking

0.5 mg of Biotin labeled FDP-aprotinin and 1 mg of aprotinin dissolved in 600 µL of NaP buffer (pH 5.8), was added 200 µg of T47D lysate and reaction stirred for 1 h, followed by the addition of 100 µL of 600 mM sodium cyanoborohydride solution. The reaction was stirred for 12 h and the crude reaction mixture was passed through Amicon Ultra 3 kDa spin-concentrator and washed with H_2O (5 x 500 µL). Proteins were redissolved in PBS and 20 µL of prepped Magbeads added to the reactions, followed by incubation (rocking) at 4 °C for 16 h. Samples were placed in a magnetic separation rack and washed 3 times with PBS followed by on-bead digestion using trypsin.

Excel sheet of analysis is attached Supplementary Data 6

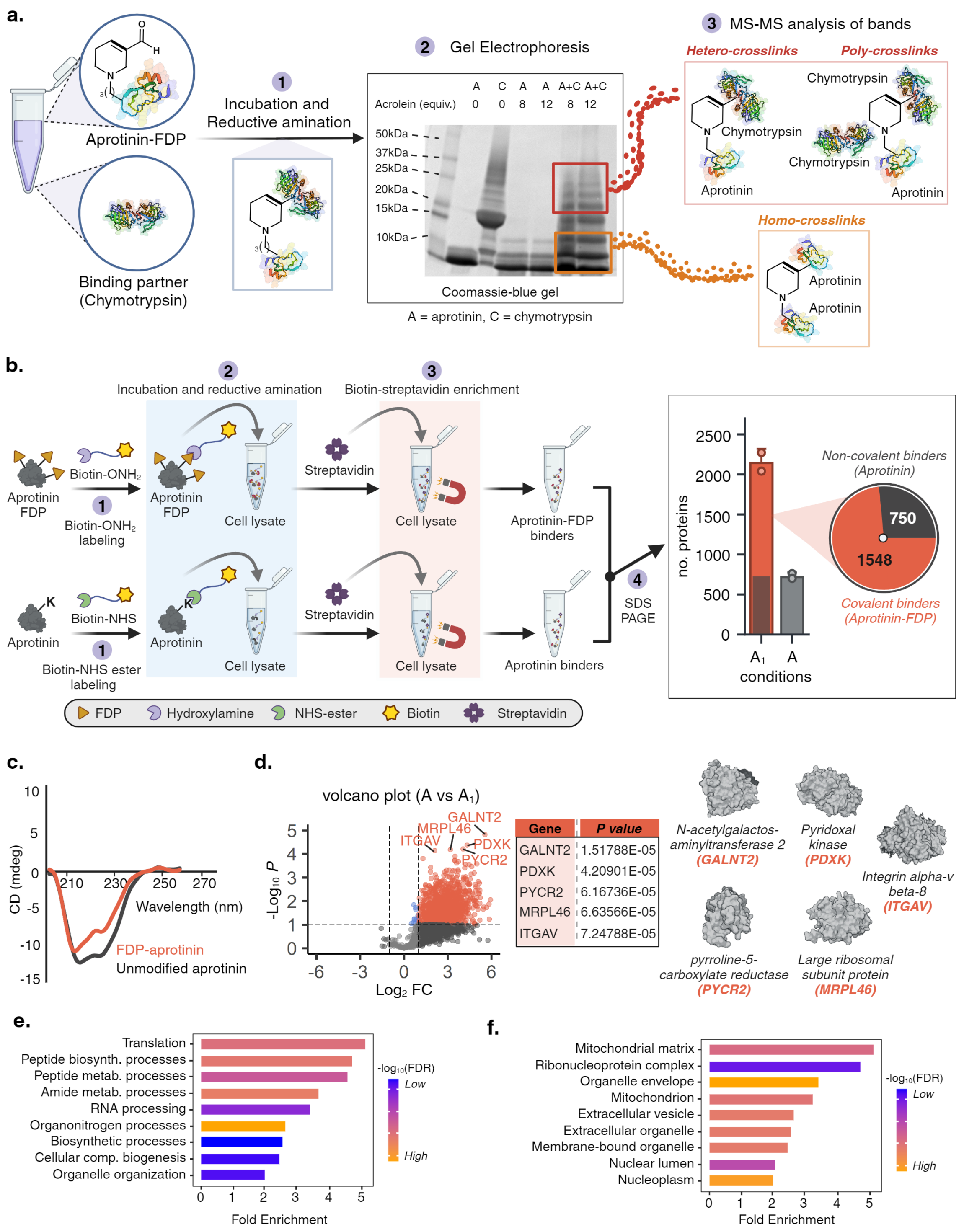

Capturing and identification of protein binding partners of FDP-modified proteins. a. Evaluation and capturing of FDP-aprotinin binding partner chymotrypsin by intermolecular crosslink between the FDP of aprotinin and lysine of chymotrypsin as analyzed by SDS PAGE. LCMS/MS analysis identified higher molecular weight crosslinks between aprotinin and chymotrypsin, in addition to lower molecular weight homo-crosslinks between FDP-aprotinin. **b.** Identification of new protein binding partners of FDP-aprotinin

from a cell lysate by enrichment and analysis. 1548 unique protein partners were observed with FDP-aprotinin with 750 proteins observed with unmodified aprotinin. Data is reported as Mean+SD. **c.** CD spectra of FDP-aprotinin and unmodified aprotinin confirms the minimal structural perturbation resulting from acrolein modification of aprotinin. **d.** Differential enrirchment analysis of FDP-aprotinin and unmodified aprotinin. Volcano plot of FDP-aprotinin mediated enriched proteins showed a significant enrichment of the top 5 most significant binders (GALNT2, PDXK, PYCR2, MRPL46, and ITGAV). Significance level is set at a p value of <0.05 and significant fold change cut-off set at 2 (Log_2FC of 1). Protein with -Log_{10}p-value > 1.30 and Log_2FC of > 1 are highlighted in red, while proteins with -Log_{10}p-value > 1.30 and positive Log_2FC of < 1 are highlighted in blue. **e.** Gene Ontology analysis of the molecular function and biological processes of enriched proteins showed significant enrichment of proteins associated with organonitrogen metabolic processes, amide and peptide metabolic processes. **f.** Enriched FDP-aprotinin proteins showed a broad diversity in spatio-temporal localization with a significant enrichment of proteins located within organelle envelops, nucleoplasm, and mitochondria. These experiments were repeated (n = 2 biological replicates) with similar results. Excel sheet of analysis is included as Supplementary Data 4 and 5. Source data are provided as a Source Data file. Figure 7, created with BioRender.com, released under a Creative Commons Attribution-NonCommercial- NoDerivs 4.0 International license" (Agreement number: EY272TGG5W).

References

1. Fields, G. B.; Noble, R. L. Solid phase peptide synthesis utilizing 9-fluorenylmethoxycarbonyl amino acids. *Int. J. Pept. Protein Res.* **1990**, 35, 161–214.

2. Furuhata, A.; Ishii, T.; Kumazawa, S.; Yamada, T.; Nakayama, T; Uchida, K. Nϵ-(3-Methylpyridinium)lysine, a Major Antigenic Adduct Generated in Acrolein-modified Protein. *J. Biol. Chem.* **2003**, 278, 48658–48665.

3. Mahesh, S.; Adebomi, V.; Muneeswaran, Z. P.; Raj. M. Bioinspired nitroalkylation for selective protein modification and peptide stapling. *Angew Chem. Int. Ed. Engl*. **2020**, 59, 2793-2801.

4. Shevchenko, A.; Tomas, H.; Havli, J.; Olsen, J. V; Mann, M. In-gel digestion for mass spectrometric characterization of proteins and proteomes. *Nat. Protoc.* **2006**, 1, 2856–2860.

5. Ge, S. X.; Jung, D.; Yao, R. ShinyGO: a graphical gene-set enrichment tool for animals and plants. *Bioinformatics* **2020**, 36, 2628–2629.

6. O'Shea, J. P.; Chou, M. F.; Quader, S. A.; Ryan, J. K.; Church, G. M.; Schwartz, D. pLogo: a probabilistic approach to visualizing sequence motifs. *Nat. Methods* **2013,** 10, 1211–1212.

7. Zhou, Y.; Zhou, B.; Pache, L.; Chang, M.; Khodabakhshi, A. H.; Tanaseichuk, O.; Benner, C.; Chanda, S. K. Metascape provides a biologist-oriented resource for the analysis of systems-level datasets. *Nat. Commun.* **2019**, 10, 1523.
